# Maize miRNA and target regulation in response to hormone depletion and light exposure during somatic embryogenesis

**DOI:** 10.3389/fpls.2015.00555

**Published:** 2015-07-22

**Authors:** Elva C. Chávez-Hernández, Naholi D. Alejandri-Ramírez, Vasti T. Juárez-González, Tzvetanka D. Dinkova

**Affiliations:** Departamento de Bioquímica, Facultad de Química, Universidad Nacional Autónoma de MéxicoMexico City, Mexico

**Keywords:** hormone depletion, maize, miRNA, photoperiod response, polyribosomes, somatic embryogenesis

## Abstract

Maize somatic embryogenesis (SE) is induced from the immature zygotic embryo in darkness and under the appropriate hormones' levels. Small RNA expression is reprogrammed and certain miRNAs become particularly enriched during induction while others, characteristic to the zygotic embryo, decrease. To explore the impact of different environmental cues on miRNA regulation in maize SE, we tested specific miRNA abundance and their target gene expression in response to photoperiod and hormone depletion for two different maize cultivars (VS-535 and H-565). The expression levels of miR156, miR159, miR164, miR168, miR397, miR398, miR408, miR528, and some predicted targets (*SBP23, GA-MYB, CUC2, AGO1c, LAC2, SOD9, GR1, SOD1A, PLC*) were examined upon staged hormone depletion in the presence of light photoperiod or darkness. Almost all examined miRNA, except miR159, increased upon hormone depletion, regardless photoperiod absence/presence. miR528, miR408, and miR398 changed the most. On the other hand, expression of miRNA target genes was strongly regulated by the photoperiod exposure. Stress-related miRNA targets showed greater differences between cultivars than development-related targets. miRNA/target inverse relationship was more frequently observed in darkness than light. Interestingly, miR528, but not miR159, miR168 or miR398, was located on polyribosome fractions suggesting a role for this miRNA at the level of translation. Overall our results demonstrate that hormone depletion exerts a great influence on specific miRNA expression during plant regeneration independently of light. However, their targets are additionally influenced by the presence of photoperiod. The reproducibility or differences observed for particular miRNA-target regulation between two different highly embryogenic genotypes provide clues for conserved miRNA roles within the SE process.

## Introduction

Maize (*Zea mays* L.) is one of the most important widely cultivated cereal crops, used as valuable source for human food, livestock feed, and raw material for the industry (Huang et al., 2002). Besides its agricultural and economic relevance, it has been a major model system in plant genetics and improvement. Common methods for maize transformation involve a process known as somatic embryogenesis (SE). SE begins with callus induction, a process characterized by re-organization and re-structuring of somatic cells in the presence of the synthetic auxin 2,4-D (Zimmerman, 1993). The second step of SE is plant regeneration. Plants can be regenerated when somatic embryos are depleted of external hormones and are exposed to photoperiod (Garrocho-Villegas et al., 2012). SE has been advantageously used in many plant species for clonal propagation, plant transformation and genetic improvement (Stasolla and Yeung, 2003).

Plant regeneration through maize SE was first reported by Green and Phillips (1975) using immature embryos as the initial explant. Various conditions for callus induction and plant regeneration have been tested since then, encountering the embryogenic potential highly dependent on explant and maize genotype used (Obert et al., 2009; Shen et al., 2012). The immature embryo's developmental stage and size, usually from 12 to 18 days upon pollination, appear as particularly relevant to generate embryogenic callus type II associated with high plant regeneration frequency over long subculture periods (Armstrong and Green, 1985). A few inbred lines have been reported with good embryogenic potential (Armstrong and Green, 1991; Jakubeková et al., 2012; Shen et al., 2012). Creole varieties VS-535 and H-565 (Costeño mejorado), derived from the germplasm of Mexican landrace Tuxpeño, are highly embryogenic and their plant regeneration frequency could be maintained for over 2 years of subculture (Garrocho-Villegas et al., 2012). However, while VS-535 has been successfully used for more than 15 years in tissue culture, H-565, released in 2008, has shown variable behavior in callus proliferation and plant regeneration responses through SE.

The mechanisms underlying gene activation/repression in the SE process have been poorly characterized (Salvo et al., 2014). Recently, a growing number of reports evidenced essential regulatory roles for microRNAs (miRNAs) in plant developmental and differentiation processes, including zygotic embryogenesis (Nodine and Bartel, 2010; Willmann et al., 2011), hormone signaling (Guo et al., 2005; Reyes and Chua, 2007) and stress response (Sunkar and Zhu, 2004). MicroRNAs are small 21–22 nt RNAs derived from longer precursors by Dicer-like (DCL) endonuclease activity and recruited to protein complexes by Argonaute (AGO) to target specific mRNA repression, either through degradation or translation inhibition (Llave et al., 2002; Li et al., 2013b).

In the past few years, several publications reported the miRNA presence/regulation during plant SE (Luo et al., 2006; Zhang et al., 2010, 2012; Chen et al., 2011; Wu et al., 2011, 2015; Li et al., 2012; Shen et al., 2012, 2013; Lin and Lai, 2013; Qiao and Xiang, 2013; Yang et al., 2013). Most of these reports used a high-throughput sequencing technology to compare the presence of conserved and species-specific miRNAs in the embryogenic callus (EC) before and during different stages of SE. Overall, an up-regulation of specific miRNAs was observed at particular stages of the somatic embryo differentiation. For most of the analyzed plant species, the proliferative embryogenic callus is characterized by low expression of many miRNAs related to flowering and leaf development, while stress-related miRNAs are increased in comparison to the original explant. Although miRNA expression patterns are altered upon callus induction, between embryogenic and non-embryogenic callus, as well as during SE and differentiation, the enriched or decreased miRNA species are particular for each of the plant species analyzed. Therefore, it is important to approach the miRNA regulation in the context of each plant SE system, taking into account the specific conditions and genotypes used in dedifferentiation as well as in plant regeneration induction.

Shen et al. (2013) analyzed by deep sequencing miRNA expression patterns upon callus induction from the maize inbred line 18-599R. They identified miR528, miR156, miR166, miR168, miR390, miR164, miR167, miR398, miR397, miR408, and miR319 as the most abundant during dedifferentiation. These miRNAs increased as the embryos were dedifferentiated into calli, with the exception of miR166 and miR167 that decreased. A degradome analysis indicated that most of their targets are involved in hormone signaling transduction pathways. In a previous study performed on VS-535-derived EC, we found that development-related miRNAs such as miR156, miR159, miR164 and miR168 decreased as the length of subculture increased, while stress-related miRNAs such as miR397, miR398, miR408, and miR528 remained highly expressed (Dinkova and Alejandri-Ramirez, 2014). However, the regulation on miRNA expression and their target mRNAs has not been explored in maize plant regeneration through SE. In this study, we addressed the impact of light exposure and hormone depletion as plant regeneration cues on maize miRNA levels and target gene expression. Taking into account the relevance of maize genotype on plant regeneration success through SE, we investigated how general the observed changes were by comparing two closely related cultivars, VS-535, and H-565.

## Materials and methods

### Callus induction and subculture

To induce embryogenic callus type II, immature embryos were collected at 15–18 days after pollination from two closely related maize cultivars, VS-535 and H-565. These cultivars have been derived from the Tuxpeño landrace germplasm (Márquez-Sánchez, 2008). Tuxpeño (VS-535) was previously shown to display high embryogenic potential during *in vitro* culture (Garrocho-Villegas et al., 2012), while Costeño mejorado (H-565) was introduced more recently (Márquez-Sánchez, 2008) and its behavior in tissue culture has been variable (unpublished data). The ears from the middle part of the husk (similar developmental conditions) were gently washed, first with 70% ethanol (1 min); then with 50% bleach solution (15 min); and three times with sterile deionized water. Next, the immature embryos were dissected and placed on a Petri dish with sterile deionized water and 1 g L^−1^ Cefotaxime. Thirty embryos (embryo axis side down) were placed per Petri dish on N6I medium (Supplementary Material, Data sheet 1) and maintained for 3 weeks under darkness at 25 ± 2°C. Upon this time, pro-embryogenic masses were selected for subculture on proliferation medium N6P (Supplementary Material, Data sheet 1). Every 3 weeks the embryogenic callus was subcultured on fresh N6P medium.

### Plant regeneration

To test the effects of hormone depletion and light on miRNA-mediated regulation during plant regeneration, the embryogenic callus was subjected to stage hormone depletion under darkness or light photoperiod (16 h light/8 h dark). During the first stage, the 2,4-D and kinetin concentrations were half-reduced and during the second stage (2 weeks after the first regeneration subculture) hormones were omitted from N6P. Samples were collected 1 week upon each subculture and stored at −70°C until used. Three biological samples were collected from each stage for RNA extraction. Regenerating callus was maintained on N6P devoid of hormones with every 2 weeks-subcultures until plantlets appeared under photoperiod. Plantlets were subcultured on MS medium (Murashige and Skoog, 1962; Supplementary Material, Data sheet 1).

### Total and polysomal RNA isolation

Total RNA was isolated from triplicates with Trizol reagent (Invitrogen, USA) according to the manufacturer's instructions. For polyribosome fractionation 5 g of embryogenic callus were pulverized in liquid nitrogen with mortar and pestle. The powder was suspended in 25 mL of lysis buffer (200 mM Tris-HCl pH 8.5, 50 mM KCl, 25 mM MgCl_2_, 2 mM ethylene glycol tetra-acetic acid and 0.05 mg mL^−1^ cycloheximide) and clarified by centrifugation at 15,000 rpm for 15 min. The supernatant was layered onto 4 mL sucrose cushion buffer (50 mM Tris-HCl pH 8.5, 25 mM KCl, 10 mM MgCl_2_, 60% sucrose and 0.05 mg mL^−1^ cycloheximide) and centrifuged at 45,000 rpm in a 75Ti rotor (Beckman Coulter, Mexico City, Mexico) for 3 h to concentrate ribosomes. The ribosomal pellet was suspended in 0.5 mL of DEPC water, layered onto 15–60% continuous sucrose gradient and centrifuged in SW-40 rotor (Beckman) at 36,000 rpm for 2.0 h. Fractionation and absorptivity at 260 nm of the gradient was performed in an Auto Densi-flow system (Labconco, Kansas City, MO, USA) connected to Econo UV Monitor EM-1 (BioRad). RNA was isolated from each fraction as described previously (Martinez-Silva et al., 2012). The RNA quality was tested by agarose gel electrophoresis and the concentration was measured with Nanodrop.

### Northern blot

Ten micrograms of total RNA were separated by electrophoresis on 15% polyacrylamide gels including 7 M urea, transferred to Hybond-N+ membranes (GE Healthcare Life Sciences, USA) and hybridized in ULTRAhyb®-Oligo hybridization buffer (Ambion, USA) with oligonucleotide probes for each miRNA (Supplementary Material, Table S1) end-labeled with [γ-32P]ATP (PerkinElmer, USA) by T4 polynucleotide kinase (NEB, USA).

### miRNA target prediction

Targets of zma-miRNAs were predicted by psRNATarget program (http://plantgrn.noble.org/psRNATarget/) with default parameters (Dai and Zhao, 2011), except for the Maximum expectation parameter which was set at five to get a higher prediction coverage. The selected genomic library for the target search was: “*Zea mays* (maize), transcript, NSF-funded Maize Genome Sequencing Project, Release 5a, filtered set.” Target gene descriptions were retrieved from biomart (Kasprzyk, 2011). Some of the predicted miRNA targets (Supplementary Material, Table S2) were chosen to evaluate their expression levels during plant regeneration (Table 1) according to the following criteria: (1) appropriate miRNA:target pairing in the seed region; (2) annotation of the target as protein coding transcript; and (3) experimental evidence as miRNA target in maize and/or in other plant species.

**Table 1:** **Analysis of miRNA targets**.

**miRNA**	**Target**	**Description**		**Hybrid**	**E**	**UPE**
miR156a-5p	*GRMZM2G126018_T01*	Squamosa promoter binding like transcription factor family; protein isoform 1 (SBP23)	miRNA	CACGAGUGAGAGAAGACAGU	1	18.45
				: : : : : : : : : : : : : : : : : : :		
			Target	GUGCUCUCUCUCUUCUGUCA		
miR159a-3p	*GRMZM2G139688_T01*	*Zea mays* GAMYB transcription factor	miRNA	GUCUCGAGGGAAGUUAGGUUU	2.5	17.184
				. . : : : : : : : : : : : : : : : : : .		
			Target	UGGAGCUCCCUUCACUCCAAG		
miR164a-5p	*GRMZM2G393433_T01*	CUC2; Putative NAC domain transcription factor superfamily	miRNA	CGUGCACGGGACGAAGAGGU	1	17.089
				: : : : : : : : : : : : : : : : : : :		
			Target	GCUCGUGCCCUGCUUCUCCA		
miR168a-5p	*GRMZM2G039455_T01*	Argonaute-like protein	miRNA	AGGGCUAGACGUGGUUCGCU	3.5	19.106
				: : : : : : : : : : : : : : : : :		
			Target	UCCCGAGCUGCACCAAGCCC		
miR397a-5p	*GRMZM2G146152_T01*	LAC2; Multicopper oxidase, Laccase, Cupredoxin	miRNA	GUAGUUGCGACGCGAGUUACU	3	23.155
				: . : : : : : : : : : : : : : : : : :		
			Target	CGUCAACGCGGCGCUCAACGA		
miR398a-3p	*GRMZM2G058522_T01*	SOD-4A; Superoxide dismutase; [Cu-Zn] 4AP (SOD9)	miRNA	GCCCCCGCUGGACUCUUGUGU	3.5	17.156
				: : : : : : . . : : : : : : : : : : :		
			Target	CGGGGGUCGCCUGAGAUCACA		
miR408a	*GRMZM2G384327_T03*	*Zea mays* gamma response I protein	miRNA	CGGUCCCUUCUCCGUCACGUC	3.5	21.422
				: : : : : : : : : : : : : : : : : :		
			Target	GCGAGAGAAGAGGCCGUGCAG		
miR528a-5p	*GRMZM2G106928_T01*	SOD-1A; Superoxide dismutase [Cu-Zn]	miRNA	GAGGAGACGUACGGGGAAGGU	2.5	11.294
				. : : : : : : : : : : : : . : : : : :		
			Target	UUCCUCCGCACGCCCUUUCCA		
miR528a-5p	*GRMZM2G107562_T01*	Plastocyanin-like Blue (type 1) copper ion binding protein	miRNA	GAGGAGACGUACGGGGAAGGU	2.5	18.145
				: : : : : : : : : : : : : : : : : : : .		
			Target	CUCCUCUGC-UGCCCCUUCCG		

*miRNA targets were predicted with psRNAtarget (http://plantgrn.noble.org/psRNATarget). The Expectation (E) value refers to the score of complementarity between each miRNA and target transcript. The UnPair Energy (UPE) value refers to the energy required for secondary structure melting around the target site. A lower UPE implies higher possibility for establishing a contact between miRNA and target mRNA, as well as for AGO-mediated cleavage*.

### qRT-PCR

Total RNA was extracted from two independent biological samples, treated with RQ1 RNase-Free DNase (Promega, USA) and reverse-transcribed using the ImProm-II™ reverse transcription system (Promega, USA). Each RNA sample was reverse-transcribed in two replicate reactions. Quantitative PCR (qPCR) was performed on the two biological samples for each genotype with three technical replicates per cDNA, using the Express GreenER qPCR reagents (GE Healthcare Life Sciences, USA) in a 7500 Real-time PCR System (Applied Biosystems, USA). Specific primers for qPCR were designed using Primer3Plus (Untergasser et al., 2007) and are available in Supplementary Material, Table S3. Relative abundance was calculated using the 2^−ΔΔ*Ct*^ method (Livak and Schmittgen, 2001). Target levels were normalized by rRNA 18S as internal housekeeping control and then compared to the levels found for the initial tissue (100% hormones, darkness). qRT-PCR fold-change data were summarized as Mean + Standard Error. The results obtained for each condition were compared performing a two-way (light × hormone)-ANalysis Of VAriance (ANOVA). The significance of mean difference within and between the groups was retrieved using Tukey Honestly Significance Difference (HSD) at *P* < 0.05.

## Results

### Plant regeneration through somatic embryogenesis

**Figure 1:**
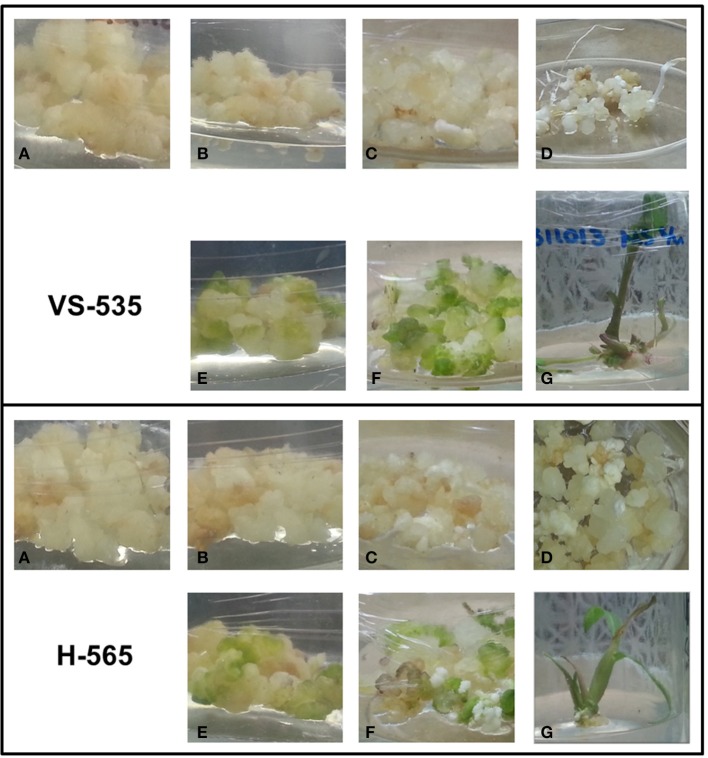
**Maize plant regeneration through somatic embryogenesis (SE)**. Embryogenic callus **(A)**, derived from VS-535 or H-565 maize cultivars, was subcultured on N6P medium with half the hormone (2,4-D and kinetin) concentration **(B,E)** and 2 weeks later on N6P medium without hormones **(C,F)**. Further subcultures were performed every 2 weeks until plant regeneration was achieved. Cultures were kept in darkness **(A–D)** or under a photoperiod of 16 h light/8 h dark **(E–G)**. Organogenesis was observed in darkness **(D)** while in photoperiod plantlets were formed **(G)**. Plantlets were subcultured on MS medium. Tissue was collected 1 week after the subculture.

Staged hormones depletion combined with light photoperiod promotes plant regeneration through SE in maize (Jakubeková et al., 2012; Garrocho-Villegas et al., 2012). Both signals exert important effects at molecular, biochemical, and physiological levels leading to a developmental switch from highly proliferating dedifferentiated tissues to fully differentiated plantlets. In agreement with this, clear morphological changes were observed upon hormone depletion in both, light photoperiod and darkness (Figure 1). Particularly, globular compact structures appeared at 50% hormone (2,4-D and kinetin) reduction in light and 0% hormones in darkness (Figure 1E and Figure 1C, respectively). The light presence rapidly (< 24 h) induced pigment deposition in the callus. For both conditions, the globular structures became elongated, but while under light they eventually resulted in plantlets, in darkness only organogenesis was appreciated (Figure 1G vs. Figure 1D). Fully developed plantlets were derived from 2 years-subcultured EC at 6–7 weeks upon hormones depletion for both, VS-535 and H-565. However, qualitative differences during plant regeneration were evident between these genotypes. For instance, the greenish color was more intense for VS-535 callus and a greater proportion of plantlets were obtained per gram of tissue for this variety (data not shown).

### Specific miRNA expression is mostly affected by hormones depletion

Previous studies in maize long-term subcultured EC indicated that miR156, miR159, miR164, miR168, and miR319 importantly reduce their levels in subcultures maintained for more than 18 months (Dinkova and Alejandri-Ramirez, 2014). On the other hand, the stress-related miR397, miR398, miR408 and miR528, become enriched upon callus induction and remain at high levels once the proliferation is established. This is in accordance with the proposal that specific miRNA expression in the undifferentiated EC associates with their proliferation maintenance, suggesting a switch from this expression pattern during plant differentiation (Luo et al., 2006). To evaluate which stimulus promotes miRNA expression changes, we tested the effect of hormone depletion in darkness or light for two independent maize 2 year-subcultured EC, owing that only in the presence of light plant regeneration could be achieved.

**Figure 2:**
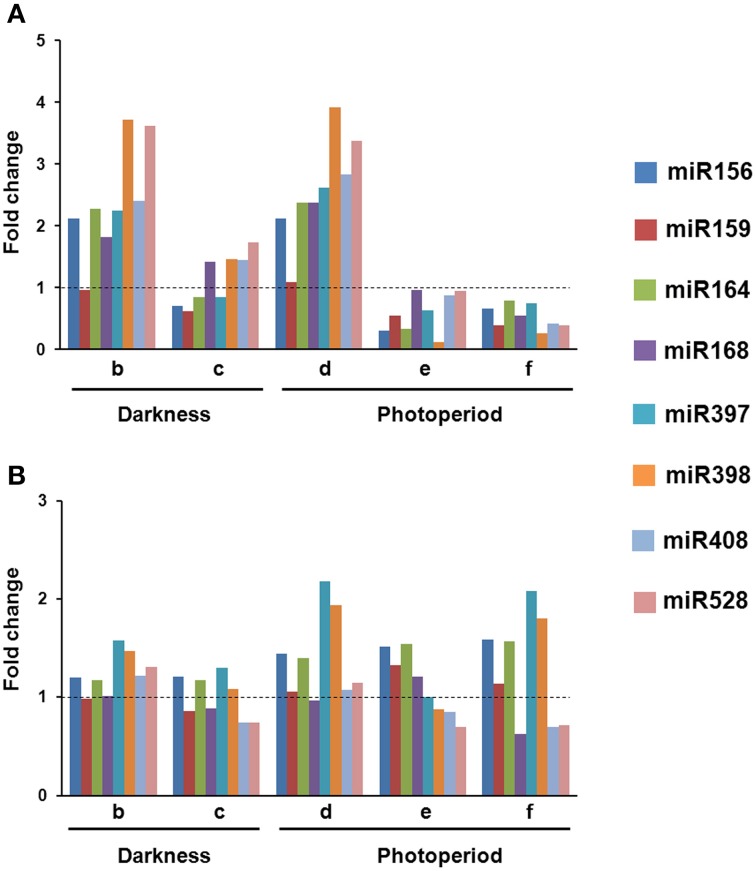
**Changes in miRNA levels occur upon hormone depletion under both, darkness and photoperiod conditions**. Northern Blot assays were performed to evaluate miRNA abundance during plant regeneration from somatic embryos in two maize cultivars: VS-535 **(A)** and H-565 **(B)**. Northern blot signals were acquired by densitometry and normalized according to 5S rRNA (Supplementary Material, Figure S1). The value corresponding to EC in 100% hormones (2,4-D and kinetin) was set as 1. Fold changes in 50% hormones (b, d); 0% hormones (c, e) and plantlet (f) with respect to 100% hormones were plotted on charts.

In VS-535-derived EC, most of the analyzed miRNAs increased in response to hormone half reduction (50%), regardless the presence of light (Figure 2A; lanes b and d). Although similar behavior was observed in H-565-derived callus, fold changes with respect to 100% hormones were distinct if compared to VS-535 (Figure 2B). For instance, at least two-fold increase in miR156, miR164, miR168 and miR408, was observed in VS-535, 50% hormones, while a modest 1.2-1.4-fold increase under the same conditions was evident for H-565 (Figure 2 and Supplementary Material, Figure S1). The next stage, 0% hormones, implied a sharp reduction for most miRNAs in VS-535, while in H-565 the reduction was lower or not observed depending on the miRNA. The levels of miR156 and miR164 decreased by three-fold (with respect to 50% hormones) for VS-535, but showed no change for H-565 (Figure 2, lanes c vs. b and e vs. d). Curiously, miRNA levels in the initial tissue (100% hormones) at 2 year-subcultures were also different between maize genotypes (data not shown). The effect of hormone depletion on miRNA expression was greater in the presence of light, at least for VS-535. miR398 decreased almost by 40-fold between 50 and 0% hormones in VS-535 against a modest two-fold decrease in H-565.

An exemption to most miRNA expression patterns was miR159, showing little or no change in response to hormones depletion in both cultures. In addition, while miR168 importantly increased (around two-fold) in 50% hormones for VS-535, it remained at similar levels for H-565. Regarding the differences observed between genotypes, it is important to stress out that tissues cultured *in vitro* are highly heterogeneous. Although for RNA analysis, EC was always sampled a week upon subculture, and visibly embryogenic differentiating tissues were selected, the heterogeneity inherent to each cultivar could not be avoided. The miRNA expression levels achieved during hormone depletion were maintained or further decreased in plantlets regenerated from VS-535 (Figure 2, lane f). Particularly, the stress-related miR397, miR398, miR408, and miR528 showed about two-fold reduction in VS-535 fully developed plantlets with respect to dedifferentiated tissues (100% hormones). However, in H-565-derived plantlets miR397 and miR398 remained expressed at higher levels.

### Light is a major stimulus affecting development-related miRNA target levels during maize SE

**Figure 3:**
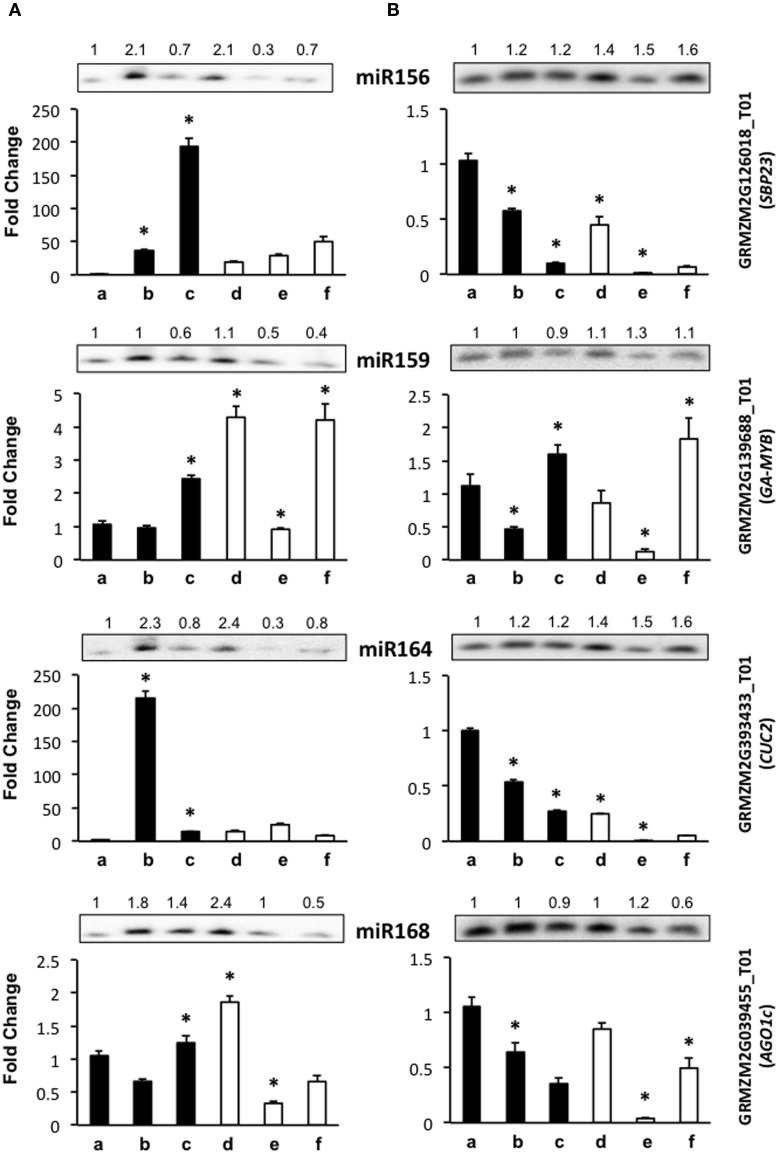
**The abundance of development-related miRNA targets is greatly affected by light presence during maize plant regeneration through SE**. Transcript levels of development-related miRNA targets were analyzed by qRT-PCR in the same samples as miRNAs for VS-535 **(A)** and H-565 **(B)**. The 18S rRNA was used as reference control and plotted values represent the expression of each mRNA relative to 100% hormones (a). Fold changes are shown accordingly for 50% hormones (b, d); 0% hormones (c, e) and plantlet (f). Filled bars represent darkness and empty bars, photoperiod. The corresponding miRNA Northern blots are shown at the top of each chart. miR156 target: *GRMZM2G126018_T01* (*SBP23*); miR159 target: *GRMZM2G139688_T01* (*GA-MYB*); miR164 target: *GRMZM2G393433_T01* (*CUC2*); miR168 target: *GRMZM2G039455_T01* (*AGO1c*). Error bars represent + Standard Error; *n* = 6. Statistically significant differences were identified using two-way ANOVA and Tukey as described in Methods at ^*^P < 0.05.

**Figure 4:**
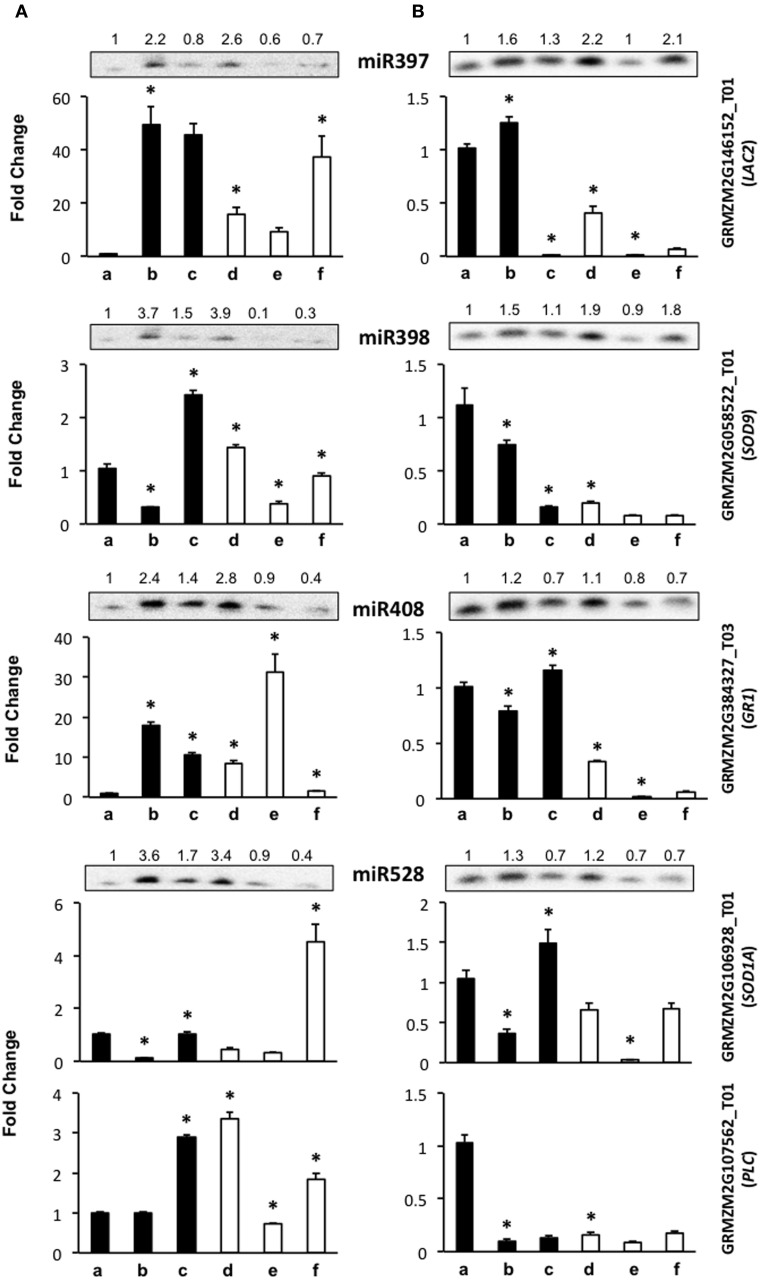
**The abundance of stress-related miRNA targets is dependent on the genotype during maize plant regeneration through SE**. Transcript levels of miRNA stress-related targets were analyzed by qRT-PCR in the same samples as miRNAs for VS-535 **(A)** and H-565 **(B)**. The18S rRNA was used as reference control and plotted values represent the expression of each mRNA relative to 100% hormones (a, set to 1). Fold changes are shown accordingly for 50% hormones (b, d); 0% hormones (c, e) and plantlet (f). Filled bars represent darkness and empty bars, photoperiod. The corresponding miRNA Northern blots are shown at the top of each chart. miR397 target: *GRMZM2G146152_T01* (*LAC2*); miR398 target: *GRMZM2G058522_T01* (*SOD9*); miR408 target: *GRMZM2G384327_T03* (*GR1*); miR528 targets: *GRMZM2G106928_T01* (*SOD1A*) and *GRMZM2G107562_T01* (*PLC*). Error bars represent + Standard Error; *n* = 6. Statistically significant differences were identified using Two-Way ANOVA and Tukey as described in Methods at ^*^P < 0.05.

Several of the miRNA targets analyzed in this study (*SBP23, GAMYB, CUC2*) encode for transcription factors known to participate in plant developmental switches including zygotic embryogenesis (Table 1). Others represent enzymes involved in plant stress response (targets of miR397, miR398, miR408, miR528) or the miRNA biogenesis pathway itself (miR168). According to this, we separated the results from qRT-PCR in development-related and stress-related miRNA targets (Figures 3, 4). The levels of *GRMZM2G039455_T01*, an AGO-like transcript also termed *AGO117* or *AGO1c* (Qian et al., 2011) were considered within the development-related targets, but according to its proposed function in miRNA biogenesis it actually corresponds to any of the subdivisions.

Development-related target mRNA levels displayed mostly contrasting behavior between darkness and photoperiod. The Squamosa Promoter Binding protein (SBP)-like transcript *GRMZM2G126018_T01* (*SBP23*) targeted by miR156 showed significantly higher levels upon hormone half reduction under darkness than in the presence of light (Figure 3, b–c vs. d–e). Under darkness, SBP23 inversely mirrored miR156 changes due to hormone depletion (Figure 3A, lanes b–c; and Figure 3B, lanes a–b). However, its levels were higher in this condition than in the presence of light. Such behavior correlates with the differentiation status of the tissue and with major influence of light on SBP23 expression. Since miRNA changes were essentially similar between darkness and light, but levels of the target displayed significant differences between these conditions, additional levels of regulation are likely operating to down-regulate *SBP23* during differentiation. Although we only tested one SBP-like target of miR156, 11 out of 26 SBP-like maize genes were predicted as targets for this miRNA, all of them exhibiting the same target sequence as *SBP23* (Supplementary Material, Table S2). Targeting of SBP-like transcripts by miR156 is highly conserved in plants (Rhoades et al., 2002; Xie et al., 2006; Gandikota et al., 2007) and has been reported as relevant for vegetative to reproductive phase changes and plastochron length (Wu and Poethig, 2006; Wang et al., 2008). For maize, it has been shown that miR156 gene family overexpression renders plants with increased number of leaves and delayed flowering (Chuck et al., 2007).

Similar to *SBP23*, a miR164 previously validated target, *GRMZM2G393433_T01* (NAC-domain transcription factor *CUC2* or *NAC107*, Zhai et al., 2013; Liu et al., 2014) was more abundant in darkness than light for either genotype (Figure 3, lanes a–c). However, while for H-565 the miRNA/target levels followed an inverse tendency with a consistent decrease of *CUC2* upon hormone removal and light exposure, for VS-535 a correlation was observed only under light (Figure 3A, lanes d–f). Global transcriptome analyses indicated that *CUC2* expression was restricted to immature inflorescence and ear in maize (Sekhon et al., 2011; Fan et al., 2014). Therefore, it is interesting that during SE this transcript was particularly abundant at 100% (H-565) or 50% (VS-535) hormones in darkness, both stages characterized by undifferentiated tissues (Figure 1).

Although miR159 and miR168 displayed slight changes in response to plant regeneration stimuli, their target levels (*GRMZM2G139688_T01, Giberellic Acid-responsive MYB, GA-MYB* for miR159 and *GRMZM2G039455_T01, AGO1c* for miR168) showed significant fluctuations at least for 0% hormones in darkness and photoperiod (Figure 3, lanes c and e). For both genotypes *GA-MYB* levels increased at 0% hormones in darkness, while strongly decreased for the same condition in light photoperiod. This correlates with the observation of drastic morphological changes in somatic embryos (Figure 1). miR159 is one of the most abundant miRNAs in maize EC (Shen et al., 2013) and its levels change the least (Figure 2). Nevertheless, an inverse correlation between *GA-MYB* and slight miR159 fluctuations were usually observed for H-565 and to a lesser extent for VS-535, suggesting that miR159 expression during SE might be important to keep *GA-MYB* levels low when not needed. Regarding normal maize plant development, *GA-MYB* expression has been detected in 24 h germinating seed and during seed development in the endosperm and pericarp, in meiotic tassel and in anthers (Sekhon et al., 2011). In addition, miR159-mediated regulation on *MYB* transcripts might depend on the tissue and developmental stage, at least in *Arabidopsis* (Woodger et al., 2003; Alonso-Peral et al., 2012).

miR168 is known as master regulator of the general miRNA pathway since it is required for fine-tuning the AGO1 levels in *Arabidopsis* (Vaucheret et al., 2006). In maize, at least five *AGO1* putative genes were previously identified (Qian et al., 2011) in contrast to only one *AGO1* encoding gene in *Arabidopsis*. *AGO1c* shows the greatest identity with the *Arabidopsis AGO1* homolog and has conserved the miR168 target sequence (Table 1). Similar to miR156 and *SBP23*, there was poor correlation between miR168 and *AGO1c* transcript levels. While the greatest increase in miR168 was observed for VS-535 at 50% hormones under photoperiod, *AGO1c* levels reached a two-fold increase in the same sample (Figure 3A, lane d). It was then decreased by four-fold at 0% hormones under the same condition when also miR168 decreased (Figure 3A, lane e). A greater reduction in this *AGO1c* transcript was observed for H-565, 0% hormones and photoperiod (25-fold) without accompanying changes in miR168 (Figure 3B, lane e). This suggests a complex regulation on miRNA biogenesis in maize, probably involving additional AGO1 isoforms during plant regeneration.

### Targets of stress-related miRNAs display essentially contrasting patterns between maize genotypes during SE

Stress-related miRNAs are highly expressed in the process of EC induction for one Chinese maize genotype (Shen et al., 2013). Stress has been also regarded as one of the main stimuli promoting dedifferentiation as well as SE. Major targets for stress-related miRNAs are copper proteins, multi-copper oxidases, superoxide dismutases and laccases (Jones-Rhoades and Bartel, 2004; Yamasaki et al., 2007; Abdel-Ghany and Pilon, 2008). Here we tested the mRNA levels for maize *GRMZM2G146152_T01* (laccase-like, *LAC2*) as target for miR397, *GRMZM2G058522_T01* (*SOD9*) as validated target for miR398 (Shen et al., 2013), *GRMZM2G106928_T01* (*SOD-1A*) and *GRMZM2G107562_T01* (plastocyanin-like protein, PLC) as targets for miR528, and *GRMZM2G384327_T03* (Gamma Response 1 protein, GR1), as previously predicted miR408 target (Li et al., 2013a). It is worth mentioning that although stress-related miRNAs have been identified as very highly expressed during germination, immature embryo dedifferentiation and stress-response, their target identification has remained elusive in maize. Our selection included both, validated and predicted targets according to the properties indicated in Table 1.

The level of miR397 target, *LAC2*, showed contrasting changes between VS-535 and H-565 genotypes throughout hormone depletion. For VS-535, it increased upon 50% hormone reduction under darkness or light (to a lesser extent), and remained unaltered after complete hormone removal (Figure 4A). On the contrary, for H-565 it was strongly reduced in 0% hormones, darkness or light (Figure 4B). For other stress-related miRNA targets, such as *SOD9* (miR398), *SOD1A* (miR528) and *GR1* (miR408), lower levels were also detected in H-565 than in VS-535, particularly in differentiating tissues under light (Figure 4A vs. Figure 4B, lanes e and f). There was poor correlation between miR397 and *LAC2* levels for either of the embryogenic cultivars, suggesting this transcript is regulated by additional mechanisms during plant regeneration. We observed an inverse correlation between miR398 (increase) and *SOD9* (decrease) upon 50% reduction in hormones for both cultivars in darkness, but only for H-565 in light (Figure 4A vs. Figure 4B). This indicates even for a validated target it is difficult to find strong miRNA/target correlation under light, particularly upon full hormone depletion. Similarly, *SOD1A* showed a clear inverse correlation with miR528 levels for both, VS-535 and H-565, in darkness but not in light (Figure 4, upper panels corresponding to miR528). It is interesting to notice that although SOD9 and SOD-1A catalyze the same reaction, their high and differential transcript expression supports a non-overlapping function in maize plant regeneration through SE. Similar to other stress-related miRNA targets, *GR1* levels were different between the tested maize genotypes (Figure 4). It increased upon hormone depletion in darkness or light for VS-535, while strongly decreased in the presence of light for H-565 (Figure 4A vs. Figure 4B). A correlation between *GR1* and miR408 was observed for H-565, but not for VS-535, suggesting this target regulation during SE could be genotype-specific.

miR528 is one of the most abundant, stress-related miRNAs in SE for maize (Shen et al., 2013), rice (Luo et al., 2006; Chen et al., 2011), and citrus (Wu et al., 2015). In our miR528 target prediction analysis, several copper-binding protein encoding transcripts, many uncharacterized genes and even transcription factors were found (Supplementary Material, Table S2). Taking into account the expected relevance for miR528-mediated regulation in SE, we tested, in addition to *SOD1A*, the expression levels of *GRMZM2G107562_T01* that codes for a plastocyanin-like protein (PLC). Unlike *SOD1A, PLC* showed a very weak correspondence with miR528 changes. However, consistent with other stress-related miRNA targets, its levels differed between VS-535 and H-565 throughout the hormone depletion stages (Figure 4A vs. Figure 4B, lower panels corresponding to miR528). Curiously, *PLC* levels were high in darkness, in spite of its predicted photosynthesis-related function (Abdel-Ghany and Pilon, 2008). In this regard, a light-independent induction of photosynthesis genes has been documented throughout embryogenesis stages (from globular to torpedo) in *Arabidopsis*. Genes involved in energy production comprised the largest up-regulated functional group, strongly biased toward components of the photosynthetic apparatus (Spencer et al., 2007). Taking this into account, high expression of PLC is probably required at early stages of SE.

### Polyribosomal distribution of miR528 in maize EC

**Figure 5:**
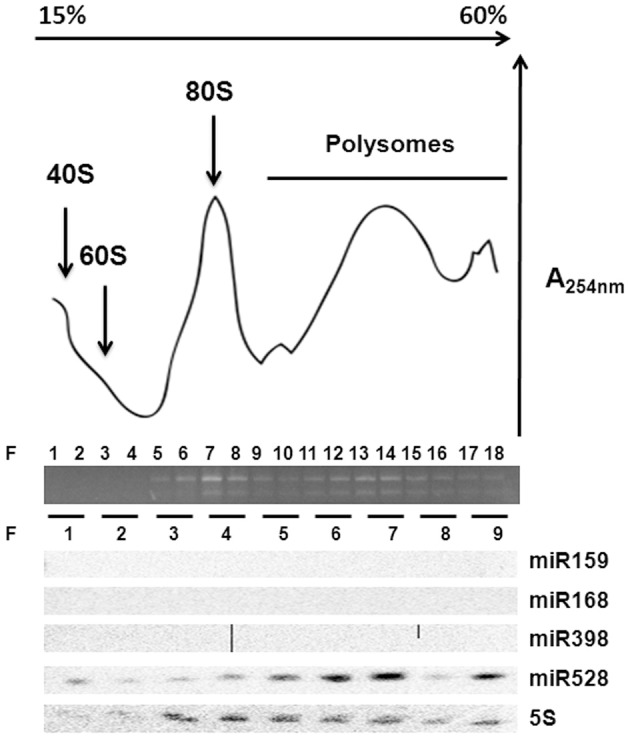
**miR528 is localized on polyribosomes**. Ribosomal profiling of two year-subcultured EC after sucrose density gradient fractionation. Total RNA abundance and integrity across fractions (F) was measured by absorbance at 254 nm and agarose gel electrophoresis. Northern blotting of selected miRNAs was performed upon RNA isolation from every two-fraction pools resulting in nine fractions. The 5S ribosomal RNA was used as control for the Northern blotting.

According to the lack of consistent correlation between levels of conserved development- or stress-related miRNAs and their targets (predicted or confirmed) during maize SE and plant regeneration, the regulation by additional mechanisms was evident. Primary regulation at transcription levels was already described for several of the analyzed targets (*SBP23, GA-MYB, CUC2*), particularly in response to developmental cues and hormones' presence. However, another level of regulation might be exerted by miRNAs at translation without affecting the transcript levels (Brodersen et al., 2008; Lanet et al., 2009; Iwakawa and Tomari, 2013). To assess the possible role of some conserved miRNAs at translation level, polyribosomal fractions were obtained from 2 years-subcultured VS-535 EC and the presence of miR159, miR168, miR398, and miR528 was analyzed (Figure 5). Only miR528 was detected in polyribosomal fractions (F 10–18) suggesting this miRNA could regulate some of its targets at translation level. In *Arabidopsis*, the presence of miR168 and miR398 in polyribosomes was previously demonstrated in 10 days-old seedlings (Lanet et al., 2009). However, we could not detect these miRNAs in maize polyribosomes from EC, although they had detectable levels in total RNA obtained from this tissue (Figure 2, Supplementary Material, Figure S1).

## Discussion

In the past few years, several publications reported conserved and species-specific miRNA levels during plant SE (Luo et al., 2006; Zhang et al., 2010, 2012; Chen et al., 2011; Wu et al., 2011, 2015; Li et al., 2012; Shen et al., 2012, 2013; Lin and Lai, 2013; Qiao and Xiang, 2013; Yang et al., 2013). These studies found that miRNA patterns change upon callus induction, between embryogenic and non-embryogenic callus, as well as during SE and differentiation in a plant species-dependent fashion. For example, while miR171, miR390, and miR398 are preferentially expressed in EC before induction of plant differentiation in rice (Luo et al., 2006); these miRNAs are increased during the differentiation process in citrus (Wu et al., 2011). Even miRNAs from the same family, i.e., miR156a and miR156b, may display differential expression patterns during the SE differentiation process, as demonstrated in a recent study on *Larix leptolepsis* (Zhang et al., 2012). Hence, the current knowledge based on global miRNA approaches highlights the relevance of exploring particular miRNA landscapes and their target regulation in the context of species-specific SE conditions.

### miRNA expression in maize SE

Separated analysis on hormone depletion and light effects during maize SE indicated that miRNA expression patterns are affected mostly by hormones, rather than the light presence. By analyzing the process in two independent embryogenic genotypes we expected to confirm whether the observed miRNA changes are inherent to the process rather than the cultivar. However, we noticed a great influence of the genotype, first on the initial miRNA level appreciated in the 2 years-subcultured EC, and second on the degree of changes registered during hormone depletion. In this sense, miRNA expression regulation resulted more dramatic in VS-535 than in H-565. Strikingly lower levels were detected for all tested miRNAs in VS-535 at 0% hormones and regenerated plantlets under light, whereas H-565 maintained the presence of some of the stress-related miRNAs (miR397 and miR398) higher. A recent study explored miRNA differential expression during immature embryo dedifferentiation in the presence of 2,4-D using a highly embryogenic maize inbred line 18-599R (Shen et al., 2013). The authors suggested miR164, miR169, miR528, and miR529 might be primarily participating in the process of EC induction through the regulation of targets involved in auxin and gibberellin signaling. However, other miRNAs, significantly up-regulated in the dedifferentiation process, were miR156k, miR168, miR397, miR398, and miR408. We found the same miRNAs transiently increased by the reduction of hormones concentration in half during plant regeneration. However, in the absence of hormones their levels were reduced. These observations support the occurrence of specific miRNA expression readjustments in embryogenic tissues in response to hormone changes in the environment.

One important question is whether the high concentration of specific miRNAs associates with the embryogenic potential of the callus. Shen et al. (2013) found that the initial increase in miRNAs was either enhanced or maintained during the dedifferentiation process. However, in maize EC subcultured for long periods (up-to 2 years) we have found a gradual reduction in miR156, miR164 and miR168 levels without impairment on the callus embryogenic potential (Dinkova and Alejandri-Ramirez, 2014). In agreement, the data presented here support an initial burst on certain miRNA levels preceding their further decrease during maize SE. Hence, miRNA expression response to hormone changes could be a major factor impacting on the embryogenic potential of maize cultivars during both, dedifferentiation and plant regeneration.

### Correlation between miRNA and target levels in maize SE

miRNAs are known to regulate their target mRNAs by degradation, translation inhibition or both. In plants, an inverse correlation between miRNA and target levels is commonly observed, suggesting that mRNA degradation is the preferred regulatory mechanism. However, global miRNA and degradome sequencing data have shown that not always the degradation products of predicted or even validated targets could be detected in the libraries (Shen et al., 2013; Wu et al., 2015). Conversely, these studies have identified novel targets for conserved miRNAs such as miR156 and miR164, or known targets with novel miRNA sites. An increasing number of reports have also revealed the lack of inverse relationship between miRNAs and their targets, depending on the tissue or the process analyzed (Brodersen et al., 2008; Wu et al., 2011; Alonso-Peral et al., 2012).

**Figure 6:**
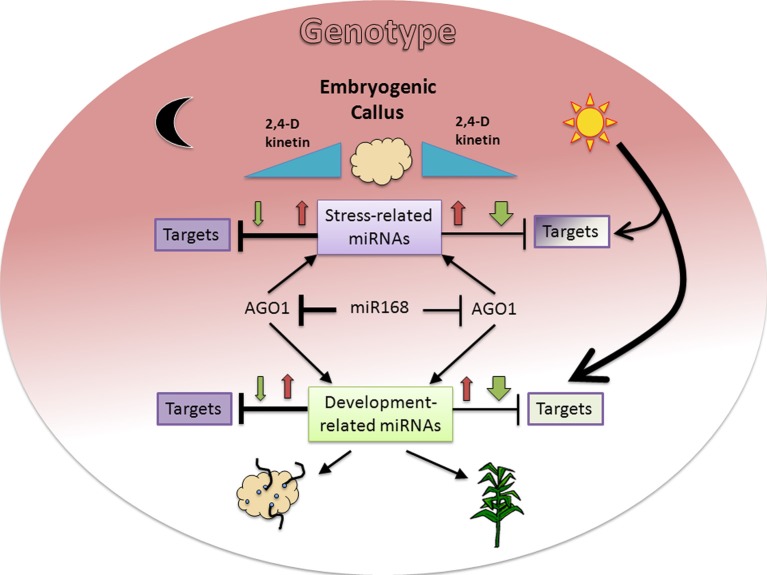
**Proposed model for miRNA and target regulation responses to environmental and genotype dependent cues in maize plant regeneration through SE**. Hormone (2,4-D and kinetin) depletion (blue triangles) affects miRNA expression patterns with initial increase (red arrows) at 50% and further decrease (green arrows) at 0% hormones in darkness or light presence. The presence of light induces a greater decrease of miRNAs in the absence of hormones, resulting in lesser effect on their targets. However the same condition represents a major regulation on target expression, particularly for development-related targets (bigger arrow). In addition to environmental cues, the genotype (colored background gradient) has an important role on both, miRNA and target regulation with greater effect on stress-related miRNAs and undifferentiated tissues (dark color). A regulatory miR168-AGO1 loop might also participate in the differential regulation observed between darkness/light and genotypes. The fading background color toward development-related miRNA targets and the physiological outcomes for either darkness (organogenesis) or light (plant regeneration) is consistent with the embryogenic potential of both maize genotypes assayed in this study.

Here we found the expected inverse correlation between a miRNA and its predicted and/or validated target is highly dependent on environmental (light presence) and internal (genotype) signals during maize plant regeneration through SE (Figure 6). Major differences were observed for development-related miRNA target levels between genotypes under darkness, but not under light where plant regeneration took place. On the other hand, inverse relationship between miRNA/target levels was more easily found in darkness than in light. Therefore, for most of the analyzed targets we propose there is a major influence of additional regulation under a photoperiod, making it difficult to appreciate the effect of miRNAs. On the other hand, increments of both, miRNA and target, upon hormone 50% reduction (e.g., miR156, miR164, miR168) suggest miRNA up-regulation might be required to control the levels of transcripts induced during SE.

Accessing miRNA-mediated regulation through quantitative transcript evaluation has been widely used in plants, particularly during SE (Li et al., 2012; Yang et al., 2013; Wu et al., 2011, 2015). However, an inverse miRNA/target relationship has not always been observed, even in the presence of miRNA-mediated degradation evidence (degradome or 5′RACE fragments). Our finding on miR528 distribution in maize EC polysomal fractions supports the notion that target regulation is probably exerted at multiple levels depending on the developmental process, miRNA, and analyzed target. In agreement, we observed such expected inverse correlation between miR528 and *SOD1A* target, but not for *PLC* target. Therefore, for future studies, it would be relevant to include miRNA-target evaluations at protein in addition to transcript levels.

### Physiological relevance of development-related miRNA regulation in maize SE

miR156 and miR164 have been found as SE-abundant miRNAs in several species, including maize (Li et al., 2012; Shen et al., 2013; Dinkova and Alejandri-Ramirez, 2014; Wu et al., 2015). The miR156-mediated suppression of *SBP* transcripts is probably required for early SE, as demonstrated for *Arabidopsis* zygotic embryogenesis (Nodine and Bartel, 2010). Similarly, miR164 initial increase during SE is consistent with maintaining low levels of its *CUC2* target during plant regeneration under light. In plants, conserved miR164 targets are NAC transcription factors (Mallory et al., 2004). The NAC family of proteins includes NAM, ATAF1-2, and CUC2. Proteins belonging to the NAC family are involved in many plant developmental processes, such as flowering, embryogenesis, senescence, auxin signaling, secondary wall thickening and others (Fan et al., 2014). NAM was reported as related to the shoot apical meristem and primordium formation in *Petunia hybia* (Souer et al., 1996) and CUC2 has been involved in *Arabidopsis* shoot apical meristem development (Aida et al., 1997). This context is consistent with the observed *CUC2* reduction in the absence of hormones and in fully differentiated tissues. Therefore, although miR164 and miR156 targets might display contrasting behavior in undifferentiated tissues (darkness, hormones' presence) between maize genotypes, their expression regulation is apparently required for plant regeneration through SE, regardless the genotype (Figure 6).

One possibility underlying the contrasting behavior of miRNA targets between darkness and light could be their transcription responsiveness to photoperiod. Another is a differential function of miRNA-mediated silencing pathways depending on light presence/absence during SE. It was previously reported that *AGO1* expression is highly induced early in carrot SE and further decreased after the globular-staged embryo (Takahata, 2008). In maize, the presence of several putative *AGO1* genes, not all of them exhibiting the miR168 target sequence, further adds a complexity level to the interpretation of miRNA-mediated regulation. According to a global transcriptome analysis for the maize B73 line, *AGO1c* transcript (target of miR168) is highly expressed in differentiating tissues, such as the shoot apical meristem, immature cob, and tassel (Sekhon et al., 2011). It remains high for few days upon pollination, while in the mature and germinating maize seed *AGO1c* and miR168 are both decreased. Another *AGO1* isoform also exhibiting the miR168 target sequence (*GRMZM2G441583_T01, AGO113* or *AGO1a*) remains highly expressed during seed maturation and germination (Sekhon et al., 2011). The behavior of *AGO1c* in maize SE is consistent with the transient increase observed in carrot and in early zygotic embryogenesis. We did not analyze other *AGO1* transcripts during maize plant regeneration, but it remains possible that they were differentially expressed depending on the genotype and regulated during SE.

### Physiological relevance of stress-related miRNA regulation in maize SE

Stress-related miRNAs and their targets have been associated with sweet orange callus embryogenic potential (Wu et al., 2011, 2015) and SE in other species (Li et al., 2012). Highly proliferating tissues are thought to produce an excess of reactive oxygen species (ROS) making transcript accumulation of stress-related genes such as superoxide dismutase, cupredoxin, and multi-copper oxidases necessary to minimize cell damage and promote SE. Members of the copper superoxide dismutase (CSD, SOD) family are miRNA targets in several plant species (Jones-Rhoades and Bartel, 2004; Jovanović et al., 2014; Naya et al., 2014). These enzymes are in charge of destroying oxygen reactive species accumulating during fast plant growth (Ravet and Pilon, 2013). On the other hand, an *Arabidopsis* ortholog of the predicted maize miR408 target, *GR1*, is expressed in mitotically active tissues, such as the shoot apical meristem and floral primordium from unstressed plants in a similar to cell cycle-related gene expression profiles (Deveaux et al., 2000). AtGR1 is responsive to genotoxic stress, such as gamma radiation, and has been proposed to block mitotic cell divisions in irradiated cells to prevent premature entry into mitosis before completion of DNA repair. Therefore, the control of this transcript by miRNA makes sense in highly proliferating undifferentiated callus (Luo et al., 2006; Shen et al., 2013). Here we found that during maize hormone depletion, targets of stress-related miRNAs often displayed higher expression under darkness. In addition, their expression patterns under light showed striking differences between genotypes contrary to what observed for development-related targets. Accordingly, we propose that while stress-related miRNAs are required to control their targets in undifferentiated tissues, their participation during the plant regeneration process could be dependent on the genotype and the physiology of the EC used to initiate SE (Figure 6).

## Conclusion

The present study provides important novel information about the separate effects of hormone depletion and light presence on miRNA patterns and their target regulation during plant regeneration from maize embryogenic callus. While development or stress-related miRNAs are responsive to hormone concentration, their targets are additionally influenced by the presence of photoperiod. The reproducibility or differences observed for particular miRNA-target regulation between two different highly embryogenic genotypes provide clues for conserved miRNA roles within the SE process. Future work should aim to approach the mechanism underlying regulation of SE-related miRNAs on their targets and its relevance for the plant regeneration success.

## Author contributions

Conceived and designed the experiments: EC, NA, TD. Performed the experiments: EC, NA, VJ. Analyzed the data: EC, NA, VJ. Contributed reagents/ materials/ analysis tools: EC, NA, VJ. Wrote the paper: EC, TD. Revised the manuscript: EC, TD. Steered the whole study: TD.

### Conflict of interest statement

The authors declare that the research was conducted in the absence of any commercial or financial relationships that could be construed as a potential conflict of interest.
