# Supplementary material for: Maize miRNA and target regulation in response to hormone depletion and light exposure during somatic embryogenesis
Source: Front Plant Sci. 2015 Jul 22;6:555. doi: 10.3389/fpls.2015.00555 (PMC4510349; doi:10.3389/fpls.2015.00555)
Supplement: Supplementary file 1 [file Table1.PDF]

Table S1. DNA probes used for Northern Blot assay.

miRNA	DNA Probe
zma_miR164a-5p	5’-TGCACGTGCCCTGCTTCTCCA
zma_miR168a-5p	5’-GTCCCGATCTGCACCAAGCGA
zma_miR156a-5p	5’-GTGCTCACTCTCTTCTGTCA
zma_miR159a-3p	5’-CAGAGGTCCCTTCAATCCAAA
zma_miR397a-5p	5’-CATCAACGCTGCGCTCAATGA
zma_miR398a-3p	5’-CGGGGGCGACCTGAGAACACA
zma_miR408a	5’-GCCAGGGAAGAGGCAGTGCAG
zma_miR528a-5p	5’-CTCCTCTGCATGCCCCTTCCA
