# Supplementary material for: Maize miRNA and target regulation in response to hormone depletion and light exposure during somatic embryogenesis
Source: Front Plant Sci. 2015 Jul 22;6:555. doi: 10.3389/fpls.2015.00555 (PMC4510349; doi:10.3389/fpls.2015.00555)
Supplement: Supplementary file 2 [file Table2.PDF]

Potential targets of zma-miRNAs.

miRNA	Target	Gene name	Gene description	Expectation	UPE	Inhibition	Hybrid
zma-miR156e-5p	AC233751.1_FGT002	NA	Squamosa promoter-binding protein-like (SBP domain) transcription factor family protein [Source:UniProtKB/TrEMBL;Acc:K7USX3]	1	23.513	Cleavage	miRNA 20 CACGAGUGAGAGAAGACAGU 1<br>:::::: :::::::::::::<br>Target 1415 GUGCUCUCUCUCUUCUGUCA 1434
zma-miR156e-5p	GRMZM2G061734_T01	NA	Uncharacterized protein [Source:UniProtKB/TrEMBL;Acc:B7ZX33]	1	17.452	Cleavage	miRNA 20 CACGAGUGAGAGAAGACAGU 1<br>:::::: :::::::::::::<br>Target 1567 GUGCUCUCUCUCUUCUGUCA 1586
zma-miR156e-5p	GRMZM2G097275_T01	NA	Uncharacterized protein [Source:UniProtKB/TrEMBL;Acc:B4FQY3]	1	21.867	Cleavage	miRNA 20 CACGAGUGAGAGAAGACAGU 1<br>:::::: :::::::::::::<br>Target 1373 GUGCUCUCUCUCUUCUGUCA 1392
zma-miR156e-5p	GRMZM2G097275_T02	NA	Uncharacterized protein [Source:UniProtKB/TrEMBL;Acc:B4FQY3]	1	21.867	Cleavage	miRNA 20 CACGAGUGAGAGAAGACAGU 1<br>:::::: :::::::::::::<br>Target 1772 GUGCUCUCUCUCUUCUGUCA 1791
zma-miR156e-5p	GRMZM2G097275_T03	NA	Uncharacterized protein [Source:UniProtKB/TrEMBL;Acc:B4FQY3]	1	21.867	Cleavage	miRNA 20 CACGAGUGAGAGAAGACAGU 1<br>:::::: :::::::::::::<br>Target 1765 GUGCUCUCUCUCUUCUGUCA 1784
zma-miR156e-5p	GRMZM2G097275_T04	NA	Uncharacterized protein [Source:UniProtKB/TrEMBL;Acc:B4FQY3]	1	21.867	Cleavage	miRNA 20 CACGAGUGAGAGAAGACAGU 1<br>:::::: :::::::::::::<br>Target 1505 GUGCUCUCUCUCUUCUGUCA 1524
zma-miR156e-5p	GRMZM2G106798_T01	NA	SBP transcription factor; Squamosa promoter-binding protein-like (SBP domain) transcription factor family protein isoform 1;<br>Squamosa promoter-binding protein-like (SBP domain) transcription factor family protein isoform 2;<br>Squamosa promoter-binding protein-like (SBP domain) transcription factor family protein isoform 3;<br>Uncharacterized protein [Source:UniProtKB/TrEMBL;Acc:C0P8S5]	1	22.998	Cleavage	miRNA 20 CACGAGUGAGAGAAGACAGU 1<br>:::::: :::::::::::::<br>Target 1134 GUGCUCUCUCUCUUCUGUCA 1153
zma-miR156e-5p	GRMZM2G106798_T02	NA	SBP transcription factor; Squamosa promoter-binding protein-like (SBP domain) transcription factor family protein isoform 1;<br>Squamosa promoter-binding protein-like (SBP domain) transcription factor family protein isoform 2;<br>Squamosa promoter-binding protein-like (SBP domain) transcription factor family protein isoform 3;<br>Uncharacterized protein [Source:UniProtKB/TrEMBL;Acc:C0P8S5]	1	22.998	Cleavage	miRNA 20 CACGAGUGAGAGAAGACAGU 1<br>:::::: :::::::::::::<br>Target 907 GUGCUCUCUCUCUUCUGUCA 926
zma-miR156e-5p	GRMZM2G106798_T03	NA	SBP transcription factor; Squamosa promoter-binding protein-like (SBP domain) transcription factor family protein isoform 1;<br>Squamosa promoter-binding protein-like (SBP domain) transcription factor family protein isoform 2;<br>Squamosa promoter-binding protein-like (SBP domain) transcription factor family protein isoform 3;<br>Uncharacterized protein [Source:UniProtKB/TrEMBL;Acc:C0P8S5]	1	22.998	Cleavage	miRNA 20 CACGAGUGAGAGAAGACAGU 1<br>:::::: :::::::::::::<br>Target 888 GUGCUCUCUCUCUUCUGUCA 907
zma-miR156e-5p	GRMZM2G126018_T01	SBP23	SBP-transcription factor 23 [Source:MaizeGDB.org];<br>Uncharacterized protein [Source:UniProtKB/TrEMBL;Acc:B4FLF0]	1	18.45	Cleavage	miRNA 20 CACGAGUGAGAGAAGACAGU 1<br>:::::: :::::::::::::<br>Target 978 GUGCUCUCUCUCUUCUGUCA 997
zma-miR156e-5p	GRMZM2G126018_T02	SBP23	SBP-transcription factor 23 [Source:MaizeGDB.org];<br>Uncharacterized protein [Source:UniProtKB/TrEMBL;Acc:B4FLF0]	1	18.45	Cleavage	miRNA 20 CACGAGUGAGAGAAGACAGU 1<br>:::::: :::::::::::::<br>Target 896 GUGCUCUCUCUCUUCUGUCA 915
zma-miR156e-5p	GRMZM2G307588_T01	NA	SBP transcription factor; Uncharacterized protein [Source:UniProtKB/TrEMBL;Acc:B4FXD9]	1	16.689	Cleavage	miRNA 20 CACGAGUGAGAGAAGACAGU 1<br>:::::: :::::::::::::<br>Target 1001 GUGCUCUCUCUCUUCUGUCA 1020
zma-miR156e-5p	GRMZM2G371033_T01	NA	Squamosa promoter-binding protein-like (SBP domain) transcription factor family protein [Source:UniProtKB/TrEMBL;Acc:K7VM73]	1	18.515	Cleavage	miRNA 20 CACGAGUGAGAGAAGACAGU 1<br>:::::: :::::::::::::<br>Target 590 GUGCUCUCUCUCUUCUGUCA 609
zma-miR156e-5p	GRMZM2G460544_T01	NA	Squamosa promoter-binding protein-like (SBP domain) transcription factor family protein;<br>Uncharacterized protein [Source:UniProtKB/TrEMBL;Acc:B7ZYE2]	1	20.772	Cleavage	miRNA 20 CACGAGUGAGAGAAGACAGU 1<br>:::::: :::::::::::::<br>Target 1138 GUGCUCUCUCUCUUCUGUCA 1157

Potential targets of zma-miRNAs.

miRNA	Target	Gene name	Gene description	Expectation	UPE	Inhibition	Hybrid
zma-miR156e-5p	GRMZM5G806833_T01	NA	Uncharacterized protein [Source:UniProtKB/TrEMBL;Acc:B4FM18]	1	15.225	Cleavage	miRNA 20 CACGAGUGAGAGAAGACAGU 1<br>:::::: :::::::::::::<br>Target 591 GUGCUCUCUCUCUUCUGUCA 610
zma-miR156e-5p	GRMZM5G878561_T01	NA	NA	1	19.127	Cleavage	miRNA 20 CACGAGUGAGAGAAGACAGU 1<br>:::::: :::::::::::::<br>Target 2340 GUGCUCUCUCUCUUCUGUCA 2359
zma-miR156e-5p	GRMZM2G067624_T01	NA	SBP domain containing protein [Source:UniProtKB/TrEMBL;Acc:B6TMJ5]	2	16.263	Cleavage	miRNA 20 CACGAGUGAGAGAAGACAGU 1<br>::::: :::::::::::::<br>Target 1040 AUGCUCUCUCUCUUCUGUCA 1059
zma-miR156e-5p	GRMZM2G067624_T02	NA	SBP domain containing protein [Source:UniProtKB/TrEMBL;Acc:B6TMJ5]	2	16.263	Cleavage	miRNA 20 CACGAGUGAGAGAAGACAGU 1<br>::::: :::::::::::::<br>Target 724 AUGCUCUCUCUCUUCUGUCA 743
zma-miR156e-5p	GRMZM2G113779_T01	NA	NA	2	14.231	Cleavage	miRNA 20 CACGAGUGAGAGAAGACAGU 1<br>::::: :::::::::::::<br>Target 319 AUGCUCUCUCUCUUCUGUCA 338
zma-miR156e-5p	GRMZM2G126827_T01	NA	SBP transcription factor; Uncharacterized protein [Source:UniProtKB/TrEMBL;Acc:B4F8H7]	2	22.697	Cleavage	miRNA 20 CACGAGUGAGAGAAGACAGU 1<br>::::: :::::::::::::<br>Target 927 AUGCUCUCUCUCUUCUGUCA 946
zma-miR156e-5p	GRMZM2G156621_T01	NA	SBP transcription factor; Uncharacterized protein [Source:UniProtKB/TrEMBL;Acc:B4F8H7]	2	22.697	Cleavage	miRNA 20 CACGAGUGAGAGAAGACAGU 1<br>::::: :::::::::::::<br>Target 859 AUGCUCUCUCUCUUCUGUCA 878
zma-miR156e-5p	GRMZM2G044697_T09	NA	ethylene-dependent gravitropism-deficient and yellow-green-like 2 [Source:Projected from Arabidopsis thaliana (AT5G05740) TAIR;Acc:AT5G05740]	2.5	8.708	Cleavage	miRNA 20 CACGAGUGAGAGAAGACAGU 1<br>::::.:::::.::::::.:<br>Target 1479 AUGCUUACUCUUUUCUGUUA 1498
zma-miR156e-5p	GRMZM2G044697_T10	NA	ethylene-dependent gravitropism-deficient and yellow-green-like 2 [Source:Projected from Arabidopsis thaliana (AT5G05740) TAIR;Acc:AT5G05740]	2.5	8.708	Cleavage	miRNA 20 CACGAGUGAGAGAAGACAGU 1<br>::::.:::::.::::::.:<br>Target 1392 AUGCUUACUCUUUUCUGUUA 1411
zma-miR156e-5p	GRMZM2G133959_T04	NA	alpha/beta-Hydrolases superfamily protein [Source:Projected from Arabidopsis thaliana (AT3G11620) TAIR;Acc:AT3G11620]	2.5	23.336	Cleavage	miRNA 20 CACGAGUGAGAGAAGACAGU 1<br>:::: ::::::::::::.:<br>Target 1146 AUGCUAACUCUCUUCUGUUA 1165
zma-miR156e-5p	GRMZM2G096352_T03	NA	Acyl-CoA N-acyltransferases (NAT) superfamily protein [Source:Projected from Arabidopsis thaliana (AT2G04845) TAIR;Acc:AT2G04845]	3	15.888	Cleavage	miRNA 20 CACGAGUGAGAGAAGACAGU 1<br>::: :::::::::.::::.<br>Target 821 GUGAGCACUCUCUUUUGUCG 840
zma-miR156e-5p	GRMZM2G111204_T01	NA	Uncharacterized protein [Source:UniProtKB/TrEMBL;Acc:B7ZYP9]	3	18.913	Cleavage	miRNA 19 ACGAGU-GAGAGAAGACAGU 1<br>:::::: :::::::::::..<br>Target 527 UGCUCAGCUCUCUUCUGUUG 546
zma-miR156e-5p	GRMZM2G079823_T02	NA	Protein RIK [Source:UniProtKB/Swiss-Prot;Acc:Q32SG5]	3.5	17.008	Cleavage	miRNA 20 CACGAGUGAGAGAAGACAGU 1<br>:::::... :::::.:::.:<br>Target 1047 GUGCUUGUGCUCUUUUGUUA 1066
zma-miR156e-5p	GRMZM2G095287_T01	NA	Fructose-1	3.5	16.026	Cleavage	miRNA 20 CACGAGUGAGAGAAGACAGU 1<br>::.:::::::.::::: ::<br>Target 1466 UUGUUCACUCUUUUCUGCCA 1485
zma-miR156e-5p	GRMZM2G113579_T01	NA	NA	3.5	14.616	Cleavage	miRNA 20 CACGAGUGAGAGAAGACAGU 1<br>::::::::.:.::.::::<br>Target 1113 AUGCUCACUUUUUUUUGUCU 1132

Potential targets of zma-miRNAs.

miRNA	Target	Gene name	Gene description	Expectation	UPE	Inhibition	Hybrid
zma-miR156e-5p	GRMZM2G113579_T02	NA	NA	3.5	14.616	Cleavage	miRNA 20 CACGAGUGAGAGAAGACAGU 1<br>::::::::.:.::.::::<br>Target 446 AUGCUCACUUUUUUUUGUCU 465
zma-miR156e-5p	GRMZM2G114459_T01	NA	NA	3.5	17.304	Cleavage	miRNA 20 CACGAGUGAGAGAAGACAGU 1<br>:: .::: :.::::::::.:<br>Target 1602 GUUUUCAGUUUCUUCUGUUA 1621
zma-miR156e-5p	GRMZM2G124715_T02	NA	Uncharacterized protein [Source:UniProtKB/TrEMBL;Acc:B7ZZL6]	3.5	18.667	Translation	miRNA 20 CACGAGUGAGAGAAGACAGU 1<br>:::::: .: ::::.::::.<br>Target 1360 GUGCUCUUUGUCUUUUGUCG 1379
zma-miR156e-5p	GRMZM2G124715_T03	NA	Uncharacterized protein [Source:UniProtKB/TrEMBL;Acc:B7ZZL6]	3.5	18.667	Translation	miRNA 20 CACGAGUGAGAGAAGACAGU 1<br>:::::: .: ::::.::::.<br>Target 1248 GUGCUCUUUGUCUUUUGUCG 1267
zma-miR156e-5p	GRMZM2G409726_T01	NA	Ubiquitin2 [Source:UniProtKB/TrEMBL;Acc:K7US22]	3.5	16.071	Translation	miRNA 20 CACGAGUGAGAGAAGACAGU 1<br>:::::::.: : :::::::<br>Target 1957 GUGCUCAUUAUGUUCUGUCU 1976
zma-miR156e-5p	GRMZM2G514443_T01	NA	Uncharacterized protein [Source:UniProtKB/TrEMBL;Acc:K7UHZ0]	3.5	10.713	Cleavage	miRNA 20 CACGAGUGAGAGAAGACAGU 1<br>:::: :: :.:::::::::<br>Target 391 GUGCCCAAUUUCUUCUGUCC 410
zma-miR156e-5p	AC208440.3_FGT003	NA	NA	4	20.16	Cleavage	miRNA 20 CACGAGUGAGAGAAGACAGU 1<br>::::::.::::.:::..<br>Target 1182 CCGCUCACUUUCUUUUGUUG 1201
zma-miR156e-5p	GRMZM2G012156_T01	NA	NA	4	17.778	Cleavage	miRNA 20 CACGAGUGAGAGAAGACAGU 1<br>:: .:::.:.:::::::: :<br>Target 1560 GUCUUCAUUUUCUUCUGUGA 1579
zma-miR156e-5p	GRMZM2G022856_T01	NA	NA	4	17.548	Translation	miRNA 20 CACGAGUGAGAGAAGACAGU 1<br>::::::.::: :::: :::<br>Target 1273 AUGCUCAUUCUGUUCUUUCA 1292
zma-miR156e-5p	GRMZM2G029527_T01	NA	Uncharacterized protein [Source:UniProtKB/TrEMBL;Acc:B7ZXW1]	4	18.109	Cleavage	miRNA 20 CACGAGUGAGAGAAGACAGU 1<br>: ::::. :.:::::::::<br>Target 2360 GGGCUCGAUUUCUUCUGUCC 2379
zma-miR156e-5p	GRMZM2G029527_T02	NA	Uncharacterized protein [Source:UniProtKB/TrEMBL;Acc:B7ZXW1]	4	18.109	Cleavage	miRNA 20 CACGAGUGAGAGAAGACAGU 1<br>: ::::. :.:::::::::<br>Target 879 GGGCUCGAUUUCUUCUGUCC 898
zma-miR156e-5p	GRMZM2G033199_T01	NA	Uncharacterized protein [Source:UniProtKB/TrEMBL;Acc:B4FJU3]	4	11.877	Cleavage	miRNA 20 CACGAGUGAGAGAAGACAGU 1<br>:::: :.::::::::: ::<br>Target 33 GUGCGCGCUCUCUUCUCUCU 52
zma-miR156e-5p	GRMZM2G033199_T02	NA	Uncharacterized protein [Source:UniProtKB/TrEMBL;Acc:B4FJU3]	4	11.877	Cleavage	miRNA 20 CACGAGUGAGAGAAGACAGU 1<br>:::: :.::::::::: ::<br>Target 33 GUGCGCGCUCUCUUCUCUCU 52
zma-miR156e-5p	GRMZM2G033199_T03	NA	Uncharacterized protein [Source:UniProtKB/TrEMBL;Acc:B4FJU3]	4	9.769	Cleavage	miRNA 20 CACGAGUGAGAGAAGACAGU 1<br>:::: :.::::::::: ::<br>Target 22 GUGCGCGCUCUCUUCUCUCU 41
zma-miR156e-5p	GRMZM2G035131_T02	NA	NA	4	15.288	Cleavage	miRNA 20 CACGAGUGAGAGAAGACAGU 1<br>:::: :. :.:.::.:::::<br>Target 839 GUGCCCGGUUUUUUUUGUCA 858

Potential targets of zma-miRNAs.

miRNA	Target	Gene name	Gene description	Expectation	UPE	Inhibition	Hybrid
zma-miR156e-5p	GRMZM2G036063_T01	NA	Uncharacterized protein [Source:UniProtKB/TrEMBL;Acc:B4G0Q1]	4	16.141	Translation	miRNA 20 CACGAGUGAGAGAAGACAGU 1<br>: ::::::: :.:::::.<br>Target 640 GAGCUCACUAUUUUCUGUUC 659
zma-miR156e-5p	GRMZM2G036063_T02	NA	Uncharacterized protein [Source:UniProtKB/TrEMBL;Acc:B4G0Q1]	4	16.141	Translation	miRNA 20 CACGAGUGAGAGAAGACAGU 1<br>: ::::::: :.:::::.<br>Target 642 GAGCUCACUAUUUUCUGUUC 661
zma-miR156e-5p	GRMZM2G036063_T03	NA	Uncharacterized protein [Source:UniProtKB/TrEMBL;Acc:B4G0Q1]	4	17.5	Translation	miRNA 20 CACGAGUGAGAGAAGACAGU 1<br>: ::::::: :.:::::.<br>Target 811 GAGCUCACUAUUUUCUGUUC 830
zma-miR156e-5p	GRMZM2G040965_T01	NA	Uncharacterized protein [Source:UniProtKB/TrEMBL;Acc:C4J111]	4	12.866	Translation	miRNA 20 CACGAGUGAGAGAAGACAGU 1<br>:::..::: ::::::::<br>Target 366 AAGCUUGCUCACUUCUGUCA 385
zma-miR156e-5p	GRMZM2G040965_T02	NA	Uncharacterized protein [Source:UniProtKB/TrEMBL;Acc:C4J111]	4	12.866	Translation	miRNA 20 CACGAGUGAGAGAAGACAGU 1<br>:::..::: ::::::::<br>Target 366 AAGCUUGCUCACUUCUGUCA 385
zma-miR156e-5p	GRMZM2G040965_T03	NA	Uncharacterized protein [Source:UniProtKB/TrEMBL;Acc:C4J111]	4	12.866	Translation	miRNA 20 CACGAGUGAGAGAAGACAGU 1<br>:::..::: ::::::::<br>Target 366 AAGCUUGCUCACUUCUGUCA 385
zma-miR156e-5p	GRMZM2G041631_T01	NA	Uncharacterized protein [Source:UniProtKB/TrEMBL;Acc:C0P2K1]	4	13.733	Cleavage	miRNA 20 CACGAGUGAGAGAAGACAGU 1<br>:::::::. ::: :::::.<br>Target 682 GUGCUCAUGCUCAUCUGUUU 701
zma-miR156e-5p	GRMZM2G070239_T04	NA	Splicing factor	4	15.221	Translation	miRNA 20 CACGAGUGAGAGAAGACAGU 1<br>: :::::: ::::::::<br>Target 273 AAGGUCACUCACUUCUGUCA 292
zma-miR156e-5p	GRMZM2G070239_T06	NA	Splicing factor	4	15.221	Translation	miRNA 20 CACGAGUGAGAGAAGACAGU 1<br>: :::::: ::::::::<br>Target 270 AAGGUCACUCACUUCUGUCA 289
zma-miR156e-5p	GRMZM2G076389_T01	NA	Uncharacterized protein [Source:UniProtKB/TrEMBL;Acc:C0P4C6]	4	17.604	Cleavage	miRNA 20 CACGAGUGAGAGAAGACAGU 1<br>: ::::. :.::::::::<br>Target 2112 GGGCUCGAUUUCUUCUGUCC 2131
zma-miR156e-5p	GRMZM2G076389_T02	NA	Uncharacterized protein [Source:UniProtKB/TrEMBL;Acc:C0P4C6]	4	17.604	Cleavage	miRNA 20 CACGAGUGAGAGAAGACAGU 1<br>: ::::. :.::::::::<br>Target 879 GGGCUCGAUUUCUUCUGUCC 898
zma-miR156e-5p	GRMZM2G093217_T05	NA	P-loop containing nucleoside triphosphate hydrolases superfamily protein [Source:Projected from Arabidopsis thaliana (AT1G04730) TAIR;Acc:AT1G04730]	4	16.833	Cleavage	miRNA 20 CACGAGUGAGAGAAGACAGU 1<br>:: ::::.:.::::.:: ::<br>Target 286 GUACUCAUUUUCUUUUGCCA 305
zma-miR156e-5p	GRMZM2G096806_T01	NA	NA	4	12.338	Cleavage	miRNA 20 CACGAGUGAGAGAAGACAGU 1<br>: :::.: ::::: ::::::<br>Target 988 GAGCUUAAUCUCUCCUGUCA 1007
zma-miR156e-5p	GRMZM2G113640_T06	NA	S-adenosyl-L-methionine-dependent methyltransferases superfamily protein [Source:Projected from Arabidopsis thaliana (AT4G28830) TAIR;Acc:AT4G28830]	4	14.508	Cleavage	miRNA 20 CACGAGUGAGAGAAGACAGU 1<br>:::. .:.:::.::.:::.:<br>Target 648 GUGUCUAUUCUUUUUUGUUA 667
zma-miR156e-5p	GRMZM2G141491_T01	NA	Uncharacterized protein [Source:UniProtKB/TrEMBL;Acc:C0P2K1]	4	13.733	Cleavage	miRNA 20 CACGAGUGAGAGAAGACAGU 1<br>:::::::. ::: :::::.<br>Target 783 GUGCUCAUGCUCAUCUGUUU 802

Potential targets of zma-miRNAs.

miRNA	Target	Gene name	Gene description	Expectation	UPE	Inhibition	Hybrid
zma-miR156e-5p	GRMZM2G145300_T01	NA	Uncharacterized protein [Source:UniProtKB/TrEMBL;Acc:C4J9L7]	4	15.027	Cleavage	miRNA 20 CACGAGUGAGAGAAGACAGU 1<br>:: .:::.:.:::::::: :<br>Target 2202 GUCUUCAUUUUCUUCUGUGA 2221
zma-miR156e-5p	GRMZM2G154229_T01	NA	Uncharacterized protein [Source:UniProtKB/TrEMBL;Acc:K7VFT6]	4	20.179	Translation	miRNA 20 CACGAGUGAGAGAAGACAGU 1<br>:::::. :::: ::::::.<br>Target 3262 GUGCUUUCUCUGUUCUGUUC 3281
zma-miR156e-5p	GRMZM2G165011_T01	NA	Uncharacterized protein [Source:UniProtKB/TrEMBL;Acc:C0P7T5]	4	17.817	Cleavage	miRNA 20 CACGAGUG-AGAGAAGACAGU 1<br>:::::::: :.::::.:::..<br>Target 2195 GUGCUCACAUUUCUUUUGUUG 2215
zma-miR156e-5p	GRMZM2G167085_T01	NA	Delta-7-sterol-C5; Uncharacterized protein [Source:UniProtKB/TrEMBL;Acc:C0PJT8]	4	20.515	Translation	miRNA 20 CACGAGUGAGAGAAGACAGU 1<br>:::::.. ::: :::::::<br>Target 1058 GUGCUUGGUCUGUUCUGUCC 1077
zma-miR156e-5p	GRMZM2G167085_T02	NA	Delta-7-sterol-C5; Uncharacterized protein [Source:UniProtKB/TrEMBL;Acc:C0PJT8]	4	20.515	Translation	miRNA 20 CACGAGUGAGAGAAGACAGU 1<br>:::::.. ::: :::::::<br>Target 1198 GUGCUUGGUCUGUUCUGUCC 1217
zma-miR156e-5p	GRMZM2G167085_T03	NA	Delta-7-sterol-C5; Uncharacterized protein [Source:UniProtKB/TrEMBL;Acc:C0PJT8]	4	20.515	Translation	miRNA 20 CACGAGUGAGAGAAGACAGU 1<br>:::::.. ::: :::::::<br>Target 502 GUGCUUGGUCUGUUCUGUCC 521
zma-miR156e-5p	GRMZM2G341698_T01	NA	Fasciclin-like arabinogalactan protein 8 [Source:UniProtKB/TrEMBL;Acc:B6TEJ6]	4	11.39	Cleavage	miRNA 20 CACGAGUGAGAGAAGACAGU 1<br>:::::.:.::::.::::.<br>Target 1173 UGGCUCAUUUUCUUUUGUCG 1192
zma-miR156e-5p	GRMZM2G351502_T01	NA	NA	4	16.934	Cleavage	miRNA 20 CACGAGUGAGAGAAGACAGU 1<br>:::::.. :.::::: ::::<br>Target 283 GUGCUUGAUUUCUUCAGUCA 302
zma-miR156e-5p	GRMZM2G382747_T01	NA	NA	4	9.175	Cleavage	miRNA 20 CACGAGUGAGAGAAGACAGU 1<br>::::: ::.:.::.::::.<br>Target 1738 AUGCUCUCUUUUUUUUGUCG 1757
zma-miR156e-5p	GRMZM2G390350_T01	NA	NA	4	21.038	Cleavage	miRNA 20 CACGAGUGAGAGAAGACAGU 1<br>: :::: .::::::: ::::<br>Target 176 GAGCUCUUUCUCUUCCGUCA 195
zma-miR156e-5p	GRMZM2G421604_T02	NA	Major facilitator superfamily protein [Source:Projected from Arabidopsis thaliana (AT5G54860) TAIR;Acc:AT5G54860]	4	18.449	Cleavage	miRNA 19 ACGAGUGAGAGAAG-ACAGU 1<br>::.:..:::::::: :::::<br>Target 1678 UGUUUGCUCUCUUCAUGUCA 1697
zma-miR156e-5p	GRMZM2G421604_T03	NA	Major facilitator superfamily protein [Source:Projected from Arabidopsis thaliana (AT5G54860) TAIR;Acc:AT5G54860]	4	18.449	Cleavage	miRNA 19 ACGAGUGAGAGAAG-ACAGU 1<br>::.:..:::::::: :::::<br>Target 1970 UGUUUGCUCUCUUCAUGUCA 1989
zma-miR156e-5p	GRMZM2G473182_T01	NA	NA	4	18.082	Cleavage	miRNA 20 CACGAGUGAGAGAAGACAGU 1<br>:::: .::::::.:::::<br>Target 1572 UAGCUCUUUCUCUUUUGUCA 1591
zma-miR156e-5p	GRMZM2G473182_T02	NA	NA	4	18.082	Cleavage	miRNA 20 CACGAGUGAGAGAAGACAGU 1<br>:::: .::::::.:::::<br>Target 1537 UAGCUCUUUCUCUUUUGUCA 1556
zma-miR156e-5p	GRMZM5G800286_T01	NA	Uncharacterized protein [Source:UniProtKB/TrEMBL;Acc:K7U9X5]	4	16.446	Cleavage	miRNA 20 CACGAGUGAGAGAAGACAGU 1<br>:::::..::::::: :::.<br>Target 452 GUGCUUGCUCUCUUAUGUUU 471

Potential targets of zma-miRNAs.

miRNA	Target	Gene name	Gene description	Expectation	UPE	Inhibition	Hybrid
zma-miR156e-5p	AC198940.4_FGT008	NA	NA	4.5	14.151	Cleavage	miRNA 20 CACGAGUGAGAGAAGACAGU 1<br>::.:..: .::::::: ::<br>Target 701 GUGUUUGCCUUCUUCUGACA 720
zma-miR156e-5p	GRMZM2G031501_T02	NA	Uncharacterized protein; p8MTCP1 [Source:UniProtKB/TrEMBL;Acc:B4FPU2]	4.5	12.597	Cleavage	miRNA 20 CACGAGUGAGAGAAGACAGU 1<br>:::::: :::::::: ::.<br>Target 129 GUGCUCUCUCUCUUCAUUCG 148
zma-miR156e-5p	GRMZM2G031501_T03	NA	Uncharacterized protein; p8MTCP1 [Source:UniProtKB/TrEMBL;Acc:B4FPU2]	4.5	14.684	Cleavage	miRNA 20 CACGAGUGAGAGAAGACAGU 1<br>:::::: :::::::: ::.<br>Target 129 GUGCUCUCUCUCUUCAUUCG 148
zma-miR156e-5p	GRMZM2G031501_T04	NA	Uncharacterized protein; p8MTCP1 [Source:UniProtKB/TrEMBL;Acc:B4FPU2]	4.5	13.01	Cleavage	miRNA 20 CACGAGUGAGAGAAGACAGU 1<br>:::::: :::::::: ::.<br>Target 129 GUGCUCUCUCUCUUCAUUCG 148
zma-miR156e-5p	GRMZM2G031501_T05	NA	Uncharacterized protein; p8MTCP1 [Source:UniProtKB/TrEMBL;Acc:B4FPU2]	4.5	11.692	Cleavage	miRNA 20 CACGAGUGAGAGAAGACAGU 1<br>:::::: :::::::: ::.<br>Target 73 GUGCUCUCUCUCUUCAUUCG 92
zma-miR156e-5p	GRMZM2G064962_T01	NA	Uncharacterized protein [Source:UniProtKB/TrEMBL;Acc:B4FJI9]	4.5	19.268	Cleavage	miRNA 20 CACGAGUGAGAGAAGACAGU 1<br>:::: ::: :::::::::<br>Target 1461 GUGCACACACUCUUCUGUAU 1480
zma-miR156e-5p	GRMZM2G064962_T02	NA	Uncharacterized protein [Source:UniProtKB/TrEMBL;Acc:B4FJI9]	4.5	19.268	Cleavage	miRNA 20 CACGAGUGAGAGAAGACAGU 1<br>:::: ::: :::::::::<br>Target 1362 GUGCACACACUCUUCUGUAU 1381
zma-miR156e-5p	GRMZM2G064962_T03	NA	Uncharacterized protein [Source:UniProtKB/TrEMBL;Acc:B4FJI9]	4.5	19.268	Cleavage	miRNA 20 CACGAGUGAGAGAAGACAGU 1<br>:::: ::: :::::::::<br>Target 1347 GUGCACACACUCUUCUGUAU 1366
zma-miR156e-5p	GRMZM2G068217_T01	NA	Ethylene insensitive 2 [Source:UniProtKB/TrEMBL;Acc:Q6JN48]	4.5	17.514	Cleavage	miRNA 20 CACGAGUGAGAGAAGACAGU 1<br>:: ::::::::.::.:: :<br>Target 4700 GUUCUCACUCUUUUUUGCCC 4719
zma-miR156e-5p	GRMZM2G071264_T02	NA	Uncharacterized protein [Source:UniProtKB/TrEMBL;Acc:K7TLU4]	4.5	17.427	Cleavage	miRNA 20 CACGAGUGAGAGAAGACAGU 1<br>:.:::.:::::::::: :<br>Target 1210 AGGUUCAUUCUCUUCUGUGA 1229
zma-miR156e-5p	GRMZM2G097353_T01	NA	Uncharacterized protein [Source:UniProtKB/TrEMBL;Acc:K7UWS1]	4.5	17.176	Cleavage	miRNA 20 CACGAGUGAGAGAAGACAGU 1<br>:.:::.:::::::::: :<br>Target 474 AGGUUCAUUCUCUUCUGUGA 493
zma-miR156e-5p	GRMZM2G116314_T01	NA	Putative ubiquitin carboxyl-terminal hydrolase superfamily protein [Source:UniProtKB/TrEMBL;Acc:K7UA60]	4.5	10.683	Cleavage	miRNA 20 CACGAGUGAGAGAAGACAGU 1<br>:::::.. ::.::.::::.<br>Target 1947 CUGCUCGUACUUUUUUGUCG 1966
zma-miR156e-5p	GRMZM2G118979_T01	NA	Uncharacterized protein [Source:UniProtKB/TrEMBL;Acc:B4G230]	4.5	13.311	Cleavage	miRNA 20 CACGAGUGAGAGAAGACAGU 1<br>: ::::.:::.:::: :::<br>Target 725 AUCCUCAUUCUUUUCUUUCA 744
zma-miR156e-5p	GRMZM2G118979_T02	NA	Uncharacterized protein [Source:UniProtKB/TrEMBL;Acc:B4G230]	4.5	13.311	Cleavage	miRNA 20 CACGAGUGAGAGAAGACAGU 1<br>: ::::.:::.:::: :::<br>Target 679 AUCCUCAUUCUUUUCUUUCA 698
zma-miR156e-5p	GRMZM2G124371_T01	NA	Uncharacterized protein [Source:UniProtKB/TrEMBL;Acc:C0HDV0]	4.5	14.245	Cleavage	miRNA 20 CACGAGUGAGAGAAGACAGU 1<br>::.:.:. .:.::::::::<br>Target 3372 UUGUUUAUCUUUUUCUGUCA 3391

miRNA	Target	Gene name	Gene description	Expectation	UPE	Inhibition	Hybrid
zma-miR156e-5p	GRMZM2G153594_T01	NA	NA	4.5	24.334	Translation	miRNA 20 CACGAGUGAGAGAAGACAGU 1<br>::::::: .::::::::<br>Target 2421 CCUCUCACUCCUUUCUGUCA 2440
zma-miR156e-5p	GRMZM2G155232_T04	NA	Uncharacterized protein [Source:UniProtKB/TrEMBL;Acc:B4FHA4]	4.5	17.375	Translation	miRNA 20 CACGAGUGAGAGAAGACAGU 1<br>::::..::: .::::::..<br>Target 871 CUGCUUGCUCAUUUCUGUUG 890
zma-miR156e-5p	GRMZM2G158197_T01	NA	Uncharacterized protein [Source:UniProtKB/TrEMBL;Acc:B4F8S1]	4.5	17.681	Cleavage	miRNA 20 CACGAGUGAGAGAAGACAGU 1<br>: ::::.:::.:::: :::<br>Target 837 CUCCUCAUUCUUUUCUUUCA 856
zma-miR156e-5p	GRMZM2G158734_T01	NA	DNA-binding protein phosphatase 1 [Source:Projected from Arabidopsis thaliana (AT2G25620) TAIR;Acc:AT2G25620]	4.5	19.145	Cleavage	miRNA 20 CACGAGUGAGAGAAGACAGU 1<br>::::: ::.:::::: .:<br>Target 589 CUGCUCUCUUUCUUCUGGUA 608
zma-miR156e-5p	GRMZM2G158734_T02	NA	DNA-binding protein phosphatase 1 [Source:Projected from Arabidopsis thaliana (AT2G25620) TAIR;Acc:AT2G25620]	4.5	19.145	Cleavage	miRNA 20 CACGAGUGAGAGAAGACAGU 1<br>::::: ::.:::::: .:<br>Target 340 CUGCUCUCUUUCUUCUGGUA 359
zma-miR156e-5p	GRMZM2G158734_T04	NA	DNA-binding protein phosphatase 1 [Source:Projected from Arabidopsis thaliana (AT2G25620) TAIR;Acc:AT2G25620]	4.5	16.503	Cleavage	miRNA 20 CACGAGUGAGAGAAGACAGU 1<br>::::: ::.:::::: .:<br>Target 594 CUGCUCUCUUUCUUCUGGUA 613
zma-miR156e-5p	GRMZM2G171317_T01	NA	DNA-3-methyladenine glycosylase I [Source:UniProtKB/TrEMBL;Acc:K7VDZ5]	4.5	19.99	Cleavage	miRNA 20 CACGAGUGAGAGAAGACAGU 1<br>::: ::.::::::.: :::<br>Target 1382 UUGCCCAUUCUCUUUUAUCA 1401
zma-miR156e-5p	GRMZM2G172657_T01	NA	Uncharacterized protein [Source:UniProtKB/TrEMBL;Acc:C0PGA9]	4.5	10.801	Cleavage	miRNA 20 CACGAGUGAGAGAAGACAGU 1<br>::::. .:.:.::.:::::<br>Target 2344 UUGCUUCUUUUUUUUUGUCA 2363
zma-miR156e-5p	GRMZM2G344967_T01	NA	Uncharacterized protein [Source:UniProtKB/TrEMBL;Acc:K7U4E0]	4.5	22.193	Cleavage	miRNA 20 CACGAGUGAGAGAAGACAGU 1<br>:::: .:.::::::::: :<br>Target 117 GUGCAUAUUCUCUUCUGCCU 136
zma-miR156e-5p	GRMZM2G402653_T01	NA	Putative oxysterol binding domain family protein [Source:UniProtKB/TrEMBL;Acc:K7TTI8]	4.5	24.442	Cleavage	miRNA 20 CACGAGUGAGAGAAGACAGU 1<br>:::.:.:.:::::: ::::<br>Target 542 GUGUUUAUUCUCUUGGGUCA 561
zma-miR156e-5p	GRMZM2G402653_T02	NA	Putative oxysterol binding domain family protein [Source:UniProtKB/TrEMBL;Acc:K7TTI8]	4.5	24.442	Cleavage	miRNA 20 CACGAGUGAGAGAAGACAGU 1<br>:::.:.:.:::::: ::::<br>Target 542 GUGUUUAUUCUCUUGGGUCA 561
zma-miR156e-5p	GRMZM2G402653_T03	NA	Putative oxysterol binding domain family protein [Source:UniProtKB/TrEMBL;Acc:K7TTI8]	4.5	24.442	Cleavage	miRNA 20 CACGAGUGAGAGAAGACAGU 1<br>:::.:.:.:::::: ::::<br>Target 722 GUGUUUAUUCUCUUGGGUCA 741
zma-miR156e-5p	GRMZM2G423169_T02	NA	Ribosomal protein L25/Gln-tRNA synthetase	4.5	15.277	Translation	miRNA 20 CACGAGUGAGAGAAGACAGU 1<br>::.:::.:: :::.::::<br>Target 960 AUGUUCAUUCACUUUUGUCU 979
zma-miR156e-5p	GRMZM2G437119_T01	NA	Uncharacterized protein [Source:UniProtKB/TrEMBL;Acc:B4FPC0]	4.5	19.814	Translation	miRNA 20 CACGAGUGAGAGAAGACAGU 1<br>:::::::: :.::::: .:<br>Target 1409 CUGCUCACUGUUUUCUGAUA 1428
zma-miR156e-5p	GRMZM2G451366_T01	NA	Nicalin; Uncharacterized protein [Source:UniProtKB/TrEMBL;Acc:B4FZF8]	4.5	14.788	Cleavage	miRNA 20 CACGAGUGAGAGAAGACAGU 1<br>:: ::..::::::::::.<br>Target 1950 CUGAUCGUUCUCUUCUGUUU 1969

miRNA	Target	Gene name	Gene description	Expectation	UPE	Inhibition	Hybrid
zma-miR156e-5p	GRMZM2G702426_T01	RPL17	60S ribosomal protein L17 [Source:UniProtKB/Swiss-Prot;Acc:O48557]	4.5	10.339	Cleavage	miRNA 20 CACGAGUGAGAGAAGACAGU 1<br>:::: ::.:.:.::.:::.<br>Target 909 GUGCCCAUUUUUUUUUGUUU 928
zma-miR156e-5p	GRMZM5G832281_T01	NA	Uncharacterized protein [Source:UniProtKB/TrEMBL;Acc:B4G267]	4.5	17.581	Cleavage	miRNA 20 CACGAGUGAGAGAAGACAGU 1<br>::::..::.::::.:::.<br>Target 935 CUGCUUGCUUUCUUUUGUUC 954
zma-miR156e-5p	AC193658.3_FGT001	NA	Uncharacterized protein [Source:UniProtKB/TrEMBL;Acc:K7VSX0]	5	14.645	Cleavage	miRNA 20 CACGAGUGAGAGAAGACAGU 1<br>:::::.:::::: ::::.<br>Target 56 CUGCUCGCUCUCUGGUGUCG 75
zma-miR156e-5p	GRMZM2G003108_T01	NA	Uncharacterized protein [Source:UniProtKB/TrEMBL;Acc:C0HEG4]	5	19.069	Cleavage	miRNA 20 CACGAGUGAGAGAAGACAGU 1<br>:.:.::::::::: ::.:<br>Target 1123 AAGUUUACUCUCUUCGGUUA 1142
zma-miR156e-5p	GRMZM2G047255_T01	NA	Uncharacterized protein [Source:UniProtKB/TrEMBL;Acc:B4FW52]	5	11.758	Cleavage	miRNA 20 CACGAGUGAGAGAAGACAGU 1<br>:::.:.:.::::::. ::.<br>Target 2355 GUGUUUAUUCUCUUUCGUUC 2374
zma-miR156e-5p	GRMZM2G047255_T02	NA	Uncharacterized protein [Source:UniProtKB/TrEMBL;Acc:B4FW52]	5	11.758	Cleavage	miRNA 20 CACGAGUGAGAGAAGACAGU 1<br>:::.:.:.::::::. ::.<br>Target 992 GUGUUUAUUCUCUUUCGUUC 1011
zma-miR156e-5p	GRMZM2G054921_T01	NA	NA	5	4.267	Cleavage	miRNA 20 CACGAGUGAGAGAAGACAGU 1<br>:::::: ::::::::: :<br>Target 166 GUGCUCUCUCUCUUCUCUGU 185
zma-miR156e-5p	GRMZM2G071270_T01	NA	NA	5	18.28	Cleavage	miRNA 20 CACGAGUGAGAGAAGACAGU 1<br>::.:.:::.::::::.:<br>Target 1202 ACACUUAUUCUUUUCUGUUA 1221
zma-miR156e-5p	GRMZM2G071270_T01	NA	NA	5	19.538	Cleavage	miRNA 20 CACGAGUGAGAGAAGACAGU 1<br>::.:.:::.::::::.:<br>Target 3989 ACACUUAUUCUUUUCUGUUA 4008
zma-miR156e-5p	GRMZM2G071270_T02	NA	NA	5	18.28	Cleavage	miRNA 20 CACGAGUGAGAGAAGACAGU 1<br>::.:.:::.::::::.:<br>Target 1202 ACACUUAUUCUUUUCUGUUA 1221
zma-miR156e-5p	GRMZM2G087243_T04	NA	Uncharacterized protein [Source:UniProtKB/TrEMBL;Acc:B4FR38]	5	16.643	Cleavage	miRNA 20 CACGAGUGAGAGAAGACAGU 1<br>:::::..::::.::.: :.<br>Target 184 GUGCUUGCUCUUUUUUUUUU 203
zma-miR156e-5p	GRMZM2G093157_T02	NA	Cyclin superfamily protein	5	16.946	Cleavage	miRNA 20 CACGAGUGAGAGAAGACAGU 1<br>:: .:::::.:.::::::<br>Target 1443 GUUUUCACUUUUUUCUGUAU 1462
zma-miR156e-5p	GRMZM2G093157_T03	NA	Cyclin superfamily protein	5	16.946	Cleavage	miRNA 20 CACGAGUGAGAGAAGACAGU 1<br>:: .:::::.:.::::::<br>Target 479 GUUUUCACUUUUUUCUGUAU 498
zma-miR156e-5p	GRMZM2G095039_T01	NA	Rapid alkalinization factor 1; Uncharacterized protein [Source:UniProtKB/TrEMBL;Acc:B4FV46]	5	12.632	Cleavage	miRNA 20 CACGAGUGAGAGAAGACAGU 1<br>::::...:.::: :::::.<br>Target 825 UUGCUUGUUUUCUCCUGUCG 844
zma-miR156e-5p	GRMZM2G106039_T01	NA	Ribosomal protein S2 [Source:UniProtKB/TrEMBL;Acc:Q6R996]	5	12.141	Cleavage	miRNA 20 CACGAGUGAGAGAAGACAGU 1<br>::.:.:::::.::. ::.:<br>Target 52 AUGUUUACUCUUUUUGGUUA 71

miRNA	Target	Gene name	Gene description	Expectation	UPE	Inhibition	Hybrid
zma-miR156e-5p	GRMZM2G114584_T02	NA	Uncharacterized protein [Source:UniProtKB/TrEMBL;Acc:B4FJL8]	5	18.88	Cleavage	miRNA 20 CACGAGUGAGAGAAGACAGU 1<br>:.:..::::.::::::..<br>Target 464 UGGUUUGCUCUUUUCUGUUG 483
zma-miR156e-5p	GRMZM2G126266_T01	NA	NA	5	15.819	Cleavage	miRNA 20 CACGAGUGAGAGAAGACAGU 1<br>::.:...:::: :::::.<br>Target 1477 UUGUUUGUUCUCUGCUGUCG 1496
zma-miR156e-5p	GRMZM2G126266_T02	NA	NA	5	15.819	Cleavage	miRNA 20 CACGAGUGAGAGAAGACAGU 1<br>::.:...:::: :::::.<br>Target 2063 UUGUUUGUUCUCUGCUGUCG 2082
zma-miR156e-5p	GRMZM2G137707_T02	NA	Uncharacterized protein [Source:UniProtKB/TrEMBL;Acc:B4FBR8]	5	17.998	Cleavage	miRNA 20 CACGAGUGAGAGAAGACAGU 1<br>:::.:...:.:.: ::::.:<br>Target 883 GUGUUUGUUUUUUGCUGUUA 902
zma-miR156e-5p	GRMZM2G140612_T01	NA	Uncharacterized protein [Source:UniProtKB/TrEMBL;Acc:B8A0V7]	5	3.151	Cleavage	miRNA 20 CACGAGUGAGAGAAGACAGU 1<br>:::.::.:::::::::<br>Target 3 ACUCUCGCUUUCUUCUGUCU 22
zma-miR156e-5p	GRMZM2G140612_T02	NA	Uncharacterized protein [Source:UniProtKB/TrEMBL;Acc:B8A0V7]	5	3.151	Cleavage	miRNA 20 CACGAGUGAGAGAAGACAGU 1<br>:::.::.:::::::::<br>Target 3 ACUCUCGCUUUCUUCUGUCU 22
zma-miR156e-5p	GRMZM2G140612_T03	NA	Uncharacterized protein [Source:UniProtKB/TrEMBL;Acc:B8A0V7]	5	3.017	Cleavage	miRNA 20 CACGAGUGAGAGAAGACAGU 1<br>:::.::.:::::::::<br>Target 1 ACUCUCGCUUUCUUCUGUCU 20
zma-miR156e-5p	GRMZM2G140612_T04	NA	Uncharacterized protein [Source:UniProtKB/TrEMBL;Acc:B8A0V7]	5	3.151	Cleavage	miRNA 20 CACGAGUGAGAGAAGACAGU 1<br>:::.::.:::::::::<br>Target 3 ACUCUCGCUUUCUUCUGUCU 22
zma-miR156e-5p	GRMZM2G143211_T01	NA	Uncharacterized protein [Source:UniProtKB/TrEMBL;Acc:C4J4C4]	5	12.475	Cleavage	miRNA 20 CACGAGUGAGAGAAGACAGU 1<br>::::.:::::.::.: :..<br>Target 2238 AUGCUUACUCUUUUUUUUUG 2257
zma-miR156e-5p	GRMZM2G143211_T02	NA	Uncharacterized protein [Source:UniProtKB/TrEMBL;Acc:C4J4C4]	5	12.475	Cleavage	miRNA 20 CACGAGUGAGAGAAGACAGU 1<br>::::.:::::.::.: :..<br>Target 658 AUGCUUACUCUUUUUUUUUG 677
zma-miR156e-5p	GRMZM2G143854_T04	NA	Uncharacterized protein [Source:UniProtKB/TrEMBL;Acc:B6SWF0]	5	21.841	Cleavage	miRNA 20 CACGAGUGAGAGAAGACAGU 1<br>:::::..::::.: ::: :.<br>Target 606 GUGCUUGCUCUUUGCUGCCG 625
zma-miR156e-5p	GRMZM2G146446_T01	NA	transmembrane protein-related [Source:Projected from Arabidopsis thaliana (AT2G46060) TAIR;Acc:AT2G46060]	5	20.15	Cleavage	miRNA 20 CACGAGUGAGAGAAGACAGU 1<br>: ::..::::::::::::<br>Target 36 GCCGUCGUUCUCUUCUGUCA 55
zma-miR156e-5p	GRMZM2G146446_T02	NA	transmembrane protein-related [Source:Projected from Arabidopsis thaliana (AT2G46060) TAIR;Acc:AT2G46060]	5	20.15	Cleavage	miRNA 20 CACGAGUGAGAGAAGACAGU 1<br>: ::..::::::::::::<br>Target 36 GCCGUCGUUCUCUUCUGUCA 55
zma-miR156e-5p	GRMZM2G147319_T02	NA	Retrotransposon protein; Uncharacterized protein [Source:UniProtKB/TrEMBL;Acc:B4FBK7]	5	9.869	Cleavage	miRNA 20 CACGAGUGAGAGAAGACAGU 1<br>:::.:.:.:::::: :::<br>Target 1958 AGGCUUAUUUUCUUCUAUCA 1977
zma-miR156e-5p	GRMZM2G148370_T01	NA	CRAL/TRIO domain containing protein; Uncharacterized protein [Source:UniProtKB/TrEMBL;Acc:B6U4K1]	5	20.082	Cleavage	miRNA 20 CACGAGUGAGAGAAGACAGU 1<br>:.:.::::::::: ::.:<br>Target 997 AAGUUUACUCUCUUCGGUUA 1016

miRNA	Target	Gene name	Gene description	Expectation	UPE	Inhibition	Hybrid
zma-miR156e-5p	GRMZM2G148370_T02	NA	CRAL/TRIO domain containing protein; Uncharacterized protein [Source:UniProtKB/TrEMBL;Acc:B6U4K1]	5	20.082	Cleavage	miRNA 20 CACGAGUGAGAGAAGACAGU 1<br>:.:.::::::::: ::.:<br>Target 1084 AAGUUUACUCUCUUCGGUUA 1103
zma-miR156e-5p	GRMZM2G148370_T03	NA	CRAL/TRIO domain containing protein; Uncharacterized protein [Source:UniProtKB/TrEMBL;Acc:B6U4K1]	5	20.082	Cleavage	miRNA 20 CACGAGUGAGAGAAGACAGU 1<br>:.:.::::::::: ::.:<br>Target 1030 AAGUUUACUCUCUUCGGUUA 1049
zma-miR156e-5p	GRMZM2G148370_T04	NA	CRAL/TRIO domain containing protein; Uncharacterized protein [Source:UniProtKB/TrEMBL;Acc:B6U4K1]	5	20.082	Cleavage	miRNA 20 CACGAGUGAGAGAAGACAGU 1<br>:.:.::::::::: ::.:<br>Target 1027 AAGUUUACUCUCUUCGGUUA 1046
zma-miR156e-5p	GRMZM2G163888_T01	NA	Putative DUF1296 domain containing family protein; Uncharacterized protein [Source:UniProtKB/TrEMBL;Acc:C0P4D9]	5	19.654	Cleavage	miRNA 20 CACGAGUGAGAGAAGACAGU 1<br>.:.:.::::::.:::::<br>Target 2965 UAUUUUAUUCUCUUUUGUCA 2984
zma-miR156e-5p	GRMZM2G176735_T02	NA	Putative uncharacterized protein [Source:UniProtKB/TrEMBL;Acc:B6SPV8]	5	18.707	Cleavage	miRNA 20 CACGAGUGAGAGAAGACAGU 1<br>::::::.:::::: .: ::<br>Target 3236 GUGCUCGCUCUCUGUUUUCU 3255
zma-miR156e-5p	GRMZM2G344001_T01	NA	NA	5	4.267	Cleavage	miRNA 20 CACGAGUGAGAGAAGACAGU 1<br>:::::: ::::::::: :<br>Target 166 GUGCUCUCUCUCUUCUCUGU 185
zma-miR156e-5p	GRMZM5G804477_T01	NA	NA	5	9.183	Cleavage	miRNA 20 CACGAGUGAGAGAAGACAGU 1<br>:::: ::::::::::<br>Target 735 UACAUCACGCUCUUCUGUCA 754
zma-miR156e-5p	GRMZM5G804661_T01	NA	Uncharacterized protein [Source:UniProtKB/TrEMBL;Acc:K7U710]	5	13.899	Cleavage	miRNA 20 CACGAGUGAGAGAAGACAGU 1<br>: :::.::::::.:::::<br>Target 102 GGUAUCAUUCUCUUUUGUCA 121
zma-miR156e-5p	GRMZM5G875735_T01	NA	NA	5	16.561	Cleavage	miRNA 20 CACGAGUGAGAGAAGACAGU 1<br>: :: ::::::::::::<br>Target 375 UUUGUCCCUCUCUUCUGUCA 394
zma-miR156e-5p	GRMZM5G885938_T02	NA	Putative uncharacterized protein [Source:UniProtKB/TrEMBL;Acc:B6T6M2]	5	9.183	Cleavage	miRNA 20 CACGAGUGAGAGAAGACAGU 1<br>:::: ::::::::::<br>Target 733 UACAUCACGCUCUUCUGUCA 752
zma-miR156e-5p	GRMZM5G899582_T02	NA	Putative uncharacterized protein [Source:UniProtKB/TrEMBL;Acc:B6T6M2]	5	9.093	Cleavage	miRNA 20 CACGAGUGAGAGAAGACAGU 1<br>:::: ::::::::::<br>Target 733 UACAUCACGCUCUUCUGUCA 752
zma-miR159a-3p	GRMZM2G534485_T01	NA	Uncharacterized protein [Source:UniProtKB/TrEMBL;Acc:K7UPX2]	0	19.069	Cleavage	miRNA 21 GUCUCGAGGGAAGUUAGGUUU 1<br>:::::::::::::::::::::<br>Target 158 CAGAGCUCCCUUCAAUCCAAA 178
zma-miR159a-3p	GRMZM2G167088_T01	NA	Putative MYB DNA-binding domain superfamily protein [Source:UniProtKB/TrEMBL;Acc:K7VW09]	1	20.367	Cleavage	miRNA 20 UCUCGAGGGAAGUUAGGUUU 1<br>::::::::::::::::::::<br>Target 1100 CGAGCUCCCUUCAAUCCAAA 1119
zma-miR159a-3p	GRMZM2G416652_T01	NA	Putative MYB DNA-binding domain superfamily protein [Source:UniProtKB/TrEMBL;Acc:K7VIH9]	1	20.367	Cleavage	miRNA 20 UCUCGAGGGAAGUUAGGUUU 1<br>::::::::::::::::::::<br>Target 890 CGAGCUCCCUUCAAUCCAAA 909
zma-miR159a-3p	GRMZM2G416652_T02	NA	Putative MYB DNA-binding domain superfamily protein [Source:UniProtKB/TrEMBL;Acc:K7VIH9]	1	20.367	Cleavage	miRNA 20 UCUCGAGGGAAGUUAGGUUU 1<br>::::::::::::::::::::<br>Target 890 CGAGCUCCCUUCAAUCCAAA 909

Potential targets of zma-miRNAs.

miRNA	Target	Gene name	Gene description	Expectation	UPE	Inhibition	Hybrid
zma-miR159a-3p	GRMZM2G004090_T01	NA	Putative MYB DNA-binding domain superfamily protein [Source:UniProtKB/TrEMBL;Acc:K7WAF3]	1.5	20.447	Cleavage	miRNA 20 UCUCGAGGGAAGUUAGGUUU 1<br>::::::::::::.::::::<br>Target 926 CGAGCUCCCUUCAGUCCAAA 945
zma-miR159a-3p	GRMZM2G127720_T01	NA	Uncharacterized protein [Source:UniProtKB/TrEMBL;Acc:K7UZM7]	1.5	20.911	Cleavage	miRNA 20 UCUCGAGGGAAGUUAGGUUU 1<br>::::::::::::.::::::<br>Target 33 AGAGCUCCCUUCGAUCCAAU 52
zma-miR159a-3p	GRMZM2G423833_T01	NA	Putative MYB DNA-binding domain superfamily protein; Uncharacterized protein [Source:UniProtKB/TrEMBL;Acc:C0PHI8]	1.5	16.255	Cleavage	miRNA 21 GUCUCGAGGGAAGUUAGGUUU 1<br>::::: :::::::.:::::::<br>Target 905 CAGAGAUCCCUUCGAUCCAAA 925
zma-miR159a-3p	AC209015.3_FGT004	NA	Uncharacterized protein [Source:UniProtKB/TrEMBL;Acc:K7UND4]	2	13.983	Cleavage	miRNA 21 GUCUCGAGGGAAGUUAGGUUU 1<br>:.::: :::::::.:::::::<br>Target 328 CGGAGAUCCCUUCGAUCCAAA 348
zma-miR159a-3p	GRMZM2G028054_T01	NA	Putative MYB DNA-binding domain superfamily protein isoform 1; Putative MYB DNA-binding domain superfamily protein isoform 2; Putative MYB DNA-binding domain superfamily protein isoform 3; Uncharacterized protein [Source:UniProtKB/TrEMBL;Acc:B4FN81]	2	16.122	Cleavage	miRNA 21 GUCUCGAGGGAAGUUAGGUUU 1<br>..:::: :::::::.::::::<br>Target 1509 UGGAGCCCCCUUCAGUCCAAA 1529
zma-miR159a-3p	GRMZM2G028054_T02	NA	Putative MYB DNA-binding domain superfamily protein isoform 1; Putative MYB DNA-binding domain superfamily protein isoform 2; Putative MYB DNA-binding domain superfamily protein isoform 3; Uncharacterized protein [Source:UniProtKB/TrEMBL;Acc:B4FN81]	2	16.122	Cleavage	miRNA 21 GUCUCGAGGGAAGUUAGGUUU 1<br>..:::: :::::::.::::::<br>Target 1419 UGGAGCCCCCUUCAGUCCAAA 1439
zma-miR159a-3p	GRMZM2G028054_T03	NA	Putative MYB DNA-binding domain superfamily protein isoform 1; Putative MYB DNA-binding domain superfamily protein isoform 2; Putative MYB DNA-binding domain superfamily protein isoform 3; Uncharacterized protein [Source:UniProtKB/TrEMBL;Acc:B4FN81]	2	16.122	Cleavage	miRNA 21 GUCUCGAGGGAAGUUAGGUUU 1<br>..:::: :::::::.::::::<br>Target 1360 UGGAGCCCCCUUCAGUCCAAA 1380
zma-miR159a-3p	GRMZM2G046443_T01	NA	NA	2	24.756	Cleavage	miRNA 21 GUCUCGAGGGAAGUUAGGUUU 1<br>..:::::: :::.::::::::<br>Target 349 UGGAGCUCCAUUCGAUCCAAA 369
zma-miR159a-3p	GRMZM2G075064_T01	NA	Putative MYB DNA-binding domain superfamily protein [Source:UniProtKB/TrEMBL;Acc:K7UZL6]	2	18.026	Cleavage	miRNA 21 GUCUCGAGGGAAGUUAGGUUU 1<br>:.::: :::::::.:::::::<br>Target 851 CGGAGAUCCCUUCGAUCCAAA 871
zma-miR159a-3p	GRMZM2G093789_T01	NA	Putative MYB DNA-binding domain superfamily protein; Uncharacterized protein [Source:UniProtKB/TrEMBL;Acc:B4FXU8]	2	17.64	Cleavage	miRNA 21 GUCUCGAGGGAAGUUAGGUUU 1<br>::::: :.:::::.:::::::<br>Target 1120 CAGAGAUUCCUUCGAUCCAAA 1140
zma-miR159a-3p	GRMZM2G376684_T01	NA	Putative MYB DNA-binding domain superfamily protein [Source:UniProtKB/TrEMBL;Acc:K7UIP9]	2	13.939	Cleavage	miRNA 21 GUCUCGAGGGAAGUUAGGUUU 1<br>..::: :::::::.:::::::<br>Target 764 UGGAGAUCCCUUCGAUCCAAA 784
zma-miR159a-3p	AC204352.3_FGT012	NA	Uncharacterized protein [Source:UniProtKB/TrEMBL;Acc:K7UUI7]	2.5	20.466	Cleavage	miRNA 21 GUCUCGAGGGAAGUUAGGUUU 1<br>:::::: :::::::: :::::<br>Target 650 CAGAGCACCCUUCAAGCCAAA 670
zma-miR159a-3p	GRMZM2G139688_T01	MYB138	MYBGA transcription factor; Transcription factor GAMYB; Uncharacterized protein [Source:UniProtKB/TrEMBL;Acc:B4F9Y7/MaizeGDB.org]	2.5	17.184	Cleavage	miRNA 21 GUCUCGAGGGAAGUUAGGUUU 1<br>..:::::::::::: :::::.<br>Target 1399 UGGAGCUCCCUUCACUCCAAG 1419
zma-miR159a-3p	GRMZM2G038195_T01	NA	Serine/threonine-protein phosphatase [Source:UniProtKB/TrEMBL;Acc:B4FSV7]	3	17.233	Cleavage	miRNA 21 GUCUCGAGGGAAGUUAGGUUU 1<br>:::: ::.::::::::.:::<br>Target 1114 CAGAACUUCCUUCAAUUCAAC 1134
zma-miR159a-3p	GRMZM2G038195_T02	NA	Serine/threonine-protein phosphatase [Source:UniProtKB/TrEMBL;Acc:B4FSV7]	3	17.233	Cleavage	miRNA 21 GUCUCGAGGGAAGUUAGGUUU 1<br>:::: ::.::::::::.:::<br>Target 1102 CAGAACUUCCUUCAAUUCAAC 1122

Potential targets of zma-miRNAs.

miRNA	Target	Gene name	Gene description	Expectation	UPE	Inhibition	Hybrid
zma-miR159a-3p	GRMZM2G070523_T01	NA	Uncharacterized protein [Source:UniProtKB/TrEMBL;Acc:B4FZJ9]	3	16.911	Cleavage	miRNA 21 GUCUCGAGGGAAGUUAGGUUU 1<br>..::::::::::::: ::::<br>Target 1095 UGGAGCUCCCUUCAAACCAAU 1115
zma-miR159a-3p	GRMZM2G070523_T02	NA	Uncharacterized protein [Source:UniProtKB/TrEMBL;Acc:B4FZJ9]	3	16.911	Cleavage	miRNA 21 GUCUCGAGGGAAGUUAGGUUU 1<br>..::::::::::::: ::::<br>Target 1044 UGGAGCUCCCUUCAAACCAAU 1064
zma-miR159a-3p	GRMZM2G070523_T03	NA	Uncharacterized protein [Source:UniProtKB/TrEMBL;Acc:B4FZJ9]	3	16.911	Cleavage	miRNA 21 GUCUCGAGGGAAGUUAGGUUU 1<br>..::::::::::::: ::::<br>Target 992 UGGAGCUCCCUUCAAACCAAU 1012
zma-miR159a-3p	GRMZM2G085550_T02	NA	Putative uncharacterized protein [Source:UniProtKB/TrEMBL;Acc:B6U485]	3	19.999	Cleavage	miRNA 21 GUCUCGAGGGAAGUUAGGUUU 1<br>:.:::: :::::::: :::::<br>Target 886 CGGAGCCCCCUUCAAACCAAA 906
zma-miR159a-3p	GRMZM2G113073_T01	NA	Rac GTPase activating protein 1 [Source:UniProtKB/TrEMBL;Acc:K7UBU1]	3	16.772	Cleavage	miRNA 20 UCUCGAGGGAAGUUAGGUUU 1<br>:: ::::::::::::::<br>Target 122 AGCCCUCCCUUCAAUCCAAC 141
zma-miR159a-3p	GRMZM2G161382_T01	NA	Cyclin delta-3 [Source:UniProtKB/TrEMBL;Acc:B6U905]	3	11.545	Cleavage	miRNA 21 GUCUCGAGGGAAGUUAGGUUU 1<br>.::: ::::::::.:: ::::<br>Target 2090 UAGACCUCCCUUCGAUGCAAA 2110
zma-miR159a-3p	GRMZM2G011588_T01	NA	Uncharacterized protein [Source:UniProtKB/TrEMBL;Acc:C0PDQ8]	3.5	12.326	Translation	miRNA 21 GUCUCGAGGGAAGUUAGGUUU 1<br>::::::::: : : :::::.:<br>Target 4 CAGAGCUCCAUCCCAUCCAGA 24
zma-miR159a-3p	GRMZM2G011588_T02	NA	Uncharacterized protein [Source:UniProtKB/TrEMBL;Acc:C0PDQ8]	3.5	12.326	Translation	miRNA 21 GUCUCGAGGGAAGUUAGGUUU 1<br>::::::::: : : :::::.:<br>Target 4 CAGAGCUCCAUCCCAUCCAGA 24
zma-miR159a-3p	GRMZM2G021364_T01	NA	NA	3.5	21.539	Translation	miRNA 21 GUCUCGAGGGAAGUUAGGUUU 1<br>..:.::::: :::::::::.<br>Target 45 UGGGGCUCCGCUCAAUCCAAG 65
zma-miR159a-3p	GRMZM2G025182_T01	NA	NA	3.5	13.834	Translation	miRNA 20 UCUCGAGGGAAGUUAGGUUU 1<br>:.::::::: ::::::::<br>Target 158 UGGGCUCCCUCCAAUCCAAU 177
zma-miR159a-3p	GRMZM2G025182_T02	NA	NA	3.5	13.834	Translation	miRNA 20 UCUCGAGGGAAGUUAGGUUU 1<br>:.::::::: ::::::::<br>Target 158 UGGGCUCCCUCCAAUCCAAU 177
zma-miR159a-3p	GRMZM2G030444_T01	NA	NA	3.5	20.795	Cleavage	miRNA 20 UCUCGAGGGAAGUUAGGUUU 1<br>: ::.: ::::.:::::.::<br>Target 37 ACAGUUGCCUUUAAUCCGAA 56
zma-miR159a-3p	GRMZM2G057674_T01	NA	Uncharacterized protein [Source:UniProtKB/TrEMBL;Acc:B4FZ32]	3.5	13.245	Translation	miRNA 21 GUCUCGAGGGAAGUUAGGUUU 1<br>::::::: :: ::.::::::<br>Target 266 CAGAGCUGACUACAGUCCAAA 286
zma-miR159a-3p	GRMZM2G057674_T02	NA	Uncharacterized protein [Source:UniProtKB/TrEMBL;Acc:B4FZ32]	3.5	13.245	Translation	miRNA 21 GUCUCGAGGGAAGUUAGGUUU 1<br>::::::: :: ::.::::::<br>Target 706 CAGAGCUGACUACAGUCCAAA 726
zma-miR159a-3p	GRMZM2G063806_T01	NA	NA	3.5	18.861	Translation	miRNA 21 GUCUCG-AGGGAAGUUAGGUUU 1<br>:::::: :.:::: ::::::::<br>Target 2998 CAGAGCAUUCCUUGAAUCCAAA 3019

Potential targets of zma-miRNAs.

miRNA	Target	Gene name	Gene description	Expectation	UPE	Inhibition	Hybrid
zma-miR159a-3p	GRMZM2G064941_T01	NA	NA	3.5	21.539	Translation	miRNA 21 GUCUCGAGGGAAGUUAGGUUU 1<br>..:.:::: :::::::::.<br>Target 45 UGGGGCUCCGCUCAAUCCAAG 65
zma-miR159a-3p	GRMZM2G079746_T01	NA	Fiber protein Fb11 [Source:UniProtKB/TrEMBL;Acc:B6SNA3]	3.5	19.08	Cleavage	miRNA 20 UCUCGAGGGAAGUUAGGUUU 1<br>: ::.:: :::.:::::.::<br>Target 532 ACAGUUCGCUUUAAUCCGAA 551
zma-miR159a-3p	GRMZM2G079746_T02	NA	Fiber protein Fb11 [Source:UniProtKB/TrEMBL;Acc:B6SNA3]	3.5	19.08	Cleavage	miRNA 20 UCUCGAGGGAAGUUAGGUUU 1<br>: ::.:: :::.:::::.::<br>Target 640 ACAGUUCGCUUUAAUCCGAA 659
zma-miR159a-3p	GRMZM2G130724_T01	NA	Uncharacterized protein [Source:UniProtKB/TrEMBL;Acc:B8A1M9]	3.5	14.923	Cleavage	miRNA 21 GUCUCGAGGGAAGUUAGGUUU 1<br>:: :::: .:::::::.::.:<br>Target 683 CAAAGCUGUCUUCAAUUCAGA 703
zma-miR159a-3p	GRMZM2G130724_T02	NA	Uncharacterized protein [Source:UniProtKB/TrEMBL;Acc:B8A1M9]	3.5	14.923	Cleavage	miRNA 21 GUCUCGAGGGAAGUUAGGUUU 1<br>:: :::: .:::::::.::.:<br>Target 749 CAAAGCUGUCUUCAAUUCAGA 769
zma-miR159a-3p	GRMZM2G133464_T01	NA	Serine/threonine-protein phosphatase [Source:UniProtKB/TrEMBL;Acc:B4FAL1]	3.5	17.661	Cleavage	miRNA 21 GUCUCGAGGGAAGUUAGGUUU 1<br>:::: ::.::::::::.:.:<br>Target 1091 CAGAACUUCCUUCAAUUCGAC 1111
zma-miR159a-3p	GRMZM2G133464_T02	NA	Serine/threonine-protein phosphatase [Source:UniProtKB/TrEMBL;Acc:B4FAL1]	3.5	17.661	Cleavage	miRNA 21 GUCUCGAGGGAAGUUAGGUUU 1<br>:::: ::.::::::::.:.:<br>Target 503 CAGAACUUCCUUCAAUUCGAC 523
zma-miR159a-3p	GRMZM2G133464_T03	NA	Serine/threonine-protein phosphatase [Source:UniProtKB/TrEMBL;Acc:B4FAL1]	3.5	17.661	Cleavage	miRNA 21 GUCUCGAGGGAAGUUAGGUUU 1<br>:::: ::.::::::::.:.:<br>Target 496 CAGAACUUCCUUCAAUUCGAC 516
zma-miR159a-3p	GRMZM2G179473_T01	NA	Inositol-tetrakisphosphate 1-kinase 3; Uncharacterized protein [Source:UniProtKB/TrEMBL;Acc:B4FHJ7]	3.5	24.342	Translation	miRNA 20 UCUCGAGGGAAGUUAGGUUU 1<br>::::::.::: ::: ::::.<br>Target 379 AGAGCUUCCUCCAACCCAAG 398
zma-miR159a-3p	GRMZM2G179473_T02	NA	Inositol-tetrakisphosphate 1-kinase 3; Uncharacterized protein [Source:UniProtKB/TrEMBL;Acc:B4FHJ7]	3.5	24.342	Translation	miRNA 20 UCUCGAGGGAAGUUAGGUUU 1<br>::::::.::: ::: ::::.<br>Target 379 AGAGCUUCCUCCAACCCAAG 398
zma-miR159a-3p	GRMZM2G310161_T01	NA	Uncharacterized protein [Source:UniProtKB/TrEMBL;Acc:K7TQH2]	3.5	15.409	Cleavage	miRNA 20 UCUCGAGGGAAGUUAGGUUU 1<br>:::: :.::::::::.::.<br>Target 1084 AGAGAACUCUUCAAUCUAAG 1103
zma-miR159a-3p	GRMZM2G317051_T01	NA	NA	3.5	15.8	Translation	miRNA 21 GUCUCGAGGGAAGUUAGGUUU 1<br>::: ::::::: :::::.::<br>Target 2362 CAGUGCUCCCUGAAAUCCGAA 2382
zma-miR159a-3p	GRMZM2G319109_T01	NA	Uncharacterized protein [Source:UniProtKB/TrEMBL;Acc:K7UJU4]	3.5	16.841	Translation	miRNA 20 UCUCGAGGGAAGUUAGGUUU 1<br>::.::::. :: .:::::::<br>Target 2366 AGGGCUCUGUUAGAUCCAAA 2385
zma-miR159a-3p	GRMZM2G319109_T02	NA	Uncharacterized protein [Source:UniProtKB/TrEMBL;Acc:K7UJU4]	3.5	16.841	Translation	miRNA 20 UCUCGAGGGAAGUUAGGUUU 1<br>::.::::. :: .:::::::<br>Target 854 AGGGCUCUGUUAGAUCCAAA 873
zma-miR159a-3p	GRMZM2G366140_T01	NA	Uncharacterized protein [Source:UniProtKB/TrEMBL;Acc:K7UWD0]	3.5	14.305	Cleavage	miRNA 20 UCUCGAGGGAAGUUAGGUUU 1<br>:::: :.::::::::.::.<br>Target 844 AGAGAACUCUUCAAUCUAAG 863

Potential targets of zma-miRNAs.

miRNA	Target	Gene name	Gene description	Expectation	UPE	Inhibition	Hybrid
zma-miR159a-3p	GRMZM2G380881_T02	NA	Uncharacterized protein [Source:UniProtKB/TrEMBL;Acc:K7UWD0]	3.5	13.344	Cleavage	miRNA 20 UCUCGAGGGAAGUUAGGUUU 1<br>:::: :.::::::::.::.<br>Target 1177 AGAGAACUCUUCAAUCUAAG 1196
zma-miR159a-3p	GRMZM2G410618_T01	NA	NA	3.5	19.736	Cleavage	miRNA 20 UCUCGAG-GGAAGUUAGGUUU 1<br>:::::: :::::.:::::::<br>Target 552 AGAGCUAGCCUUCGAUCCAAA 572
zma-miR159a-3p	GRMZM2G471745_T01	NA	NA	3.5	21.553	Translation	miRNA 21 GUCUCGAGGGAAGUUAGGUUU 1<br>..:.::::: :::::::::.<br>Target 50 UGGGGCUCCGCUCAAUCCAAG 70
zma-miR159a-3p	GRMZM5G860226_T01	NA	NA	3.5	16.876	Cleavage	miRNA 20 UCUCGAGGGAAGUUAGGUUU 1<br>.:. ::.:::::.::.::::<br>Target 681 GGGACUUCCUUCGAUUCAAA 700
zma-miR159a-3p	AC149818.2_FGT008	NA	Uncharacterized protein [Source:UniProtKB/TrEMBL;Acc:K7VLT2]	4	22.258	Translation	miRNA 21 GUCUCGAGGGAAGUUAGGUUU 1<br>:::::::.:.: :::: ::.:<br>Target 774 CAGAGCUUCUUGCAAUACAGA 794
zma-miR159a-3p	GRMZM2G005365_T01	NA	zinc ion binding [Source:Projected from Arabidopsis thaliana (AT5G65740) TAIR;Acc:AT5G65740]	4	23.45	Cleavage	miRNA 20 UCUCGAGGGAAGUUAGGUUU 1<br>::. :::..::::: :::::<br>Target 1065 AGGCCUCUUUUCAAGCCAAA 1084
zma-miR159a-3p	GRMZM2G021514_T01	NA	NA	4	9.728	Cleavage	miRNA 21 GUCUCGAGGGAAGUUAGGUUU 1<br>:: :.: :.::::::::::<br>Target 1086 CAACGUUACUUUCAAUCCAAA 1106
zma-miR159a-3p	GRMZM2G035688_T02	NA	Response regulator [Source:UniProtKB/TrEMBL;Acc:Q9FXQ7]	4	14.825	Cleavage	miRNA 21 GUCUCGAGGGAAGUUAGGUUU 1<br>: :::.: ::::::::...::<br>Target 769 CCGAGUUGCCUUCAAUUUGAA 789
zma-miR159a-3p	GRMZM2G041312_T01	NA	SNF4 [Source:UniProtKB/TrEMBL;Acc:B6TQ12]	4	10.998	Cleavage	miRNA 21 GUCUCGAGGGAAGUUAGGUUU 1<br>:::::: ::::::: :::::<br>Target 79 CAGAGCCCCCUUCACACCAAA 99
zma-miR159a-3p	GRMZM2G041312_T02	NA	SNF4 [Source:UniProtKB/TrEMBL;Acc:B6TQ12]	4	10.998	Cleavage	miRNA 21 GUCUCGAGGGAAGUUAGGUUU 1<br>:::::: ::::::: :::::<br>Target 79 CAGAGCCCCCUUCACACCAAA 99
zma-miR159a-3p	GRMZM2G041312_T03	NA	SNF4 [Source:UniProtKB/TrEMBL;Acc:B6TQ12]	4	16.695	Cleavage	miRNA 21 GUCUCGAGGGAAGUUAGGUUU 1<br>:::::: ::::::: :::::<br>Target 214 CAGAGCCCCCUUCACACCAAA 234
zma-miR159a-3p	GRMZM2G049510_T01	NA	NA	4	14.91	Translation	miRNA 20 UCUCGAGGGAAGUUAGGUUU 1<br>:::::..: ::.:::::.<br>Target 2001 UGAGCUCUUUACAGUCCAAG 2020
zma-miR159a-3p	GRMZM2G052890_T01	NA	ZAG1 protein; Zea AGAMOUS-like protein [Source:UniProtKB/TrEMBL;Acc:Q41876]	4	22.692	Translation	miRNA 20 UCUCGAGGGAAGUUAGGUUU 1<br>.:::::.::: ::.:.:::<br>Target 891 GGAGCUUCCUGCAGUUCAAC 910
zma-miR159a-3p	GRMZM2G057402_T01	NA	Uncharacterized protein [Source:UniProtKB/TrEMBL;Acc:K7U5Z5]	4	13.655	Cleavage	miRNA 20 UCUCGAGGGAAGUUAGGUUU 1<br>::: .:.:.::::::.:::<br>Target 5026 AGAAUUUCUUUCAAUUCAAU 5045
zma-miR159a-3p	GRMZM2G057402_T02	NA	Uncharacterized protein [Source:UniProtKB/TrEMBL;Acc:K7U5Z5]	4	13.655	Cleavage	miRNA 20 UCUCGAGGGAAGUUAGGUUU 1<br>::: .:.:.::::::.:::<br>Target 5026 AGAAUUUCUUUCAAUUCAAU 5045

miRNA	Target	Gene name	Gene description	Expectation	UPE	Inhibition	Hybrid
zma-miR159a-3p	GRMZM2G057402_T03	NA	Uncharacterized protein [Source:UniProtKB/TrEMBL;Acc:K7U5Z5]	4	13.655	Cleavage	miRNA 20 UCUCGAGGGAAGUUAGGUUU 1<br>::: .:.:.:::::.:::<br>Target 5026 AGAAUUUCUUUCAAUUCAAU 5045
zma-miR159a-3p	GRMZM2G059893_T01	NA	Uncharacterized protein [Source:UniProtKB/TrEMBL;Acc:B4FID3]	4	19.828	Translation	miRNA 21 GUCUCGAGGGAAGUUAGGUUU 1<br>:: :: ::.:: :::::::.<br>Target 854 CACAGGUCUCUCCAAUCCAAG 874
zma-miR159a-3p	GRMZM2G059893_T02	NA	Uncharacterized protein [Source:UniProtKB/TrEMBL;Acc:B4FID3]	4	19.828	Translation	miRNA 21 GUCUCGAGGGAAGUUAGGUUU 1<br>:: :: ::.:: :::::::.<br>Target 585 CACAGGUCUCUCCAAUCCAAG 605
zma-miR159a-3p	GRMZM2G059893_T03	NA	Uncharacterized protein [Source:UniProtKB/TrEMBL;Acc:B4FID3]	4	19.828	Translation	miRNA 21 GUCUCGAGGGAAGUUAGGUUU 1<br>:: :: ::.:: :::::::.<br>Target 296 CACAGGUCUCUCCAAUCCAAG 316
zma-miR159a-3p	GRMZM2G059893_T04	NA	Uncharacterized protein [Source:UniProtKB/TrEMBL;Acc:B4FID3]	4	19.828	Translation	miRNA 21 GUCUCGAGGGAAGUUAGGUUU 1<br>:: :: ::.:: :::::::.<br>Target 296 CACAGGUCUCUCCAAUCCAAG 316
zma-miR159a-3p	GRMZM2G062854_T01	NA	ATP synthase subunit beta [Source:UniProtKB/TrEMBL;Acc:K7UV85]	4	11.051	Cleavage	miRNA 21 GUCUCGAGGGAAGUUAGGUUU 1<br>:::: . :::::.:::..:::<br>Target 3082 CAGAAUACCCUUUAAUUUAAA 3102
zma-miR159a-3p	GRMZM2G073540_T01	NA	Acyl-desaturase [Source:UniProtKB/TrEMBL;Acc:B6TXC6]	4	18.532	Translation	miRNA 20 UCUCGAGGGAAGUUAGGUUU 1<br>::::.:.: ::::::::<br>Target 1273 CUAGCUUCUUCCAAUCCAAA 1292
zma-miR159a-3p	GRMZM2G084252_T01	NA	NA	4	24.991	Translation	miRNA 21 GUCUCGAGGGAAGUUAGGUUU 1<br>:.:::.::.:: : :::::.:<br>Target 1757 CGGAGUUCUCUGCCAUCCAGA 1777
zma-miR159a-3p	GRMZM2G086030_T01	NA	Uncharacterized protein [Source:UniProtKB/TrEMBL;Acc:K7TWZ6]	4	15.832	Cleavage	miRNA 20 UCUCGAGGGAAGUUAGGUUU 1<br>:::: :.:.::::::.::.<br>Target 4353 AGAGAUUCUUUCAAUUCAGC 4372
zma-miR159a-3p	GRMZM2G097021_T01	NA	Gb protein; Uncharacterized protein [Source:UniProtKB/TrEMBL;Acc:C0P5V0]	4	19.84	Cleavage	miRNA 21 GUCUCGAGGGAAGUUAGGUUU 1<br>:::.: ::::::. .::::.:<br>Target 359 CAGGGAUCCCUUUUGUCCAGA 379
zma-miR159a-3p	GRMZM2G104546_T01	AKHSDH2	Bifunctional aspartokinase/homoserine dehydrogenase 2	4	13.178	Translation	miRNA 21 GUCUCGAGGGAAGUUAGGUUU 1<br>.: ::.::.:: ::::..:::<br>Target 751 UAUAGUUCUCUACAAUUUAAA 771
zma-miR159a-3p	GRMZM2G108919_T01	NA	Polyadenylate-binding protein 2 [Source:UniProtKB/TrEMBL;Acc:B6T3R4]	4	24.847	Translation	miRNA 21 GUCUCGAGGGAAGUUAGGUUU 1<br>.:::::::.: : ::::::.<br>Target 1094 UAGAGCUCUCCUAAAUCCAGC 1114
zma-miR159a-3p	GRMZM2G108919_T07	NA	Polyadenylate-binding protein 2 [Source:UniProtKB/TrEMBL;Acc:B6T3R4]	4	24.847	Translation	miRNA 21 GUCUCGAGGGAAGUUAGGUUU 1<br>.:::::::.: : ::::::.<br>Target 1430 UAGAGCUCUCCUAAAUCCAGC 1450
zma-miR159a-3p	GRMZM2G111529_T01	NA	Uncharacterized protein [Source:UniProtKB/TrEMBL;Acc:C0PCF0]	4	15.479	Cleavage	miRNA 20 UCUCGAGGGAAGUUAGGUUU 1<br>::::::.: ::::: .:::.<br>Target 435 AGAGCUUCAUUCAAAUCAAG 454
zma-miR159a-3p	GRMZM2G135713_T01	NA	NA	4	5.18	Cleavage	miRNA 21 GUCUCGAGGGAAGUUAGGUUU 1<br>.: ::::.::::::.::. ::<br>Target 84 UAAAGCUUCCUUCAGUCUCAA 104

Potential targets of zma-miRNAs.

miRNA	Target	Gene name	Gene description	Expectation	UPE	Inhibition	Hybrid
zma-miR159a-3p	GRMZM2G149295_T01	NA	Cupin	4	18.629	Cleavage	miRNA 20 UCUCGAGGGAAGUUAGGUUU 1<br>.:: ::.:.:::..:::.::<br>Target 206 GGAACUUCUUUCGGUCCGAA 225
zma-miR159a-3p	GRMZM2G149903_T01	NA	Inositol-tetrakisphosphate 1-kinase 3; Uncharacterized protein [Source:UniProtKB/TrEMBL;Acc:B6TDJ5]	4	21.233	Translation	miRNA 20 UCUCGAGGGAAGUUAGGUUU 1<br>::::::.::: ::. ::::.<br>Target 499 AGAGCUUCCUGCAGCCCAAG 518
zma-miR159a-3p	GRMZM2G149903_T02	NA	Inositol-tetrakisphosphate 1-kinase 3; Uncharacterized protein [Source:UniProtKB/TrEMBL;Acc:B6TDJ5]	4	21.233	Translation	miRNA 20 UCUCGAGGGAAGUUAGGUUU 1<br>::::::.::: ::. ::::.<br>Target 499 AGAGCUUCCUGCAGCCCAAG 518
zma-miR159a-3p	GRMZM2G157729_T01	NA	CONTAINS InterPro DOMAIN/s: Uncharacterised conserved protein UCP017207	4	18.93	Translation	miRNA 20 UCUCGAGGGAAGUUAGGUUU 1<br>::.:::::: :.:::..::<br>Target 670 AGGGCUCCCCUUAAUUUAAU 689
zma-miR159a-3p	GRMZM2G167957_T01	NA	Inositol-tetrakisphosphate 1-kinase 3 [Source:UniProtKB/TrEMBL;Acc:B6TBA5]	4	24.63	Translation	miRNA 20 UCUCGAGGGAAGUUAGGUUU 1<br>::::::.::: ::. ::::.<br>Target 396 AGAGCUUCCUGCAGCCCAAG 415
zma-miR159a-3p	GRMZM2G167957_T02	NA	Inositol-tetrakisphosphate 1-kinase 3 [Source:UniProtKB/TrEMBL;Acc:B6TBA5]	4	24.63	Translation	miRNA 20 UCUCGAGGGAAGUUAGGUUU 1<br>::::::.::: ::. ::::.<br>Target 396 AGAGCUUCCUGCAGCCCAAG 415
zma-miR159a-3p	GRMZM2G181378_T01	NA	Uncharacterized protein [Source:UniProtKB/TrEMBL;Acc:B7ZZW0]	4	13.515	Cleavage	miRNA 20 UCUCGAGGGAAGUUAGGUUU 1<br>:::::..::::.:::.::<br>Target 1843 CAAGCUCUUUUCAGUCCGAA 1862
zma-miR159a-3p	GRMZM2G317287_T01	NA	Uncharacterized protein [Source:UniProtKB/TrEMBL;Acc:K7TLM6]	4	19.438	Translation	miRNA 20 UCUCGAGGGAAGUUAGGUUU 1<br>::::.:.: ::::::::<br>Target 1826 CUAGCUUCUUCCAAUCCAAA 1845
zma-miR159a-3p	GRMZM2G329636_T01	NA	ATP synthase subunit a [Source:UniProtKB/Swiss-Prot;Acc:P07925]	4	21.62	Cleavage	miRNA 20 UCUCGAGGGAAGUUAGGUUU 1<br>:.:::.:.:::::: :::<br>Target 1146 UGGGCUUCUUUCAAUCGAAA 1165
zma-miR159a-3p	GRMZM2G371651_T01	NA	Uncharacterized protein [Source:UniProtKB/TrEMBL;Acc:K7VC66]	4	15.303	Cleavage	miRNA 21 GUCUCGAGGGAAGUUAGGUUU 1<br>::::::::: :::. ::::::<br>Target 86 CAGAGCUCC-UUCGCUCCAAA 105
zma-miR159a-3p	GRMZM2G386944_T01	NA	Uncharacterized protein [Source:UniProtKB/TrEMBL;Acc:K7V9U6]	4	21.237	Cleavage	miRNA 21 GUCUCGAGGGAAGUUAGGUUU 1<br>:.: ::::::::::: ::::<br>Target 690 CGGUGCUCCCUUCAAACCAAU 710
zma-miR159a-3p	GRMZM2G388148_T01	NA	Uncharacterized protein [Source:UniProtKB/TrEMBL;Acc:C0HEZ6]	4	14.268	Translation	miRNA 20 UCUCGAGGGAAGUUAGGUUU 1<br>:.:::::: ::..::.:::<br>Target 1523 CGGGCUCCCAUCGGUCUAAA 1542
zma-miR159a-3p	GRMZM2G388148_T02	NA	Uncharacterized protein [Source:UniProtKB/TrEMBL;Acc:C0HEZ6]	4	14.268	Translation	miRNA 20 UCUCGAGGGAAGUUAGGUUU 1<br>:.:::::: ::..::.:::<br>Target 1523 CGGGCUCCCAUCGGUCUAAA 1542
zma-miR159a-3p	GRMZM2G405592_T01	NA	Uncharacterized protein [Source:UniProtKB/TrEMBL;Acc:K7V0B1]	4	19.435	Cleavage	miRNA 21 GUCUCGAGGGAAGUUAGGUUU 1<br>::::::::: :::. ::::::<br>Target 31 CAGAGCUCC-UUCGCUCCAAA 50
zma-miR159a-3p	GRMZM2G427337_T01	NA	Uncharacterized protein [Source:UniProtKB/TrEMBL;Acc:K7UN57]	4	18.952	Translation	miRNA 21 GUCUCGAGGGAAGUUAGGUUU 1<br>::: :::.::: ::::.:..:<br>Target 505 CAGUGCUUCCUCCAAUUCGGA 525

Potential targets of zma-miRNAs.

miRNA	Target	Gene name	Gene description	Expectation	UPE	Inhibition	Hybrid
zma-miR159a-3p	GRMZM2G434669_T01	NA	Putative uncharacterized protein [Source:UniProtKB/TrEMBL;Acc:B6TT22]	4	21.788	Translation	miRNA 20 UCUCGAGGGAAGUUAGGUUU 1<br>:::::.:: .::::::..<br>Target 1265 CGAGCUCUCUGUAAUCCAGG 1284
zma-miR159a-3p	GRMZM2G449496_T01	NA	plastid transcriptionally active 16 [Source:Projected from Arabidopsis thaliana (AT3G46780) TAIR;Acc:AT3G46780]	4	16.144	Translation	miRNA 20 UCUCGAGGGAAGUUAGGUUU 1<br>.:.:::: :::::::::<br>Target 21 CUGGUUCCCCUCAAUCCAAA 40
zma-miR159a-3p	GRMZM2G451374_T01	NA	NA	4	16.299	Cleavage	miRNA 21 GUCUCGAGGGAAGUUAGGUUU 1<br>:::. ::.:.:::..::::.:<br>Target 735 CAGGACUUCUUUCGGUCCAGA 755
zma-miR159a-3p	GRMZM2G451605_T01	NA	Uncharacterized protein [Source:UniProtKB/TrEMBL;Acc:K7UTR1]	4	22.374	Cleavage	miRNA 21 GUCUCGAGGGAAGUUAGGUUU 1<br>:.: ::::::::::: ::::<br>Target 690 CGGUGCUCCCUUCAAACCAAU 710
zma-miR159a-3p	GRMZM2G452633_T01	NA	Uncharacterized protein [Source:UniProtKB/TrEMBL;Acc:C0HE67]	4	15.457	Cleavage	miRNA 21 GUCUCGAGGGAAGUUAGGUUU 1<br>:: ::::.:::: ::: :::<br>Target 395 CACAGCUCUCUUCCAUCAAAA 415
zma-miR159a-3p	GRMZM2G452633_T02	NA	Uncharacterized protein [Source:UniProtKB/TrEMBL;Acc:C0HE67]	4	15.457	Cleavage	miRNA 21 GUCUCGAGGGAAGUUAGGUUU 1<br>:: ::::.:::: ::: :::<br>Target 802 CACAGCUCUCUUCCAUCAAAA 822
zma-miR159a-3p	GRMZM2G452633_T03	NA	Uncharacterized protein [Source:UniProtKB/TrEMBL;Acc:C0HE67]	4	15.457	Cleavage	miRNA 21 GUCUCGAGGGAAGUUAGGUUU 1<br>:: ::::.:::: ::: :::<br>Target 685 CACAGCUCUCUUCCAUCAAAA 705
zma-miR159a-3p	GRMZM2G465806_T01	NA	NA	4	18.736	Cleavage	miRNA 21 GUCUCGAGGGAAGUUAGGUUU 1<br>:::::.:..:::. ::..:::<br>Target 756 CAGAGUUUUCUUUUAUUUAAA 776
zma-miR159a-3p	GRMZM5G856777_T01	PSBC	Photosystem II CP43 chlorophyll apoprotein [Source:UniProtKB/Swiss-Prot;Acc:P48187]	4	20.347	Translation	miRNA 21 GUCUCGAGGGAAGUUAGGUUU 1<br>:::: .:::.: ::::.:.::<br>Target 1578 CAGAAUUCCUUACAAUUCGAA 1598
zma-miR159a-3p	GRMZM5G856812_T01	NA	Uncharacterized protein [Source:UniProtKB/TrEMBL;Acc:K7W3G1]	4	21.144	Translation	miRNA 20 UCUCGAGGGAAGUUAGGUUU 1<br>:::::..: ::.:::::.<br>Target 1712 UGAGCUCUUUACAGUCCAAG 1731
zma-miR159a-3p	GRMZM5G862565_T01	NA	NA	4	12.914	Cleavage	miRNA 20 UCUCGAGGGAAGUUAGGUUU 1<br>::.:::.:.::.:.:...::<br>Target 2560 AGGGCUUCUUUUAGUUUGAA 2579
zma-miR159a-3p	AC183315.4_FGT006	NA	NA	4.5	23.997	Cleavage	miRNA 20 UCUCGAGGGAAGUUAGGUUU 1<br>::.:::::::::. ::.:<br>Target 506 AGGGCUCCCUUCAGGGCAGA 525
zma-miR159a-3p	GRMZM2G010302_T01	NA	DIP1; Uncharacterized protein [Source:UniProtKB/TrEMBL;Acc:B4FH94]	4.5	14.203	Translation	miRNA 21 GUCUCGAGGGAAGUUAGGUUU 1<br>..:::.:::.:: .::.:.:.<br>Target 1311 UGGAGUUCCUUUAGAUUCGAG 1331
zma-miR159a-3p	GRMZM2G010302_T02	NA	DIP1; Uncharacterized protein [Source:UniProtKB/TrEMBL;Acc:B4FH94]	4.5	14.203	Translation	miRNA 21 GUCUCGAGGGAAGUUAGGUUU 1<br>..:::.:::.:: .::.:.:.<br>Target 1244 UGGAGUUCCUUUAGAUUCGAG 1264
zma-miR159a-3p	GRMZM2G016184_T01	NA	Uncharacterized protein [Source:UniProtKB/TrEMBL;Acc:B4FRP7]	4.5	12.029	Cleavage	miRNA 20 UCUCGAGGGAAGUUAGGUUU 1<br>:::::::.::.: :.:::.<br>Target 502 UGAGCUCCUUUUAUUUCAAG 521

Potential targets of zma-miRNAs.

miRNA	Target	Gene name	Gene description	Expectation	UPE	Inhibition	Hybrid
zma-miR159a-3p	GRMZM2G016184_T02	NA	Uncharacterized protein [Source:UniProtKB/TrEMBL;Acc:B4FRP7]	4.5	12.768	Cleavage	miRNA 20 UCUCGAGGGAAGUUAGGUUU 1<br>:::::::.::.: :.:::.<br>Target 628 UGAGCUCCUUUUAUUUCAAG 647
zma-miR159a-3p	GRMZM2G016184_T03	NA	Uncharacterized protein [Source:UniProtKB/TrEMBL;Acc:B4FRP7]	4.5	11.611	Cleavage	miRNA 20 UCUCGAGGGAAGUUAGGUUU 1<br>:::::::.::.: :.:::.<br>Target 329 UGAGCUCCUUUUAUUUCAAG 348
zma-miR159a-3p	GRMZM2G016774_T01	NA	NA	4.5	12.988	Cleavage	miRNA 20 UCUCGAGGGAAGUUAGGUUU 1<br>::::.:...:::: :.::.:<br>Target 51 AGAGUUUUUUUCACUUCAGA 70
zma-miR159a-3p	GRMZM2G027272_T01	NA	Nitrate-induced NOI protein; Uncharacterized protein [Source:UniProtKB/TrEMBL;Acc:B4FE44]	4.5	15.183	Cleavage	miRNA 21 GUCUCGAGGGAAGUUAGGUUU 1<br>:::::::.::::::::::<br>Target 649 CAGAGCUUCCUUCAAUCCUCC 669
zma-miR159a-3p	GRMZM2G027431_T01	NA	Putative endonuclease or glycosyl hydrolase [Source:Projected from Arabidopsis thaliana (AT2G15560) TAIR;Acc:AT2G15560]	4.5	21.555	Translation	miRNA 21 GUCUCGAGGGAAGUUAGGUUU 1<br>:::: ::::: :::::: ::<br>Target 1318 CAGACCUCCCGUCAAUCAAAU 1338
zma-miR159a-3p	GRMZM2G027431_T02	NA	Putative endonuclease or glycosyl hydrolase [Source:Projected from Arabidopsis thaliana (AT2G15560) TAIR;Acc:AT2G15560]	4.5	21.555	Translation	miRNA 21 GUCUCGAGGGAAGUUAGGUUU 1<br>:::: ::::: :::::: ::<br>Target 1072 CAGACCUCCCGUCAAUCAAAU 1092
zma-miR159a-3p	GRMZM2G027955_T01	NA	Glucose-1-phosphate adenylyltransferase large subunit 2	4.5	18.113	Cleavage	miRNA 21 GUCUCGAGGGAAGUUAGGUUU 1<br>:::: ::..:::..:: ::::<br>Target 1619 CAGAUCUUUCUUUGAUGCAAA 1639
zma-miR159a-3p	GRMZM2G027955_T02	NA	Glucose-1-phosphate adenylyltransferase large subunit 2	4.5	15.968	Cleavage	miRNA 21 GUCUCGAGGGAAGUUAGGUUU 1<br>:::: ::..:::..:: ::::<br>Target 1343 CAGAUCUUUCUUUGAUGCAAA 1363
zma-miR159a-3p	GRMZM2G027955_T03	NA	Glucose-1-phosphate adenylyltransferase large subunit 2	4.5	15.968	Cleavage	miRNA 21 GUCUCGAGGGAAGUUAGGUUU 1<br>:::: ::..:::..:: ::::<br>Target 1373 CAGAUCUUUCUUUGAUGCAAA 1393
zma-miR159a-3p	GRMZM2G035514_T01	NA	NA	4.5	20.286	Translation	miRNA 21 GUCUCGAGGGAAGUUAGGUUU 1<br>:.::::::::: ::: .:.:.<br>Target 1279 CGGAGCUCCCUCCAACUCGAG 1299
zma-miR159a-3p	GRMZM2G044281_T01	NA	NA	4.5	17.112	Cleavage	miRNA 20 UCUCGAGGGAAGUUAGGUUU 1<br>:.:.:::.::.: ::::::<br>Target 1903 CGGGUUCCUUUUAUUCCAAA 1922
zma-miR159a-3p	GRMZM2G044281_T02	NA	NA	4.5	17.112	Cleavage	miRNA 20 UCUCGAGGGAAGUUAGGUUU 1<br>:.:.:::.::.: ::::::<br>Target 1903 CGGGUUCCUUUUAUUCCAAA 1922
zma-miR159a-3p	GRMZM2G044281_T03	NA	NA	4.5	17.112	Cleavage	miRNA 20 UCUCGAGGGAAGUUAGGUUU 1<br>:.:.:::.::.: ::::::<br>Target 1903 CGGGUUCCUUUUAUUCCAAA 1922
zma-miR159a-3p	GRMZM2G051403_T01	NA	Uncharacterized protein [Source:UniProtKB/TrEMBL;Acc:B4FSG1]	4.5	9.594	Cleavage	miRNA 21 GUCUCGAGGGAAGUUAGGUUU 1<br>.::.:.: :.:::. ::::::<br>Target 1541 UAGGGUUACUUUCGUUCCAAA 1561
zma-miR159a-3p	GRMZM2G052105_T01	NA	Putative uncharacterized protein [Source:UniProtKB/TrEMBL;Acc:B6TG05]	4.5	18.445	Cleavage	miRNA 20 UCUCGAGGGAAGUUAGGUUU 1<br>::::..::::::.::::<br>Target 537 CCUGCUCUUUUCAAUUCAAA 556

Potential targets of zma-miRNAs.

miRNA	Target	Gene name	Gene description	Expectation	UPE	Inhibition	Hybrid
zma-miR159a-3p	GRMZM2G080439_T01	NA	Uncharacterized protein [Source:UniProtKB/TrEMBL;Acc:C4J431]	4.5	19.341	Cleavage	miRNA 20 UCUCGAGGGAAGUUAGGUUU 1<br>::::..::::.:.:.::<br>Target 1824 CAAGCUCUUUUCAGUUCGAA 1843
zma-miR159a-3p	GRMZM2G090935_T02	NA	Seed specific protein Bn15D14A; Seed specific protein Bn15D14A isoform 1; Seed specific protein Bn15D14A isoform 2; Uncharacterized protein [Source:UniProtKB/TrEMBL;Acc:B6T3N0]	4.5	11.894	Cleavage	miRNA 21 GUCUCGAGGGAAGUUAGGUUU 1<br>:: :::::..::: ::: :::<br>Target 663 CACAGCUCUUUUCCAUCAAAA 683
zma-miR159a-3p	GRMZM2G095469_T01	NA	NA	4.5	16.362	Translation	miRNA 21 GUCUCGAGGGAAGUUAGGUUU 1<br>:::: .:::.: .::::..::<br>Target 925 CAGAUUUCCUUGUAAUCUGAA 945
zma-miR159a-3p	GRMZM2G109787_T01	NA	NA	4.5	19.762	Cleavage	miRNA 21 GUCUCGAGGGAAGUUAGGUUU 1<br>:: ::.:::.::..::.::..<br>Target 667 CACAGUUCCUUUUGAUUCAGG 687
zma-miR159a-3p	GRMZM2G116087_T02	NA	Chorismate mutase; Uncharacterized protein [Source:UniProtKB/TrEMBL;Acc:B4G0R4]	4.5	16.146	Cleavage	miRNA 20 UCUCGAGGGAAGUUAGGUUU 1<br>::::..:::..::.::::<br>Target 1515 UCAGCUUUCUUUGAUUCAAA 1534
zma-miR159a-3p	GRMZM2G116087_T03	NA	Chorismate mutase; Uncharacterized protein [Source:UniProtKB/TrEMBL;Acc:B4G0R4]	4.5	16.146	Cleavage	miRNA 20 UCUCGAGGGAAGUUAGGUUU 1<br>::::..:::..::.::::<br>Target 1494 UCAGCUUUCUUUGAUUCAAA 1513
zma-miR159a-3p	GRMZM2G119546_T01	NA	Serine/threonine-protein phosphatase [Source:UniProtKB/TrEMBL;Acc:B4FQJ0]	4.5	17.613	Translation	miRNA 21 GUCUCGAGGGAAGUUAGGUUU 1<br>:::: ::.::: ::::.:.:<br>Target 1117 CAGAACUUCCUCCAAUUCGAC 1137
zma-miR159a-3p	GRMZM2G119546_T02	NA	Serine/threonine-protein phosphatase [Source:UniProtKB/TrEMBL;Acc:B4FQJ0]	4.5	17.613	Translation	miRNA 21 GUCUCGAGGGAAGUUAGGUUU 1<br>:::: ::.::: ::::.:.:<br>Target 1225 CAGAACUUCCUCCAAUUCGAC 1245
zma-miR159a-3p	GRMZM2G120954_T03	NA	Uncharacterized protein [Source:UniProtKB/TrEMBL;Acc:B4FKT7]	4.5	20.985	Cleavage	miRNA 20 UCUCGAGGGAAGUUAGGUUU 1<br>::: :::::::.:::.:.<br>Target 332 UCAGCGCCCUUCAGUCCGAG 351
zma-miR159a-3p	GRMZM2G121460_T01	NA	NA	4.5	14.995	Cleavage	miRNA 21 GUCUCGAGGGAAGUUAGGUUU 1<br>..:: .:.:.::...::::::<br>Target 855 UGGAAUUUCUUUUGGUCCAAA 875
zma-miR159a-3p	GRMZM2G132084_T01	NA	Uncharacterized protein [Source:UniProtKB/TrEMBL;Acc:B4FPQ5]	4.5	12.965	Cleavage	miRNA 21 GUCUCGAGGGAAGUUAGGUUU 1<br>: :::::::.::.: :.:::.<br>Target 329 CUGAGCUCCUUUUAUUUCAAG 349
zma-miR159a-3p	GRMZM2G132084_T02	NA	Uncharacterized protein [Source:UniProtKB/TrEMBL;Acc:B4FPQ5]	4.5	14.091	Cleavage	miRNA 21 GUCUCGAGGGAAGUUAGGUUU 1<br>: :::::::.::.: :.:::.<br>Target 329 CUGAGCUCCUUUUAUUUCAAG 349
zma-miR159a-3p	GRMZM2G132084_T03	NA	Uncharacterized protein [Source:UniProtKB/TrEMBL;Acc:B4FPQ5]	4.5	15.933	Cleavage	miRNA 21 GUCUCGAGGGAAGUUAGGUUU 1<br>: :::::::.::.: :.:::.<br>Target 332 CUGAGCUCCUUUUAUUUCAAG 352
zma-miR159a-3p	GRMZM2G132084_T04	NA	Uncharacterized protein [Source:UniProtKB/TrEMBL;Acc:B4FPQ5]	4.5	14.091	Cleavage	miRNA 21 GUCUCGAGGGAAGUUAGGUUU 1<br>: :::::::.::.: :.:::.<br>Target 442 CUGAGCUCCUUUUAUUUCAAG 462
zma-miR159a-3p	GRMZM2G135706_T01	NA	NA	4.5	23.504	Cleavage	miRNA 20 UCUCGAGGGAAGUUAGGUUU 1<br>::.::::::::::. ::.:<br>Target 1056 AGGGCUCCCUUCAGGGCAGA 1075

Potential targets of zma-miRNAs.

miRNA	Target	Gene name	Gene description	Expectation	UPE	Inhibition	Hybrid
zma-miR159a-3p	GRMZM2G135706_T02	NA	NA	4.5	23.504	Cleavage	miRNA 20 UCUCGAGGGAAGUUAGGUUU 1<br>::.:::::::::. ::.:<br>Target 504 AGGGCUCCCUUCAGGGCAGA 523
zma-miR159a-3p	GRMZM2G138349_T01	NA	Uncharacterized protein [Source:UniProtKB/TrEMBL;Acc:C0PAH7]	4.5	16.089	Cleavage	miRNA 20 UCUCGAGGGAAGUUAGGUUU 1<br>::.::::.::::: .:::<br>Target 1429 AGGGCUCUCUUCAAAAUAAA 1448
zma-miR159a-3p	GRMZM2G138349_T02	NA	Uncharacterized protein [Source:UniProtKB/TrEMBL;Acc:C0PAH7]	4.5	19.287	Cleavage	miRNA 20 UCUCGAGGGAAGUUAGGUUU 1<br>::.::::.::::: .:::<br>Target 1333 AGGGCUCUCUUCAAAAUAAA 1352
zma-miR159a-3p	GRMZM2G138349_T03	NA	Uncharacterized protein [Source:UniProtKB/TrEMBL;Acc:C0PAH7]	4.5	16.089	Cleavage	miRNA 20 UCUCGAGGGAAGUUAGGUUU 1<br>::.::::.::::: .:::<br>Target 1269 AGGGCUCUCUUCAAAAUAAA 1288
zma-miR159a-3p	GRMZM2G138349_T04	NA	Uncharacterized protein [Source:UniProtKB/TrEMBL;Acc:C0PAH7]	4.5	16.089	Cleavage	miRNA 20 UCUCGAGGGAAGUUAGGUUU 1<br>::.::::.::::: .:::<br>Target 980 AGGGCUCUCUUCAAAAUAAA 999
zma-miR159a-3p	GRMZM2G144873_T06	NA	Galactosyltransferase family protein [Source:Projected from Arabidopsis thaliana (AT1G32930) TAIR;Acc:AT1G32930]	4.5	18.835	Cleavage	miRNA 20 UCUCGAGGGAAGUUAGGUUU 1<br>.:.::::..::::: ::..<br>Target 940 GGGGCUCUUUUCAAUACAGG 959
zma-miR159a-3p	GRMZM2G158237_T03	NA	Peptidyl-prolyl cis-trans isomerase [Source:UniProtKB/TrEMBL;Acc:B4FJW5]	4.5	22.802	Cleavage	miRNA 20 UCUCGAGGGAAGUUAGGUUU 1<br>::.:.::::: ::.::::<br>Target 1032 UAAGUUUCCUUCCAUUCAAA 1051
zma-miR159a-3p	GRMZM2G158237_T04	NA	Peptidyl-prolyl cis-trans isomerase [Source:UniProtKB/TrEMBL;Acc:B4FJW5]	4.5	22.802	Cleavage	miRNA 20 UCUCGAGGGAAGUUAGGUUU 1<br>::.:.::::: ::.::::<br>Target 1268 UAAGUUUCCUUCCAUUCAAA 1287
zma-miR159a-3p	GRMZM2G180568_T01	NA	NA	4.5	18.515	Cleavage	miRNA 21 GUCUCGAGGGAAGUUAGGUUU 1<br>:.:.:: : ::::.::::<br>Target 852 CGGGGCACACUUCAGUCCAAU 872
zma-miR159a-3p	GRMZM2G305124_T01	NA	ARABIDOPSIS TRITHORAX-RELATED PROTEIN 6 [Source:Projected from Arabidopsis thaliana (AT5G24330) TAIR;Acc:AT5G24330]	4.5	23.514	Translation	miRNA 21 GUCUCGAGGGAAGUUAGGUUU 1<br>:::::.:::: ...::::.:<br>Target 374 CAGAGUUCCCGCUGGUCCAGA 394
zma-miR159a-3p	GRMZM2G305148_T01	NA	NA	4.5	11.537	Cleavage	miRNA 21 GUCUCGAGGGAAGUUAGGUUU 1<br>:: .:.:. .::::::.:::<br>Target 1078 CAAGGUUUGUUUCAAUCUAAA 1098
zma-miR159a-3p	GRMZM2G370275_T01	NA	NA	4.5	18.741	Translation	miRNA 21 GUCUCGAGGGAAGUUAGGUUU 1<br>..::::.:.:: :: ::::.<br>Target 486 UGGAGCUUCUUUGAAGCCAAG 506
zma-miR159a-3p	GRMZM2G374085_T01	NA	Uncharacterized protein [Source:UniProtKB/TrEMBL;Acc:B4FCZ3]	4.5	23.911	Translation	miRNA 21 GUCUCGAGGGAAGUUAGGUUU 1<br>:.::::::. ::: :::::<br>Target 313 CGGAGCUCCUAUCAUUCCAAU 333
zma-miR159a-3p	GRMZM2G374085_T02	NA	Uncharacterized protein [Source:UniProtKB/TrEMBL;Acc:B4FCZ3]	4.5	23.911	Translation	miRNA 21 GUCUCGAGGGAAGUUAGGUUU 1<br>:.::::::. ::: :::::<br>Target 285 CGGAGCUCCUAUCAUUCCAAU 305
zma-miR159a-3p	GRMZM2G383234_T01	NA	Uncharacterized protein [Source:UniProtKB/TrEMBL;Acc:K7UNJ2]	4.5	18.336	Translation	miRNA 21 GUCUCGAGGGAAGUUAGGUUU 1<br>:::: ::.::: ::::.:.:<br>Target 102 CAGAACUUCCUCCAAUUCGAC 122

miRNA	Target	Gene name	Gene description	Expectation	UPE	Inhibition	Hybrid
zma-miR159a-3p	GRMZM2G416491_T01	NA	NA	4.5	18.737	Translation	miRNA 21 GUCUCGAGGGAAGUUAGGUUU 1<br>:::::::.: :::.: ::::<br>Target 764 CAGAGCUUCAGUCAGUGCAAA 784
zma-miR159a-3p	GRMZM2G416491_T02	NA	NA	4.5	17.134	Translation	miRNA 21 GUCUCGAGGGAAGUUAGGUUU 1<br>:::::::.: :::.: ::::<br>Target 121 CAGAGCUUCAGUCAGUGCAAA 141
zma-miR159a-3p	GRMZM2G429237_T01	NA	Cysteine-type endopeptidase/ ubiquitin thiolesterase [Source:UniProtKB/TrEMBL;Acc:K7TVB6]	4.5	20.004	Cleavage	miRNA 20 UCUCGAGGGAAGUUAGGUUU 1<br>.::::::::: :::..::<br>Target 1515 CUGGCUCCCUUCUAUCUGAA 1534
zma-miR159a-3p	GRMZM2G476902_T01	NA	Putative ARM repeat-containing protein containing family protein [Source:UniProtKB/TrEMBL;Acc:K7V204]	4.5	23.504	Cleavage	miRNA 20 UCUCGAGGGAAGUUAGGUUU 1<br>::.::::::::. ::.:<br>Target 714 AGGGCUCCCUUCAGGGCAGA 733
zma-miR159a-3p	AC234202.1_FGT005	NA	Uncharacterized protein [Source:UniProtKB/TrEMBL;Acc:K7U7E4]	5	19.192	Cleavage	miRNA 21 GUCUCGAGGGAAGUUAGGUUU 1<br>:::::::..:::. ::.: .:<br>Target 486 CAGAGCUUUCUUUUAUUCUGA 506
zma-miR159a-3p	GRMZM2G034804_T07	NA	Nuclear cap-binding protein subunit 2; Uncharacterized protein [Source:UniProtKB/TrEMBL;Acc:B6SNB6]	5	13.875	Cleavage	miRNA 20 UCUCGAGGGAAGUUAGGUUU 1<br>. ::.:.:::::::::.: :<br>Target 417 GUAGUUUCCUUCAAUCUACA 436
zma-miR159a-3p	GRMZM2G034804_T08	NA	Nuclear cap-binding protein subunit 2; Uncharacterized protein [Source:UniProtKB/TrEMBL;Acc:B6SNB6]	5	13.875	Cleavage	miRNA 20 UCUCGAGGGAAGUUAGGUUU 1<br>. ::.:.:::::::::.: :<br>Target 348 GUAGUUUCCUUCAAUCUACA 367
zma-miR159a-3p	GRMZM2G037314_T01	NA	NA	5	17.718	Cleavage	miRNA 21 GUCUCGAGGGAAGUUAGGUUU 1<br>:::: :::..::::.:.:..<br>Target 1762 CAGAACUCUUUUCAGUUCGGC 1782
zma-miR159a-3p	GRMZM2G058472_T03	NA	Secondary cell wall glycosyltransferase family 8 isoform 1; Secondary cell wall glycosyltransferase family 8 isoform 2; Uncharacterized protein [Source:UniProtKB/TrEMBL;Acc:C4J490]	5	23.329	Cleavage	miRNA 21 GUCUCGAGGGAAGUUAGGUUU 1<br>:: .:::.:::::..:.:...<br>Target 166 CAAGGCUUCCUUCGGUUCGGG 186
zma-miR159a-3p	GRMZM2G075101_T01	NA	CONTAINS InterPro DOMAIN/s: Plant nuclear matrix 1 (InterPro:IPR010604); Has 30201 Blast hits to 17322 proteins in 780 species: Archae - 12; Bacteria - 1396; Metazoa - 17338; Fungi - 3422; Plants - 5037; Viruses - 0; Other Eukaryotes - 2996 (source: /.../BLink). [Source:Projected from Arabidopsis thaliana (AT5G17620) TAIR;Acc:AT5G17620]	5	8.913	Cleavage	miRNA 20 UCUCGAGGGAAGUUAGGUUU 1<br>: :::::::..:::::::<br>Target 1634 UGUACUCCCUUUGAUCCAAA 1653
zma-miR159a-3p	GRMZM2G075101_T02	NA	CONTAINS InterPro DOMAIN/s: Plant nuclear matrix 1 (InterPro:IPR010604); Has 30201 Blast hits to 17322 proteins in 780 species: Archae - 12; Bacteria - 1396; Metazoa - 17338; Fungi - 3422; Plants - 5037; Viruses - 0; Other Eukaryotes - 2996 (source: /.../BLink). [Source:Projected from Arabidopsis thaliana (AT5G17620) TAIR;Acc:AT5G17620]	5	8.913	Cleavage	miRNA 20 UCUCGAGGGAAGUUAGGUUU 1<br>: :::::::..:::::::<br>Target 1295 UGUACUCCCUUUGAUCCAAA 1314
zma-miR159a-3p	GRMZM2G075101_T03	NA	CONTAINS InterPro DOMAIN/s: Plant nuclear matrix 1 (InterPro:IPR010604); Has 30201 Blast hits to 17322 proteins in 780 species: Archae - 12; Bacteria - 1396; Metazoa - 17338; Fungi - 3422; Plants - 5037; Viruses - 0; Other Eukaryotes - 2996 (source: /.../BLink). [Source:Projected from Arabidopsis thaliana (AT5G17620) TAIR;Acc:AT5G17620]	5	8.913	Cleavage	miRNA 20 UCUCGAGGGAAGUUAGGUUU 1<br>: :::::::..:::::::<br>Target 1454 UGUACUCCCUUUGAUCCAAA 1473
zma-miR159a-3p	GRMZM2G078926_T01	NA	NA	5	11.895	Cleavage	miRNA 21 GUCUCGAGGGAAGUUAGGUUU 1<br>:: ::.:..:::..::.:::<br>Target 2564 CAUAGUUUUCUUUGAUUCAAU 2584
zma-miR159a-3p	GRMZM2G083749_T04	NA	Uncharacterized protein [Source:UniProtKB/TrEMBL;Acc:B4F9D2]	5	18.604	Cleavage	miRNA 21 GUCUCGAGGGAAGUUAGGUUU 1<br>: :::.::.:::::::....<br>Target 566 CUGAGUUCUCUUCAAUUUGGU 586
zma-miR159a-3p	GRMZM2G113137_T01	NA	Cellulose synthase-6 [Source:UniProtKB/TrEMBL;Acc:Q9LLI4]	5	21.745	Cleavage	miRNA 20 UCUCGAGGGAAGUUAGGUUU 1<br>:::.:::::::: :::::<br>Target 975 CCCGCUUCCUUCAAGCCAAA 994

Potential targets of zma-miRNAs.

miRNA	Target	Gene name	Gene description	Expectation	UPE	Inhibition	Hybrid
zma-miR159a-3p	GRMZM2G146267_T01	NA	NA	5	16.107	Cleavage	miRNA 21 GUCUCGAGGGAAGUUAGGUUU 1<br>:::.:.:..:::::::: : :<br>Target 2786 CAGGGUUUUCUUCAAUCGAUA 2806
zma-miR159a-3p	GRMZM2G146267_T02	NA	NA	5	16.107	Cleavage	miRNA 21 GUCUCGAGGGAAGUUAGGUUU 1<br>:::.:.:..:::::::: : :<br>Target 2233 CAGGGUUUUCUUCAAUCGAUA 2253
zma-miR159a-3p	GRMZM2G164705_T01	NA	Uncharacterized protein [Source:UniProtKB/TrEMBL;Acc:K7UV68]	5	19.075	Cleavage	miRNA 21 GUCUCGAGGGAAGUUAGGUUU 1<br>:.:::: :::::::: .: :<br>Target 464 CGGAGCCCCCUUCAAUGUACA 484
zma-miR159a-3p	GRMZM2G314371_T01	NA	Putative uncharacterized protein orf149-a [Source:UniProtKB/TrEMBL;Acc:Q1KKC7]	5	13.311	Cleavage	miRNA 21 GUCUCGAGGGAAGUUAGGUUU 1<br>:::. .:.:.::::::: :.:<br>Target 101 CAGGCUUUCUUUCAAUCAAGA 121
zma-miR159a-3p	GRMZM2G316807_T01	NA	Uncharacterized protein [Source:UniProtKB/TrEMBL;Acc:K7VDU6]	5	21.87	Cleavage	miRNA 21 GUCUCGAGGGAAGUUAGGUUU 1<br>:::.: :::.:::..:.:.:<br>Target 842 CAGGGGUCCUUUCGGUUCGAU 862
zma-miR159a-3p	GRMZM2G321420_T01	NA	Uncharacterized protein [Source:UniProtKB/TrEMBL;Acc:K7VQA8]	5	22.808	Translation	miRNA 21 GUCUCGAGGGAAGUUAGGUUU 1<br>:.:::::.:: :.:::..:.<br>Target 190 CGGAGCUUCCAUUAAUUUAGC 210
zma-miR159a-3p	GRMZM2G433162_T01	NA	Uncharacterized protein [Source:UniProtKB/TrEMBL;Acc:K7UCG6]	5	20.072	Translation	miRNA 20 UCUCGAGGGAAGUUAGGUUU 1<br>::::::::: :.:::::<br>Target 223 CGAGCUCCCUCCGAUCCAUC 242
zma-miR159a-3p	GRMZM2G448776_T01	NA	Uncharacterized protein [Source:UniProtKB/TrEMBL;Acc:K7VP90]	5	9.854	Cleavage	miRNA 20 UCUCGAGGGAAGUUAGGUUU 1<br>:::.:::::::: :::::<br>Target 37 CCCGCUUCCUUCAAGCCAAA 56
zma-miR159a-3p	GRMZM2G479110_T01	NA	Uncharacterized protein [Source:UniProtKB/TrEMBL;Acc:B4FYK6]	5	20.839	Translation	miRNA 21 GUCUCGAGGGAAGUUAGGUUU 1<br>:::: :::.: :::::::: .<br>Target 317 CAGACCUCUCGUCAAUCCAUG 337
zma-miR159a-3p	GRMZM5G832805_T01	NA	Indole-3-acetate beta-glucosyltransferase; Uncharacterized protein [Source:UniProtKB/TrEMBL;Acc:B7ZYZ7]	5	16.892	Cleavage	miRNA 21 GUCUCGAGGGAAGUUAGGUUU 1<br>:::. .:...:::.::::::<br>Target 462 CAGGUUUUUUUUCGAUCCAAU 482
zma-miR159a-3p	GRMZM5G846068_T01	NA	Putative uncharacterized protein orf149-a [Source:UniProtKB/TrEMBL;Acc:Q1KKC7]	5	13.311	Cleavage	miRNA 21 GUCUCGAGGGAAGUUAGGUUU 1<br>:::. .:.:.::::::: :.:<br>Target 101 CAGGCUUUCUUUCAAUCAAGA 121
zma-miR164b-5p	GRMZM2G063522_T01	NA	Uncharacterized protein [Source:UniProtKB/TrEMBL;Acc:C0PB43]	1	19.743	Cleavage	miRNA 20 CGUGCACGGGACGAAGAGGU 1<br>::: ::::::::::::::::<br>Target 896 GCAAGUGCCCUGCUUCUCCA 915
zma-miR164b-5p	GRMZM2G114850_T01	NA	NA	1	20.062	Cleavage	miRNA 20 CGUGCACGGGACGAAGAGGU 1<br>::: ::::::::::::::::<br>Target 1181 GCAGGUGCCCUGCUUCUCCA 1200
zma-miR164b-5p	GRMZM2G139700_T01	NA	Putative uncharacterized protein nam2 [Source:UniProtKB/TrEMBL;Acc:Q5F4H1]	1	20.392	Cleavage	miRNA 20 CGUGCACGGGACGAAGAGGU 1<br>:: :::::::::::::::::<br>Target 738 GCUCGUGCCCUGCUUCUCCA 757
zma-miR164b-5p	GRMZM2G393433_T01	NACTF119	CUC2; Putative NAC domain transcription factor superfamily protein [Source:UniProtKB/TrEMBL;Acc:B6TZM7/MaizeGDB.org]	1	17.089	Cleavage	miRNA 20 CGUGCACGGGACGAAGAGGU 1<br>:: :::::::::::::::::<br>Target 855 GCUCGUGCCCUGCUUCUCCA 874

Potential targets of zma-miRNAs.

miRNA	Target	Gene name	Gene description	Expectation	UPE	Inhibition	Hybrid
zma-miR164b-5p	GRMZM2G393433_T02	NACTF119	CUC2; Putative NAC domain transcription factor superfamily protein [Source:UniProtKB/TrEMBL;Acc:B6TZM7/MaizeGDB.org]	1	17.089	Cleavage	miRNA 20 CGUGCACGGGACGAAGAGGU 1<br>:: :::::::::::::::::<br>Target 815 GCUCGUGCCCUGCUUCUCCA 834
zma-miR164b-5p	GRMZM2G146380_T01	NA	NAC30 NAC type transcription factor [Source:UniProtKB/TrEMBL;Acc:K0DCP5]	2	17.042	Cleavage	miRNA 20 CGUGCACGGGACGAAGAGGU 1<br>:::::: ::::::::::::<br>Target 448 ACACGUGACCUGCUUCUCCA 467
zma-miR164b-5p	GRMZM2G053531_T02	NA	Uncharacterized protein [Source:UniProtKB/TrEMBL;Acc:C0HIG8]	2.5	15.445	Cleavage	miRNA 20 CGUGCACGGGACGAAGAGGU 1<br>:: :::::.::::::::.:.<br>Target 71 GCUCGUGCUCUGCUUCUUCG 90
zma-miR164b-5p	GRMZM2G096358_T01	NA	MYB transcription factor; Putative MYB DNA-binding domain superfamily protein; Uncharacterized protein [Source:UniProtKB/TrEMBL;Acc:B4FU27]	2.5	22.616	Cleavage	miRNA 20 CGUGCACGGGACGAAGAGGU 1<br>:: :::::::::::::::.<br>Target 910 GCCGGUGCCCUGCUUCUCCG 929
zma-miR164b-5p	GRMZM2G121649_T01	NA	Putative uncharacterized protein [Source:UniProtKB/TrEMBL;Acc:B6UHL8]	2.5	21.824	Cleavage	miRNA 21 ACGUGCACGGGACGAAGAGGU 1<br>:::.: :::.::::::::.::<br>Target 531 UGCGCCUGCUCUGCUUCUUCA 551
zma-miR164b-5p	GRMZM2G121649_T02	NA	Putative uncharacterized protein [Source:UniProtKB/TrEMBL;Acc:B6UHL8]	2.5	21.824	Cleavage	miRNA 21 ACGUGCACGGGACGAAGAGGU 1<br>:::.: :::.::::::::.::<br>Target 531 UGCGCCUGCUCUGCUUCUUCA 551
zma-miR164b-5p	GRMZM2G121649_T03	NA	Putative uncharacterized protein [Source:UniProtKB/TrEMBL;Acc:B6UHL8]	2.5	21.824	Cleavage	miRNA 21 ACGUGCACGGGACGAAGAGGU 1<br>:::.: :::.::::::::.::<br>Target 531 UGCGCCUGCUCUGCUUCUUCA 551
zma-miR164b-5p	GRMZM2G173085_T03	NA	Lipase/lipooxygenase	2.5	23.433	Cleavage	miRNA 20 CGUGCACGGGACGAAGAGGU 1<br>:.. :::::.::::::::::<br>Target 356 GUGGGUGCCUUGCUUCUCCA 375
zma-miR164b-5p	GRMZM2G305856_T02	NA	Putative MYB DNA-binding domain superfamily protein [Source:UniProtKB/TrEMBL;Acc:K7UBC4]	2.5	24.772	Cleavage	miRNA 21 ACGUGCACGGGACGAAGAGGU 1<br>::..::::::::::::::.:<br>Target 988 UGUGCGUGCCCUGCUUCUUCU 1008
zma-miR164b-5p	GRMZM2G003769_T01	NA	Peroxisomal biogenesis factor 11 family protein [Source:UniProtKB/TrEMBL;Acc:B6SGC2]	3	15.987	Cleavage	miRNA 20 CGUGCACGGGACGAAGAGGU 1<br>::::: :.:::::::::.:<br>Target 77 GCACGCGUCCUGCUUCUUCU 96
zma-miR164b-5p	GRMZM2G011553_T01	NA	Harpin-induced protein [Source:UniProtKB/TrEMBL;Acc:B6SQ86]	3	19.012	Translation	miRNA 21 ACGUGCACGGGACGAAGAGGU 1<br>:::.: :::::: :::::::.<br>Target 533 UGCGCCUGCCCUCCUUCUCCG 553
zma-miR164b-5p	GRMZM2G022616_T01	NA	Uncharacterized protein [Source:UniProtKB/TrEMBL;Acc:B4FX05]	3	21.425	Cleavage	miRNA 20 CGUGCACGGGACGAAGAGGU 1<br>.:::: :::::.:::::::<br>Target 1430 CUACGUCCCCUGUUUCUCCA 1449
zma-miR164b-5p	GRMZM2G078024_T01	NA	NA	3	21.573	Translation	miRNA 20 CGUGCACGGGACGAAGAGGU 1<br>.::::::.: :::::::::<br>Target 321 CUACGUGCUCAGCUUCUCCA 340
zma-miR164b-5p	GRMZM2G082906_T01	NA	Uncharacterized protein [Source:UniProtKB/TrEMBL;Acc:C4J9W2]	3	15.042	Cleavage	miRNA 20 CGUGCACGGGACGAAGAGGU 1<br>:::: :::.:::::::.::<br>Target 63 CCACGAGCCUUGCUUCUUCA 82
zma-miR164b-5p	GRMZM2G087291_T01	NA	Uncharacterized protein [Source:UniProtKB/TrEMBL;Acc:C0PCW1]	3	11.425	Cleavage	miRNA 20 CGUGCACGGGACGAAGAGGU 1<br>:::: :::.:::::::.::<br>Target 28 CCACGAGCCUUGCUUCUUCA 47

Potential targets of zma-miRNAs.

miRNA	Target	Gene name	Gene description	Expectation	UPE	Inhibition	Hybrid
zma-miR164b-5p	GRMZM2G087291_T02	NA	Uncharacterized protein [Source:UniProtKB/TrEMBL;Acc:C0PCW1]	3	11.425	Cleavage	miRNA 20 CGUGCACGGGACGAAGAGGU 1<br>:::: :::.:::::::.::<br>Target 28 CCACGAGCCUUGCUUCUUCA 47
zma-miR164b-5p	GRMZM2G104419_T01	NA	Uncharacterized protein [Source:UniProtKB/TrEMBL;Acc:K7URL6]	3	19.239	Cleavage	miRNA 20 CGUGCACGGGACGAAGAGGU 1<br>:::.::::..:::: :::::<br>Target 2545 GCAUGUGCUUUGCUCCUCCA 2564
zma-miR164b-5p	GRMZM2G148768_T01	NA	NA	3	15.708	Cleavage	miRNA 20 CGUGCACGGGACGAAGAGGU 1<br>::.: :::.::::::::::<br>Target 207 GCGCCCGCCUUGCUUCUCCA 226
zma-miR164b-5p	GRMZM2G332976_T05	NA	Sex determination protein tasselseed-2 [Source:UniProtKB/TrEMBL;Acc:B6T3V9]	3	15.693	Cleavage	miRNA 20 CGUGCACGGGACGAAGAGGU 1<br>:..::::::::::::::..<br>Target 207 GUGCGUGCCCUGCUUCUUUC 226
zma-miR164b-5p	GRMZM2G422782_T01	NA	Uncharacterized protein [Source:UniProtKB/TrEMBL;Acc:B4FPX3]	3	15.708	Cleavage	miRNA 20 CGUGCACGGGACGAAGAGGU 1<br>::.: :::.::::::::::<br>Target 461 GCGCCCGCCUUGCUUCUCCA 480
zma-miR164b-5p	GRMZM2G004694_T01	NA	NA	3.5	22.882	Translation	miRNA 20 CGUGCACGGGACGAAGAGGU 1<br>.::::: ::: ::::::::<br>Target 1329 CUACGUGGCCUUCUUCUCCA 1348
zma-miR164b-5p	GRMZM2G006468_T01	NA	Putative wound responsive protein; Putative wound responsive protein isoform 1; Putative wound responsive protein isoform 2; Wound responsive protein [Source:UniProtKB/TrEMBL;Acc:B6SUR2]	3.5	18.45	Cleavage	miRNA 20 CGUGCACGGGACGAAGAGGU 1<br>: :::::.::::::::.:.<br>Target 158 CCUCGUGCUCUGCUUCUUCG 177
zma-miR164b-5p	GRMZM2G006468_T02	NA	Putative wound responsive protein; Putative wound responsive protein isoform 1; Putative wound responsive protein isoform 2; Wound responsive protein [Source:UniProtKB/TrEMBL;Acc:B6SUR2]	3.5	17.855	Cleavage	miRNA 20 CGUGCACGGGACGAAGAGGU 1<br>: :::::.::::::::.:.<br>Target 158 CCUCGUGCUCUGCUUCUUCG 177
zma-miR164b-5p	GRMZM2G006468_T03	NA	Putative wound responsive protein; Putative wound responsive protein isoform 1; Putative wound responsive protein isoform 2; Wound responsive protein [Source:UniProtKB/TrEMBL;Acc:B6SUR2]	3.5	17.855	Cleavage	miRNA 20 CGUGCACGGGACGAAGAGGU 1<br>: :::::.::::::::.:.<br>Target 158 CCUCGUGCUCUGCUUCUUCG 177
zma-miR164b-5p	GRMZM2G006468_T04	NA	Putative wound responsive protein; Putative wound responsive protein isoform 1; Putative wound responsive protein isoform 2; Wound responsive protein [Source:UniProtKB/TrEMBL;Acc:B6SUR2]	3.5	17.796	Cleavage	miRNA 20 CGUGCACGGGACGAAGAGGU 1<br>: :::::.::::::::.:.<br>Target 158 CCUCGUGCUCUGCUUCUUCG 177
zma-miR164b-5p	GRMZM2G006468_T05	NA	Putative wound responsive protein; Putative wound responsive protein isoform 1; Putative wound responsive protein isoform 2; Wound responsive protein [Source:UniProtKB/TrEMBL;Acc:B6SUR2]	3.5	18.45	Cleavage	miRNA 20 CGUGCACGGGACGAAGAGGU 1<br>: :::::.::::::::.:.<br>Target 158 CCUCGUGCUCUGCUUCUUCG 177
zma-miR164b-5p	GRMZM2G010740_T01	NA	Uncharacterized protein [Source:UniProtKB/TrEMBL;Acc:K7VBD3]	3.5	24.16	Translation	miRNA 20 CGUGCACGGGACGAAGAGGU 1<br>::::: .:: :::::::::<br>Target 1078 CCACGUCUCCAGCUUCUCCA 1097
zma-miR164b-5p	GRMZM2G011156_T01	NA	NA	3.5	18.491	Cleavage	miRNA 20 CGUGCACGGGACGAAGAGGU 1<br>.:::::. ::::::::::.<br>Target 750 CUACGUGUACUGCUUCUCCG 769
zma-miR164b-5p	GRMZM2G032351_T01	NA	Phototropin-1 [Source:UniProtKB/TrEMBL;Acc:B6SS20]	3.5	9.223	Cleavage	miRNA 21 ACGUGCACGGGACGAAGAGGU 1<br>::..: ::::::::::::.:<br>Target 21 UGUGCCUGCCCUGCUUCUUCC 41
zma-miR164b-5p	GRMZM2G032351_T02	NA	Phototropin-1 [Source:UniProtKB/TrEMBL;Acc:B6SS20]	3.5	9.223	Cleavage	miRNA 21 ACGUGCACGGGACGAAGAGGU 1<br>::..: ::::::::::::.:<br>Target 21 UGUGCCUGCCCUGCUUCUUCC 41

miRNA	Target	Gene name	Gene description	Expectation	UPE	Inhibition	Hybrid
zma-miR164b-5p	GRMZM2G037152_T01	NA	NA	3.5	22.974	Translation	miRNA 20 CGUGCACGGGACGAAGAGGU 1<br>:::::::..:: : ::::::<br>Target 474 GCACGUGUUCUACCUCUCCA 493
zma-miR164b-5p	GRMZM2G043240_T01	NA	Putative uncharacterized protein [Source:UniProtKB/TrEMBL;Acc:B6U1A6]	3.5	4.755	Translation	miRNA 20 CGUGCACGGGACGAAGAGGU 1<br>::::::::: :::::::.<br>Target 271 ACACGUGCCCGCCUUCUCCG 290
zma-miR164b-5p	GRMZM2G043240_T02	NA	Putative uncharacterized protein [Source:UniProtKB/TrEMBL;Acc:B6U1A6]	3.5	4.755	Translation	miRNA 20 CGUGCACGGGACGAAGAGGU 1<br>::::::::: :::::::.<br>Target 271 ACACGUGCCCGCCUUCUCCG 290
zma-miR164b-5p	GRMZM2G043240_T03	NA	Putative uncharacterized protein [Source:UniProtKB/TrEMBL;Acc:B6U1A6]	3.5	4.755	Translation	miRNA 20 CGUGCACGGGACGAAGAGGU 1<br>::::::::: :::::::.<br>Target 271 ACACGUGCCCGCCUUCUCCG 290
zma-miR164b-5p	GRMZM2G043240_T04	NA	Putative uncharacterized protein [Source:UniProtKB/TrEMBL;Acc:B6U1A6]	3.5	4.755	Translation	miRNA 20 CGUGCACGGGACGAAGAGGU 1<br>::::::::: :::::::.<br>Target 271 ACACGUGCCCGCCUUCUCCG 290
zma-miR164b-5p	GRMZM2G057930_T01	NA	Transferase	3.5	21.569	Cleavage	miRNA 20 CGUGCACGGGACGAAGAGGU 1<br>::.::: ::::::: ::.::<br>Target 819 GCGCGUCCCCUGCUACUUCA 838
zma-miR164b-5p	GRMZM2G060579_T01	NA	Uncharacterized protein [Source:UniProtKB/TrEMBL;Acc:C0PC88]	3.5	17.319	Translation	miRNA 21 ACGUGCACGGGACGAAGAGGU 1<br>: .::::::::: :::::::<br>Target 654 UCUACGUGCCCUUCUUCUCCU 674
zma-miR164b-5p	GRMZM2G086880_T01	NA	Uncharacterized protein [Source:UniProtKB/TrEMBL;Acc:K7V3M5]	3.5	22.512	Cleavage	miRNA 21 ACGUGCACGGGACGAAGAGGU 1<br>::: : : ::::::::::.::<br>Target 413 UGCUCCUCCCCUGCUUCUUCA 433
zma-miR164b-5p	GRMZM2G089525_T01	NA	NA	3.5	23.045	Cleavage	miRNA 21 ACGUGCACGGGACGAAGAGGU 1<br>::: : : ::::::::::.::<br>Target 403 UGCUCCUCCCCUGCUUCUUCA 423
zma-miR164b-5p	GRMZM2G104489_T01	NA	Uncharacterized protein [Source:UniProtKB/TrEMBL;Acc:K7TPI4]	3.5	18.34	Cleavage	miRNA 21 ACGUGCACGGGACGAAGAGGU 1<br>::: : ::.::::.:::::.:<br>Target 184 UGCCCCUGUCCUGUUUCUCUA 204
zma-miR164b-5p	GRMZM2G121208_T01	NA	Uncharacterized protein [Source:UniProtKB/TrEMBL;Acc:C0PPI1]	3.5	24.909	Translation	miRNA 20 CGUGCACGGGACGAAGAGGU 1<br>:..:::::: ::::::.::<br>Target 941 ACGUGUGCCCAGCUUCUUCA 960
zma-miR164b-5p	GRMZM2G125424_T01	NA	Uncharacterized protein [Source:UniProtKB/TrEMBL;Acc:C0PGU4]	3.5	21.079	Translation	miRNA 20 CGUGCACGGGACGAAGAGGU 1<br>::::::.:: ::::::::<br>Target 317 CCACGUGUCCACCUUCUCCA 336
zma-miR164b-5p	GRMZM2G125424_T02	NA	Uncharacterized protein [Source:UniProtKB/TrEMBL;Acc:C0PGU4]	3.5	21.079	Translation	miRNA 20 CGUGCACGGGACGAAGAGGU 1<br>::::::.:: ::::::::<br>Target 405 CCACGUGUCCACCUUCUCCA 424
zma-miR164b-5p	GRMZM2G125424_T03	NA	Uncharacterized protein [Source:UniProtKB/TrEMBL;Acc:C0PGU4]	3.5	21.079	Translation	miRNA 20 CGUGCACGGGACGAAGAGGU 1<br>::::::.:: ::::::::<br>Target 2279 CCACGUGUCCACCUUCUCCA 2298
zma-miR164b-5p	GRMZM2G125424_T04	NA	Uncharacterized protein [Source:UniProtKB/TrEMBL;Acc:C0PGU4]	3.5	21.079	Translation	miRNA 20 CGUGCACGGGACGAAGAGGU 1<br>::::::.:: ::::::::<br>Target 3088 CCACGUGUCCACCUUCUCCA 3107

Potential targets of zma-miRNAs.

miRNA	Target	Gene name	Gene description	Expectation	UPE	Inhibition	Hybrid
zma-miR164b-5p	GRMZM2G128445_T01	NA	NA	3.5	21.604	Translation	miRNA 20 CGUGCACGGGACGAAGAGGU 1<br>:::::::..:: : ::::::<br>Target 396 GCACGUGUUCUACCUCUCCA 415
zma-miR164b-5p	GRMZM2G131351_T01	NA	NA	3.5	19.275	Translation	miRNA 20 CGUGCACGGGACGAAGAGGU 1<br>:::::::..:: : ::::::<br>Target 276 GCACGUGUUCUACCUCUCCA 295
zma-miR164b-5p	GRMZM2G133558_T01	NA	Uncharacterized protein [Source:UniProtKB/TrEMBL;Acc:K7UL84]	3.5	23.584	Translation	miRNA 21 ACGUGCACGGGACGAAGAGGU 1<br>::: : :::.: :::::::::<br>Target 378 UGCCCUUGCUCGGCUUCUCCA 398
zma-miR164b-5p	GRMZM2G133558_T02	NA	Uncharacterized protein [Source:UniProtKB/TrEMBL;Acc:K7UL84]	3.5	23.584	Translation	miRNA 21 ACGUGCACGGGACGAAGAGGU 1<br>::: : :::.: :::::::::<br>Target 378 UGCCCUUGCUCGGCUUCUCCA 398
zma-miR164b-5p	GRMZM2G133558_T03	NA	Uncharacterized protein [Source:UniProtKB/TrEMBL;Acc:K7UL84]	3.5	23.584	Translation	miRNA 21 ACGUGCACGGGACGAAGAGGU 1<br>::: : :::.: :::::::::<br>Target 378 UGCCCUUGCUCGGCUUCUCCA 398
zma-miR164b-5p	GRMZM2G168681_T01	FPS	Farnesyl pyrophosphate synthase [Source:UniProtKB/Swiss-Prot;Acc:P49353]	3.5	20.253	Cleavage	miRNA 21 ACGUGCACGGGACGAAGAGGU 1<br>::::.:::::::::: :::.<br>Target 874 UGCAUGUGCCCUGCUGCUCUC 894
zma-miR164b-5p	GRMZM2G168681_T02	FPS	Farnesyl pyrophosphate synthase [Source:UniProtKB/Swiss-Prot;Acc:P49353]	3.5	20.253	Cleavage	miRNA 21 ACGUGCACGGGACGAAGAGGU 1<br>::::.:::::::::: :::.<br>Target 544 UGCAUGUGCCCUGCUGCUCUC 564
zma-miR164b-5p	GRMZM2G176117_T01	NA	Uncharacterized protein [Source:UniProtKB/TrEMBL;Acc:B4FMG4]	3.5	9.672	Cleavage	miRNA 20 CGUGCACGGGACGAAGAGGU 1<br>.:: : ::::::::::::<br>Target 41 UUACCUUCCCUGCUUCUCCA 60
zma-miR164b-5p	GRMZM2G178289_T03	NA	Uncharacterized protein [Source:UniProtKB/TrEMBL;Acc:B6UG90]	3.5	20.61	Translation	miRNA 20 CGUGCACGGGACGAAGAGGU 1<br>:.:::::.: ::::::::.<br>Target 1006 CCGCGUGCUCGGCUUCUCCG 1025
zma-miR164b-5p	GRMZM2G330379_T01	NA	NA	3.5	23.361	Cleavage	miRNA 20 CGUGCACGGGACGAAGAGGU 1<br>::.::: :.:::: ::::::<br>Target 641 GCGCGUCCUCUGCGUCUCCA 660
zma-miR164b-5p	GRMZM2G471904_T01	NA	ATP binding microtubule motor family protein [Source:Projected from Arabidopsis thaliana (AT2G37420) TAIR;Acc:AT2G37420]	3.5	17.449	Cleavage	miRNA 21 ACGUGCACGGGACGAAGAGGU 1<br>:: ::::::::::: ::.:.<br>Target 4992 UGGACGUGCCCUGCUCCUUCG 5012
zma-miR164b-5p	GRMZM2G471904_T02	NA	ATP binding microtubule motor family protein [Source:Projected from Arabidopsis thaliana (AT2G37420) TAIR;Acc:AT2G37420]	3.5	17.449	Cleavage	miRNA 21 ACGUGCACGGGACGAAGAGGU 1<br>:: ::::::::::: ::.:.<br>Target 5007 UGGACGUGCCCUGCUCCUUCG 5027
zma-miR164b-5p	GRMZM2G587231_T01	NA	Uncharacterized protein [Source:UniProtKB/TrEMBL;Acc:C0PDV6]	3.5	20.052	Cleavage	miRNA 20 CGUGCACGGGACGAAGAGGU 1<br>::.:: :::::::::::<br>Target 132 GCGCGCCCCCUGCUUCUCCU 151
zma-miR164b-5p	GRMZM5G852968_T01	NA	Triosephosphate isomerase [Source:UniProtKB/TrEMBL;Acc:B4FCE2]	3.5	24.777	Cleavage	miRNA 20 CGUGCACGGGACGAAGAGGU 1<br>:..: ::..::::::::.::<br>Target 337 GUGCAUGUUCUGCUUCUUCA 356
zma-miR164b-5p	AC187065.3_FGT003	NA	Uncharacterized protein [Source:UniProtKB/TrEMBL;Acc:K7UVT9]	4	23.526	Cleavage	miRNA 21 ACGUGCACGGGACGAAGAGGU 1<br>::: :: :::::::::.::.<br>Target 24 UGCUCGCGCCCUGCUUUUCUC 44

Potential targets of zma-miRNAs.

miRNA	Target	Gene name	Gene description	Expectation	UPE	Inhibition	Hybrid
zma-miR164b-5p	AC204383.2_FGT002	NA	Uncharacterized protein [Source:UniProtKB/TrEMBL;Acc:K7U1M2]	4	20.369	Translation	miRNA 21 ACGUGCACGGGACGAAGAGGU 1<br>:::::::::. : :::: :::<br>Target 168 UGCACGUGCUGUCCUUCACCA 188
zma-miR164b-5p	GRMZM2G003426_T01	NA	Uncharacterized protein [Source:UniProtKB/TrEMBL;Acc:B4FEQ1]	4	11.857	Cleavage	miRNA 21 ACGUGCACGGGACGAAGAGGU 1<br>:::.: ::.::::. ::::::<br>Target 164 UGCGCCUGUCCUGUGUCUCCA 184
zma-miR164b-5p	GRMZM2G013200_T01	NA	Polyphosphoinositide binding protein Ssh2p [Source:UniProtKB/TrEMBL;Acc:B6TDP3]	4	19.512	Translation	miRNA 21 ACGUGCACGGGACGAAGAGGU 1<br>: :::::::::: : :::.::<br>Target 686 UCCACGUGCCCUACGUCUUCA 706
zma-miR164b-5p	GRMZM2G013200_T02	NA	Polyphosphoinositide binding protein Ssh2p [Source:UniProtKB/TrEMBL;Acc:B6TDP3]	4	19.512	Translation	miRNA 21 ACGUGCACGGGACGAAGAGGU 1<br>: :::::::::: : :::.::<br>Target 694 UCCACGUGCCCUACGUCUUCA 714
zma-miR164b-5p	GRMZM2G013200_T03	NA	Polyphosphoinositide binding protein Ssh2p [Source:UniProtKB/TrEMBL;Acc:B6TDP3]	4	19.512	Translation	miRNA 21 ACGUGCACGGGACGAAGAGGU 1<br>: :::::::::: : :::.::<br>Target 678 UCCACGUGCCCUACGUCUUCA 698
zma-miR164b-5p	GRMZM2G013630_T01	NA	Polyphosphoinositide binding protein Ssh2p [Source:UniProtKB/TrEMBL;Acc:B6TDP3]	4	19.512	Translation	miRNA 21 ACGUGCACGGGACGAAGAGGU 1<br>: :::::::::: : :::.::<br>Target 686 UCCACGUGCCCUACGUCUUCA 706
zma-miR164b-5p	GRMZM2G013630_T02	NA	Polyphosphoinositide binding protein Ssh2p [Source:UniProtKB/TrEMBL;Acc:B6TDP3]	4	19.512	Translation	miRNA 21 ACGUGCACGGGACGAAGAGGU 1<br>: :::::::::: : :::.::<br>Target 694 UCCACGUGCCCUACGUCUUCA 714
zma-miR164b-5p	GRMZM2G013630_T03	NA	Polyphosphoinositide binding protein Ssh2p [Source:UniProtKB/TrEMBL;Acc:B6TDP3]	4	19.512	Translation	miRNA 21 ACGUGCACGGGACGAAGAGGU 1<br>: :::::::::: : :::.::<br>Target 678 UCCACGUGCCCUACGUCUUCA 698
zma-miR164b-5p	GRMZM2G019501_T01	NA	Uncharacterized protein [Source:UniProtKB/TrEMBL;Acc:B4FHW0]	4	23.291	Cleavage	miRNA 21 ACGUGCACGGGACGAAGAGGU 1<br>:::.: : .:.:::::::::.<br>Target 105 UGCGCCUAUCUUGCUUCUCCG 125
zma-miR164b-5p	GRMZM2G019501_T02	NA	Uncharacterized protein [Source:UniProtKB/TrEMBL;Acc:B4FHW0]	4	23.497	Cleavage	miRNA 21 ACGUGCACGGGACGAAGAGGU 1<br>:::.: : .:.:::::::::.<br>Target 81 UGCGCCUAUCUUGCUUCUCCG 101
zma-miR164b-5p	GRMZM2G024104_T01	GLN2	Glutamine synthetase root isozyme 2 [Source:UniProtKB/Swiss-Prot;Acc:P38560]	4	24.997	Cleavage	miRNA 20 CGUGCACGGGACGAAGAGGU 1<br>:..:::::.:::: :.:.::<br>Target 67 GUGCGUGCUCUGCCUUUUCA 86
zma-miR164b-5p	GRMZM2G024104_T02	GLN2	Glutamine synthetase root isozyme 2 [Source:UniProtKB/Swiss-Prot;Acc:P38560]	4	24.997	Cleavage	miRNA 20 CGUGCACGGGACGAAGAGGU 1<br>:..:::::.:::: :.:.::<br>Target 67 GUGCGUGCUCUGCCUUUUCA 86
zma-miR164b-5p	GRMZM2G032163_T01	NA	nudix hydrolase homolog 3 [Source:Projected from Arabidopsis thaliana (AT1G79690) TAIR;Acc:AT1G79690]	4	24.828	Cleavage	miRNA 20 CGUGCACGGGACGAAGAGGU 1<br>.:::::.::: :::::::<br>Target 1317 CAGCGUGCUCUGAUUCUCCA 1336
zma-miR164b-5p	GRMZM2G037644_T01	NA	Uncharacterized protein [Source:UniProtKB/TrEMBL;Acc:C0P8Y6]	4	18.763	Cleavage	miRNA 21 ACGUGCACGGGACGAAGAGGU 1<br>::.:::: :::::.: :::.:<br>Target 1692 UGUACGUACCCUGUUGCUCUA 1712
zma-miR164b-5p	GRMZM2G051005_T01	NA	Uncharacterized protein [Source:UniProtKB/TrEMBL;Acc:C0PC30]	4	20.377	Cleavage	miRNA 21 ACGUGCACGGGACGAAGAGGU 1<br>:::::::: .::: ::::::<br>Target 143 UGCACGUGGUCUGGCUCUCCA 163

Potential targets of zma-miRNAs.

miRNA	Target	Gene name	Gene description	Expectation	UPE	Inhibition	Hybrid
zma-miR164b-5p	GRMZM2G056075_T01	MUS1	DNA mismatch repair protein MSH2 [Source:UniProtKB/Swiss-Prot;Acc:Q9XGC9]	4	21.785	Translation	miRNA 21 ACGUGCACGGGACGAAGAGGU 1<br>:::::: ::.: ::::.:::<br>Target 2885 UGCACGGGCUCGGCUUUUCCU 2905
zma-miR164b-5p	GRMZM2G059580_T03	NA	Elongation factor 1-gamma 3 [Source:UniProtKB/TrEMBL;Acc:B6T7G7]	4	18.807	Translation	miRNA 21 ACGUGCACGGGACGAAGAGGU 1<br>::::.:::: .: .:::::.:<br>Target 25 UGCAUGUGCAUUUUUUCUCUA 45
zma-miR164b-5p	GRMZM2G065989_T01	NA	NA	4	19.464	Cleavage	miRNA 21 ACGUGCACGGGACGAAGAGGU 1<br>::..: ::::.:: ::.::::<br>Target 1089 UGUGCUUGCCUUGGUUUUCCA 1109
zma-miR164b-5p	GRMZM2G065989_T02	NA	NA	4	19.464	Cleavage	miRNA 21 ACGUGCACGGGACGAAGAGGU 1<br>::..: ::::.:: ::.::::<br>Target 1148 UGUGCUUGCCUUGGUUUUCCA 1168
zma-miR164b-5p	GRMZM2G075100_T01	NA	Putative lung seven transmembrane receptor family protein [Source:UniProtKB/TrEMBL;Acc:K7TQN5]	4	20.209	Translation	miRNA 21 ACGUGCACGGGACGAAGAGGU 1<br>: .:::::..: ::::::.::<br>Target 815 UCUACGUGUUCGGCUUCUUCA 835
zma-miR164b-5p	GRMZM2G084440_T01	NA	GDP dissociation inhibitor family protein / Rab GTPase activator family protein [Source:Projected from Arabidopsis thaliana (AT5G09550) TAIR;Acc:AT5G09550]	4	16.252	Cleavage	miRNA 21 ACGUGCACGGGACGAAGAGGU 1<br>::.:::::..::::: ::::<br>Target 1097 UGUACGUGUUCUGCUGCUCCU 1117
zma-miR164b-5p	GRMZM2G087186_T01	NA	Pyruvate decarboxylase isozyme 3 [Source:UniProtKB/Swiss-Prot;Acc:Q05327]	4	11.581	Cleavage	miRNA 20 CGUGCACGGGACGAAGAGGU 1<br>:..:: :::::::::::<br>Target 2125 ACGUGUUCCCUGCUUCUCCC 2144
zma-miR164b-5p	GRMZM2G087186_T02	NA	Pyruvate decarboxylase isozyme 3 [Source:UniProtKB/Swiss-Prot;Acc:Q05327]	4	11.581	Cleavage	miRNA 20 CGUGCACGGGACGAAGAGGU 1<br>:..:: :::::::::::<br>Target 2122 ACGUGUUCCCUGCUUCUCCC 2141
zma-miR164b-5p	GRMZM2G087186_T03	NA	Pyruvate decarboxylase isozyme 3 [Source:UniProtKB/Swiss-Prot;Acc:Q05327]	4	11.581	Cleavage	miRNA 20 CGUGCACGGGACGAAGAGGU 1<br>:..:: :::::::::::<br>Target 2106 ACGUGUUCCCUGCUUCUCCC 2125
zma-miR164b-5p	GRMZM2G087186_T04	NA	Pyruvate decarboxylase isozyme 3 [Source:UniProtKB/Swiss-Prot;Acc:Q05327]	4	11.581	Cleavage	miRNA 20 CGUGCACGGGACGAAGAGGU 1<br>:..:: :::::::::::<br>Target 2215 ACGUGUUCCCUGCUUCUCCC 2234
zma-miR164b-5p	GRMZM2G089952_T01	NA	Uncharacterized protein [Source:UniProtKB/TrEMBL;Acc:C0P603]	4	21.35	Translation	miRNA 21 ACGUGCACGGGACGAAGAGGU 1<br>: ::::::::: : :::.::<br>Target 642 UCCACGUGCCCUACAUCUUCA 662
zma-miR164b-5p	GRMZM2G094563_T01	NA	Uncharacterized protein [Source:UniProtKB/TrEMBL;Acc:B4FYY6]	4	24.482	Translation	miRNA 21 ACGUGCACGGGACGAAGAGGU 1<br>: .:::::..: ::::::.::<br>Target 1011 UCUACGUGUUCGGCUUCUUCA 1031
zma-miR164b-5p	GRMZM2G098011_T01	NA	Uncharacterized protein [Source:UniProtKB/TrEMBL;Acc:B8A2T1]	4	13.878	Cleavage	miRNA 21 ACGUGCACGGGACGAAGAGGU 1<br>::. : ::::::::::::.:<br>Target 1573 UGUUCCUGCCCUGCUUCUUCC 1593
zma-miR164b-5p	GRMZM2G098011_T02	NA	Uncharacterized protein [Source:UniProtKB/TrEMBL;Acc:B8A2T1]	4	13.878	Cleavage	miRNA 21 ACGUGCACGGGACGAAGAGGU 1<br>::. : ::::::::::::.:<br>Target 1558 UGUUCCUGCCCUGCUUCUUCC 1578
zma-miR164b-5p	GRMZM2G114162_T01	NA	NA	4	17.435	Translation	miRNA 20 CGUGCACGGGACGAAGAGGU 1<br>:.::::::.: :::::.:.<br>Target 88 UCGCGUGCCUUUCUUCUUCG 107

miRNA	Target	Gene name	Gene description	Expectation	UPE	Inhibition	Hybrid
zma-miR164b-5p	GRMZM2G124566_T01	NA	Growth-regulating factor [Source:UniProtKB/TrEMBL;Acc:B6UGM8]	4	22.209	Translation	miRNA 20 CGUGCACGGGACGAAGAGGU 1<br>::::::::.: :: :::::<br>Target 740 GCACGUGCUCGGCCUCUCCU 759
zma-miR164b-5p	GRMZM2G124566_T02	NA	Growth-regulating factor [Source:UniProtKB/TrEMBL;Acc:B6UGM8]	4	22.209	Translation	miRNA 20 CGUGCACGGGACGAAGAGGU 1<br>::::::::.: :: :::::<br>Target 716 GCACGUGCUCGGCCUCUCCU 735
zma-miR164b-5p	GRMZM2G136367_T03	NA	Uncharacterized protein [Source:UniProtKB/TrEMBL;Acc:B4FAR6]	4	17.765	Cleavage	miRNA 20 CGUGCACGGGACGAAGAGGU 1<br>::.:: ::::::::::.:<br>Target 756 UCAUGUCCCCUGCUUCUUCC 775
zma-miR164b-5p	GRMZM2G136367_T04	NA	Uncharacterized protein [Source:UniProtKB/TrEMBL;Acc:B4FAR6]	4	17.765	Cleavage	miRNA 20 CGUGCACGGGACGAAGAGGU 1<br>::.:: ::::::::::.:<br>Target 1025 UCAUGUCCCCUGCUUCUUCC 1044
zma-miR164b-5p	GRMZM2G136367_T05	NA	Uncharacterized protein [Source:UniProtKB/TrEMBL;Acc:B4FAR6]	4	17.765	Cleavage	miRNA 20 CGUGCACGGGACGAAGAGGU 1<br>::.:: ::::::::::.:<br>Target 976 UCAUGUCCCCUGCUUCUUCC 995
zma-miR164b-5p	GRMZM2G136367_T06	NA	Uncharacterized protein [Source:UniProtKB/TrEMBL;Acc:B4FAR6]	4	17.765	Cleavage	miRNA 20 CGUGCACGGGACGAAGAGGU 1<br>::.:: ::::::::::.:<br>Target 966 UCAUGUCCCCUGCUUCUUCC 985
zma-miR164b-5p	GRMZM2G162336_T01	NA	Uncharacterized protein [Source:UniProtKB/TrEMBL;Acc:K7UL75]	4	20.183	Cleavage	miRNA 20 CGUGCACGGGACGAAGAGGU 1<br>::.::: ::::::: ::::<br>Target 189 GCGCGUCCCCUGCUCCUCCC 208
zma-miR164b-5p	GRMZM2G172065_T01	NA	Uncharacterized protein [Source:UniProtKB/TrEMBL;Acc:B7ZXJ5]	4	19.974	Translation	miRNA 21 ACGUGCACGGGACGAAGAGGU 1<br>::.:.:::.:. ::::::::<br>Target 1610 UGUAUGUGUCUAGCUUCUCCU 1630
zma-miR164b-5p	GRMZM2G173289_T01	NA	Protein GPR107 [Source:UniProtKB/TrEMBL;Acc:B6UCS7]	4	24.372	Translation	miRNA 21 ACGUGCACGGGACGAAGAGGU 1<br>: .:::::..: ::::::.::<br>Target 842 UCUACGUGUUCGGCUUCUUCA 862
zma-miR164b-5p	GRMZM2G365515_T01	NA	Mitochondrial carrier-like protein [Source:UniProtKB/TrEMBL;Acc:B6STR5]	4	16.925	Cleavage	miRNA 21 ACGUGCACGGGACGAAGAGGU 1<br>::::. :::..::.::::..:<br>Target 1452 UGCAUUUGCUUUGUUUCUUUA 1472
zma-miR164b-5p	GRMZM2G397661_T01	NA	Thioesterase family protein [Source:UniProtKB/TrEMBL;Acc:K7TQ73]	4	21.909	Cleavage	miRNA 20 CGUGCACGGGACGAAGAGGU 1<br>:..::::.::::::::: :<br>Target 602 UCGUGUGCUCUGCUUCUCGA 621
zma-miR164b-5p	GRMZM2G402977_T01	NA	Putative uncharacterized protein [Source:UniProtKB/TrEMBL;Acc:B6U425]	4	19.285	Cleavage	miRNA 20 CGUGCACGGGACGAAGAGGU 1<br>: .:::::::::: :::::<br>Target 641 CCCUGUGCCCUGCUCCUCCA 660
zma-miR164b-5p	GRMZM2G439337_T01	NA	Uncharacterized protein [Source:UniProtKB/TrEMBL;Acc:K7V9B2]	4	13.057	Cleavage	miRNA 20 CGUGCACGGGACGAAGAGGU 1<br>::::: :::::::::: .:<br>Target 297 GCACGCGCCCUGCUUCCUCC 316
zma-miR164b-5p	GRMZM2G477340_T03	NA	Uncharacterized protein [Source:UniProtKB/TrEMBL;Acc:B4F9I3]	4	14.752	Cleavage	miRNA 20 CGUGCACGGGACGAAGAGGU 1<br>:::: :::.:::::::::<br>Target 1125 GCACAUGCUCUGCUUCUCAU 1144
zma-miR164b-5p	GRMZM2G479744_T01	NA	defective in exine formation protein (DEX1) [Source:Projected from Arabidopsis thaliana (AT3G09090) TAIR;Acc:AT3G09090]	4	12.784	Cleavage	miRNA 20 CGUGCACGGGACGAAGAGGU 1<br>:::: ..:::::::::::<br>Target 1442 CAACGUCUUCUGCUUCUCCA 1461

Potential targets of zma-miRNAs.

miRNA	Target	Gene name	Gene description	Expectation	UPE	Inhibition	Hybrid
zma-miR164b-5p	GRMZM2G704151_T01	NA	Uncharacterized protein [Source:UniProtKB/TrEMBL;Acc:K7VGG9]	4	13.855	Translation	miRNA 20 CGUGCACGGGACGAAGAGGU 1<br>:.::::::::: ::::::<br>Target 7 GUACGUGCCCUCCUUCUCGC 26
zma-miR164b-5p	GRMZM5G840145_T01	NA	Uncharacterized protein [Source:UniProtKB/TrEMBL;Acc:B4FL98]	4	12.095	Cleavage	miRNA 20 CGUGCACGGGACGAAGAGGU 1<br>::.::::..:::. :::::.<br>Target 28 GCGCGUGUUCUGUCUCUCCG 47
zma-miR164b-5p	GRMZM5G840145_T02	NA	Uncharacterized protein [Source:UniProtKB/TrEMBL;Acc:B4FL98]	4	12.095	Cleavage	miRNA 20 CGUGCACGGGACGAAGAGGU 1<br>::.::::..:::. :::::.<br>Target 28 GCGCGUGUUCUGUCUCUCCG 47
zma-miR164b-5p	GRMZM5G843302_T01	NA	Putative glycerol 3-phosphate permease; Uncharacterized protein [Source:UniProtKB/TrEMBL;Acc:B8A0W6]	4	22.641	Translation	miRNA 21 ACGUGCACGGGACGAAGAGGU 1<br>::: ::::.::: :: :::::<br>Target 1880 UGCCCGUGUCCUCCUCCUCCA 1900
zma-miR164b-5p	GRMZM5G843302_T02	NA	Putative glycerol 3-phosphate permease; Uncharacterized protein [Source:UniProtKB/TrEMBL;Acc:B8A0W6]	4	22.641	Translation	miRNA 21 ACGUGCACGGGACGAAGAGGU 1<br>::: ::::.::: :: :::::<br>Target 1786 UGCCCGUGUCCUCCUCCUCCA 1806
zma-miR164b-5p	GRMZM2G000764_T01	NA	Uncharacterized protein [Source:UniProtKB/TrEMBL;Acc:K7UA13]	4.5	21.725	Cleavage	miRNA 20 CGUGCACGGGACGAAGAGGU 1<br>.:::::::::::: :: ::<br>Target 396 CUACGUGCCCUGCUACUACA 415
zma-miR164b-5p	GRMZM2G014397_T01	NA	Uncharacterized protein [Source:UniProtKB/TrEMBL;Acc:B4FG49]	4.5	15.833	Cleavage	miRNA 20 CGUGCACGGGACGAAGAGGU 1<br>.:::::::::.::.:..:<br>Target 1280 UAGCGUGCCCUGUUUUUUUA 1299
zma-miR164b-5p	GRMZM2G014397_T02	NA	Uncharacterized protein [Source:UniProtKB/TrEMBL;Acc:B4FG49]	4.5	15.833	Cleavage	miRNA 20 CGUGCACGGGACGAAGAGGU 1<br>.:::::::::.::.:..:<br>Target 1201 UAGCGUGCCCUGUUUUUUUA 1220
zma-miR164b-5p	GRMZM2G017269_T01	NA	HEAT repeat family; HEAT repeat family protein; Uncharacterized protein [Source:UniProtKB/TrEMBL;Acc:B4FX70]	4.5	15.323	Cleavage	miRNA 20 CGUGCACGGGACGAAGAGGU 1<br>::..::::.:::: ::::.<br>Target 232 GCGUGUGCUCUGCCUCUCUC 251
zma-miR164b-5p	GRMZM2G094589_T01	NA	NA	4.5	13.047	Cleavage	miRNA 21 ACGUGCACGGGACGAAGAGGU 1<br>::: .::::..:::::.:...<br>Target 961 UGCUUGUGCUUUGCUUUUUUG 981
zma-miR164b-5p	GRMZM2G104176_T01	NA	Uncharacterized protein [Source:UniProtKB/TrEMBL;Acc:B4G221]	4.5	21.22	Cleavage	miRNA 20 CGUGCACGGGACGAAGAGGU 1<br>::::::.::::::: .::<br>Target 1659 AAACGUGCUCUGCUUCGUCA 1678
zma-miR164b-5p	GRMZM2G124321_T01	NA	uvrB/uvrC motif-containing protein [Source:Projected from Arabidopsis thaliana (AT2G03390) TAIR;Acc:AT2G03390]	4.5	12.626	Cleavage	miRNA 21 ACGUGCACGGGACGAAGAGGU 1<br>: :..:::::.:::::.: ::<br>Target 1482 UACGUGUGCCUUGCUUUUACA 1502
zma-miR164b-5p	GRMZM2G124321_T02	NA	uvrB/uvrC motif-containing protein [Source:Projected from Arabidopsis thaliana (AT2G03390) TAIR;Acc:AT2G03390]	4.5	12.626	Cleavage	miRNA 21 ACGUGCACGGGACGAAGAGGU 1<br>: :..:::::.:::::.: ::<br>Target 1475 UACGUGUGCCUUGCUUUUACA 1495
zma-miR164b-5p	GRMZM2G124321_T03	NA	uvrB/uvrC motif-containing protein [Source:Projected from Arabidopsis thaliana (AT2G03390) TAIR;Acc:AT2G03390]	4.5	12.626	Cleavage	miRNA 21 ACGUGCACGGGACGAAGAGGU 1<br>: :..:::::.:::::.: ::<br>Target 2018 UACGUGUGCCUUGCUUUUACA 2038
zma-miR164b-5p	GRMZM2G124321_T04	NA	uvrB/uvrC motif-containing protein [Source:Projected from Arabidopsis thaliana (AT2G03390) TAIR;Acc:AT2G03390]	4.5	12.626	Cleavage	miRNA 21 ACGUGCACGGGACGAAGAGGU 1<br>: :..:::::.:::::.: ::<br>Target 1942 UACGUGUGCCUUGCUUUUACA 1962

Potential targets of zma-miRNAs.

miRNA	Target	Gene name	Gene description	Expectation	UPE	Inhibition	Hybrid
zma-miR164b-5p	GRMZM2G124321_T05	NA	uvrB/uvrC motif-containing protein [Source:Projected from Arabidopsis thaliana (AT2G03390) TAIR;Acc:AT2G03390]	4.5	12.626	Cleavage	miRNA 21 ACGUGCACGGGACGAAGAGGU 1<br>: :..:::::.:::::.: ::<br>Target 1526 UACGUGUGCCUUGCUUUUACA 1546
zma-miR164b-5p	GRMZM2G140893_T01	NA	NA	4.5	21.96	Cleavage	miRNA 20 CGUGCACGGGACGAAGAGGU 1<br>.::::::::::: :: ::<br>Target 390 CUACGUGCCCUGCUACUACA 409
zma-miR164b-5p	GRMZM2G140893_T02	NA	NA	4.5	21.96	Cleavage	miRNA 20 CGUGCACGGGACGAAGAGGU 1<br>.::::::::::: :: ::<br>Target 390 CUACGUGCCCUGCUACUACA 409
zma-miR164b-5p	GRMZM2G308944_T01	NA	Photosystem II core complex protein psbY [Source:UniProtKB/TrEMBL;Acc:K7UBV2]	4.5	13.087	Cleavage	miRNA 21 ACGUGCACGGGACGAAGAGGU 1<br>: .:::::::.::.::.:.:<br>Target 856 UCUACGUGCCUUGUUUUUUCC 876
zma-miR164b-5p	GRMZM2G356338_T01	NA	Uncharacterized protein [Source:UniProtKB/TrEMBL;Acc:B4FC85]	4.5	18.62	Cleavage	miRNA 21 ACGUGCACGGGACGAAGAGGU 1<br>:::.::: ::.:::: ::::<br>Target 155 UGCGCGUCCCUUGCUCCUCCC 175
zma-miR164b-5p	GRMZM2G414373_T01	NA	PB1 domain containing protein [Source:UniProtKB/TrEMBL;Acc:B6TWP6]	4.5	16.724	Cleavage	miRNA 21 ACGUGCACGGGACGAAGAGGU 1<br>: :.:::::.:::::::: :<br>Target 1141 UUCGCGUGCUCUGCUUCUGCU 1161
zma-miR164b-5p	GRMZM5G807267_T02	NA	Uncharacterized protein [Source:UniProtKB/TrEMBL;Acc:B4FMZ2]	4.5	17.934	Translation	miRNA 21 ACGUGCACGGGACGAAGAGGU 1<br>:::..::::::: :::.:..<br>Target 38 UGCGUGUGCCCUCCUUUUUUU 58
zma-miR164b-5p	GRMZM2G011520_T01	NA	Rhodanese/Cell cycle control phosphatase superfamily protein [Source:Projected from Arabidopsis thaliana (AT5G03455) TAIR;Acc:AT5G03455]	5	16.714	Cleavage	miRNA 21 ACGUGCACGGGACGAAGAGGU 1<br>::::.:::::.::: :::..<br>Target 721 UGCAUGUGCCUUGCAGCUCUG 741
zma-miR164b-5p	GRMZM2G011520_T02	NA	Rhodanese/Cell cycle control phosphatase superfamily protein [Source:Projected from Arabidopsis thaliana (AT5G03455) TAIR;Acc:AT5G03455]	5	16.714	Cleavage	miRNA 21 ACGUGCACGGGACGAAGAGGU 1<br>::::.:::::.::: :::..<br>Target 491 UGCAUGUGCCUUGCAGCUCUG 511
zma-miR164b-5p	GRMZM2G057091_T01	NA	Uncharacterized protein [Source:UniProtKB/TrEMBL;Acc:B4FLJ9]	5	16.223	Cleavage	miRNA 21 ACGUGCACGGGACGAAGAGGU 1<br>::::.::::..::.::. ::<br>Target 1252 UGCAUGUGCUUUGUUUUGCCU 1272
zma-miR164b-5p	GRMZM2G057091_T02	NA	Uncharacterized protein [Source:UniProtKB/TrEMBL;Acc:B4FLJ9]	5	16.223	Cleavage	miRNA 21 ACGUGCACGGGACGAAGAGGU 1<br>::::.::::..::.::. ::<br>Target 1275 UGCAUGUGCUUUGUUUUGCCU 1295
zma-miR164b-5p	GRMZM2G057091_T03	NA	Uncharacterized protein [Source:UniProtKB/TrEMBL;Acc:B4FLJ9]	5	16.223	Cleavage	miRNA 21 ACGUGCACGGGACGAAGAGGU 1<br>::::.::::..::.::. ::<br>Target 1457 UGCAUGUGCUUUGUUUUGCCU 1477
zma-miR164b-5p	GRMZM2G063390_T02	NA	NA	5	17.541	Cleavage	miRNA 21 ACGUGCACGGGACGAAGAGGU 1<br>::::.:::.:.::.:: :...<br>Target 99 UGCAUGUGUCUUGUUUAUUUG 119
zma-miR164b-5p	GRMZM2G338160_T01	NA	Putative uncharacterized protein [Source:UniProtKB/TrEMBL;Acc:B6UBX3]	5	23.303	Cleavage	miRNA 21 ACGUGCACGGGACGAAGAGGU 1<br>::...:::::.:::: :: ::<br>Target 1021 UGUGUGUGCCUUGCUGCUGCA 1041
zma-miR164b-5p	GRMZM2G405581_T01	NA	Uncharacterized protein [Source:UniProtKB/TrEMBL;Acc:B8A1V6]	5	9.32	Cleavage	miRNA 20 CGUGCACGGGACGAAGAGGU 1<br>. ::::::.:::::.:::<br>Target 536 UUCCGUGCCUUGCUUUUCCC 555

Potential targets of zma-miRNAs.

miRNA	Target	Gene name	Gene description	Expectation	UPE	Inhibition	Hybrid
zma-miR164b-5p	GRMZM2G405581_T04	NA	Uncharacterized protein [Source:UniProtKB/TrEMBL;Acc:B8A1V6]	5	9.32	Cleavage	miRNA 20 CGUGCACGGGACGAAGAGGU 1<br>. :::::.:::::.:::<br>Target 633 UUCCGUGCCUUGCUUUUCCC 652
zma-miR164b-5p	GRMZM5G830545_T02	NA	Uncharacterized protein [Source:UniProtKB/TrEMBL;Acc:C0P5Q1]	5	18.456	Cleavage	miRNA 21 ACGUGCACGGGACGAAGAGGU 1<br>::.::::::.:::.::. .:<br>Target 1675 UGUACGUGCUCUGUUUUCUCU 1695
zma-miR164b-5p	GRMZM5G830545_T03	NA	Uncharacterized protein [Source:UniProtKB/TrEMBL;Acc:C0P5Q1]	5	18.456	Cleavage	miRNA 21 ACGUGCACGGGACGAAGAGGU 1<br>::.::::::.:::.::. .:<br>Target 1537 UGUACGUGCUCUGUUUUCUCU 1557
zma-miR168a-5p	GRMZM2G162356_T01	NA	Anaphase-promoting complex subunit 11 [Source:UniProtKB/TrEMBL;Acc:B6TKR6]	2.5	19.552	Cleavage	miRNA 20 AGGGCUAGACGUGGUUCGCU 1<br>::. :: ::::::::::::<br>Target 478 UCUAGAACUGCACCAAGCGA 497
zma-miR168a-5p	GRMZM2G045128_T01	NA	NA	3	11.643	Translation	miRNA 20 AGGGCUAGACGUGGUUCGCU 1<br>::.: :::::: :::::.::<br>Target 257 UCUCUAUCUGCCCCAAGUGA 276
zma-miR168a-5p	GRMZM2G083459_T01	NA	Discordia 1; Discordia1 [Source:UniProtKB/TrEMBL;Acc:B7UBT1]	3	18.096	Cleavage	miRNA 20 AGGGCUAGACGUGGUUCGCU 1<br>::. :::.::::::::::<br>Target 1825 UCUAGAUUUGCACCAAGCGC 1844
zma-miR168a-5p	GRMZM2G083459_T02	NA	Discordia 1; Discordia1 [Source:UniProtKB/TrEMBL;Acc:B7UBT1]	3	18.096	Cleavage	miRNA 20 AGGGCUAGACGUGGUUCGCU 1<br>::. :::.::::::::::<br>Target 1821 UCUAGAUUUGCACCAAGCGC 1840
zma-miR168a-5p	GRMZM2G157822_T02	NA	NA	3	22.835	Translation	miRNA 20 AGGGCUAGACGUGGUUCGCU 1<br>:::::: :: : ::::::::<br>Target 287 UCCCGACCUUCUCCAAGCGA 306
zma-miR168a-5p	GRMZM2G157822_T03	NA	NA	3	22.835	Translation	miRNA 20 AGGGCUAGACGUGGUUCGCU 1<br>:::::: :: : ::::::::<br>Target 287 UCCCGACCUUCUCCAAGCGA 306
zma-miR168a-5p	GRMZM2G361518_T01	NA	NA	3	23.803	Cleavage	miRNA 20 AGGGCUAGACGUGGUUCGCU 1<br>:::::: ::::.:::::: :<br>Target 279 UCCCGAGCUGCGCCAAGCAA 298
zma-miR168a-5p	GRMZM2G702086_T01	NA	NA	3	17.681	Translation	miRNA 20 AGGGCUAGACGUGGUUCGCU 1<br>:.::::::: :: ::::::.<br>Target 12 UUCCGAUCUCCAGCAAGCGG 31
zma-miR168a-5p	GRMZM2G702088_T01	NA	NA	3	15.949	Translation	miRNA 20 AGGGCUAGACGUGGUUCGCU 1<br>:.::::::: :: ::::::.<br>Target 12 UUCCGAUCUCCAGCAAGCGG 31
zma-miR168a-5p	AC199001.3_FGT005	NA	Uncharacterized protein [Source:UniProtKB/TrEMBL;Acc:K7UYI5]	3.5	21.887	Cleavage	miRNA 20 AGGGCUAGACGUGGUUCGCU 1<br>:::::: ::::::::::<br>Target 504 UCCCGAGCUGCACCAAGCCC 523
zma-miR168a-5p	AC209206.3_FGT011	NA	Putative argonaute family protein [Source:UniProtKB/TrEMBL;Acc:K7U605]	3.5	15.618	Cleavage	miRNA 20 AGGGCUAGACGUGGUUCGCU 1<br>:::::: ::::::::::<br>Target 429 UCCCGAGCUGCACCAAGCCC 448
zma-miR168a-5p	AC212023.4_FGT002	NA	NA	3.5	15.577	Cleavage	miRNA 20 AGGGCUAGACGUGGUUCGCU 1<br>::.:::: ::.:.::.:.::<br>Target 76 UCUCGAUGUGUAUCAGGUGA 95

Potential targets of zma-miRNAs.

miRNA	Target	Gene name	Gene description	Expectation	UPE	Inhibition	Hybrid
zma-miR168a-5p	GRMZM2G031588_T01	NA	Pentatricopeptide repeat (PPR) superfamily protein [Source:Projected from Arabidopsis thaliana (AT2G03380) TAIR;Acc:AT2G03380]	3.5	22.532	Cleavage	miRNA 20 AGGGCUAGACGUGGUUCGCU 1<br>:..::.:.::::::::: ::<br>Target 837 UUUCGGUUUGCACCAAGGGA 856
zma-miR168a-5p	GRMZM2G039455_T01	NA	Putative argonaute family protein [Source:GenBank;Acc:DAA36422]	3.5	19.106	Cleavage	miRNA 20 AGGGCUAGACGUGGUUCGCU 1<br>:::::: :::::::::::<br>Target 591 UCCCGAGCUGCACCAAGCCC 610
zma-miR168a-5p	GRMZM2G110646_T01	NA	CDP-diacylglycerol--inositol 3-phosphatidyltransferase 1; Phosphatidylinositol synthase; Uncharacterized protein [Source:UniProtKB/TrEMBL;Acc:B4FND0]	3.5	19.145	Translation	miRNA 20 AGGGCUAGACGUGGUUCGCU 1<br>:::.:::: : :.:.::::<br>Target 269 UCCUGAUCUUCUCUAGGCGA 288
zma-miR168a-5p	GRMZM2G335930_T01	NA	Uncharacterized protein [Source:UniProtKB/TrEMBL;Acc:B4FZ22]	3.5	18.573	Cleavage	miRNA 21 CAGGGCUAGACGUGGUUCGCU 1<br>:::.. ::.::.:::::::.<br>Target 1925 GUCUUAAUUUGUACCAAGCGG 1945
zma-miR168a-5p	GRMZM2G441583_T01	NA	Putative argonaute family protein [Source:UniProtKB/TrEMBL;Acc:K7UF32]	3.5	23.07	Cleavage	miRNA 20 AGGGCUAGACGUGGUUCGCU 1<br>:::::: :::::::::::<br>Target 626 UCCCGAGCUGCACCAAGCCC 645
zma-miR168a-5p	GRMZM2G700046_T01	NA	NA	3.5	11.576	Translation	miRNA 20 AGGGCUAGACGUGGUUCGCU 1<br>:.:. :::: ::::.::::<br>Target 57 UUCUUAUCUUCACCGAGCGA 76
zma-miR168a-5p	AC203535.4_FGT002	NA	NAC domain-containing protein 68 [Source:UniProtKB/TrEMBL;Acc:B6TYA5]	4	21.913	Cleavage	miRNA 20 AGGGCUAGACGUGGUUCGCU 1<br>::: .:: ::::::::::.<br>Target 546 GCCCCGUCGGCACCAAGCGG 565
zma-miR168a-5p	GRMZM2G024992_T01	NA	Uncharacterized protein [Source:UniProtKB/TrEMBL;Acc:B4FBT8]	4	17.181	Translation	miRNA 20 AGGGCUAGACGUGGUUCGCU 1<br>::: :::: ::.::::.::<br>Target 3350 ACCCAAUCUCCAUCAAGUGA 3369
zma-miR168a-5p	GRMZM2G049568_T01	NA	Ubiquitin carboxyl-terminal hydrolase [Source:UniProtKB/TrEMBL;Acc:B6TEU9]	4	16.53	Translation	miRNA 20 AGGGCUAGACGUGGUUCGCU 1<br>::::::::: ..::::::<br>Target 49 GCCCGAUCUGAGUCAAGCGU 68
zma-miR168a-5p	GRMZM2G049568_T02	NA	Ubiquitin carboxyl-terminal hydrolase [Source:UniProtKB/TrEMBL;Acc:B6TEU9]	4	16.63	Translation	miRNA 20 AGGGCUAGACGUGGUUCGCU 1<br>::::::::: ..::::::<br>Target 47 GCCCGAUCUGAGUCAAGCGU 66
zma-miR168a-5p	GRMZM2G067299_T01	NA	Uncharacterized protein [Source:UniProtKB/TrEMBL;Acc:B4F9U5]	4	13.191	Translation	miRNA 20 AGGGCUAGACGUGGUUCGCU 1<br>:.: :.::: :::::::::<br>Target 102 UUCAGGUCUCCACCAAGCGC 121
zma-miR168a-5p	GRMZM2G067299_T02	NA	Uncharacterized protein [Source:UniProtKB/TrEMBL;Acc:B4F9U5]	4	13.191	Translation	miRNA 20 AGGGCUAGACGUGGUUCGCU 1<br>:.: :.::: :::::::::<br>Target 102 UUCAGGUCUCCACCAAGCGC 121
zma-miR168a-5p	GRMZM2G067299_T03	NA	Uncharacterized protein [Source:UniProtKB/TrEMBL;Acc:B4F9U5]	4	13.191	Translation	miRNA 20 AGGGCUAGACGUGGUUCGCU 1<br>:.: :.::: :::::::::<br>Target 102 UUCAGGUCUCCACCAAGCGC 121
zma-miR168a-5p	GRMZM2G067299_T04	NA	Uncharacterized protein [Source:UniProtKB/TrEMBL;Acc:B4F9U5]	4	13.191	Translation	miRNA 20 AGGGCUAGACGUGGUUCGCU 1<br>:.: :.::: :::::::::<br>Target 102 UUCAGGUCUCCACCAAGCGC 121
zma-miR168a-5p	GRMZM2G067299_T05	NA	Uncharacterized protein [Source:UniProtKB/TrEMBL;Acc:B4F9U5]	4	15.404	Translation	miRNA 20 AGGGCUAGACGUGGUUCGCU 1<br>:.: :.::: :::::::::<br>Target 51 UUCAGGUCUCCACCAAGCGC 70

Potential targets of zma-miRNAs.

miRNA	Target	Gene name	Gene description	Expectation	UPE	Inhibition	Hybrid
zma-miR168a-5p	GRMZM2G067299_T06	NA	Uncharacterized protein [Source:UniProtKB/TrEMBL;Acc:B4F9U5]	4	16.027	Translation	miRNA 20 AGGGCUAGACGUGGUUCGCU 1<br>:.: :.::: :::::::::<br>Target 46 UUCAGGUCUCCACCAAGCGC 65
zma-miR168a-5p	GRMZM2G089484_T01	NA	Mitogen activated protein kinase 6; Putative MAP kinase family protein; Uncharacterized protein [Source:UniProtKB/TrEMBL;Acc:Q6TAR9]	4	21.487	Cleavage	miRNA 20 AGGGCUAGACGUGGUUCGCU 1<br>.:.:::::::::::. :<br>Target 673 GUCUGAUCUGCACCAAGUUA 692
zma-miR168a-5p	GRMZM2G095757_T01	NA	Uncharacterized protein [Source:UniProtKB/TrEMBL;Acc:K7VPH2]	4	19.422	Cleavage	miRNA 20 AGGGCUAGACGUGGUUCGCU 1<br>:::.::::::::.::::.<br>Target 1397 UCCUGAUCUGCAUCAAGUUU 1416
zma-miR168a-5p	GRMZM2G095757_T02	NA	Uncharacterized protein [Source:UniProtKB/TrEMBL;Acc:K7VPH2]	4	19.422	Cleavage	miRNA 20 AGGGCUAGACGUGGUUCGCU 1<br>:::.::::::::.::::.<br>Target 2458 UCCUGAUCUGCAUCAAGUUU 2477
zma-miR168a-5p	GRMZM2G132465_T01	NA	Uncharacterized protein [Source:UniProtKB/TrEMBL;Acc:B4FLV1]	4	22.053	Cleavage	miRNA 20 AGGGCUAGACGUGGUUCGCU 1<br>::::: ::::::.: ::::<br>Target 712 GCCCGAGCUGCACUACGCGA 731
zma-miR168a-5p	GRMZM2G132465_T02	NA	Uncharacterized protein [Source:UniProtKB/TrEMBL;Acc:B4FLV1]	4	22.053	Cleavage	miRNA 20 AGGGCUAGACGUGGUUCGCU 1<br>::::: ::::::.: ::::<br>Target 712 GCCCGAGCUGCACUACGCGA 731
zma-miR168a-5p	GRMZM2G132465_T03	NA	Uncharacterized protein [Source:UniProtKB/TrEMBL;Acc:B4FLV1]	4	22.053	Cleavage	miRNA 20 AGGGCUAGACGUGGUUCGCU 1<br>::::: ::::::.: ::::<br>Target 712 GCCCGAGCUGCACUACGCGA 731
zma-miR168a-5p	GRMZM2G132465_T04	NA	Uncharacterized protein [Source:UniProtKB/TrEMBL;Acc:B4FLV1]	4	22.053	Cleavage	miRNA 20 AGGGCUAGACGUGGUUCGCU 1<br>::::: ::::::.: ::::<br>Target 712 GCCCGAGCUGCACUACGCGA 731
zma-miR168a-5p	GRMZM2G132465_T05	NA	Uncharacterized protein [Source:UniProtKB/TrEMBL;Acc:B4FLV1]	4	22.053	Cleavage	miRNA 20 AGGGCUAGACGUGGUUCGCU 1<br>::::: ::::::.: ::::<br>Target 712 GCCCGAGCUGCACUACGCGA 731
zma-miR168a-5p	GRMZM2G154892_T01	NA	Uncharacterized protein [Source:UniProtKB/TrEMBL;Acc:K7V063]	4	18.981	Cleavage	miRNA 21 CAGGGCUAGACGUGGUUCGCU 1<br>:::: ::.:::: ::::.::<br>Target 945 GUCCAUAUUUGCAACAAGUGA 965
zma-miR168a-5p	GRMZM2G306028_T01	NA	Putative MAP kinase family protein [Source:UniProtKB/TrEMBL;Acc:K7UDV1]	4	18.525	Translation	miRNA 20 AGGGCUAGACGUGGUUCGCU 1<br>.:.::::: ::.::::.::<br>Target 382 GUCUGAUCUCCAUCAAGUGA 401
zma-miR168a-5p	GRMZM2G306028_T02	NA	Putative MAP kinase family protein [Source:UniProtKB/TrEMBL;Acc:K7UDV1]	4	18.525	Translation	miRNA 20 AGGGCUAGACGUGGUUCGCU 1<br>.:.::::: ::.::::.::<br>Target 508 GUCUGAUCUCCAUCAAGUGA 527
zma-miR168a-5p	GRMZM2G349709_T01	NA	Uncharacterized protein [Source:UniProtKB/TrEMBL;Acc:K7TKD5]	4	24.621	Translation	miRNA 21 CAGGGCUAGACGUGGUUCGCU 1<br>:::.: ::: ::::.:::::<br>Target 565 GUCUCCAUCGACACCGAGCGA 585
zma-miR168a-5p	GRMZM2G349709_T02	NA	Uncharacterized protein [Source:UniProtKB/TrEMBL;Acc:K7TKD5]	4	24.621	Translation	miRNA 21 CAGGGCUAGACGUGGUUCGCU 1<br>:::.: ::: ::::.:::::<br>Target 565 GUCUCCAUCGACACCGAGCGA 585
zma-miR168a-5p	GRMZM2G372928_T01	NA	Uncharacterized protein [Source:UniProtKB/TrEMBL;Acc:K7URC0]	4	22.211	Cleavage	miRNA 20 AGGGCUAGACGUGGUUCGCU 1<br>: :::: :::.:::.::::<br>Target 285 UGCCGACCUGUACCGAGCGU 304

Potential targets of zma-miRNAs.

miRNA	Target	Gene name	Gene description	Expectation	UPE	Inhibition	Hybrid
zma-miR168a-5p	GRMZM2G374088_T01	NA	Putative MAP kinase family protein isoform 1; Putative MAP kinase family protein isoform 2; Putative MAP kinase family protein isoform 3; Putative MAP kinase family protein isoform 4; Uncharacterized protein [Source:UniProtKB/TrEMBL;Acc:B4FQC8]	4	22.821	Cleavage	miRNA 20 AGGGCUAGACGUGGUUCGCU 1<br>.:.:: ::::.::::.::<br>Target 370 GUCUGACCUGCAUCAAGUGA 389
zma-miR168a-5p	GRMZM2G374088_T02	NA	Putative MAP kinase family protein isoform 1; Putative MAP kinase family protein isoform 2; Putative MAP kinase family protein isoform 3; Putative MAP kinase family protein isoform 4; Uncharacterized protein [Source:UniProtKB/TrEMBL;Acc:B4FQC8]	4	22.821	Cleavage	miRNA 20 AGGGCUAGACGUGGUUCGCU 1<br>.:.:: ::::.::::.::<br>Target 752 GUCUGACCUGCAUCAAGUGA 771
zma-miR168a-5p	GRMZM2G374088_T03	NA	Putative MAP kinase family protein isoform 1; Putative MAP kinase family protein isoform 2; Putative MAP kinase family protein isoform 3; Putative MAP kinase family protein isoform 4; Uncharacterized protein [Source:UniProtKB/TrEMBL;Acc:B4FQC8]	4	22.821	Cleavage	miRNA 20 AGGGCUAGACGUGGUUCGCU 1<br>.:.:: ::::.::::.::<br>Target 800 GUCUGACCUGCAUCAAGUGA 819
zma-miR168a-5p	GRMZM2G374088_T04	NA	Putative MAP kinase family protein isoform 1; Putative MAP kinase family protein isoform 2; Putative MAP kinase family protein isoform 3; Putative MAP kinase family protein isoform 4; Uncharacterized protein [Source:UniProtKB/TrEMBL;Acc:B4FQC8]	4	22.821	Cleavage	miRNA 20 AGGGCUAGACGUGGUUCGCU 1<br>.:.:: ::::.::::.::<br>Target 565 GUCUGACCUGCAUCAAGUGA 584
zma-miR168a-5p	GRMZM2G428835_T01	NA	NA	4	16.329	Translation	miRNA 20 AGGGCUAGACGUGGUUCGCU 1<br>:.:: ::: ::::::.::<br>Target 225 CCUCGCUCUCCACCAAGUGA 244
zma-miR168a-5p	GRMZM2G438755_T01	NA	NA	4	15.685	Cleavage	miRNA 20 AGGGCUAGACGUGGUUCGCU 1<br>::.:.:.:::::::::.:<br>Target 966 ACCUGGUUUGCACCAAGUGU 985
zma-miR168a-5p	GRMZM2G454081_T03	NA	Putative uncharacterized protein [Source:UniProtKB/TrEMBL;Acc:B6TKU4]	4	14.52	Cleavage	miRNA 20 AGGGCUAGACGUGGUUCGCU 1<br>::..::::::...::::: :<br>Target 2682 UCUUGAUCUGUGUCAAGCUA 2701
zma-miR168a-5p	GRMZM2G470569_T01	NA	Uncharacterized protein [Source:UniProtKB/TrEMBL;Acc:K7V8U2]	4	22.103	Cleavage	miRNA 20 AGGGCUAGACGUGGUUCGCU 1<br>::. :::.::::::: :::.<br>Target 1153 UCUGGAUUUGCACCAUGCGG 1172
zma-miR168a-5p	GRMZM5G844094_T02	NA	Uncharacterized protein [Source:UniProtKB/TrEMBL;Acc:K7UER0]	4	20.213	Cleavage	miRNA 20 AGGGCUAGACGUGGUUCGCU 1<br>:.:.:: ::::::: .::::<br>Target 792 UUCUGAACUGCACCCGGCGA 811
zma-miR168a-5p	GRMZM5G884914_T01	NA	Uncharacterized protein [Source:UniProtKB/TrEMBL;Acc:K7URN9]	4	19.268	Translation	miRNA 21 CAGGGCUAGACGUGGUUCGCU 1<br>: ::::: :::: .::::.::<br>Target 405 GACCCGACCUGCCUCAAGUGA 425
zma-miR168a-5p	GRMZM2G004455_T01	NA	Uncharacterized protein [Source:UniProtKB/TrEMBL;Acc:K7UC69]	4.5	19.706	Translation	miRNA 21 CAGGGCUAGACGUGGUUCGCU 1<br>::: ::::.:: :::::: :.<br>Target 1360 GUCACGAUUUGGACCAAGAGG 1380
zma-miR168a-5p	GRMZM2G004455_T02	NA	Uncharacterized protein [Source:UniProtKB/TrEMBL;Acc:K7UC69]	4.5	19.706	Translation	miRNA 21 CAGGGCUAGACGUGGUUCGCU 1<br>::: ::::.:: :::::: :.<br>Target 1350 GUCACGAUUUGGACCAAGAGG 1370
zma-miR168a-5p	GRMZM2G009837_T01	NA	Uncharacterized protein [Source:UniProtKB/TrEMBL;Acc:B4FH23]	4.5	18.073	Cleavage	miRNA 20 AGGGCUAGACGUGGUUCGCU 1<br>:.::: ::::::: :.:::<br>Target 857 UUCCGUUCUGCACGAGGCGC 876
zma-miR168a-5p	GRMZM2G038820_T01	NA	Putative ARF GTPase activating domain protein with ankyrin repeat-containing protein; Uncharacterized protein [Source:UniProtKB/TrEMBL;Acc:C0PJM6]	4.5	20.119	Cleavage	miRNA 20 AGGGCUAGACGUGGUUCGCU 1<br>:.:.:::.::::...::.:<br>Target 99 UUCUGAUUUGCAUUGAGUGC 118
zma-miR168a-5p	GRMZM2G041055_T01	NA	Uncharacterized protein [Source:UniProtKB/TrEMBL;Acc:C0P2P8]	4.5	22.729	Translation	miRNA 21 CAGGGCUAGACGUGGUUCGCU 1<br>:::.::: :: :::::.:.:<br>Target 766 GUCUCGACCUUCACCAGGUGC 786

Potential targets of zma-miRNAs.

miRNA	Target	Gene name	Gene description	Expectation	UPE	Inhibition	Hybrid
zma-miR168a-5p	GRMZM2G046574_T01	NA	TPR repeat region family protein; Uncharacterized protein [Source:UniProtKB/TrEMBL;Acc:B6SMW8]	4.5	21.222	Translation	miRNA 21 CAGGGCUAGACGUGGUUCGCU 1<br>: :::::::: :::::.:. :<br>Target 537 GACCCGAUCUACACCAGGUCA 557
zma-miR168a-5p	GRMZM2G052364_T01	NA	Uncharacterized protein [Source:UniProtKB/TrEMBL;Acc:K7V309]	4.5	18.433	Cleavage	miRNA 20 AGGGCUAGACGUGGUUCGCU 1<br>::::.:: ::::.:::: :<br>Target 1050 CCCCGGUCAGCACUAAGCAA 1069
zma-miR168a-5p	GRMZM2G055960_T01	NA	Uncharacterized protein [Source:UniProtKB/TrEMBL;Acc:B7ZXY1]	4.5	24.325	Cleavage	miRNA 20 AGGGCUAGACGUGGUUCGCU 1<br>::.: :::::.::::: ::<br>Target 835 UCUCCAUCUGUACCAAACGU 854
zma-miR168a-5p	GRMZM2G074501_T01	NA	Uncharacterized protein [Source:UniProtKB/TrEMBL;Acc:B4FZS7]	4.5	24.886	Cleavage	miRNA 20 AGGGCUAGACGUGGUUCGCU 1<br>:.::: ::::::: :.:::<br>Target 832 UUCCGUUCUGCACGAGGCGC 851
zma-miR168a-5p	GRMZM2G074501_T02	NA	Uncharacterized protein [Source:UniProtKB/TrEMBL;Acc:B4FZS7]	4.5	24.407	Cleavage	miRNA 20 AGGGCUAGACGUGGUUCGCU 1<br>:.::: ::::::: :.:::<br>Target 766 UUCCGUUCUGCACGAGGCGC 785
zma-miR168a-5p	GRMZM2G092120_T01	NA	Ankyrin-like protein isoform 1; Ankyrin-like protein isoform 2; Uncharacterized protein [Source:UniProtKB/TrEMBL;Acc:C4J1K3]	4.5	24.284	Translation	miRNA 20 AGGGCUAGACGUGGUUCGCU 1<br>:::::.::::: ::.:: :<br>Target 625 UCCCGGUCUGCCCCGAGAGC 644
zma-miR168a-5p	GRMZM2G092120_T02	NA	Ankyrin-like protein isoform 1; Ankyrin-like protein isoform 2; Uncharacterized protein [Source:UniProtKB/TrEMBL;Acc:C4J1K3]	4.5	24.284	Translation	miRNA 20 AGGGCUAGACGUGGUUCGCU 1<br>:::::.::::: ::.:: :<br>Target 625 UCCCGGUCUGCCCCGAGAGC 644
zma-miR168a-5p	GRMZM2G119258_T01	NA	Ethylene response protein [Source:UniProtKB/TrEMBL;Acc:K7U3I3]	4.5	18.182	Translation	miRNA 20 AGGGCUAGACGUGGUUCGCU 1<br>:::.:.:: :::.:::::<br>Target 1613 CGCCGGUUUGAACCGAGCGA 1632
zma-miR168a-5p	GRMZM2G124042_T01	NA	NA	4.5	20.002	Cleavage	miRNA 20 AGGGCUAGACGUGGUUCGCU 1<br>::::::: :::::. :::.<br>Target 1105 CCCCGAUCCGCACCGUGCGG 1124
zma-miR168a-5p	GRMZM2G134308_T02	NA	Uncharacterized protein [Source:UniProtKB/TrEMBL;Acc:C4J054]	4.5	22.293	Cleavage	miRNA 21 CAGGGCUAGACGUGGUUCGCU 1<br>::::: . :::::.::::: :<br>Target 689 GUCCCCGACUGCAUCAAGCAA 709
zma-miR168a-5p	GRMZM2G154574_T01	NA	Met-10+ like family protein / kelch repeat-containing protein [Source:Projected from Arabidopsis thaliana (AT4G04670) TAIR;Acc:AT4G04670]	4.5	15.615	Cleavage	miRNA 20 AGGGCUAGACGUGGUUCGCU 1<br>::..::::::::.. ::.:.<br>Target 1923 UCUUGAUCUGCAUUCAGUGG 1942
zma-miR168a-5p	GRMZM2G154574_T02	NA	Met-10+ like family protein / kelch repeat-containing protein [Source:Projected from Arabidopsis thaliana (AT4G04670) TAIR;Acc:AT4G04670]	4.5	15.615	Cleavage	miRNA 20 AGGGCUAGACGUGGUUCGCU 1<br>::..::::::::.. ::.:.<br>Target 1031 UCUUGAUCUGCAUUCAGUGG 1050
zma-miR168a-5p	GRMZM2G160430_T01	NA	Uncharacterized protein [Source:UniProtKB/TrEMBL;Acc:K7URW6]	4.5	18.685	Cleavage	miRNA 20 AGGGCUAGACGUGGUUCGCU 1<br>:.:::.:. :::.::::.:<br>Target 1440 UUCCGGUUAGCAUCAAGUGU 1459
zma-miR168a-5p	GRMZM2G160430_T02	NA	Uncharacterized protein [Source:UniProtKB/TrEMBL;Acc:K7URW6]	4.5	23.206	Cleavage	miRNA 20 AGGGCUAGACGUGGUUCGCU 1<br>:.:::.:. :::.::::.:<br>Target 345 UUCCGGUUAGCAUCAAGUGU 364
zma-miR168a-5p	GRMZM2G160430_T03	NA	Uncharacterized protein [Source:UniProtKB/TrEMBL;Acc:K7URW6]	4.5	19.037	Cleavage	miRNA 20 AGGGCUAGACGUGGUUCGCU 1<br>:.:::.:. :::.::::.:<br>Target 129 UUCCGGUUAGCAUCAAGUGU 148

miRNA	Target	Gene name	Gene description	Expectation	UPE	Inhibition	Hybrid
zma-miR168a-5p	GRMZM2G160430_T04	NA	Uncharacterized protein [Source:UniProtKB/TrEMBL;Acc:K7URW6]	4.5	18.685	Cleavage	miRNA 20 AGGGCUAGACGUGGUUCGCU 1<br>:.:::.:. :::.::::.:<br>Target 1329 UUCCGGUUAGCAUCAAGUGU 1348
zma-miR168a-5p	GRMZM2G177458_T02	NA	NA	4.5	15.092	Translation	miRNA 21 CAGGGCUAGACGUGGUUCGCU 1<br>:: :.:.::: :::::::: :<br>Target 1076 GUACUGGUCUUCACCAAGCAA 1096
zma-miR168a-5p	GRMZM2G333579_T01	NA	Uncharacterized protein [Source:UniProtKB/TrEMBL;Acc:K7UW84]	4.5	17.27	Translation	miRNA 21 CAGGGCUAGACGUGGUUCGCU 1<br>::: ::::.:: :::::: :.<br>Target 352 GUCACGAUUUGGACCAAGAGG 372
zma-miR168a-5p	GRMZM2G361256_T01	NA	Uncharacterized protein [Source:UniProtKB/TrEMBL;Acc:K7VHD1]	4.5	24.147	Cleavage	miRNA 21 CAGGGCUAGACGUGGUUCGCU 1<br>:::.: .::::::::: :.:.<br>Target 222 GUCUCUGUCUGCACCACGUGG 242
zma-miR168a-5p	GRMZM2G503308_T01	NA	NA	4.5	15.419	Translation	miRNA 21 CAGGGCUAGACGUGGUUCGCU 1<br>::::.:. :::: : ::::::<br>Target 734 GUCCUGGACUGCCCAAAGCGA 754
zma-miR168a-5p	GRMZM2G522468_T01	NA	Uncharacterized protein [Source:UniProtKB/TrEMBL;Acc:K7U6W5]	4.5	18.386	Cleavage	miRNA 20 AGGGCUAGACGUGGUUCGCU 1<br>::.: .::::::: :::::<br>Target 39 UCUCUGUCUGCACGAAGCGC 58
zma-miR168a-5p	GRMZM2G560440_T01	NA	Uncharacterized protein [Source:UniProtKB/TrEMBL;Acc:K7U6W5]	4.5	20.36	Cleavage	miRNA 20 AGGGCUAGACGUGGUUCGCU 1<br>::.: .::::::: :::::<br>Target 39 UCUCUGUCUGCACGAAGCGC 58
zma-miR168a-5p	GRMZM2G560444_T01	NA	Uncharacterized protein [Source:UniProtKB/TrEMBL;Acc:K7U6W5]	4.5	20.36	Cleavage	miRNA 20 AGGGCUAGACGUGGUUCGCU 1<br>::.: .::::::: :::::<br>Target 39 UCUCUGUCUGCACGAAGCGC 58
zma-miR168a-5p	GRMZM5G836588_T01	NA	Appr-1-p processing enzyme family protein [Source:UniProtKB/TrEMBL;Acc:B6UEI4]	4.5	21.927	Cleavage	miRNA 21 CAGGGCUAGACGUGGUUCGCU 1<br>::.: ::: ::.:::: ::::<br>Target 1728 GUUCAGAUAUGUACCACGCGA 1748
zma-miR168a-5p	GRMZM2G001652_T01	NA	NA	5	20.817	Cleavage	miRNA 20 AGGGCUAGACGUGGUUCGCU 1<br>::.: :::::.:::::::.<br>Target 1105 UCUCCAUCUGUACCAAGUUU 1124
zma-miR168a-5p	GRMZM2G017086_T01	NA	Ubiquitin carboxyl-terminal hydrolase [Source:UniProtKB/TrEMBL;Acc:B6T6V5]	5	16.766	Cleavage	miRNA 20 AGGGCUAGACGUGGUUCGCU 1<br>..:::.:::.:::::.:.<br>Target 1812 GAUUGAUUUGCGCCAAGUGG 1831
zma-miR168a-5p	GRMZM2G017086_T02	NA	Ubiquitin carboxyl-terminal hydrolase [Source:UniProtKB/TrEMBL;Acc:B6T6V5]	5	16.666	Cleavage	miRNA 20 AGGGCUAGACGUGGUUCGCU 1<br>..:::.:::.:::::.:.<br>Target 1898 GAUUGAUUUGCGCCAAGUGG 1917
zma-miR168a-5p	GRMZM2G017086_T03	NA	Ubiquitin carboxyl-terminal hydrolase [Source:UniProtKB/TrEMBL;Acc:B6T6V5]	5	16.666	Cleavage	miRNA 20 AGGGCUAGACGUGGUUCGCU 1<br>..:::.:::.:::::.:.<br>Target 1827 GAUUGAUUUGCGCCAAGUGG 1846
zma-miR168a-5p	GRMZM2G017086_T04	NA	Ubiquitin carboxyl-terminal hydrolase [Source:UniProtKB/TrEMBL;Acc:B6T6V5]	5	16.666	Cleavage	miRNA 20 AGGGCUAGACGUGGUUCGCU 1<br>..:::.:::.:::::.:.<br>Target 1763 GAUUGAUUUGCGCCAAGUGG 1782
zma-miR168a-5p	GRMZM2G039577_T01	NA	Uncharacterized protein [Source:UniProtKB/TrEMBL;Acc:C0P4Z3]	5	19.419	Cleavage	miRNA 21 CAGGGCUAGACGUGG-UUCGCU 1<br>:::.: .:.:::::: ::::::<br>Target 1651 GUCUCUGUUUGCACCGAAGCGA 1672

Potential targets of zma-miRNAs.

miRNA	Target	Gene name	Gene description	Expectation	UPE	Inhibition	Hybrid
zma-miR168a-5p	GRMZM2G039577_T02	NA	Uncharacterized protein [Source:UniProtKB/TrEMBL;Acc:C0P4Z3]	5	17.976	Cleavage	miRNA 21 CAGGGCUAGACGUGG-UUCGCU 1<br>:::.: .:.:::::: ::::::<br>Target 2110 GUCUCUGUUUGCACCGAAGCGA 2131
zma-miR168a-5p	GRMZM2G039577_T03	NA	Uncharacterized protein [Source:UniProtKB/TrEMBL;Acc:C0P4Z3]	5	18.199	Cleavage	miRNA 21 CAGGGCUAGACGUGG-UUCGCU 1<br>:::.: .:.:::::: ::::::<br>Target 1570 GUCUCUGUUUGCACCGAAGCGA 1591
zma-miR168a-5p	GRMZM2G039577_T04	NA	Uncharacterized protein [Source:UniProtKB/TrEMBL;Acc:C0P4Z3]	5	17.319	Cleavage	miRNA 21 CAGGGCUAGACGUGG-UUCGCU 1<br>:::.: .:.:::::: ::::::<br>Target 604 GUCUCUGUUUGCACCGAAGCGA 625
zma-miR168a-5p	GRMZM2G084440_T01	NA	GDP dissociation inhibitor family protein / Rab GTPase activator family protein [Source:Projected from Arabidopsis thaliana (AT5G09550) TAIR;Acc:AT5G09550]	5	24.559	Cleavage	miRNA 21 CAGGGCUAGACGUGGUU-CGCU 1<br>::::.:::: ::::::. :.::<br>Target 364 GUCCUGAUCCGCACCAGCGUGA 385
zma-miR168a-5p	GRMZM2G109472_T03	NA	Uncharacterized protein; Uridine 5-monophosphate synthase [Source:UniProtKB/TrEMBL;Acc:B4G0S0]	5	19.468	Cleavage	miRNA 21 CAGGGCUAGACGUGGUUCGCU 1<br>::... ::.::::.::::.:<br>Target 1722 GUUUUCAUUUGCAUCAAGUGU 1742
zma-miR168a-5p	GRMZM2G109472_T04	NA	Uncharacterized protein; Uridine 5-monophosphate synthase [Source:UniProtKB/TrEMBL;Acc:B4G0S0]	5	17.359	Cleavage	miRNA 21 CAGGGCUAGACGUGGUUCGCU 1<br>::... ::.::::.::::.:<br>Target 781 GUUUUCAUUUGCAUCAAGUGU 801
zma-miR168a-5p	GRMZM2G110834_T01	NA	Uncharacterized protein [Source:UniProtKB/TrEMBL;Acc:B4FJE2]	5	18.943	Cleavage	miRNA 20 AGGGCUAGACGUGGUUCGCU 1<br>:::. :::::::.::::.<br>Target 1617 UCCUAAUCUGCAUCAAGUUU 1636
zma-miR168a-5p	GRMZM2G110834_T02	NA	Uncharacterized protein [Source:UniProtKB/TrEMBL;Acc:B4FJE2]	5	18.943	Cleavage	miRNA 20 AGGGCUAGACGUGGUUCGCU 1<br>:::. :::::::.::::.<br>Target 1428 UCCUAAUCUGCAUCAAGUUU 1447
zma-miR168a-5p	GRMZM2G117989_T02	NA	Win1; Win1 isoform 1; Win1 isoform 2; Win1 isoform 3 [Source:UniProtKB/TrEMBL;Acc:B6SH12]	5	16.243	Cleavage	miRNA 21 CAGGGCUAGACGUGGUUCGCU 1<br>: :.:::::::::: :.:: :<br>Target 401 GGCUCGAUCUGCACAAGGCAA 421
zma-miR168a-5p	GRMZM2G124288_T02	NA	Uncharacterized protein [Source:UniProtKB/TrEMBL;Acc:C0P7N5]	5	17.264	Cleavage	miRNA 20 AGGGCU-AGACGUGGUUCGCU 1<br>:::::: :::::::::.::: .<br>Target 254 UCCCGAAUCUGCACCGAGCAG 274
zma-miR168a-5p	GRMZM2G139300_T01	NA	Beta-fructofuranosidase	5	18.26	Cleavage	miRNA 21 CAGGGCUAGACGUGGUUCGCU 1<br>:::: :::::.:: :. :::<br>Target 1586 GUCCGGAUCUGUACAAGCCGA 1606
zma-miR168a-5p	GRMZM2G157279_T01	NA	Uncharacterized protein [Source:UniProtKB/TrEMBL;Acc:K7VPM0]	5	14.612	Translation	miRNA 20 AGGGCUAGACGUGGUUCGCU 1<br>::.:::::: :::::::<br>Target 477 ACCUGAUCUGAACCAAGCUU 496
zma-miR168a-5p	GRMZM2G157279_T01	NA	Uncharacterized protein [Source:UniProtKB/TrEMBL;Acc:K7VPM0]	5	22.308	Translation	miRNA 20 AGGGCUAGACGUGGUUCGCU 1<br>::.:::::: :::::::<br>Target 267 ACCUGAUCUGAACCAAGCUU 286
zma-miR168a-5p	GRMZM2G157279_T01	NA	Uncharacterized protein [Source:UniProtKB/TrEMBL;Acc:K7VPM0]	5	23.851	Translation	miRNA 20 AGGGCUAGACGUGGUUCGCU 1<br>::.:::::: :::::::<br>Target 99 ACCUGAUCUGAACCAAGCUU 118
zma-miR168a-5p	GRMZM2G356076_T01	NA	NA	5	20.714	Cleavage	miRNA 21 CAGGGCUAGACGUGGUUCGCU 1<br>::.:::.::::::::: :<br>Target 1824 GUUCCGGUCUGCACCAACCCU 1844

Potential targets of zma-miRNAs.

miRNA	Target	Gene name	Gene description	Expectation	UPE	Inhibition	Hybrid
zma-miR168a-5p	GRMZM2G402088_T01	NA	NA	5	12.962	Cleavage	miRNA 20 AGGGCUAGACGUGGUUCGCU 1<br>:::.: :.::.::::::<br>Target 2755 UCCUGUUUUGUACCAAGCCU 2774
zma-miR168a-5p	GRMZM2G405684_T01	NA	Uncharacterized protein [Source:UniProtKB/TrEMBL;Acc:B6TNA1]	5	16.114	Cleavage	miRNA 20 AGGGCUAGACGUGGUUCGCU 1<br>:::: :::::::: ::: .<br>Target 427 UCCCCAUCUGCACCUAGCUG 446
zma-miR168a-5p	GRMZM2G416684_T01	NA	NA	5	17.145	Cleavage	miRNA 21 CAGGGCUAGACGUGGUUCGCU 1<br>:::.: .::::::.::. .::<br>Target 1355 GUCUCUGUCUGCAUCAGUUGA 1375
zma-miR168a-5p	GRMZM5G869299_T01	NA	Uncharacterized protein [Source:UniProtKB/TrEMBL;Acc:B4G0D6]	5	14.011	Cleavage	miRNA 21 CAGGGCUAGACGUGGUUCGCU 1<br>::::.::: ::..:..::.:<br>Target 1512 GUCCUGAUAUGUGCUGAGUGC 1532
zma-miR397b-5p	GRMZM2G072808_T01	NA	Uncharacterized protein [Source:UniProtKB/TrEMBL;Acc:C0PGP6]	0	15.392	Cleavage	miRNA 21 GUAGUUGCGACGCGAGUUACU 1<br>:::::::::::::::::::::<br>Target 810 CAUCAACGCUGCGCUCAAUGA 830
zma-miR397b-5p	AC192330.1_FGT004	NA	Uncharacterized protein [Source:UniProtKB/TrEMBL;Acc:K7TS37]	2	11.202	Cleavage	miRNA 21 GUAGUUGCGACGCGAGUUACU 1<br>..:.::::::::: :::::::<br>Target 885 UGUUAACGCUGCGAUCAAUGA 905
zma-miR397b-5p	AC209966.4_FGT005	NA	NA	2	15.099	Cleavage	miRNA 21 GUAGUUGCGACGCGAGUUACU 1<br>..:.::::::::: :::::::<br>Target 1161 UGUUAACGCUGCGAUCAAUGA 1181
zma-miR397b-5p	AF466202.2_FGT010	NA	Uncharacterized protein [Source:UniProtKB/TrEMBL;Acc:K7TVV7]	2	13.925	Cleavage	miRNA 21 GUAGUUGCGACGCGAGUUACU 1<br>..:.::::::::: :::::::<br>Target 1455 UGUUAACGCUGCGAUCAAUGA 1475
zma-miR397b-5p	GRMZM2G464246_T01	NA	NA	2	12.083	Cleavage	miRNA 21 GUAGUUGCGACGCGAGUUACU 1<br>..:.::::::::: :::::::<br>Target 1926 UGUUAACGCUGCGAUCAAUGA 1946
zma-miR397b-5p	GRMZM2G072780_T01	NA	Uncharacterized protein [Source:UniProtKB/TrEMBL;Acc:C0P789]	2.5	24.379	Cleavage	miRNA 21 GUAGUUGCGACGCGAGUUACU 1<br>::::::::: :::::::: ::<br>Target 820 CAUCAACGCGGCGCUCAACGA 840
zma-miR397b-5p	GRMZM2G073614_T01	NA	NA	2.5	12.111	Cleavage	miRNA 21 GUAGUUGCGACGCGAGUUACU 1<br>::::::: : :.:::::::::<br>Target 630 CAUCAACCCCGUGCUCAAUGA 650
zma-miR397b-5p	GRMZM2G164467_T01	NA	Uncharacterized protein [Source:UniProtKB/TrEMBL;Acc:C0P789]	2.5	24.379	Cleavage	miRNA 21 GUAGUUGCGACGCGAGUUACU 1<br>::::::::: :::::::: ::<br>Target 896 CAUCAACGCGGCGCUCAACGA 916
zma-miR397b-5p	GRMZM2G305526_T01	NA	Uncharacterized protein [Source:UniProtKB/TrEMBL;Acc:B4FDI9]	2.5	14.554	Cleavage	miRNA 21 GUAGUUGCGACGCGAGUUACU 1<br>::::::::: :::::::: ::<br>Target 846 CAUCAACGCCGCGCUCAACGA 866
zma-miR397b-5p	GRMZM2G447271_T01	NA	Putative laccase family protein [Source:UniProtKB/TrEMBL;Acc:K7V3L7]	2.5	21.58	Cleavage	miRNA 21 GUAGUUGCGACGCGAGUUACU 1<br>::::::::: :::::::: ::<br>Target 1201 CAUCAACGCGGCGCUCAACGA 1221
zma-miR397b-5p	GRMZM2G039454_T01	NA	Cellulose synthase-3 [Source:UniProtKB/TrEMBL;Acc:Q9LLI7]	3	23.772	Translation	miRNA 20 UAGUUGCGACGCGAGUUACU 1<br>::::..:::: :::::::<br>Target 230 AUCAGUGCUGCCCUCAAUGC 249

Potential targets of zma-miRNAs.

miRNA	Target	Gene name	Gene description	Expectation	UPE	Inhibition	Hybrid
zma-miR397b-5p	GRMZM2G039454_T02	NA	Cellulose synthase-3 [Source:UniProtKB/TrEMBL;Acc:Q9LLI7]	3	23.772	Translation	miRNA 20 UAGUUGCGACGCGAGUUACU 1<br>::::..::::: :::::::<br>Target 335 AUCAGUGCUGCCCUCAAUGC 354
zma-miR397b-5p	GRMZM2G039454_T03	NA	Cellulose synthase-3 [Source:UniProtKB/TrEMBL;Acc:Q9LLI7]	3	23.772	Translation	miRNA 20 UAGUUGCGACGCGAGUUACU 1<br>::::..::::: :::::::<br>Target 312 AUCAGUGCUGCCCUCAAUGC 331
zma-miR397b-5p	GRMZM2G051103_T01	NA	Putative CBL-interacting protein kinase family protein; Uncharacterized protein [Source:UniProtKB/TrEMBL;Acc:C0P6L2]	3	16.219	Translation	miRNA 21 GUAGUUGCGACGCGAGUUACU 1<br>:::..:.::::: .:::::::<br>Target 1147 CAUUGAUGCUGCAUUCAAUGA 1167
zma-miR397b-5p	GRMZM2G066489_T01	NA	Uncharacterized protein [Source:UniProtKB/TrEMBL;Acc:B8A0W8]	3	22.938	Cleavage	miRNA 21 GUAGUUGCGACGCGAGUUACU 1<br>::::. .:::::: :::::::<br>Target 477 CAUCGCUGCUGCGGUCAAUGA 497
zma-miR397b-5p	GRMZM2G066489_T02	NA	Uncharacterized protein [Source:UniProtKB/TrEMBL;Acc:B8A0W8]	3	22.938	Cleavage	miRNA 21 GUAGUUGCGACGCGAGUUACU 1<br>::::. .:::::: :::::::<br>Target 477 CAUCGCUGCUGCGGUCAAUGA 497
zma-miR397b-5p	GRMZM2G066489_T03	NA	Uncharacterized protein [Source:UniProtKB/TrEMBL;Acc:B8A0W8]	3	22.938	Cleavage	miRNA 21 GUAGUUGCGACGCGAGUUACU 1<br>::::. .:::::: :::::::<br>Target 288 CAUCGCUGCUGCGGUCAAUGA 308
zma-miR397b-5p	GRMZM2G102475_T01	NA	Uncharacterized protein [Source:UniProtKB/TrEMBL;Acc:B4FA58]	3	21.143	Translation	miRNA 21 GUAGUUGCGACGCGAGUUACU 1<br>:::::::: ::: :::::::<br>Target 1437 CAUCAACGAUGCAAUCAAUGA 1457
zma-miR397b-5p	GRMZM2G102475_T02	NA	Uncharacterized protein [Source:UniProtKB/TrEMBL;Acc:B4FA58]	3	21.143	Translation	miRNA 21 GUAGUUGCGACGCGAGUUACU 1<br>:::::::: ::: :::::::<br>Target 1324 CAUCAACGAUGCAAUCAAUGA 1344
zma-miR397b-5p	GRMZM2G102475_T03	NA	Uncharacterized protein [Source:UniProtKB/TrEMBL;Acc:B4FA58]	3	21.143	Translation	miRNA 21 GUAGUUGCGACGCGAGUUACU 1<br>:::::::: ::: :::::::<br>Target 1368 CAUCAACGAUGCAAUCAAUGA 1388
zma-miR397b-5p	GRMZM2G118800_T01	NA	Aldehyde dehydrogenase [Source:UniProtKB/TrEMBL;Acc:C0P7X2]	3	24.023	Cleavage	miRNA 20 UAGUUGCGACGCGAGUUACU 1<br>::::.::: :::::: ::::<br>Target 452 AUCAGCGCCGCGCUCCAUGA 471
zma-miR397b-5p	GRMZM2G118800_T02	NA	Aldehyde dehydrogenase [Source:UniProtKB/TrEMBL;Acc:C0P7X2]	3	24.023	Cleavage	miRNA 20 UAGUUGCGACGCGAGUUACU 1<br>::::.::: :::::: ::::<br>Target 452 AUCAGCGCCGCGCUCCAUGA 471
zma-miR397b-5p	GRMZM2G146152_T01	NA	Putative laccase family protein [Source:UniProtKB/TrEMBL;Acc:K7UTI2]	3	23.155	Cleavage	miRNA 21 GUAGUUGCGACGCGAGUUACU 1<br>:.::::::: :::::::: ::<br>Target 803 CGUCAACGCGGCGCUCAACGA 823
zma-miR397b-5p	GRMZM2G156754_T01	NA	Uncharacterized protein [Source:UniProtKB/TrEMBL;Acc:K7V3Q4]	3	24.67	Translation	miRNA 21 GUAGUUGCGACGCGAGUUACU 1<br>::::..:::::. :::.::::<br>Target 108 CAUCGGCGCUGUCCUCGAUGA 128
zma-miR397b-5p	GRMZM5G802725_T01	NA	NA	3	18.748	Translation	miRNA 21 GUAGUUGCGACGCGAGUUACU 1<br>:::..:.::::: .:::::::<br>Target 723 CAUUGAUGCUGCAUUCAAUGA 743
zma-miR397b-5p	AC190757.3_FGT001	NA	Uncharacterized protein [Source:UniProtKB/TrEMBL;Acc:K7VLX9]	3.5	11.947	Cleavage	miRNA 21 GUAGUUGCGACGCGAGUUACU 1<br>..:.::::::::: ::::::<br>Target 1827 UGUUAACGCUGCGAACAAUGA 1847

Potential targets of zma-miRNAs.

miRNA	Target	Gene name	Gene description	Expectation	UPE	Inhibition	Hybrid
zma-miR397b-5p	AC191526.3_FGT001	NA	NA	3.5	14.101	Cleavage	miRNA 21 GUAGUUGCGACGCGAGUUACU 1<br>..:.::::::::: ::::::<br>Target 2040 UGUUAACGCUGCGAACAAUGA 2060
zma-miR397b-5p	AC212463.3_FGT001	NA	NA	3.5	14.568	Cleavage	miRNA 21 GUAGUUGCGACGCGAGUUACU 1<br>..:.::::::::: ::::::<br>Target 1482 UGUUAACGCUGCGAACAAUGA 1502
zma-miR397b-5p	AC217056.3_FGT001	NA	NA	3.5	18.097	Cleavage	miRNA 21 GUAGUUGCGACGCGAGUUACU 1<br>..:. :.:::::::::::::<br>Target 2371 UGUUCAUGCUGCGCUCAAUGC 2391
zma-miR397b-5p	GRMZM2G009032_T01	NA	NA	3.5	15.671	Cleavage	miRNA 21 GUAGUUGCGACGCGAGUUACU 1<br>..:.::::::::: ::::::<br>Target 1389 UGUUAACGCUGCGAACAAUGA 1409
zma-miR397b-5p	GRMZM2G044348_T01	NA	Uncharacterized protein [Source:UniProtKB/TrEMBL;Acc:B4FL90]	3.5	9.758	Cleavage	miRNA 20 UAGUUGCGACGCGAGUUACU 1<br>.:::::::::.::::: ::<br>Target 580 GUCAACGCUGUGCUCACUGC 599
zma-miR397b-5p	GRMZM2G044348_T02	NA	Uncharacterized protein [Source:UniProtKB/TrEMBL;Acc:B4FL90]	3.5	9.758	Cleavage	miRNA 20 UAGUUGCGACGCGAGUUACU 1<br>.:::::::::.::::: ::<br>Target 580 GUCAACGCUGUGCUCACUGC 599
zma-miR397b-5p	GRMZM2G044348_T03	NA	Uncharacterized protein [Source:UniProtKB/TrEMBL;Acc:B4FL90]	3.5	9.758	Cleavage	miRNA 20 UAGUUGCGACGCGAGUUACU 1<br>.:::::::::.::::: ::<br>Target 481 GUCAACGCUGUGCUCACUGC 500
zma-miR397b-5p	GRMZM2G044348_T04	NA	Uncharacterized protein [Source:UniProtKB/TrEMBL;Acc:B4FL90]	3.5	9.758	Cleavage	miRNA 20 UAGUUGCGACGCGAGUUACU 1<br>.:::::::::.::::: ::<br>Target 580 GUCAACGCUGUGCUCACUGC 599
zma-miR397b-5p	GRMZM2G055575_T01	NA	Uncharacterized protein [Source:UniProtKB/TrEMBL;Acc:C0P3A2]	3.5	18.508	Translation	miRNA 21 GUAGUUGCGACGCGAGUUACU 1<br>:.:..:.::::: .:::::::<br>Target 1048 CGUUGAUGCUGCAUUCAAUGA 1068
zma-miR397b-5p	GRMZM2G055575_T02	NA	Uncharacterized protein [Source:UniProtKB/TrEMBL;Acc:C0P3A2]	3.5	15.82	Translation	miRNA 21 GUAGUUGCGACGCGAGUUACU 1<br>:.:..:.::::: .:::::::<br>Target 1048 CGUUGAUGCUGCAUUCAAUGA 1068
zma-miR397b-5p	GRMZM2G055575_T03	NA	Uncharacterized protein [Source:UniProtKB/TrEMBL;Acc:C0P3A2]	3.5	20.663	Translation	miRNA 21 GUAGUUGCGACGCGAGUUACU 1<br>:.:..:.::::: .:::::::<br>Target 841 CGUUGAUGCUGCAUUCAAUGA 861
zma-miR397b-5p	GRMZM2G067373_T01	NA	electron carriers;protein disulfide oxidoreductases [Source:Projected from Arabidopsis thaliana (AT4G08550) TAIR;Acc:AT4G08550]	3.5	21.538	Translation	miRNA 21 GUAGUUGCGACGCGAGUUACU 1<br>.:::.:.:.: .:.:::::::<br>Target 1053 UAUCGAUGUUCUGUUCAAUGA 1073
zma-miR397b-5p	GRMZM2G091588_T01	NA	Cytochrome P450 monooxygenase CYP92A1 [Source:UniProtKB/TrEMBL;Acc:Q8VYA9]	3.5	23.867	Cleavage	miRNA 21 GUAGUUGCGACGCGAGUUACU 1<br>::::.: ::::.:::::: ::<br>Target 1140 CAUCGAGGCUGUGCUCAAGGA 1160
zma-miR397b-5p	GRMZM2G094699_T02	NA	Uncharacterized protein [Source:UniProtKB/TrEMBL;Acc:C0PM23]	3.5	22.268	Cleavage	miRNA 21 GUAGUUGCGACGCGAGUUACU 1<br>:::::. ::::::::.:::.<br>Target 266 CAUCAGAUCUGCGCUCGAUGG 286
zma-miR397b-5p	GRMZM2G134214_T01	NA	Uncharacterized protein [Source:UniProtKB/TrEMBL;Acc:C0P2B1]	3.5	17.933	Cleavage	miRNA 21 GUAGUUGCGACGCGAGUUACU 1<br>:: :::.::::.:.:..::::<br>Target 1918 CAACAAUGCUGUGUUUGAUGA 1938

Potential targets of zma-miRNAs.

miRNA	Target	Gene name	Gene description	Expectation	UPE	Inhibition	Hybrid
zma-miR397b-5p	GRMZM2G147467_T01	NA	Flavonoid 3-monooxygenase [Source:UniProtKB/TrEMBL;Acc:B6TYK7]	3.5	19.823	Cleavage	miRNA 21 GUAGUUGCGACGCGAGUUACU 1<br>::::.: ::::.::::: ::<br>Target 1164 CAUCGAGGCUGUGCUCAAGGA 1184
zma-miR397b-5p	GRMZM2G150251_T01	NA	Uncharacterized protein [Source:UniProtKB/TrEMBL;Acc:B4FP26]	3.5	12.799	Cleavage	miRNA 20 UAGUUGCGACGCGAGUUACU 1<br>.:::::::::.::::: ::<br>Target 491 GUCAACGCUGUGCUCACUGC 510
zma-miR397b-5p	GRMZM2G150251_T02	NA	Uncharacterized protein [Source:UniProtKB/TrEMBL;Acc:B4FP26]	3.5	12.728	Cleavage	miRNA 20 UAGUUGCGACGCGAGUUACU 1<br>.:::::::::.::::: ::<br>Target 482 GUCAACGCUGUGCUCACUGC 501
zma-miR397b-5p	GRMZM2G168953_T01	NA	Putative lectin-like receptor protein kinase family protein; Uncharacterized protein [Source:UniProtKB/TrEMBL;Acc:C0PKZ2]	3.5	21.437	Cleavage	miRNA 21 GUAGUUGCGACGCGAGUUACU 1<br>:.:::: :: ::::::::::<br>Target 2700 CGUCAAAGCGGCGCUCAAUGC 2720
zma-miR397b-5p	GRMZM2G168953_T02	NA	Putative lectin-like receptor protein kinase family protein; Uncharacterized protein [Source:UniProtKB/TrEMBL;Acc:C0PKZ2]	3.5	21.437	Cleavage	miRNA 21 GUAGUUGCGACGCGAGUUACU 1<br>:.:::: :: ::::::::::<br>Target 882 CGUCAAAGCGGCGCUCAAUGC 902
zma-miR397b-5p	GRMZM2G168953_T03	NA	Putative lectin-like receptor protein kinase family protein; Uncharacterized protein [Source:UniProtKB/TrEMBL;Acc:C0PKZ2]	3.5	21.437	Cleavage	miRNA 21 GUAGUUGCGACGCGAGUUACU 1<br>:.:::: :: ::::::::::<br>Target 1347 CGUCAAAGCGGCGCUCAAUGC 1367
zma-miR397b-5p	GRMZM2G172726_T01	NA	Uncharacterized protein [Source:UniProtKB/TrEMBL;Acc:B4FA71]	3.5	18.317	Cleavage	miRNA 20 UAGUUGCGACGCGAGUUACU 1<br>:::::: ::.:.::.::::<br>Target 1019 UUCAACGAUGUGUUCGAUGA 1038
zma-miR397b-5p	GRMZM2G386923_T01	NA	Uncharacterized protein [Source:UniProtKB/TrEMBL;Acc:K7VG96]	3.5	22.07	Translation	miRNA 20 UAGUUGCGACGCGAGUUACU 1<br>:::::.:.::. :::::::<br>Target 1295 AUCAAUGUUGUCCUCAAUGC 1314
zma-miR397b-5p	GRMZM2G414141_T01	NA	Uncharacterized protein [Source:UniProtKB/TrEMBL;Acc:B4FIK6]	3.5	14.099	Cleavage	miRNA 21 GUAGUUGCGACGCGAGUUACU 1<br>.: ::: ::::.:::..::::<br>Target 3042 UAGCAAAGCUGUGCUUGAUGA 3062
zma-miR397b-5p	GRMZM2G414141_T02	NA	Uncharacterized protein [Source:UniProtKB/TrEMBL;Acc:B4FIK6]	3.5	14.099	Cleavage	miRNA 21 GUAGUUGCGACGCGAGUUACU 1<br>.: ::: ::::.:::..::::<br>Target 2565 UAGCAAAGCUGUGCUUGAUGA 2585
zma-miR397b-5p	GRMZM2G433333_T01	NA	Uncharacterized protein [Source:UniProtKB/TrEMBL;Acc:K7UZW2]	3.5	14.489	Translation	miRNA 20 UAGUUGCGACGCGAGUUACU 1<br>::.::.::::: :::.:::<br>Target 2008 AUUAAUGCUGCUGUCAGUGA 2027
zma-miR397b-5p	GRMZM2G433333_T02	NA	Uncharacterized protein [Source:UniProtKB/TrEMBL;Acc:K7UZW2]	3.5	14.489	Translation	miRNA 20 UAGUUGCGACGCGAGUUACU 1<br>::.::.::::: :::.:::<br>Target 2008 AUUAAUGCUGCUGUCAGUGA 2027
zma-miR397b-5p	GRMZM2G433333_T03	NA	Uncharacterized protein [Source:UniProtKB/TrEMBL;Acc:K7UZW2]	3.5	14.489	Translation	miRNA 20 UAGUUGCGACGCGAGUUACU 1<br>::.::.::::: :::.:::<br>Target 2008 AUUAAUGCUGCUGUCAGUGA 2027
zma-miR397b-5p	AC209050.3_FGT001	NA	Uncharacterized protein [Source:UniProtKB/TrEMBL;Acc:K7UYY3]	4	19.908	Cleavage	miRNA 21 GUAGUUGCGACGCGAGUUACU 1<br>::::: ::: :.:::::: ::<br>Target 1950 CAUCAUCGCCGUGCUCAACGA 1970
zma-miR397b-5p	AC220954.3_FGT005	NA	NA	4	17.744	Cleavage	miRNA 20 UAGUUGCGACGCGAGUUACU 1<br>.::.::::::.:.:::::<br>Target 1192 CCUAAUGCUGCGUUUAAUGA 1211

Potential targets of zma-miRNAs.

miRNA	Target	Gene name	Gene description	Expectation	UPE	Inhibition	Hybrid
zma-miR397b-5p	GRMZM2G005024_T01	TSB2	Tryptophan synthase beta chain 2	4	24.202	Translation	miRNA 21 GUAGUUGCGACGCGAGUUACU 1<br>:::::..:::: ::::.:: :<br>Target 1210 CAUCAGUGCUGGGCUCGAUUA 1230
zma-miR397b-5p	GRMZM2G010176_T01	NA	Uncharacterized protein [Source:UniProtKB/TrEMBL;Acc:B4FZC1]	4	15.113	Cleavage	miRNA 21 GUAGUUGCGACGCGAGUUACU 1<br>:::.. ::. :.:::::::::<br>Target 1796 CAUUGUCGUCGUGCUCAAUGA 1816
zma-miR397b-5p	GRMZM2G024834_T02	NA	Putative glycogen synthase kinase family protein [Source:UniProtKB/TrEMBL;Acc:K7U9E2]	4	16.919	Translation	miRNA 21 GUAGUUGCGACGCGAGUUACU 1<br>:.:..:.:::: .::::::.<br>Target 1125 CGUUGAUGCUGCAUUCAAUGG 1145
zma-miR397b-5p	GRMZM2G029531_T01	NA	NA	4	17.008	Translation	miRNA 21 GUAGUUGCGACGCGAGUUACU 1<br>..::.::: :: :.::.::::<br>Target 763 UGUCGACGAUGAGUUCGAUGA 783
zma-miR397b-5p	GRMZM2G029531_T02	NA	NA	4	15.212	Translation	miRNA 21 GUAGUUGCGACGCGAGUUACU 1<br>..::.::: :: :.::.::::<br>Target 763 UGUCGACGAUGAGUUCGAUGA 783
zma-miR397b-5p	GRMZM2G043415_T01	NA	Uncharacterized protein [Source:UniProtKB/TrEMBL;Acc:C0PMP7]	4	19.819	Cleavage	miRNA 21 GUAGUUGCGACGCGAGUUACU 1<br>:::::::: ::::::..::<br>Target 1032 CAUCAACGACGCGCUCGGUGC 1052
zma-miR397b-5p	GRMZM2G050687_T01	NA	Uncharacterized protein [Source:UniProtKB/TrEMBL;Acc:K7VJ16]	4	17.255	Cleavage	miRNA 21 GUAGUUGCGACGCGAGUUACU 1<br>..:. :.::::::.::::::<br>Target 1432 UGUUCAUGCUGCGUUCAAUGC 1452
zma-miR397b-5p	GRMZM2G089132_T01	NA	Uncharacterized protein [Source:UniProtKB/TrEMBL;Acc:C0P9N0]	4	20.727	Cleavage	miRNA 20 UAGUUGCGACGCGAGUUACU 1<br>:::.:::::::.:.: :::<br>Target 1398 UUCAGCGCUGCGUUUAUUGA 1417
zma-miR397b-5p	GRMZM2G123459_T01	NA	Uncharacterized protein [Source:UniProtKB/TrEMBL;Acc:C0HHR1]	4	19.683	Cleavage	miRNA 21 GUAGUUGCGACGCGAGUUACU 1<br>:: : :.:.::.::::.::::<br>Target 1456 CAACUAUGUUGUGCUCGAUGA 1476
zma-miR397b-5p	GRMZM2G131309_T01	NA	Uncharacterized protein [Source:UniProtKB/TrEMBL;Acc:K7WAJ9]	4	20.643	Cleavage	miRNA 21 GUAGUUGCGACGCGAGUUACU 1<br>::::: ::: :.:::::: ::<br>Target 2225 CAUCAUCGCCGUGCUCAACGA 2245
zma-miR397b-5p	GRMZM2G140107_T01	NA	NA	4	22.81	Translation	miRNA 21 GUAGUUGCGACGCGAGUUACU 1<br>::::::: :::: :::. :::<br>Target 1911 CAUCAACCCUGCUCUCGUUGA 1931
zma-miR397b-5p	GRMZM2G153292_T01	TUBA2	Tubulin alpha-1 chain [Source:UniProtKB/Swiss-Prot;Acc:P14640]	4	18.932	Translation	miRNA 20 UAGUUGCGACGCGAGUUACU 1<br>.::...:::: :.::.::::<br>Target 1464 GUCGGUGCUGAGUUCGAUGA 1483
zma-miR397b-5p	GRMZM2G153292_T02	TUBA2	Tubulin alpha-1 chain [Source:UniProtKB/Swiss-Prot;Acc:P14640]	4	18.932	Translation	miRNA 20 UAGUUGCGACGCGAGUUACU 1<br>.::...:::: :.::.::::<br>Target 1522 GUCGGUGCUGAGUUCGAUGA 1541
zma-miR397b-5p	GRMZM2G153292_T03	TUBA2	Tubulin alpha-1 chain [Source:UniProtKB/Swiss-Prot;Acc:P14640]	4	18.932	Translation	miRNA 20 UAGUUGCGACGCGAGUUACU 1<br>.::...:::: :.::.::::<br>Target 1522 GUCGGUGCUGAGUUCGAUGA 1541
zma-miR397b-5p	GRMZM2G153292_T04	TUBA2	Tubulin alpha-1 chain [Source:UniProtKB/Swiss-Prot;Acc:P14640]	4	18.932	Translation	miRNA 20 UAGUUGCGACGCGAGUUACU 1<br>.::...:::: :.::.::::<br>Target 1444 GUCGGUGCUGAGUUCGAUGA 1463

Potential targets of zma-miRNAs.

miRNA	Target	Gene name	Gene description	Expectation	UPE	Inhibition	Hybrid
zma-miR397b-5p	GRMZM2G153292_T05	TUBA2	Tubulin alpha-1 chain [Source:UniProtKB/Swiss-Prot;Acc:P14640]	4	15.748	Translation	miRNA 20 UAGUUGCGACGCGAGUUACU 1<br>.:...:::: :.::.::::<br>Target 1441 GUCGGUGCUGAGUUCGAUGA 1460
zma-miR397b-5p	GRMZM2G154245_T02	NA	Putative uncharacterized protein [Source:UniProtKB/TrEMBL;Acc:B6U9R3]	4	22.024	Translation	miRNA 21 GUAGUUGCGACGCGAGUUACU 1<br>:: ::: ::::: .::.::::<br>Target 69 CACCAAGGCUGCCUUCGAUGA 89
zma-miR397b-5p	GRMZM2G159732_T02	NA	Uncharacterized protein [Source:UniProtKB/TrEMBL;Acc:C0HHN4]	4	16.354	Translation	miRNA 21 GUAGUUGCGACGCGAGUUACU 1<br>.::.::.:::: .::: :::<br>Target 800 UAUUAAUGCUGCCUUCACUGA 820
zma-miR397b-5p	GRMZM2G180990_T01	NA	pseudouridine synthase and archaeosine transglycosylase (PUA) domain-containing protein [Source:Projected from Arabidopsis thaliana (AT1G09150) TAIR;Acc:AT1G09150]	4	15.508	Cleavage	miRNA 21 GUAGUUGCGACGCGAGUUACU 1<br>.:: ::.::::.:.:::.::<br>Target 1042 UAUGAAUGCUGUGUUCAGUGC 1062
zma-miR397b-5p	GRMZM2G180990_T02	NA	pseudouridine synthase and archaeosine transglycosylase (PUA) domain-containing protein [Source:Projected from Arabidopsis thaliana (AT1G09150) TAIR;Acc:AT1G09150]	4	15.508	Cleavage	miRNA 21 GUAGUUGCGACGCGAGUUACU 1<br>.:: ::.::::.:.:::.::<br>Target 1306 UAUGAAUGCUGUGUUCAGUGC 1326
zma-miR397b-5p	GRMZM2G400390_T01	NA	NA	4	19.702	Cleavage	miRNA 21 GUAGUUGCGACGCGAGUUACU 1<br>.::::::::::::::::::<br>Target 645 UAUCAACGCUGCGCUCAACAC 665
zma-miR397b-5p	GRMZM2G415327_T04	NA	Uncharacterized protein [Source:UniProtKB/TrEMBL;Acc:B4FKI8]	4	18.005	Cleavage	miRNA 21 GUAGUUGCGACGCGAGUUACU 1<br>..:::: ::::.:.::.:::<br>Target 59 UGUCAAAGCUGUGUUCGAUGC 79
zma-miR397b-5p	GRMZM2G419782_T01	NA	Acyl-coenzyme A oxidase [Source:UniProtKB/TrEMBL;Acc:K7U8E4]	4	24.368	Cleavage	miRNA 21 GUAGUUGCGACGCGAGUUACU 1<br>:: :::.:::::::. :::.<br>Target 624 CAGCAAUGCUGCGCUUCAUGG 644
zma-miR397b-5p	GRMZM2G419782_T02	NA	Acyl-coenzyme A oxidase [Source:UniProtKB/TrEMBL;Acc:K7U8E4]	4	24.368	Cleavage	miRNA 21 GUAGUUGCGACGCGAGUUACU 1<br>:: :::.:::::::. :::.<br>Target 654 CAGCAAUGCUGCGCUUCAUGG 674
zma-miR397b-5p	GRMZM2G445999_T01	NA	NA	4	21.799	Cleavage	miRNA 21 GUAGUUGCGACGCGAGUUACU 1<br>.: ::.::::.: :..::::<br>Target 123 UAGCAGCGCUGUGGUUGAUGA 143
zma-miR397b-5p	GRMZM5G875238_T01	SPS	Sucrose-phosphate synthase [Source:UniProtKB/Swiss-Prot;Acc:P31927]	4	19.379	Translation	miRNA 21 GUAGUUGCGACGCGAGUUACU 1<br>::::::: :::: :::. :::<br>Target 1847 CAUCAACCCUGCUCUCGUUGA 1867
zma-miR397b-5p	AC185265.3_FGT003	NA	NA	4.5	12.878	Cleavage	miRNA 20 UAGUUGCGACGCGAGUUACU 1<br>. ::::.:::: :::::::<br>Target 1222 GCAAACGUUGCGAUCAAUGA 1241
zma-miR397b-5p	AC185443.3_FGT004	NA	Uncharacterized protein [Source:UniProtKB/TrEMBL;Acc:K7UQ31]	4.5	12.354	Cleavage	miRNA 20 UAGUUGCGACGCGAGUUACU 1<br>. ::::.:::: :::::::<br>Target 1222 GCAAACGUUGCGAUCAAUGA 1241
zma-miR397b-5p	AC208212.3_FGT001	NA	NA	4.5	18.221	Cleavage	miRNA 21 GUAGUUGCGACGCGAGUUACU 1<br>..:: ::::::.:::::: :<br>Target 99 UGUCUACGCUGUGCUCAAGGU 119
zma-miR397b-5p	GRMZM2G010034_T01	NA	Putative uncharacterized protein [Source:UniProtKB/TrEMBL;Acc:B6TZN4]	4.5	20.892	Translation	miRNA 21 GUAGUUGCGACGCGAGUUACU 1<br>..:.::.::: ::: :.::::<br>Target 431 UGUUAAUGCUUCGCCCGAUGA 451

Potential targets of zma-miRNAs.

miRNA	Target	Gene name	Gene description	Expectation	UPE	Inhibition	Hybrid
zma-miR397b-5p	GRMZM2G010034_T02	NA	Putative uncharacterized protein [Source:UniProtKB/TrEMBL;Acc:B6TZN4]	4.5	20.892	Translation	miRNA 21 GUAGUUGCGACGCGAGUUACU 1<br>..:.::.::: ::: :.::::<br>Target 431 UGUUAAUGCUUCGCCCGAUGA 451
zma-miR397b-5p	GRMZM2G010034_T03	NA	Putative uncharacterized protein [Source:UniProtKB/TrEMBL;Acc:B6TZN4]	4.5	20.892	Translation	miRNA 21 GUAGUUGCGACGCGAGUUACU 1<br>..:.::.::: ::: :.::::<br>Target 431 UGUUAAUGCUUCGCCCGAUGA 451
zma-miR397b-5p	GRMZM2G010034_T04	NA	Putative uncharacterized protein [Source:UniProtKB/TrEMBL;Acc:B6TZN4]	4.5	20.892	Translation	miRNA 21 GUAGUUGCGACGCGAGUUACU 1<br>..:.::.::: ::: :.::::<br>Target 500 UGUUAAUGCUUCGCCCGAUGA 520
zma-miR397b-5p	GRMZM2G010034_T05	NA	Putative uncharacterized protein [Source:UniProtKB/TrEMBL;Acc:B6TZN4]	4.5	20.892	Translation	miRNA 21 GUAGUUGCGACGCGAGUUACU 1<br>..:.::.::: ::: :.::::<br>Target 431 UGUUAAUGCUUCGCCCGAUGA 451
zma-miR397b-5p	GRMZM2G010034_T06	NA	Putative uncharacterized protein [Source:UniProtKB/TrEMBL;Acc:B6TZN4]	4.5	17.112	Translation	miRNA 21 GUAGUUGCGACGCGAGUUACU 1<br>..:.::.::: ::: :.::::<br>Target 421 UGUUAAUGCUUCGCCCGAUGA 441
zma-miR397b-5p	GRMZM2G027331_T08	NA	Haloacid dehalogenase-like hydrolase (HAD) superfamily protein [Source:Projected from Arabidopsis thaliana (AT5G45170) TAIR;Acc:AT5G45170]	4.5	16.932	Translation	miRNA 21 GUAGUUGCGACGCGAGUUACU 1<br>:::..::: ::: ::...:::<br>Target 714 CAUUGACGAUGCACUUGGUGA 734
zma-miR397b-5p	GRMZM2G037343_T01	NA	Uncharacterized protein [Source:UniProtKB/TrEMBL;Acc:K7V5P7]	4.5	24.256	Cleavage	miRNA 21 GUAGUUGCGACGCGAGUUACU 1<br>:.::..::: :.::::..::.<br>Target 1892 CGUCGGCGCCGUGCUCGGUGG 1912
zma-miR397b-5p	GRMZM2G068024_T02	NA	NA	4.5	10.373	Translation	miRNA 21 GUAGUUGCGACGCGAGUUACU 1<br>.::.::.::: .:.::::: :<br>Target 115 UAUUAAUGCUAUGUUCAAUCA 135
zma-miR397b-5p	GRMZM2G083763_T01	NA	Putative uncharacterized protein [Source:UniProtKB/TrEMBL;Acc:B6TGY4]	4.5	16.491	Translation	miRNA 21 GUAGUUGCGACGCGAGUUACU 1<br>::::.:.::::: .:. ::::<br>Target 728 CAUCGAUGCUGCAUUUCAUGA 748
zma-miR397b-5p	GRMZM2G088112_T01	NA	Uncharacterized protein [Source:UniProtKB/TrEMBL;Acc:K7V9Z3]	4.5	18.185	Translation	miRNA 21 GUAGUUGCGACGCGAGUUACU 1<br>::::::: .::: : :.::::<br>Target 906 CAUCAACAUUGCACCCGAUGA 926
zma-miR397b-5p	GRMZM2G099666_T01	NA	Acyl-coenzyme A oxidase [Source:UniProtKB/TrEMBL;Acc:K7TR99]	4.5	18.711	Cleavage	miRNA 21 GUAGUUGCGACGCGAGUUACU 1<br>:. :::.::::::::. :::.<br>Target 767 CGGCAAUGCUGCGCUUCAUGG 787
zma-miR397b-5p	GRMZM2G109268_T03	NA	NA	4.5	13.796	Translation	miRNA 21 GUAGUUGCGACGCGAGUUACU 1<br>: ::::::.::: ::..:::<br>Target 817 CCUCAACGUUGCUCUUGAUGC 837
zma-miR397b-5p	GRMZM2G112672_T01	NA	Calcineurin B-like protein [Source:UniProtKB/TrEMBL;Acc:C3TRS0]	4.5	20.802	Cleavage	miRNA 21 GUAGUUGCGACGCGAGUUACU 1<br>:: ::..::::.: :..::::<br>Target 198 CAGCAGUGCUGUGAUUGAUGA 218
zma-miR397b-5p	GRMZM2G112672_T02	NA	Calcineurin B-like protein [Source:UniProtKB/TrEMBL;Acc:C3TRS0]	4.5	20.802	Cleavage	miRNA 21 GUAGUUGCGACGCGAGUUACU 1<br>:: ::..::::.: :..::::<br>Target 198 CAGCAGUGCUGUGAUUGAUGA 218
zma-miR397b-5p	GRMZM2G112728_T05	NA	Uncharacterized protein [Source:UniProtKB/TrEMBL;Acc:B4FAH8]	4.5	17.173	Translation	miRNA 21 GUAGUUGCGACGCGAGUUACU 1<br>:::..:. ::: :.:::.:::<br>Target 1212 CAUUGAUUCUGAGUUCAGUGA 1232

Potential targets of zma-miRNAs.

miRNA	Target	Gene name	Gene description	Expectation	UPE	Inhibition	Hybrid
zma-miR397b-5p	GRMZM2G115077_T01	NA	Uncharacterized protein [Source:UniProtKB/TrEMBL;Acc:K7TZ18]	4.5	13.518	Translation	miRNA 21 GUAGUUGCGACGCGAGUUACU 1<br>:::..:. ::: :.:::.:::<br>Target 411 CAUUGAUUCUGAGUUCAGUGA 431
zma-miR397b-5p	GRMZM2G124872_T01	NA	O-fucosyltransferase family protein [Source:Projected from Arabidopsis thaliana (AT1G29200) TAIR;Acc:AT1G29200]	4.5	20.049	Translation	miRNA 21 GUAGUUGCGACGCGAGUUACU 1<br>:.::. :::: .::::::::<br>Target 860 CGUCGCCGCUCUGCUCAAUGC 880
zma-miR397b-5p	GRMZM2G135817_T01	NA	NA	4.5	21.078	Cleavage	miRNA 20 UAGUUGCGACGCGAGUUACU 1<br>::::.:::::::.:..: ::<br>Target 1949 UUCAAUGCUGCGUUUGAGGA 1968
zma-miR397b-5p	GRMZM2G153404_T01	NA	NA	4.5	21.219	Cleavage	miRNA 21 GUAGUUGCGACGCGAGUUACU 1<br>:. ::: ::::::.: :::::<br>Target 408 CGCCAAAGCUGCGUUAAAUGA 428
zma-miR397b-5p	GRMZM2G310758_T01	NA	Uncharacterized protein [Source:UniProtKB/TrEMBL;Acc:K7UVA1]	4.5	16.722	Cleavage	miRNA 21 GUAGUUGCG-ACGCGAGUUACU 1<br>::::::.:: ::.:.:.::::.<br>Target 870 CAUCAAUGCAUGUGUUUAAUGG 891
zma-miR397b-5p	GRMZM2G341083_T01	NA	Uncharacterized protein [Source:UniProtKB/TrEMBL;Acc:K7TZ86]	4.5	24.269	Translation	miRNA 21 GUAGUUGCGACGCGAGUUACU 1<br>..::.:::::: :::.. :::<br>Target 149 UGUCGACGCUGAGCUUGUUGA 169
zma-miR397b-5p	GRMZM2G445791_T01	NA	Ribosomal protein L15 [Source:UniProtKB/TrEMBL;Acc:B6SWX0]	4.5	23.67	Cleavage	miRNA 21 GUAGUUGCGACGCGAGUUACU 1<br>:. :::.:::::::. :::.<br>Target 624 CGGCAAUGCUGCGCUUCAUGG 644
zma-miR397b-5p	GRMZM2G445791_T02	NA	Ribosomal protein L15 [Source:UniProtKB/TrEMBL;Acc:B6SWX0]	4.5	23.67	Cleavage	miRNA 21 GUAGUUGCGACGCGAGUUACU 1<br>:. :::.:::::::. :::.<br>Target 654 CGGCAAUGCUGCGCUUCAUGG 674
zma-miR397b-5p	GRMZM5G820287_T01	NA	NA	4.5	21.465	Cleavage	miRNA 21 GUAGUUGCGACGCGAGUUACU 1<br>:. :::.:::::::. :::.<br>Target 618 CGGCAAUGCUGCGCUUCAUGG 638
zma-miR397b-5p	GRMZM5G820287_T02	NA	NA	4.5	21.465	Cleavage	miRNA 21 GUAGUUGCGACGCGAGUUACU 1<br>:. :::.:::::::. :::.<br>Target 618 CGGCAAUGCUGCGCUUCAUGG 638
zma-miR397b-5p	GRMZM5G825834_T01	NA	NA	4.5	18.766	Translation	miRNA 21 GUAGUUGCGACGCGAGUUACU 1<br>:.:..:.::::: :::: :::<br>Target 534 CGUUGAUGCUGCACUCAUUGA 554
zma-miR397b-5p	AC203101.3_FGT002	NA	Uncharacterized protein [Source:UniProtKB/TrEMBL;Acc:K7UQA3]	5	19.305	Cleavage	miRNA 21 GUAGUUGCGACGCGAGUUACU 1<br>:::..:.: ::.::::.: ::<br>Target 330 CAUUGAUGGUGUGCUCGAGGA 350
zma-miR397b-5p	AC233854.1_FGT002	NA	Uncharacterized protein [Source:UniProtKB/TrEMBL;Acc:K7VDX3]	5	19.536	Cleavage	miRNA 21 GUAGUUGCGACGCGAGUUACU 1<br>:: ..:.:.::.:::: ::::<br>Target 1161 CACUGAUGUUGUGCUCCAUGA 1181
zma-miR397b-5p	GRMZM2G019859_T01	NA	Uncharacterized protein [Source:UniProtKB/TrEMBL;Acc:K7VCK2]	5	22.537	Cleavage	miRNA 20 UAGUUGCGACGCGAGUUACU 1<br>:::::.::.:.::.:::<br>Target 1387 UACAACGUUGUGUUCGAUGU 1406
zma-miR397b-5p	GRMZM2G051417_T01	NA	Uncharacterized protein [Source:UniProtKB/TrEMBL;Acc:K7U8B3]	5	20.049	Translation	miRNA 21 GUAGUUGCGACGCGAGUUACU 1<br>:.::::.::: ::::: .::<br>Target 611 CGUCAAUGCUCCGCUCCGUGC 631

Potential targets of zma-miRNAs.

miRNA	Target	Gene name	Gene description	Expectation	UPE	Inhibition	Hybrid
zma-miR397b-5p	GRMZM2G094375_T01	NA	Uncharacterized protein [Source:UniProtKB/TrEMBL;Acc:K7TF84]	5	20.244	Cleavage	miRNA 21 GUAGUUGCGACGCGAGUUACU 1<br>::::::::: ::::::::<br>Target 806 CAUCAACGCCGCGCUCAACAC 826
zma-miR397b-5p	GRMZM2G094375_T02	NA	Uncharacterized protein [Source:UniProtKB/TrEMBL;Acc:K7TF84]	5	20.244	Cleavage	miRNA 21 GUAGUUGCGACGCGAGUUACU 1<br>::::::::: ::::::::<br>Target 949 CAUCAACGCCGCGCUCAACAC 969
zma-miR397b-5p	GRMZM2G094375_T03	NA	Uncharacterized protein [Source:UniProtKB/TrEMBL;Acc:K7TF84]	5	23.694	Cleavage	miRNA 21 GUAGUUGCGACGCGAGUUACU 1<br>::::::::: ::::::::<br>Target 897 CAUCAACGCCGCGCUCAACAC 917
zma-miR397b-5p	GRMZM2G106807_T01	NA	NA	5	20.052	Cleavage	miRNA 21 GUAGUUGCGACGCGAGUUACU 1<br>:: ..:.:.::.:::: ::::<br>Target 723 CACUGAUGUUGUGCUCCAUGA 743
zma-miR397b-5p	GRMZM2G108859_T01	NA	NA	5	21.964	Cleavage	miRNA 21 GUAGUUGCGACGCGAGUUACU 1<br>: ::::::. ::::::::: .<br>Target 324 CUUCAACGUCGCGCUCAAUCG 344
zma-miR397b-5p	GRMZM2G111112_T01	NA	Putative uncharacterized protein [Source:UniProtKB/TrEMBL;Acc:B6SHN3]	5	20.476	Cleavage	miRNA 21 GUAGUUGCGACGCGAGUUACU 1<br>::::..::. :.::: .::::<br>Target 327 CAUCGGCGUCGUGCUGGAUGA 347
zma-miR397b-5p	GRMZM2G111112_T02	NA	Putative uncharacterized protein [Source:UniProtKB/TrEMBL;Acc:B6SHN3]	5	20.476	Cleavage	miRNA 21 GUAGUUGCGACGCGAGUUACU 1<br>::::..::. :.::: .::::<br>Target 201 CAUCGGCGUCGUGCUGGAUGA 221
zma-miR397b-5p	GRMZM2G132169_T01	NA	Uncharacterized protein [Source:UniProtKB/TrEMBL;Acc:C0P4F2]	5	24.778	Cleavage	miRNA 21 GUAGUUGCGACGCGAGUUACU 1<br>::::::::: :::::::: :<br>Target 776 CAUCAACGCCGCGCUCAACCA 796
zma-miR397b-5p	GRMZM2G132169_T02	NA	Uncharacterized protein [Source:UniProtKB/TrEMBL;Acc:C0P4F2]	5	24.778	Cleavage	miRNA 21 GUAGUUGCGACGCGAGUUACU 1<br>::::::::: :::::::: :<br>Target 918 CAUCAACGCCGCGCUCAACCA 938
zma-miR397b-5p	GRMZM2G134806_T01	NA	HSP70-interacting protein 1 [Source:Projected from Arabidopsis thaliana (AT4G22670) TAIR;Acc:AT4G22670]	5	18.515	Cleavage	miRNA 20 UAGUUGCGACGCGAGUUACU 1<br>:::::.::::.:::::: :<br>Target 796 AUCAAUGCUGUGCUCAAGAA 815
zma-miR397b-5p	GRMZM2G336337_T01	NA	Putative laccase family protein; Uncharacterized protein [Source:UniProtKB/TrEMBL;Acc:C0P5Q0]	5	22.647	Cleavage	miRNA 21 GUAGUUGCGACGCGAGUUACU 1<br>::::::::: :::::::: :<br>Target 926 CAUCAACGCCGCGCUCAACCA 946
zma-miR397b-5p	GRMZM2G359779_T01	NA	NA	5	21.964	Cleavage	miRNA 21 GUAGUUGCGACGCGAGUUACU 1<br>: ::::::. ::::::::: .<br>Target 321 CUUCAACGUCGCGCUCAAUCG 341
zma-miR397b-5p	GRMZM2G383210_T01	NA	Putative jumonji-like transcription factor family protein; Uncharacterized protein [Source:UniProtKB/TrEMBL;Acc:B8A2P9]	5	12.886	Cleavage	miRNA 21 GUAGUUGCGACGCGAGUUACU 1<br>:::::: :.::::::..::<br>Target 3196 CAUCAAAGUUGCGCUUGAUUU 3216
zma-miR397b-5p	GRMZM2G383210_T02	NA	Putative jumonji-like transcription factor family protein; Uncharacterized protein [Source:UniProtKB/TrEMBL;Acc:B8A2P9]	5	12.886	Cleavage	miRNA 21 GUAGUUGCGACGCGAGUUACU 1<br>:::::: :.::::::..::<br>Target 3153 CAUCAAAGUUGCGCUUGAUUU 3173
zma-miR397b-5p	GRMZM2G383210_T03	NA	Putative jumonji-like transcription factor family protein; Uncharacterized protein [Source:UniProtKB/TrEMBL;Acc:B8A2P9]	5	12.886	Cleavage	miRNA 21 GUAGUUGCGACGCGAGUUACU 1<br>:::::: :.::::::..::<br>Target 1639 CAUCAAAGUUGCGCUUGAUUU 1659

Potential targets of zma-miRNAs.

miRNA	Target	Gene name	Gene description	Expectation	UPE	Inhibition	Hybrid
zma-miR397b-5p	GRMZM2G383210_T04	NA	Putative jumonji-like transcription factor family protein; Uncharacterized protein [Source:UniProtKB/TrEMBL;Acc:B8A2P9]	5	12.886	Cleavage	miRNA 21 GUAGUUGCGACGCGAGUUACU 1<br>:::::: :.::::::..::<br>Target 1639 CAUCAAAGUUGCGCUUGAUUU 1659
zma-miR397b-5p	GRMZM2G388587_T01	NA	Putative laccase family protein [Source:UniProtKB/TrEMBL;Acc:K7UAF3]	5	23.649	Cleavage	miRNA 21 GUAGUUGCGACGCGAGUUACU 1<br>::::::::: ::::::::<br>Target 825 CAUCAACGCCGCGCUCAACAC 845
zma-miR397b-5p	GRMZM2G408881_T01	NA	Uncharacterized protein [Source:UniProtKB/TrEMBL;Acc:K7VTL4]	5	18.125	Cleavage	miRNA 21 GUAGUUGCGACGCGAGUUACU 1<br>:: ..:.:.::.:::: ::::<br>Target 1206 CACUGAUGUUGUGCUCCAUGA 1226
zma-miR398b-3p	GRMZM2G068710_T07	NA	Uncharacterized protein [Source:UniProtKB/TrEMBL;Acc:B4FMJ6]	3	18.306	Cleavage	miRNA 21 GCCCCCGCUGGACUCUUGUGU 1<br>:: :: :::::::::.::.::<br>Target 1389 CGUGGCCGACCUGAGGACGCA 1409
zma-miR398b-3p	GRMZM2G068710_T08	NA	Uncharacterized protein [Source:UniProtKB/TrEMBL;Acc:B4FMJ6]	3	18.306	Cleavage	miRNA 21 GCCCCCGCUGGACUCUUGUGU 1<br>:: :: :::::::::.::.::<br>Target 1336 CGUGGCCGACCUGAGGACGCA 1356
zma-miR398b-3p	GRMZM2G338696_T01	NA	Uncharacterized protein [Source:UniProtKB/TrEMBL;Acc:C0PG03]	3	19.997	Translation	miRNA 20 CCCCCGCUGGACUCUUGUGU 1<br>::::::::: ::::::::<br>Target 52 AGGGGCGACCCGAGAACACC 71
zma-miR398b-3p	GRMZM2G338696_T02	NA	Uncharacterized protein [Source:UniProtKB/TrEMBL;Acc:C0PG03]	3	19.997	Translation	miRNA 20 CCCCCGCUGGACUCUUGUGU 1<br>::::::::: ::::::::<br>Target 52 AGGGGCGACCCGAGAACACC 71
zma-miR398b-3p	GRMZM2G338696_T03	NA	Uncharacterized protein [Source:UniProtKB/TrEMBL;Acc:C0PG03]	3	19.997	Translation	miRNA 20 CCCCCGCUGGACUCUUGUGU 1<br>::::::::: ::::::::<br>Target 52 AGGGGCGACCCGAGAACACC 71
zma-miR398b-3p	GRMZM2G338696_T04	NA	Uncharacterized protein [Source:UniProtKB/TrEMBL;Acc:C0PG03]	3	19.997	Translation	miRNA 20 CCCCCGCUGGACUCUUGUGU 1<br>::::::::: ::::::::<br>Target 52 AGGGGCGACCCGAGAACACC 71
zma-miR398b-3p	GRMZM2G338696_T05	NA	Uncharacterized protein [Source:UniProtKB/TrEMBL;Acc:C0PG03]	3	19.997	Translation	miRNA 20 CCCCCGCUGGACUCUUGUGU 1<br>::::::::: ::::::::<br>Target 52 AGGGGCGACCCGAGAACACC 71
zma-miR398b-3p	GRMZM2G338696_T06	NA	Uncharacterized protein [Source:UniProtKB/TrEMBL;Acc:C0PG03]	3	19.997	Translation	miRNA 20 CCCCCGCUGGACUCUUGUGU 1<br>::::::::: ::::::::<br>Target 52 AGGGGCGACCCGAGAACACC 71
zma-miR398b-3p	GRMZM2G025992_T02	SODCC.1	Superoxide dismutase [Cu-Zn] 2 [Source:UniProtKB/Swiss-Prot;Acc:P11428]	3.5	20.794	Cleavage	miRNA 20 CCCCCGCUGGACUCUUGUGU 1<br>:::::. .::::::: ::::<br>Target 186 GGGGGUCGCCUGAGAUCACA 205
zma-miR398b-3p	GRMZM2G025992_T04	SODCC.1	Superoxide dismutase [Cu-Zn] 2 [Source:UniProtKB/Swiss-Prot;Acc:P11428]	3.5	20.639	Cleavage	miRNA 20 CCCCCGCUGGACUCUUGUGU 1<br>:::::. .::::::: ::::<br>Target 186 GGGGGUCGCCUGAGAUCACA 205
zma-miR398b-3p	GRMZM2G058522_T01	SODCC.2<br>SOD9	Superoxide dismutase [Cu-Zn] 4AP [Source:UniProtKB/Swiss-Prot;Acc:P23346]	3.5	17.156	Cleavage	miRNA 21 GCCCCCGCUGGACUCUUGUGU 1<br>::::::. .::::::: ::::<br>Target 233 CGGGGGUCGCCUGAGAUCACA 253
zma-miR398b-3p	GRMZM2G058522_T02	SODCC.2<br>SOD9	Superoxide dismutase [Cu-Zn] 4AP [Source:UniProtKB/Swiss-Prot;Acc:P23346]	3.5	17.156	Cleavage	miRNA 21 GCCCCCGCUGGACUCUUGUGU 1<br>::::::. .::::::: ::::<br>Target 233 CGGGGGUCGCCUGAGAUCACA 253

miRNA	Target	Gene name	Gene description	Expectation	UPE	Inhibition	Hybrid
zma-miR398b-3p	GRMZM2G058522_T03	SODCC.2<br>SOD9	Superoxide dismutase [Cu-Zn] 4AP [Source:UniProtKB/Swiss-Prot;Acc:P23346]	3.5	17.156	Cleavage	miRNA 21 GCCCCCGCUGGACUCUUGUGU 1<br>::::::. .::::::: ::::<br>Target 233 CGGGGGUCGCCUGAGAUCACA 253
zma-miR398b-3p	GRMZM2G058522_T05	SODCC.2<br>SOD9	Superoxide dismutase [Cu-Zn] 4AP [Source:UniProtKB/Swiss-Prot;Acc:P23346]	3.5	17.156	Cleavage	miRNA 21 GCCCCCGCUGGACUCUUGUGU 1<br>::::::. .::::::: ::::<br>Target 209 CGGGGGUCGCCUGAGAUCACA 229
zma-miR398b-3p	GRMZM2G073860_T01	NA	Purple acid phosphatase; Uncharacterized protein [Source:UniProtKB/TrEMBL;Acc:B4FLK0]	3.5	18.911	Translation	miRNA 20 CCCCCGCUGGACUCUUGUGU 1<br>:::::.:.:: ::::: :::<br>Target 346 GGGGGUGGCCAGAGAAAACA 365
zma-miR398b-3p	GRMZM2G086497_T01	NA	Uncharacterized protein [Source:UniProtKB/TrEMBL;Acc:B6T9J0]	3.5	18.833	Cleavage	miRNA 20 CCCCCGCUGGACUCUUGUGU 1<br>:: ::::: :::.::.::.:<br>Target 7 GGUGGCGAGCUGGGAGCAUA 26
zma-miR398b-3p	GRMZM2G105307_T01	NA	HIT-type Zinc finger family protein [Source:Projected from Arabidopsis thaliana (AT4G28820) TAIR;Acc:AT4G28820]	3.5	23.32	Translation	miRNA 21 GCCCCCGCUGGACUCUUGUGU 1<br>.:::: :::.: ::::::::<br>Target 811 UGGGGUAGACUUUAGAACACA 831
zma-miR398b-3p	GRMZM2G105307_T03	NA	HIT-type Zinc finger family protein [Source:Projected from Arabidopsis thaliana (AT4G28820) TAIR;Acc:AT4G28820]	3.5	23.32	Translation	miRNA 21 GCCCCCGCUGGACUCUUGUGU 1<br>.:::: :::.: ::::::::<br>Target 507 UGGGGUAGACUUUAGAACACA 527
zma-miR398b-3p	GRMZM2G119627_T01	NA	Uncharacterized protein [Source:UniProtKB/TrEMBL;Acc:B4FT69]	3.5	22.336	Cleavage	miRNA 20 CCCCCGCUGGACUCUUGUGU 1<br>::: ::.::::::::..::<br>Target 1540 UGGGUCGGCCUGAGAAUGCA 1559
zma-miR398b-3p	GRMZM2G169890_T01	SODCC.3	Superoxide dismutase [Cu-Zn] 4A [Source:UniProtKB/Swiss-Prot;Acc:P23345]	3.5	19.066	Cleavage	miRNA 21 GCCCCCGCUGGACUCUUGUGU 1<br>::::::. .::::::: ::::<br>Target 214 CGGGGGUCGCCUGAGAUCACA 234
zma-miR398b-3p	GRMZM2G169890_T03	SODCC.3	Superoxide dismutase [Cu-Zn] 4A [Source:UniProtKB/Swiss-Prot;Acc:P23345]	3.5	19.066	Cleavage	miRNA 21 GCCCCCGCUGGACUCUUGUGU 1<br>::::::. .::::::: ::::<br>Target 198 CGGGGGUCGCCUGAGAUCACA 218
zma-miR398b-3p	GRMZM2G169890_T04	SODCC.3	Superoxide dismutase [Cu-Zn] 4A [Source:UniProtKB/Swiss-Prot;Acc:P23345]	3.5	19.066	Cleavage	miRNA 21 GCCCCCGCUGGACUCUUGUGU 1<br>::::::. .::::::: ::::<br>Target 198 CGGGGGUCGCCUGAGAUCACA 218
zma-miR398b-3p	AC197377.3_FGT004	NA	Uncharacterized protein [Source:UniProtKB/TrEMBL;Acc:K7TXZ1]	4	24.938	Translation	miRNA 21 GCCCCCGCUGGACUCUUGUGU 1<br>::: :::::: :.::.::::<br>Target 281 CGGUGGCGACGCGGGAGCACA 301
zma-miR398b-3p	AC203408.4_FGT003	NA	Uncharacterized protein [Source:UniProtKB/TrEMBL;Acc:K7V9T9]	4	21.03	Cleavage	miRNA 21 GCCCCCGCUGGACUCUUGUGU 1<br>.:: :::.:::::.:::..<br>Target 187 UGGCAGCGGCCUGAGGACAUG 207
zma-miR398b-3p	GRMZM2G091535_T02	NA	NA	4	22.53	Translation	miRNA 20 CCCCCGCUGGACUCUUGUGU 1<br>::::::::: ::.::. ::.<br>Target 486 GGGGGCGACGUGGGAGGACG 505
zma-miR398b-3p	GRMZM2G140095_T01	NA	Protein kinase APK1A; Uncharacterized protein [Source:UniProtKB/TrEMBL;Acc:B6TEK0]	4	24.657	Cleavage	miRNA 20 CCCCCGCUGGACUCUUGUGU 1<br>::: :: :.:::::.::::<br>Target 1033 AGGGCCGCCUUGAGAGCACA 1052
zma-miR398b-3p	GRMZM2G140095_T02	NA	Protein kinase APK1A; Uncharacterized protein [Source:UniProtKB/TrEMBL;Acc:B6TEK0]	4	24.657	Cleavage	miRNA 20 CCCCCGCUGGACUCUUGUGU 1<br>::: :: :.:::::.::::<br>Target 658 AGGGCCGCCUUGAGAGCACA 677

miRNA	Target	Gene name	Gene description	Expectation	UPE	Inhibition	Hybrid
zma-miR398b-3p	GRMZM2G140095_T03	NA	Protein kinase APK1A; Uncharacterized protein [Source:UniProtKB/TrEMBL;Acc:B6TEK0]	4	22.919	Cleavage	miRNA 20 CCCCCGCUGGACUCUUGUGU 1<br>::: :: :.:::::.::::<br>Target 658 AGGGCCGCCUUGAGAGCACA 677
zma-miR398b-3p	GRMZM2G140095_T04	NA	Protein kinase APK1A; Uncharacterized protein [Source:UniProtKB/TrEMBL;Acc:B6TEK0]	4	24.657	Cleavage	miRNA 20 CCCCCGCUGGACUCUUGUGU 1<br>::: :: :.:::::.::::<br>Target 312 AGGGCCGCCUUGAGAGCACA 331
zma-miR398b-3p	GRMZM2G331566_T02	NA	Uncharacterized protein [Source:UniProtKB/TrEMBL;Acc:B4FVM4]	4	23.545	Translation	miRNA 21 GCCCCCGCUGGACUCUUGUGU 1<br>.::: :::.:: :::.:::..<br>Target 756 UGGGAGCGGCCGGAGGACAUG 776
zma-miR398b-3p	GRMZM2G405815_T02	NA	Ribonuclease II/R family protein [Source:Projected from Arabidopsis thaliana (AT1G77680) TAIR;Acc:AT1G77680]	4	21.587	Translation	miRNA 21 GCCCCCGCUGGACUCUUGUGU 1<br>.: :::: ::: :.::.::::<br>Target 2 UGAGGGCCACCGGGGAGCACA 22
zma-miR398b-3p	GRMZM2G474367_T01	NA	NA	4	21.242	Translation	miRNA 21 GCCCCCGCUGGACUCUUGUGU 1<br>::: :::::: :.::.::::<br>Target 137 CGGUGGCGACGCGGGAGCACA 157
zma-miR398b-3p	GRMZM2G474367_T02	NA	NA	4	21.82	Translation	miRNA 21 GCCCCCGCUGGACUCUUGUGU 1<br>::: :::::: :.::.::::<br>Target 114 CGGUGGCGACGCGGGAGCACA 134
zma-miR398b-3p	GRMZM2G478238_T01	NA	Uncharacterized protein [Source:UniProtKB/TrEMBL;Acc:K7TU71]	4	23.011	Translation	miRNA 21 GCCCCCGCUGGACUCUUGUGU 1<br>::: :::::: :.::.::::<br>Target 708 CGGUGGCGACGCGGGAGCACA 728
zma-miR398b-3p	AC217270.3_FGT003	NA	Uncharacterized protein [Source:UniProtKB/TrEMBL;Acc:K7UU15]	4.5	19.816	Cleavage	miRNA 20 CCCCCGCUGGACUCUUGUGU 1<br>:::::: :::.::::::<br>Target 134 UCGGGCGAGCUGGGAACACU 153
zma-miR398b-3p	AC217270.3_FGT004	NA	Uncharacterized protein [Source:UniProtKB/TrEMBL;Acc:K7TYX3]	4.5	19.816	Cleavage	miRNA 20 CCCCCGCUGGACUCUUGUGU 1<br>:::::: :::.::::::<br>Target 134 UCGGGCGAGCUGGGAACACU 153
zma-miR398b-3p	GRMZM2G019266_T01	NA	NA	4.5	21.123	Cleavage	miRNA 20 CCCCCGCUGGACUCUUGUGU 1<br>::::: ...::.:.:::::<br>Target 279 UGGGGCAGUUUGGGGACACA 298
zma-miR398b-3p	GRMZM2G019266_T02	NA	NA	4.5	21.123	Cleavage	miRNA 20 CCCCCGCUGGACUCUUGUGU 1<br>::::: ...::.:.:::::<br>Target 201 UGGGGCAGUUUGGGGACACA 220
zma-miR398b-3p	GRMZM2G019266_T03	NA	NA	4.5	21.123	Cleavage	miRNA 20 CCCCCGCUGGACUCUUGUGU 1<br>::::: ...::.:.:::::<br>Target 279 UGGGGCAGUUUGGGGACACA 298
zma-miR398b-3p	GRMZM2G026952_T01	NA	Uncharacterized protein [Source:UniProtKB/TrEMBL;Acc:K7VY75]	4.5	24.105	Translation	miRNA 21 GCCCCCGCUGGACUCUUGUGU 1<br>.:::::::::. ::::.::<br>Target 1598 UGGGGGCGACUGGAGAGCAAC 1618
zma-miR398b-3p	GRMZM2G030659_T01	NA	Putative DEAD-box ATP-dependent RNA helicase family protein [Source:UniProtKB/TrEMBL;Acc:K7THT7]	4.5	17.937	Cleavage	miRNA 20 CCCCCGCUGGACUCUUGUGU 1<br>:: ::.::.:::. :.::::<br>Target 2851 GGAGGUGAUCUGGCAGCACA 2870
zma-miR398b-3p	GRMZM2G039385_T04	NA	Phosphatidylserine synthase 2; Uncharacterized protein [Source:UniProtKB/TrEMBL;Acc:B6TFK2]	4.5	18.886	Cleavage	miRNA 21 GCCCCCGC-UGGACUCUUGUGU 1<br>::: :::: ::::::::.:::<br>Target 2175 CGGUGGCGCACCUGAGAGCACC 2196

Potential targets of zma-miRNAs.

miRNA	Target	Gene name	Gene description	Expectation	UPE	Inhibition	Hybrid
zma-miR398b-3p	GRMZM2G082191_T01	NA	NA	4.5	19.002	Cleavage	miRNA 20 CCCCCGCUGGACUCUUGUGU 1<br>:: :::::.:::. ::::..<br>Target 4025 GGAGGCGAUCUGGUAACAUG 4044
zma-miR398b-3p	GRMZM2G089365_T01	NA	Fructose-bisphosphate aldolase [Source:UniProtKB/TrEMBL;Acc:B4FV58]	4.5	24.432	Cleavage	miRNA 20 CCCCCGCUGGACUCUUGUGU 1<br>::::: :::::::::...<br>Target 1132 AAGGGCGCCCUGAGAACGUG 1151
zma-miR398b-3p	GRMZM2G089365_T02	NA	Fructose-bisphosphate aldolase [Source:UniProtKB/TrEMBL;Acc:B4FV58]	4.5	24.432	Cleavage	miRNA 20 CCCCCGCUGGACUCUUGUGU 1<br>::::: :::::::::...<br>Target 927 AAGGGCGCCCUGAGAACGUG 946
zma-miR398b-3p	GRMZM2G097457_T01	NA	Pyruvate	4.5	24.585	Cleavage	miRNA 21 GCCCCCGCUGGACUCUUGUGU 1<br>: : :::::.:::.:::::.<br>Target 1905 CAGUGGCGAUCUGGGAACAUU 1925
zma-miR398b-3p	GRMZM2G097457_T02	NA	Pyruvate	4.5	24.585	Cleavage	miRNA 21 GCCCCCGCUGGACUCUUGUGU 1<br>: : :::::.:::.:::::.<br>Target 1645 CAGUGGCGAUCUGGGAACAUU 1665
zma-miR398b-3p	GRMZM2G104833_T01	NA	Non-lysosomal glucosylceramidase [Source:UniProtKB/TrEMBL;Acc:K7TV85]	4.5	20.84	Cleavage	miRNA 21 GCCCCCGCUGGACUCUUGUGU 1<br>: : : .::::::.:::::.:<br>Target 104 CAGUGUUGACCUGGGAACAUA 124
zma-miR398b-3p	GRMZM2G104833_T02	NA	Non-lysosomal glucosylceramidase [Source:UniProtKB/TrEMBL;Acc:K7TV85]	4.5	20.84	Cleavage	miRNA 21 GCCCCCGCUGGACUCUUGUGU 1<br>: : : .::::::.:::::.:<br>Target 104 CAGUGUUGACCUGGGAACAUA 124
zma-miR398b-3p	GRMZM2G148098_T01	NA	NA	4.5	21.204	Cleavage	miRNA 20 CCCCCGCUGGACUCUUGUGU 1<br>:::::: :::::: ::. ::<br>Target 1369 GGGGGCUACCUGAAAAUUCA 1388
zma-miR398b-3p	GRMZM2G157267_T01	NA	Putative inositol polyphosphate phosphatase (Synaptogenin-like) family protein [Source:UniProtKB/TrEMBL;Acc:K7UKR9]	4.5	18.955	Cleavage	miRNA 20 CCCCCGCUGGACUCUUGUGU 1<br>: :.:::.::.:::::::<br>Target 2348 UUGUGUGACUUGGGAACACA 2367
zma-miR398b-3p	GRMZM2G158609_T01	NA	NA	4.5	21.872	Cleavage	miRNA 21 GCCCCCGCUGGACUCUUGUGU 1<br>:: :: .:..:::::..::::<br>Target 2811 CGUGGUUGGUCUGAGGGCACA 2831
zma-miR398b-3p	GRMZM2G169486_T03	NA	Peroxin Pex14 [Source:UniProtKB/TrEMBL;Acc:B6TM66]	4.5	17.983	Cleavage	miRNA 21 GCCCCCGCUGGACUCUUGUGU 1<br>:: :: :::.:::. ::::::<br>Target 160 CGUGGUCGAUCUGGAAACACA 180
zma-miR398b-3p	GRMZM2G169486_T04	NA	Peroxin Pex14 [Source:UniProtKB/TrEMBL;Acc:B6TM66]	4.5	17.983	Cleavage	miRNA 21 GCCCCCGCUGGACUCUUGUGU 1<br>:: :: :::.:::. ::::::<br>Target 160 CGUGGUCGAUCUGGAAACACA 180
zma-miR398b-3p	GRMZM5G837538_T01	NA	NA	4.5	24.973	Cleavage	miRNA 20 CCCCCGCUGGACUCUUGUGU 1<br>::: :.:.::::.: ::.::<br>Target 149 GGGCGUGGCCUGGGCACGCA 168
zma-miR398b-3p	AC203257.2_FGT003	NA	NA	5	22.903	Translation	miRNA 21 GCCCCCGCUGGACUCUUGUGU 1<br>.:::::::.:. :::.::: .<br>Target 2 UGGGGGCGGCUGGAGGACAGG 22
zma-miR398b-3p	AC205833.3_FGT002	NA	NA	5	14.49	Translation	miRNA 21 GCCCCCGCUGGACUCUUGUGU 1<br>.:::::::.:. :::.::: .<br>Target 2 UGGGGGCGGCUGGAGGACAGG 22

Potential targets of zma-miRNAs.

miRNA	Target	Gene name	Gene description	Expectation	UPE	Inhibition	Hybrid
zma-miR398b-3p	AC212886.3_FGT006	NA	Uncharacterized protein [Source:UniProtKB/TrEMBL;Acc:K7VTD9]	5	23.686	Cleavage	miRNA 21 GCCCCCGCUGGACUCUUGUGU 1<br>.:: ::. :::::::::::<br>Target 910 UGGCGGUCACCUGAGAACAAC 930
zma-miR398b-3p	GRMZM2G030122_T01	NA	Uncharacterized protein [Source:UniProtKB/TrEMBL;Acc:B4FEC4]	5	23.686	Cleavage	miRNA 21 GCCCCCGCUGGACUCUUGUGU 1<br>.:: ::. :::::::::::<br>Target 1371 UGGCGGUCACCUGAGAACAAC 1391
zma-miR398b-3p	GRMZM2G030122_T02	NA	Uncharacterized protein [Source:UniProtKB/TrEMBL;Acc:B4FEC4]	5	23.686	Cleavage	miRNA 21 GCCCCCGCUGGACUCUUGUGU 1<br>.:: ::. :::::::::::<br>Target 1371 UGGCGGUCACCUGAGAACAAC 1391
zma-miR398b-3p	GRMZM2G042074_T01	NA	NA	5	10.527	Cleavage	miRNA 21 GCCCCCGCUGGACUCUUGUGU 1<br>: ::: .:..::::::::: :<br>Target 2877 CUGGGAUGGUCUGAGAACAAA 2897
zma-miR398b-3p	GRMZM2G042074_T02	NA	NA	5	10.527	Cleavage	miRNA 21 GCCCCCGCUGGACUCUUGUGU 1<br>: ::: .:..::::::::: :<br>Target 2107 CUGGGAUGGUCUGAGAACAAA 2127
zma-miR398b-3p	GRMZM2G059799_T01	NA	Ethylene-responsive protein [Source:UniProtKB/TrEMBL;Acc:B6T3Q3]	5	14.76	Cleavage	miRNA 20 CCCCCGCUGGACUCUUGUGU 1<br>::::::::::::.:: :<br>Target 87 ACAGGCGACCUGAGAGCAGA 106
zma-miR398b-3p	GRMZM2G104925_T01	NA	Leucine-rich repeat (LRR) family protein [Source:Projected from Arabidopsis thaliana (AT1G65380) TAIR;Acc:AT1G65380]	5	17.553	Cleavage	miRNA 20 CCCCCGCUGGACUCUUGUGU 1<br>:::.: .:::::::..::<br>Target 1711 AAGGGUGCUCUGAGAAUGCA 1730
zma-miR398b-3p	GRMZM2G129209_T01	NA	Omega-3 fatty acid desaturase [Source:UniProtKB/TrEMBL;Acc:B6STM5]	5	24.217	Cleavage	miRNA 20 CCCCCGCUGGACUCUUGUGU 1<br>::::.:.::::: :::.:<br>Target 1249 CGGGGUGGCCUGACAACGCU 1268
zma-miR398b-3p	GRMZM2G175728_T01	NA	Copper chaperone for superoxide dismutase [Source:UniProtKB/TrEMBL;Acc:B6TLT7]	5	17.981	Cleavage	miRNA 20 CCCCCGCUGGACUCUUGUGU 1<br>::.::::::.::::::<br>Target 865 CUUGGUGACCUGGGAACACU 884
zma-miR398b-3p	GRMZM2G317610_T01	NA	NA	5	22.803	Cleavage	miRNA 20 CCCCCGCUGGACUCUUGUGU 1<br>::::::.:.:::::....<br>Target 844 AGGGGCGGCUUGAGAGUGUU 863
zma-miR398b-3p	GRMZM2G515667_T01	NA	NA	5	23.876	Cleavage	miRNA 20 CCCCCGCUGGACUCUUGUGU 1<br>:: :::.:::.::::::<br>Target 491 UCGGACGAUCUGGGAACACU 510
zma-miR408a	GRMZM2G023847_T01	NA	Uncharacterized protein [Source:UniProtKB/TrEMBL;Acc:B4FAB0]	2	21.294	Cleavage	miRNA 20 GGUCCCUUCUCCGUCACGUC 1<br>::::: :::::::::::::<br>Target 851 CCAGGAUAGAGGCAGUGCAG 870
zma-miR408a	GRMZM2G000842_T01	NA	Putative bZIP transcription factor superfamily protein [Source:UniProtKB/TrEMBL;Acc:K7UK01]	3	24.267	Cleavage	miRNA 20 GGUCCCUUCUCCGUCACGUC 1<br>:: ::::.::::..::::.:<br>Target 1017 CCCGGGAGGAGGUGGUGCGG 1036
zma-miR408a	GRMZM2G000842_T02	NA	Putative bZIP transcription factor superfamily protein [Source:UniProtKB/TrEMBL;Acc:K7UK01]	3	24.267	Cleavage	miRNA 20 GGUCCCUUCUCCGUCACGUC 1<br>:: ::::.::::..::::.:<br>Target 947 CCCGGGAGGAGGUGGUGCGG 966
zma-miR408a	GRMZM2G000842_T03	NA	Putative bZIP transcription factor superfamily protein [Source:UniProtKB/TrEMBL;Acc:K7UK01]	3	24.267	Cleavage	miRNA 20 GGUCCCUUCUCCGUCACGUC 1<br>:: ::::.::::..::::.:<br>Target 942 CCCGGGAGGAGGUGGUGCGG 961

Potential targets of zma-miRNAs.

miRNA	Target	Gene name	Gene description	Expectation	UPE	Inhibition	Hybrid
zma-miR408a	GRMZM2G000842_T04	NA	Putative bZIP transcription factor superfamily protein [Source:UniProtKB/TrEMBL;Acc:K7UK01]	3	22.752	Cleavage	miRNA 20 GGUCCCUUCUCCGUCACGUC 1<br>:: ::::.::::..::::.:<br>Target 1049 CCCGGGAGGAGGUGGUGCGG 1068
zma-miR408a	GRMZM2G106026_T01	NA	NA	3	24.705	Cleavage	miRNA 21 CGGUCCCUUCUCCGUCACGUC 1<br>:::. :::::::: .::::::<br>Target 722 GCCGAGGAAGAGGAGGUGCAG 742
zma-miR408a	GRMZM2G138710_T01	NA	NA	3	21.891	Cleavage	miRNA 21 CGGUCCCUUCUCCGUCACGUC 1<br>:::.:: :::::::.::::..:<br>Target 1055 GCCGGGAAAGAGGCGGUGUGG 1075
zma-miR408a	GRMZM2G331566_T02	NA	Uncharacterized protein [Source:UniProtKB/TrEMBL;Acc:B4FVM4]	3	23.197	Cleavage	miRNA 20 GGUCCCUUCUCCGUCACGUC 1<br>: :: ::.:::::::::::.:<br>Target 174 CAAGCGAGGAGGCAGUGCGG 193
zma-miR408a	GRMZM2G459896_T01	NA	NA	3	24.159	Cleavage	miRNA 20 GGUCCCUUCUCCGUCACGUC 1<br>::: :::.::::: ::::::<br>Target 155 CCACGGAGGAGGCCGUGCAG 174
zma-miR408a	GRMZM2G075892_T01	NA	Uncharacterized protein [Source:UniProtKB/TrEMBL;Acc:C0HFX6]	3.5	23.165	Cleavage	miRNA 21 CGGUCCCUUCUCCGUCACGUC 1<br>:::.:: :.:::::.::::..:<br>Target 1055 GCCGGGAAGGAGGCGGUGUGG 1075
zma-miR408a	GRMZM2G082940_T01	NA	Blue copper protein [Source:UniProtKB/TrEMBL;Acc:B6UHQ8]	3.5	22.212	Cleavage	miRNA 21 CGGUCCCUUCUCCGUCACGUC 1<br>:..::: ::::::::::::: :<br>Target 653 GUUAGGCAAGAGGCAGUGCUG 673
zma-miR408a	GRMZM2G336337_T01	NA	Putative laccase family protein; Uncharacterized protein [Source:UniProtKB/TrEMBL;Acc:C0P5Q0]	3.5	20.698	Cleavage	miRNA 21 CGGUCCCUUCUCCGUCACGUC 1<br>::::: :::::::: :::::<br>Target 362 GCCAGUGAAGAGGCUGUGCAA 382
zma-miR408a	GRMZM2G384327_T02	NA	gamma response I protein [Source:RefSeq peptide;Acc:NP_001151159]	3.5	21.422	Cleavage	miRNA 21 CGGUCCCUUCUCCGUCACGUC 1<br>:: :: :::::::: ::::::<br>Target 1176 GCGAGAGAAGAGGCCGUGCAG 1196
zma-miR408a	GRMZM2G384327_T03	NA	gamma response I protein [Source:RefSeq peptide;Acc:NP_001151159]	3.5	21.422	Cleavage	miRNA 21 CGGUCCCUUCUCCGUCACGUC 1<br>:: :: :::::::: ::::::<br>Target 1387 GCGAGAGAAGAGGCCGUGCAG 1407
zma-miR408a	GRMZM2G384327_T04	NA	gamma response I protein [Source:RefSeq peptide;Acc:NP_001151159]	3.5	21.422	Cleavage	miRNA 21 CGGUCCCUUCUCCGUCACGUC 1<br>:: :: :::::::: ::::::<br>Target 1620 GCGAGAGAAGAGGCCGUGCAG 1640
zma-miR408a	GRMZM2G384327_T05	NA	gamma response I protein [Source:RefSeq peptide;Acc:NP_001151159]	3.5	21.422	Cleavage	miRNA 21 CGGUCCCUUCUCCGUCACGUC 1<br>:: :: :::::::: ::::::<br>Target 1414 GCGAGAGAAGAGGCCGUGCAG 1434
zma-miR408a	GRMZM2G458665_T01	NA	NA	3.5	17.376	Cleavage	miRNA 20 GGUCCCUUCUCCGUCACGUC 1<br>.:: ::.::::::::::.:<br>Target 1607 UCAUGGGAGAGGCAGUGUAU 1626
zma-miR408a	GRMZM2G001606_T01	NA	Uncharacterized protein [Source:UniProtKB/TrEMBL;Acc:C0HIB5]	4	22.103	Cleavage	miRNA 21 CGGUCCCUUCUCCGUCACGUC 1<br>:.:: ::::::::::::.:<br>Target 962 GUCAACGAAGAGGCAGUGUAA 982
zma-miR408a	GRMZM2G002898_T02	NA	Uncharacterized protein [Source:UniProtKB/TrEMBL;Acc:B4FC10]	4	17.862	Cleavage	miRNA 20 GGUCCCUUCUCCGUCACGUC 1<br>::::::::::::: ::..:<br>Target 1095 CCAGGGAAGAGGCCAUGUGG 1114

Potential targets of zma-miRNAs.

miRNA	Target	Gene name	Gene description	Expectation	UPE	Inhibition	Hybrid
zma-miR408a	GRMZM2G010439_T01	NA	Uncharacterized protein [Source:UniProtKB/TrEMBL;Acc:K7U1F2]	4	24.84	Cleavage	miRNA 21 CGGUCCCUUCUCCGUCACGUC 1<br>: :.::::.:::::.:: :::<br>Target 1934 GACGGGGAGGAGGCGGUACAG 1954
zma-miR408a	GRMZM2G044481_T01	NA	Ent-copalyl diphosphate synthase [Source:UniProtKB/TrEMBL;Acc:Q672R4]	4	22.733	Cleavage	miRNA 21 CGGUCCCUUCUCCGUC-ACGUC 1<br>:.::::::. :::::: :::::<br>Target 2077 GUCAGGGAGAAGGCAGAUGCAG 2098
zma-miR408a	GRMZM2G069784_T01	NA	Uncharacterized protein [Source:UniProtKB/TrEMBL;Acc:K7TJ73]	4	24.08	Cleavage	miRNA 20 GGUCCCUUCUCCGUCACGUC 1<br>::::::.:::: ::: :::<br>Target 1295 ACAGGGAGGAGGAAGUUCAG 1314
zma-miR408a	GRMZM2G097896_T01	NA	Acyl transferase/acyl hydrolase/lysophospholipase superfamily protein [Source:Projected from Arabidopsis thaliana (AT1G33270) TAIR;Acc:AT1G33270]	4	17.501	Cleavage	miRNA 21 CGGUCCCUUCUCCGUCACGUC 1<br>:: ::::: :.::.:::::..:<br>Target 499 GCGAGGGACGGGGUAGUGUGG 519
zma-miR408a	GRMZM2G099105_T01	NA	NA	4	22.465	Cleavage	miRNA 21 CGGUCCCUUCUCCGUCACGUC 1<br>:: :: ::.::::: ::::::<br>Target 339 GCGAGAGAGGAGGCCGUGCAG 359
zma-miR408a	GRMZM2G108615_T01	NA	NA	4	16.241	Cleavage	miRNA 21 CGGUC-CCUUCUCCGUCACGUC 1<br>:.:.: :::.::::..::::::<br>Target 454 GUCGGAGGAGGAGGUGGUGCAG 475
zma-miR408a	GRMZM2G113860_T01	NA	Uncharacterized protein [Source:UniProtKB/TrEMBL;Acc:K7VL82]	4	23.458	Cleavage	miRNA 21 CGGUCCCUUCUCCGUCACGUC 1<br>:.:. :::.:::: .::::::<br>Target 769 GUCGAGGAGGAGGAGGUGCAG 789
zma-miR408a	GRMZM2G118063_T01	NA	Homeodomain leucine zipper family IV protein; Putative homeobox/lipid-binding domain family protein isoform 1; Putative homeobox/lipid-binding domain family protein isoform 2; Uncharacterized protein [Source:UniProtKB/TrEMBL;Acc:B7ZX75]	4	19.926	Cleavage	miRNA 21 CGGUCCCUUCUCCGUCACGUC 1<br>:: :: :.::.::..::::::<br>Target 2751 GCGAGAGGAGGGGUGGUGCAG 2771
zma-miR408a	GRMZM2G118063_T02	NA	Homeodomain leucine zipper family IV protein; Putative homeobox/lipid-binding domain family protein isoform 1; Putative homeobox/lipid-binding domain family protein isoform 2; Uncharacterized protein [Source:UniProtKB/TrEMBL;Acc:B7ZX75]	4	21.877	Cleavage	miRNA 21 CGGUCCCUUCUCCGUCACGUC 1<br>:: :: :.::.::..::::::<br>Target 2840 GCGAGAGGAGGGGUGGUGCAG 2860
zma-miR408a	GRMZM2G118063_T03	NA	Homeodomain leucine zipper family IV protein; Putative homeobox/lipid-binding domain family protein isoform 1; Putative homeobox/lipid-binding domain family protein isoform 2; Uncharacterized protein [Source:UniProtKB/TrEMBL;Acc:B7ZX75]	4	19.926	Cleavage	miRNA 21 CGGUCCCUUCUCCGUCACGUC 1<br>:: :: :.::.::..::::::<br>Target 2739 GCGAGAGGAGGGGUGGUGCAG 2759
zma-miR408a	GRMZM2G129218_T01	NA	Uncharacterized protein [Source:UniProtKB/TrEMBL;Acc:K7UL98]	4	16.24	Cleavage	miRNA 20 GGUCCCUUCUCCGUCACGUC 1<br>::::: :::::::. ::::<br>Target 729 CCAGGAAAGAGGCGCUGCAA 748
zma-miR408a	GRMZM2G131957_T01	NA	Uncharacterized protein [Source:UniProtKB/TrEMBL;Acc:K7UN74]	4	10.284	Cleavage	miRNA 20 GGUCCCUUCUCCGUCACGUC 1<br>...:::::::::..::: ::<br>Target 1089 UUGGGGAAGAGGUGGUGGAG 1108
zma-miR408a	GRMZM2G131957_T02	NA	Uncharacterized protein [Source:UniProtKB/TrEMBL;Acc:K7UN74]	4	10.284	Cleavage	miRNA 20 GGUCCCUUCUCCGUCACGUC 1<br>...:::::::::..::: ::<br>Target 1089 UUGGGGAAGAGGUGGUGGAG 1108
zma-miR408a	GRMZM2G132169_T01	NA	Uncharacterized protein [Source:UniProtKB/TrEMBL;Acc:C0P4F2]	4	20.433	Cleavage	miRNA 21 CGGUCCCUUCUCCGUCACGUC 1<br>:::.: :::::::: :::::<br>Target 212 GCCGGUGAAGAGGCUGUGCAA 232
zma-miR408a	GRMZM2G132169_T02	NA	Uncharacterized protein [Source:UniProtKB/TrEMBL;Acc:C0P4F2]	4	20.433	Cleavage	miRNA 21 CGGUCCCUUCUCCGUCACGUC 1<br>:::.: :::::::: :::::<br>Target 212 GCCGGUGAAGAGGCUGUGCAA 232

Potential targets of zma-miRNAs.

miRNA	Target	Gene name	Gene description	Expectation	UPE	Inhibition	Hybrid
zma-miR408a	GRMZM2G165969_T01	NA	Uncharacterized protein [Source:UniProtKB/TrEMBL;Acc:B4FLK5]	4	23.609	Cleavage	miRNA 21 CGGUCCCUUCUCCGUCACGUC 1<br>:: . ::::::::::: :::<br>Target 567 GCAGCGGAAGAGGCAGUACAG 587
zma-miR408a	GRMZM2G165969_T02	NA	Uncharacterized protein [Source:UniProtKB/TrEMBL;Acc:B4FLK5]	4	23.609	Cleavage	miRNA 21 CGGUCCCUUCUCCGUCACGUC 1<br>:: . ::::::::::: :::<br>Target 592 GCAGCGGAAGAGGCAGUACAG 612
zma-miR408a	GRMZM2G165969_T03	NA	Uncharacterized protein [Source:UniProtKB/TrEMBL;Acc:B4FLK5]	4	23.609	Cleavage	miRNA 21 CGGUCCCUUCUCCGUCACGUC 1<br>:: . ::::::::::: :::<br>Target 497 GCAGCGGAAGAGGCAGUACAG 517
zma-miR408a	GRMZM2G171317_T01	NA	DNA-3-methyladenine glycosylase I [Source:UniProtKB/TrEMBL;Acc:K7VDZ5]	4	20.798	Cleavage	miRNA 21 CGGUCCCUUCUCCGUCACGUC 1<br>:: ::::::::: .::::.<br>Target 1125 GCAAGGGAAGAGGAGGUGCGC 1145
zma-miR408a	GRMZM2G322748_T01	NA	Disease resistance gene analog PIC15 [Source:UniProtKB/TrEMBL;Acc:Q9ZTJ0]	4	22.163	Cleavage	miRNA 21 CGGUCCCUUCUCCGUCACGUC 1<br>: :::::: ::::.:::: ::<br>Target 680 GACAGGGACGAGGUAGUGAAG 700
zma-miR408a	GRMZM2G341405_T03	NA	NA	4	24.119	Translation	miRNA 21 CGGUCCCUUCUCCGUCACGUC 1<br>::::: :.::: :::::: ::<br>Target 2762 GCCAGUGGAGAUGCAGUGGAG 2782
zma-miR408a	GRMZM2G341405_T05	NA	NA	4	24.262	Translation	miRNA 21 CGGUCCCUUCUCCGUCACGUC 1<br>::::: :.::: :::::: ::<br>Target 2953 GCCAGUGGAGAUGCAGUGGAG 2973
zma-miR408a	AC212463.3_FGT009	NA	NA	4.5	19.457	Cleavage	miRNA 21 CGGUCCCUUCUCCGUCACGUC 1<br>:...:::.::.:::.::: ::<br>Target 277 GUUGGGGGAGGGGCGGUGGAG 297
zma-miR408a	GRMZM2G015605_T01	NA	NaCl stress protein1; Uncharacterized protein [Source:UniProtKB/TrEMBL;Acc:C0HIJ6]	4.5	15.689	Cleavage	miRNA 21 CGGUCCCUUCUCCGUCACGUC 1<br>:::::::::::::: ::.<br>Target 312 GCCAGGGAAGAGGCAAAGCGA 332
zma-miR408a	GRMZM2G035843_T01	NA	Putative calcium-dependent protein kinase family protein [Source:UniProtKB/TrEMBL;Acc:K7V3X5]	4.5	23.866	Cleavage	miRNA 21 CGGUCCCUUCUCCGUCACGUC 1<br>::..::: ::.:::::: :.:<br>Target 1013 GCUGGGGCAGGGGCAGUUCGG 1033
zma-miR408a	GRMZM2G053722_T01	NA	protein serine/threonine phosphatases;protein kinases;catalytics;cAMP-dependent protein kinase regulators;<br>ATP binding;protein serine/threonine phosphatases [Source:Projected from Arabidopsis thaliana (AT2G20050) TAIR;Acc:AT2G20050]	4.5	19.757	Cleavage	miRNA 21 CGGUCCCUUCUCCGUCACGUC 1<br>:.:.:::.::::: :: :.::<br>Target 546 GUCGGGGGAGAGGGAGGGUAG 566
zma-miR408a	GRMZM2G069976_T01	NA	Uncharacterized protein [Source:UniProtKB/TrEMBL;Acc:B4FBT3]	4.5	23.534	Cleavage	miRNA 21 CGGUCCCUUCUCCGUCACGUC 1<br>: :.::::.:::::. :::.:<br>Target 154 GGCGGGGAGGAGGCGAUGCGG 174
zma-miR408a	GRMZM2G071928_T01	NA	Uncharacterized protein [Source:UniProtKB/TrEMBL;Acc:K7TXX0]	4.5	22.788	Cleavage	miRNA 21 CGGUCCCUUCUCCGUCACGUC 1<br>:.:. ::::::::..::: ::<br>Target 211 GUCGAGGAAGAGGUGGUGAAG 231
zma-miR408a	GRMZM2G077340_T01	NA	Uncharacterized protein [Source:UniProtKB/TrEMBL;Acc:K7V958]	4.5	21.445	Cleavage	miRNA 21 CGGUCCCUUCUCCGUCACGUC 1<br>:.:: :::::::::. ::::<br>Target 3176 GUCAUGGAAGAGGCGAUGCAC 3196
zma-miR408a	GRMZM2G077340_T02	NA	Uncharacterized protein [Source:UniProtKB/TrEMBL;Acc:K7V958]	4.5	21.445	Cleavage	miRNA 21 CGGUCCCUUCUCCGUCACGUC 1<br>:.:: :::::::::. ::::<br>Target 3176 GUCAUGGAAGAGGCGAUGCAC 3196

Potential targets of zma-miRNAs.

miRNA	Target	Gene name	Gene description	Expectation	UPE	Inhibition	Hybrid
zma-miR408a	GRMZM2G081048_T01	NA	FAD-dependent oxidoreductase family protein [Source:Projected from Arabidopsis thaliana (AT5G48440) TAIR;Acc:AT5G48440]	4.5	22.46	Translation	miRNA 21 CGGUCCCUUCUCCGUCACGUC 1<br>::::::::.: :::.: :::<br>Target 508 GCCAGGGAGGCGGCGGAGCAC 528
zma-miR408a	GRMZM2G081048_T02	NA	FAD-dependent oxidoreductase family protein [Source:Projected from Arabidopsis thaliana (AT5G48440) TAIR;Acc:AT5G48440]	4.5	22.46	Translation	miRNA 21 CGGUCCCUUCUCCGUCACGUC 1<br>::::::::.: :::.: :::<br>Target 622 GCCAGGGAGGCGGCGGAGCAC 642
zma-miR408a	GRMZM2G081048_T03	NA	FAD-dependent oxidoreductase family protein [Source:Projected from Arabidopsis thaliana (AT5G48440) TAIR;Acc:AT5G48440]	4.5	22.46	Translation	miRNA 21 CGGUCCCUUCUCCGUCACGUC 1<br>::::::::.: :::.: :::<br>Target 640 GCCAGGGAGGCGGCGGAGCAC 660
zma-miR408a	GRMZM2G081048_T04	NA	FAD-dependent oxidoreductase family protein [Source:Projected from Arabidopsis thaliana (AT5G48440) TAIR;Acc:AT5G48440]	4.5	22.46	Translation	miRNA 21 CGGUCCCUUCUCCGUCACGUC 1<br>::::::::.: :::.: :::<br>Target 514 GCCAGGGAGGCGGCGGAGCAC 534
zma-miR408a	GRMZM2G081048_T05	NA	FAD-dependent oxidoreductase family protein [Source:Projected from Arabidopsis thaliana (AT5G48440) TAIR;Acc:AT5G48440]	4.5	24.24	Translation	miRNA 21 CGGUCCCUUCUCCGUCACGUC 1<br>::::::::.: :::.: :::<br>Target 514 GCCAGGGAGGCGGCGGAGCAC 534
zma-miR408a	GRMZM2G081048_T06	NA	FAD-dependent oxidoreductase family protein [Source:Projected from Arabidopsis thaliana (AT5G48440) TAIR;Acc:AT5G48440]	4.5	22.46	Translation	miRNA 21 CGGUCCCUUCUCCGUCACGUC 1<br>::::::::.: :::.: :::<br>Target 496 GCCAGGGAGGCGGCGGAGCAC 516
zma-miR408a	GRMZM2G103884_T01	NA	NA	4.5	22.681	Cleavage	miRNA 21 CGGUCCCUUCUCCGUCACGUC 1<br>:.::::::.:.:::.: :::<br>Target 563 GUCAGGGAGGGGGCGGCGCAU 583
zma-miR408a	GRMZM2G103884_T02	NA	NA	4.5	22.681	Cleavage	miRNA 21 CGGUCCCUUCUCCGUCACGUC 1<br>:.::::::.:.:::.: :::<br>Target 575 GUCAGGGAGGGGGCGGCGCAU 595
zma-miR408a	GRMZM2G106338_T01	NA	Putative uncharacterized protein [Source:UniProtKB/TrEMBL;Acc:B6TDQ1]	4.5	23.166	Cleavage	miRNA 21 CGGUCCCUUCUCCGUCACGUC 1<br>:.::::::.:.:::.: :::<br>Target 601 GUCAGGGAGGGGGCGGCGCAU 621
zma-miR408a	GRMZM2G106680_T01	NA	NA	4.5	13.067	Cleavage	miRNA 21 CGGUCCCUUCUCCGUCACGUC 1<br>::.:::::::::: : :::.<br>Target 153 GCUAGGGAAGAGGAACUGCGA 173
zma-miR408a	GRMZM2G124715_T02	NA	Uncharacterized protein [Source:UniProtKB/TrEMBL;Acc:B7ZZL6]	4.5	20.687	Cleavage	miRNA 20 GGUCCCUUCUCCGUCACGUC 1<br>. ::::.::.::::: ::::<br>Target 1240 UGAGGGGAGGGGCAGAGCAG 1259
zma-miR408a	GRMZM2G124715_T03	NA	Uncharacterized protein [Source:UniProtKB/TrEMBL;Acc:B7ZZL6]	4.5	20.687	Cleavage	miRNA 20 GGUCCCUUCUCCGUCACGUC 1<br>. ::::.::.::::: ::::<br>Target 1128 UGAGGGGAGGGGCAGAGCAG 1147
zma-miR408a	GRMZM2G126204_T01	NA	Uncharacterized protein [Source:UniProtKB/TrEMBL;Acc:K7V958]	4.5	21.445	Cleavage	miRNA 21 CGGUCCCUUCUCCGUCACGUC 1<br>:.:: :::::::::. ::::<br>Target 3176 GUCAUGGAAGAGGCGAUGCAC 3196
zma-miR408a	GRMZM2G128981_T01	NA	Uncharacterized protein [Source:UniProtKB/TrEMBL;Acc:K7UTX0]	4.5	23.614	Cleavage	miRNA 21 CGGUCCCUUCUCCGUCACGUC 1<br>:.:. ::.:::::::.: ::::<br>Target 776 GUCGUGGGAGAGGCGGAGCAG 796
zma-miR408a	GRMZM2G138419_T01	NA	Thaumatin-like protein [Source:UniProtKB/TrEMBL;Acc:B6U579]	4.5	21.894	Cleavage	miRNA 21 CGGUCCCUUCUCCGUCACGUC 1<br>:::.::: ::::::::: ::<br>Target 3643 GCCGGGGCAGAGGCAGUUGAG 3663

Potential targets of zma-miRNAs.

miRNA	Target	Gene name	Gene description	Expectation	UPE	Inhibition	Hybrid
zma-miR408a	GRMZM2G139643_T01	NA	Heterogeneous nuclear ribonucleoprotein A3 isoform 1; Heterogeneous nuclear ribonucleoprotein A3 isoform 2; Heterogeneous nuclear ribonucleoprotein A3 isoform 3; Uncharacterized protein [Source:UniProtKB/TrEMBL;Acc:B4FHJ8]	4.5	24.525	Cleavage	miRNA 21 CGGUCCCUUCUCCGUCACGUC 1<br>:.:::::.:::: .: ::.:<br>Target 527 GUCAGGGGAGAGGGGGCGCGG 547
zma-miR408a	GRMZM2G157307_T01	NA	NA	4.5	23.79	Cleavage	miRNA 21 CGGUCCCUUCUCCGUCACGUC 1<br>:...:::.::.:::.::: ::<br>Target 236 GUUGGGGGAGGGGCGGUGGAG 256
zma-miR408a	GRMZM2G161382_T01	NA	Cyclin delta-3 [Source:UniProtKB/TrEMBL;Acc:B6U905]	4.5	24.673	Cleavage	miRNA 21 CGGUCCCUUCUCCGUCACGUC 1<br>:::: :::.:.::..:::: :<br>Target 485 GCCAAGGAGGGGGUGGUGCUG 505
zma-miR408a	GRMZM2G161382_T02	NA	Cyclin delta-3 [Source:UniProtKB/TrEMBL;Acc:B6U905]	4.5	24.673	Cleavage	miRNA 21 CGGUCCCUUCUCCGUCACGUC 1<br>:::: :::.:.::..:::: :<br>Target 498 GCCAAGGAGGGGGUGGUGCUG 518
zma-miR408a	GRMZM2G327999_T01	NA	NA	4.5	22.22	Cleavage	miRNA 21 CGGUCCCUUCUCCGUCACGUC 1<br>:...:::.::.:::.::: ::<br>Target 1607 GUUGGGGGAGGGGCGGUGGAG 1627
zma-miR408a	GRMZM2G347226_T01	NA	Putative calcium-dependent protein kinase family protein [Source:UniProtKB/TrEMBL;Acc:K7TJ28]	4.5	23.714	Cleavage	miRNA 21 CGGUCCCUUCUCCGUCACGUC 1<br>::..::: ::.:::::: :.:<br>Target 461 GCUGGGGCAGGGGCAGUUCGG 481
zma-miR408a	GRMZM2G347226_T03	NA	Putative calcium-dependent protein kinase family protein [Source:UniProtKB/TrEMBL;Acc:K7TJ28]	4.5	23.714	Cleavage	miRNA 21 CGGUCCCUUCUCCGUCACGUC 1<br>::..::: ::.:::::: :.:<br>Target 766 GCUGGGGCAGGGGCAGUUCGG 786
zma-miR408a	GRMZM2G369469_T01	NA	Uncharacterized protein [Source:UniProtKB/TrEMBL;Acc:K7VCY2]	4.5	22.921	Cleavage	miRNA 21 CGGUCCCUUCUCCGUCACGUC 1<br>:::. :::.::::..::: ::<br>Target 320 GCCGAGGAGGAGGUGGUGGAG 340
zma-miR408a	GRMZM5G837318_T01	NA	NA	4.5	24.353	Cleavage	miRNA 21 CGGUCCCUUCUCCGUCACGUC 1<br>:::.::::.:::::.: :: :<br>Target 865 GCCGGGGAGGAGGCGGGGCCG 885
zma-miR408a	GRMZM5G896114_T01	NA	NA	4.5	22.97	Cleavage	miRNA 21 CGGUCCCUUCUCCGUCACGUC 1<br>::..:::.::.::..: ::::<br>Target 704 GCUGGGGGAGGGGUGGAGCAG 724
zma-miR408a	GRMZM2G039381_T01	NA	Uncharacterized protein [Source:UniProtKB/TrEMBL;Acc:K7TMU1]	5	24.271	Cleavage	miRNA 20 GGUCCCUUCUCCGUCACGUC 1<br>::::::::::::::: :<br>Target 229 GCAGGGAAGAGGCAGUGGUG 248
zma-miR408a	GRMZM2G116614_T01	NA	Uncharacterized protein [Source:UniProtKB/TrEMBL;Acc:C0PFI1]	5	22.032	Cleavage	miRNA 21 CGGUCCCUUCUCCGUCACGUC 1<br>::..::::.:::::. ::::<br>Target 486 GCUGGGGAGGAGGCGCAGCAG 506
zma-miR528b-5p	GRMZM2G068271_T02	NA	Uncharacterized protein [Source:UniProtKB/TrEMBL;Acc:B6STF9]	2	24.442	Translation	miRNA 20 AGGAGACGUACGGGGAAGGU 1<br>::::: ::: :::::::::<br>Target 77 UCCUCCGCACGCCCCUUCCA 96
zma-miR528b-5p	GRMZM2G148937_T01	NA	MATE efflux family protein [Source:Projected from Arabidopsis thaliana (AT1G71870) TAIR;Acc:AT1G71870]	2	24.771	Cleavage	miRNA 21 GAGGAGACGUACGGGGAAGGU 1<br>::::::::: ::::.:::.::<br>Target 102 CUCCUCUGCCUGCCUCUUUCA 122
zma-miR528b-5p	GRMZM2G045955_T02	NA	Uncharacterized protein [Source:UniProtKB/TrEMBL;Acc:C0PE57]	2.5	14.805	Cleavage	miRNA 20 AGGAGACGUACGGGGAAGGU 1<br>::::.::::::.:: :::::<br>Target 1261 UCCUUUGCAUGUCCAUUCCA 1280

Potential targets of zma-miRNAs.

miRNA	Target	Gene name	Gene description	Expectation	UPE	Inhibition	Hybrid
zma-miR528b-5p	GRMZM2G065893_T01	NA	Uncharacterized protein [Source:UniProtKB/TrEMBL;Acc:K7V2F6]	2.5	20.149	Translation	miRNA 21 GAGGAGACGUACGGGGAAGGU 1<br>.:::::::: : ::::::::.<br>Target 1447 UUCCUCUGCCUCCCCCUUCCG 1467
zma-miR528b-5p	GRMZM2G106928_T01	NA	Superoxide dismutase [Cu-Zn] [Source:UniProtKB/TrEMBL;Acc:B1PEY4]	2.5	11.294	Translation	miRNA 21 GAGGAGACGUACGGGGAAGGU 1<br>.::::: ::: ::::.:::::<br>Target 171 UUCCUCCGCACGCCCUUUCCA 191
zma-miR528b-5p	GRMZM2G106928_T02	NA	Superoxide dismutase [Cu-Zn] [Source:UniProtKB/TrEMBL;Acc:B1PEY4]	2.5	11.294	Translation	miRNA 21 GAGGAGACGUACGGGGAAGGU 1<br>.::::: ::: ::::.:::::<br>Target 173 UUCCUCCGCACGCCCUUUCCA 193
zma-miR528b-5p	GRMZM2G107562_T01	NA	Copper ion binding protein; Uncharacterized protein [Source:UniProtKB/TrEMBL;Acc:B6TG00]	2.5	18.145	Cleavage	miRNA 21 GAGGAGACGUACGGGGAAGGU 1<br>::::::::: ::::::::::.<br>Target 570 CUCCUCUGC-UGCCCCUUCCG 589
zma-miR528b-5p	GRMZM5G839518_T02	NA	NA	2.5	14.21	Cleavage	miRNA 21 GAGGAGACGUACGGGGAAGGU 1<br>:: .:.:::::::.:::::::<br>Target 1660 CUGUUUUGCAUGCUCCUUCCA 1680
zma-miR528b-5p	GRMZM5G839518_T03	NA	NA	2.5	14.21	Cleavage	miRNA 21 GAGGAGACGUACGGGGAAGGU 1<br>:: .:.:::::::.:::::::<br>Target 1763 CUGUUUUGCAUGCUCCUUCCA 1783
zma-miR528b-5p	AC207620.2_FGT007	NA	NA	3	21.312	Translation	miRNA 21 GAGGAGACGUACGGGGAAGGU 1<br>:::::::::: ::::::::..<br>Target 623 CUCCUCUGCA-GCCCCUUCUG 642
zma-miR528b-5p	GRMZM2G020150_T01	NA	Putative AP2/EREBP transcription factor superfamily protein [Source:UniProtKB/TrEMBL;Acc:K7UL38]	3	14.751	Translation	miRNA 21 GAGGAGACGUACGGGGAAGGU 1<br>::::::::..: ..:::::::<br>Target 888 CUCCUCUGUGUCUUCCUUCCA 908
zma-miR528b-5p	GRMZM2G040278_T01	NA	F-box protein GID2; Uncharacterized protein [Source:UniProtKB/TrEMBL;Acc:C0PDX0]	3	21.767	Translation	miRNA 21 GAGGAGACGUACGGGGAAGGU 1<br>::::::::: :.::.:::::<br>Target 664 CUCCUCUGCUCGUCCUUUCCA 684
zma-miR528b-5p	GRMZM2G053047_T01	NA	NA	3	18.045	Translation	miRNA 20 AGGAGACGUACGGGGAAGGU 1<br>::.::::::::: ::::.::<br>Target 936 UCUUCUGCAUGAGCCUUUCA 955
zma-miR528b-5p	GRMZM2G053047_T02	NA	NA	3	13.091	Translation	miRNA 20 AGGAGACGUACGGGGAAGGU 1<br>::.::::::::: ::::.::<br>Target 871 UCUUCUGCAUGAGCCUUUCA 890
zma-miR528b-5p	GRMZM2G053047_T03	NA	NA	3	13.091	Translation	miRNA 20 AGGAGACGUACGGGGAAGGU 1<br>::.::::::::: ::::.::<br>Target 871 UCUUCUGCAUGAGCCUUUCA 890
zma-miR528b-5p	GRMZM2G073504_T01	NA	Uncharacterized protein [Source:UniProtKB/TrEMBL;Acc:B4FZ01]	3	16.871	Cleavage	miRNA 20 AGGAGACGUACGGGGAAGGU 1<br>:.::: ::.::::::::::..<br>Target 1063 UUCUCGGCGUGCCCCUUCUG 1082
zma-miR528b-5p	GRMZM2G073504_T02	NA	Uncharacterized protein [Source:UniProtKB/TrEMBL;Acc:B4FZ01]	3	16.871	Cleavage	miRNA 20 AGGAGACGUACGGGGAAGGU 1<br>:.::: ::.::::::::::..<br>Target 1097 UUCUCGGCGUGCCCCUUCUG 1116
zma-miR528b-5p	GRMZM2G132121_T01	NA	40S ribosomal protein S27 [Source:UniProtKB/TrEMBL;Acc:Q9ZQX9]	3	19.72	Cleavage	miRNA 21 GAGGAGACGUACGGGGAAGGU 1<br>.:::::: :::::::.::.:<br>Target 320 UUCCUCUUCAUGCCCUUUUCU 340

miRNA	Target	Gene name	Gene description	Expectation	UPE	Inhibition	Hybrid
zma-miR528b-5p	GRMZM2G177781_T03	NA	Uncharacterized protein [Source:UniProtKB/TrEMBL;Acc:B4F9M6]	3	8.991	Cleavage	miRNA 21 GAGGAGACGUACGGGGAAGGU 1<br>:: .::: :::::::::.::<br>Target 6 CUGUUCUCCAUGCCCCUUUCA 26
zma-miR528b-5p	GRMZM2G342815_T01	NA	NA	3	16.224	Translation	miRNA 20 AGGAGACGUACGGGGAAGGU 1<br>::.:::::::: ::::.::<br>Target 714 UCUUCUGCAUGAGCCUUUCA 733
zma-miR528b-5p	GRMZM2G367668_T01	NA	L-ascorbate oxidase [Source:UniProtKB/TrEMBL;Acc:B6UCC4]	3	14.609	Cleavage	miRNA 21 GAGGAGACGUACGGGGAAGGU 1<br>::.::: :::::.:::::::<br>Target 222 CUUCUCCGCAUGUCCCUUCCU 242
zma-miR528b-5p	GRMZM2G384695_T01	NA	NA	3	16.382	Cleavage	miRNA 21 GAGGAGACGUACGGGGAAGGU 1<br>.:::::::: ::: ::::::<br>Target 13 UUCCUCUGCCUGCACCUUCCU 33
zma-miR528b-5p	GRMZM2G440543_T01	NA	F-box protein GID2 [Source:UniProtKB/TrEMBL;Acc:B6SQT1]	3	23.994	Translation	miRNA 21 GAGGAGACGUACGGGGAAGGU 1<br>::::::::: :.::.:::::<br>Target 748 CUCCUCUGCUCGUCCUUUCCA 768
zma-miR528b-5p	GRMZM2G470382_T01	NA	NA	3	19.87	Translation	miRNA 20 AGGAGACGUACGGGGAAGGU 1<br>::.:::::::: ::::.::<br>Target 1674 UCUUCUGCAUGAGCCUUUCA 1693
zma-miR528b-5p	GRMZM2G478599_T01	NA	NA	3	19.476	Cleavage	miRNA 21 GAGGAGACGUACGGGGAAGGU 1<br>.::.: :::::::.:::::..<br>Target 31 UUCUUGUGCAUGCUCCUUCUG 51
zma-miR528b-5p	AC230011.2_FGT002	NA	Rust resistance protein rp3-1 [Source:UniProtKB/TrEMBL;Acc:Q6PW75]	3.5	22.741	Cleavage	miRNA 21 GAGGAGACGUACGGGGAAGGU 1<br>.::.:: : ::::..::::::<br>Target 3414 UUCUUCAGGAUGCUUCUUCCA 3434
zma-miR528b-5p	AC231183.1_FGT002	NA	NA	3.5	16.941	Translation	miRNA 21 GAGGAGACGUACGGGGAAGGU 1<br>:::::: ::. :::::::::<br>Target 758 CUCCUCCGCGGCCCCCUUCCA 778
zma-miR528b-5p	GRMZM2G005126_T03	NA	Uncharacterized protein [Source:UniProtKB/TrEMBL;Acc:K7TI82]	3.5	20.243	Cleavage	miRNA 21 GAGGAGACGUACGGGGAAGGU 1<br>:::.::::..::::..::::..<br>Target 888 CUCUUCUGUGUGCCUUUUCUG 908
zma-miR528b-5p	GRMZM2G013761_T01	NA	Uncharacterized protein [Source:UniProtKB/TrEMBL;Acc:K7TSE5]	3.5	16.827	Cleavage	miRNA 21 GAGGAGACGUACGGGGAAGGU 1<br>::::::: : ::::::: :::<br>Target 93 CUCCUCUCCCUGCCCCUCCCA 113
zma-miR528b-5p	GRMZM2G013761_T02	NA	Uncharacterized protein [Source:UniProtKB/TrEMBL;Acc:K7TSE5]	3.5	16.827	Cleavage	miRNA 21 GAGGAGACGUACGGGGAAGGU 1<br>::::::: : ::::::: :::<br>Target 93 CUCCUCUCCCUGCCCCUCCCA 113
zma-miR528b-5p	GRMZM2G013761_T03	NA	Uncharacterized protein [Source:UniProtKB/TrEMBL;Acc:K7TSE5]	3.5	16.827	Cleavage	miRNA 21 GAGGAGACGUACGGGGAAGGU 1<br>::::::: : ::::::: :::<br>Target 93 CUCCUCUCCCUGCCCCUCCCA 113
zma-miR528b-5p	GRMZM2G015238_T01	NA	Prolyl endopeptidase [Source:UniProtKB/TrEMBL;Acc:K7TFH9]	3.5	8.291	Cleavage	miRNA 21 GAGGAGACGUACGGGGAAGGU 1<br>: :::: :: :::.:::::::<br>Target 77 CCCCUCCGCCUGCUCCUUCCA 97
zma-miR528b-5p	GRMZM2G018770_T01	NA	Uncharacterized protein [Source:UniProtKB/TrEMBL;Acc:B4FFZ3]	3.5	17.2	Translation	miRNA 21 GAGGAGACGUACGGGGAAGGU 1<br>.:::::: ::: :.::::::<br>Target 741 UUCCUCUCCAUCCUCCUUCCU 761

Potential targets of zma-miRNAs.

miRNA	Target	Gene name	Gene description	Expectation	UPE	Inhibition	Hybrid
zma-miR528b-5p	GRMZM2G054393_T01	NA	Uncharacterized protein [Source:UniProtKB/TrEMBL;Acc:B6TMF5]	3.5	24.615	Translation	miRNA 21 GAGGAGACGUACGGGGAAGGU 1<br>:::::::..: .:::::::<br>Target 554 CUCCUCUGUGUCUCCCUUCCU 574
zma-miR528b-5p	GRMZM2G054393_T02	NA	Uncharacterized protein [Source:UniProtKB/TrEMBL;Acc:B6TMF5]	3.5	24.615	Translation	miRNA 21 GAGGAGACGUACGGGGAAGGU 1<br>:::::::..: .:::::::<br>Target 554 CUCCUCUGUGUCUCCCUUCCU 574
zma-miR528b-5p	GRMZM2G057519_T01	NA	Uncharacterized protein [Source:UniProtKB/TrEMBL;Acc:K7UD89]	3.5	14.981	Cleavage	miRNA 21 GAGGAGACGUACGGGGAAGGU 1<br>::::::: : ::::::: :::<br>Target 90 CUCCUCUCCCUGCCCCUCCCA 110
zma-miR528b-5p	GRMZM2G064001_T01	NA	Haloacid dehalogenase-like hydrolase (HAD) superfamily protein [Source:Projected from Arabidopsis thaliana (AT3G10970) TAIR;Acc:AT3G10970]	3.5	8.105	Translation	miRNA 21 GAGGAGACGUACGGGGAAGGU 1<br>: .:::::: : :::::::::<br>Target 86 CCUCUCUGCUUUCCCCUUCCA 106
zma-miR528b-5p	GRMZM2G064001_T02	NA	Haloacid dehalogenase-like hydrolase (HAD) superfamily protein [Source:Projected from Arabidopsis thaliana (AT3G10970) TAIR;Acc:AT3G10970]	3.5	8.105	Translation	miRNA 21 GAGGAGACGUACGGGGAAGGU 1<br>: .:::::: : :::::::::<br>Target 86 CCUCUCUGCUUUCCCCUUCCA 106
zma-miR528b-5p	GRMZM2G064001_T03	NA	Haloacid dehalogenase-like hydrolase (HAD) superfamily protein [Source:Projected from Arabidopsis thaliana (AT3G10970) TAIR;Acc:AT3G10970]	3.5	8.105	Translation	miRNA 21 GAGGAGACGUACGGGGAAGGU 1<br>: .:::::: : :::::::::<br>Target 86 CCUCUCUGCUUUCCCCUUCCA 106
zma-miR528b-5p	GRMZM2G064283_T01	NA	NA	3.5	13.796	Cleavage	miRNA 21 GAGGAGACGUACGGGGAAGGU 1<br>.::: :::: ::::.:::.:.<br>Target 1090 UUCCACUGCUUGCCUCUUUCG 1110
zma-miR528b-5p	GRMZM2G067974_T01	NA	NA	3.5	16.617	Cleavage	miRNA 21 GAGGAGACGUACGGGGAAGGU 1<br>::::::: : ::::::: :::<br>Target 93 CUCCUCUCCCUGCCCCUCCCA 113
zma-miR528b-5p	GRMZM2G067974_T02	NA	NA	3.5	16.617	Cleavage	miRNA 21 GAGGAGACGUACGGGGAAGGU 1<br>::::::: : ::::::: :::<br>Target 93 CUCCUCUCCCUGCCCCUCCCA 113
zma-miR528b-5p	GRMZM2G067974_T03	NA	NA	3.5	16.617	Cleavage	miRNA 21 GAGGAGACGUACGGGGAAGGU 1<br>::::::: : ::::::: :::<br>Target 93 CUCCUCUCCCUGCCCCUCCCA 113
zma-miR528b-5p	GRMZM2G075701_T01	NA	Uncharacterized protein [Source:UniProtKB/TrEMBL;Acc:B4FB47]	3.5	21.71	Cleavage	miRNA 21 GAGGAGACGUACGGGGAAGGU 1<br>.:::::: :::::::.: ::.<br>Target 886 UUCCUCUCCAUGCCCUUCCCG 906
zma-miR528b-5p	GRMZM2G080689_T01	NA	Peroxidase 52 [Source:UniProtKB/TrEMBL;Acc:K7VH54]	3.5	17.568	Cleavage	miRNA 21 GAGGAGACGUACGGGGAAGGU 1<br>:: :::::: :::..:::::.<br>Target 233 CUGCUCUGCCUGCUUCUUCCG 253
zma-miR528b-5p	GRMZM2G082079_T01	NA	Uncharacterized protein [Source:UniProtKB/TrEMBL;Acc:K7UVY4]	3.5	19.102	Cleavage	miRNA 21 GAGGAGACGUACGGGGAAGGU 1<br>::::::: : ::::::: :::<br>Target 93 CUCCUCUCCCUGCCCCUCCCA 113
zma-miR528b-5p	GRMZM2G084465_T01	NA	40S ribosomal protein S23; Uncharacterized protein [Source:UniProtKB/TrEMBL;Acc:B4FBY5]	3.5	18.997	Cleavage	miRNA 20 AGGAGACGUACGGGGAAGGU 1<br>.:::::: :::::.::.::<br>Target 547 GUCUCUGCUUGCCCUUUUCA 566
zma-miR528b-5p	GRMZM2G084465_T02	NA	40S ribosomal protein S23; Uncharacterized protein [Source:UniProtKB/TrEMBL;Acc:B4FBY5]	3.5	18.997	Cleavage	miRNA 20 AGGAGACGUACGGGGAAGGU 1<br>.:::::: :::::.::.::<br>Target 720 GUCUCUGCUUGCCCUUUUCA 739

Potential targets of zma-miRNAs.

miRNA	Target	Gene name	Gene description	Expectation	UPE	Inhibition	Hybrid
zma-miR528b-5p	GRMZM2G084465_T03	NA	40S ribosomal protein S23; Uncharacterized protein [Source:UniProtKB/TrEMBL;Acc:B4FBY5]	3.5	18.997	Cleavage	miRNA 20 AGGAGACGUACGGGGAAGGU 1<br>.:::::: :::::.::.::<br>Target 1369 GUCUCUGCUUGCCCUUUUCA 1388
zma-miR528b-5p	GRMZM2G084465_T04	NA	40S ribosomal protein S23; Uncharacterized protein [Source:UniProtKB/TrEMBL;Acc:B4FBY5]	3.5	18.997	Cleavage	miRNA 20 AGGAGACGUACGGGGAAGGU 1<br>.:::::: :::::.::.::<br>Target 620 GUCUCUGCUUGCCCUUUUCA 639
zma-miR528b-5p	GRMZM2G107469_T01	NA	NA	3.5	19.214	Cleavage	miRNA 21 GAGGAGACGUACGGGGAAGGU 1<br>::::::: : ::::::: :::<br>Target 93 CUCCUCUCCCUGCCCCUCCCA 113
zma-miR528b-5p	GRMZM2G107469_T02	NA	NA	3.5	19.214	Cleavage	miRNA 21 GAGGAGACGUACGGGGAAGGU 1<br>::::::: : ::::::: :::<br>Target 93 CUCCUCUCCCUGCCCCUCCCA 113
zma-miR528b-5p	GRMZM2G116517_T03	NA	Cytochrome c oxidase copper chaperone [Source:UniProtKB/TrEMBL;Acc:B6STD7]	3.5	24.618	Translation	miRNA 21 GAGGAGACGUACGGGGAAGGU 1<br>.:.::.::: : .::::::::<br>Target 64 UUUCUUUGCUUCUCCCUUCCA 84
zma-miR528b-5p	GRMZM2G116517_T05	NA	Cytochrome c oxidase copper chaperone [Source:UniProtKB/TrEMBL;Acc:B6STD7]	3.5	24.931	Translation	miRNA 21 GAGGAGACGUACGGGGAAGGU 1<br>.:.::.::: : .::::::::<br>Target 64 UUUCUUUGCUUCUCCCUUCCA 84
zma-miR528b-5p	GRMZM2G116517_T06	NA	Cytochrome c oxidase copper chaperone [Source:UniProtKB/TrEMBL;Acc:B6STD7]	3.5	24.605	Translation	miRNA 21 GAGGAGACGUACGGGGAAGGU 1<br>.:.::.::: : .::::::::<br>Target 64 UUUCUUUGCUUCUCCCUUCCA 84
zma-miR528b-5p	GRMZM2G123667_T01	NA	Putative NAC domain transcription factor superfamily protein; Uncharacterized protein [Source:UniProtKB/TrEMBL;Acc:C4J9Z1]	3.5	15.099	Translation	miRNA 21 GAGGAGACGUACGGGGAAGGU 1<br>::: ::: :: :::.::::::<br>Target 845 CUCAUCUCCAAGCCUCUUCCA 865
zma-miR528b-5p	GRMZM2G123667_T06	NA	Putative NAC domain transcription factor superfamily protein; Uncharacterized protein [Source:UniProtKB/TrEMBL;Acc:C4J9Z1]	3.5	15.86	Translation	miRNA 21 GAGGAGACGUACGGGGAAGGU 1<br>::: ::: :: :::.::::::<br>Target 104 CUCAUCUCCAAGCCUCUUCCA 124
zma-miR528b-5p	GRMZM2G126302_T01	NA	Uncharacterized protein [Source:UniProtKB/TrEMBL;Acc:K7UJX4]	3.5	15.459	Cleavage	miRNA 20 AGGAGACGUACGGGGAAGGU 1<br>:.: ::::::.::.:::::<br>Target 735 CCUUAUGCAUGUCCUUUCCA 754
zma-miR528b-5p	GRMZM2G130548_T01	NA	HVA22-like protein a; Uncharacterized protein [Source:UniProtKB/TrEMBL;Acc:B6TLK6]	3.5	14.176	Cleavage	miRNA 20 AGGAGACGUACGGGGAAGGU 1<br>:::::::.:::::.:: ..:<br>Target 353 UCCUCUGUAUGCCUCUGUUA 372
zma-miR528b-5p	GRMZM2G130548_T02	NA	HVA22-like protein a; Uncharacterized protein [Source:UniProtKB/TrEMBL;Acc:B6TLK6]	3.5	14.176	Cleavage	miRNA 20 AGGAGACGUACGGGGAAGGU 1<br>:::::::.:::::.:: ..:<br>Target 295 UCCUCUGUAUGCCUCUGUUA 314
zma-miR528b-5p	GRMZM2G130548_T03	NA	HVA22-like protein a; Uncharacterized protein [Source:UniProtKB/TrEMBL;Acc:B6TLK6]	3.5	14.176	Cleavage	miRNA 20 AGGAGACGUACGGGGAAGGU 1<br>:::::::.:::::.:: ..:<br>Target 353 UCCUCUGUAUGCCUCUGUUA 372
zma-miR528b-5p	GRMZM2G147667_T01	NA	Ubiquitin carboxyl-terminal hydrolase [Source:UniProtKB/TrEMBL;Acc:B4FF77]	3.5	11.017	Cleavage	miRNA 21 GAGGAGACGUACGGGGAAGGU 1<br>.::: :::: ::::.:::.:.<br>Target 917 UUCCACUGCUUGCCUCUUUCG 937
zma-miR528b-5p	GRMZM2G147667_T02	NA	Ubiquitin carboxyl-terminal hydrolase [Source:UniProtKB/TrEMBL;Acc:B4FF77]	3.5	16.009	Cleavage	miRNA 21 GAGGAGACGUACGGGGAAGGU 1<br>.::: :::: ::::.:::.:.<br>Target 819 UUCCACUGCUUGCCUCUUUCG 839

Potential targets of zma-miRNAs.

miRNA	Target	Gene name	Gene description	Expectation	UPE	Inhibition	Hybrid
zma-miR528b-5p	GRMZM2G147667_T03	NA	Ubiquitin carboxyl-terminal hydrolase [Source:UniProtKB/TrEMBL;Acc:B4FF77]	3.5	14.635	Cleavage	miRNA 21 GAGGAGACGUACGGGGAAGGU 1<br>.::: :::: ::::.:::.:.<br>Target 1228 UUCCACUGCUUGCCUCUUUCG 1248
zma-miR528b-5p	GRMZM2G150912_T01	NA	endoribonucleases [Source:Projected from Arabidopsis thaliana (AT3G04480) TAIR;Acc:AT3G04480]	3.5	18.588	Cleavage	miRNA 21 GAGGAGACGUACGGGGAAGGU 1<br>:: . :::.:::.::::::::<br>Target 1826 CUAUACUGUAUGUCCCUUCCA 1846
zma-miR528b-5p	GRMZM2G161222_T03	NA	Uncharacterized protein [Source:UniProtKB/TrEMBL;Acc:B7ZXD0]	3.5	4.557	Translation	miRNA 21 GAGGAGACGUACGGGGAAGGU 1<br>:::::: :::: :::::::.<br>Target 118 CUCCUCAGCAUACCCCUUCUU 138
zma-miR528b-5p	GRMZM2G164074_T01	NA	Cytochrome P450 CYP94B12; Uncharacterized protein [Source:UniProtKB/TrEMBL;Acc:B6SRJ8]	3.5	17.228	Cleavage	miRNA 20 AGGAGACGUACGGGGAAGGU 1<br>:.:::::: ::::.: ::::<br>Target 222 UUCUCUGCCUGCCUCCUCCA 241
zma-miR528b-5p	GRMZM2G302002_T01	NA	NA	3.5	16.284	Cleavage	miRNA 21 GAGGAGACGUACGGGGAAGGU 1<br>::::::: . :::..::::::<br>Target 444 CUCCUCUUUUUGCUUCUUCCA 464
zma-miR528b-5p	GRMZM2G348100_T01	NA	Uncharacterized protein [Source:UniProtKB/TrEMBL;Acc:K7VNI6]	3.5	8.539	Translation	miRNA 21 GAGGAGACGUACGGGGAAGGU 1<br>:::: :: :: :::.::::::<br>Target 83 CUCCGCUUCAAGCCUCUUCCA 103
zma-miR528b-5p	GRMZM2G367023_T01	NA	NA	3.5	24.237	Translation	miRNA 20 AGGAGACGUA-CGGGGAAGGU 1<br>::::.::::: :::.:::::.<br>Target 143 UCCUUUGCAUCGCCUCUUCCG 163
zma-miR528b-5p	GRMZM2G466394_T01	NA	NA	3.5	13.583	Cleavage	miRNA 21 GAGGAGACGUACGGGGAAGGU 1<br>:::::.:: :::..:::::::<br>Target 2852 CUCCUUUGGUUGUUCCUUCCA 2872
zma-miR528b-5p	GRMZM2G701288_T01	NA	NA	3.5	19.072	Cleavage	miRNA 21 GAGGAGACGUACGGGGAAGGU 1<br>::::::: : ::::::: :::<br>Target 93 CUCCUCUCCCUGCCCCUCCCA 113
zma-miR528b-5p	GRMZM2G701288_T02	NA	NA	3.5	19.072	Cleavage	miRNA 21 GAGGAGACGUACGGGGAAGGU 1<br>::::::: : ::::::: :::<br>Target 93 CUCCUCUCCCUGCCCCUCCCA 113
zma-miR528b-5p	AC197082.3_FGT004	NA	NA	4	18.476	Cleavage	miRNA 20 AGGAGACGUACGGGGAAGGU 1<br>:::::::: :::: ::::.<br>Target 9 UCCUCUGCCUGCCGCUUCUC 28
zma-miR528b-5p	GRMZM2G004459_T01	NA	NA	4	17.6	Cleavage	miRNA 21 GAGGAGACGUACGGGGAAGGU 1<br>.:..:::::::::::. :..:<br>Target 921 UUUUUCUGCAUGCCCUCUUUA 941
zma-miR528b-5p	GRMZM2G004459_T02	NA	NA	4	17.6	Cleavage	miRNA 21 GAGGAGACGUACGGGGAAGGU 1<br>.:..:::::::::::. :..:<br>Target 921 UUUUUCUGCAUGCCCUCUUUA 941
zma-miR528b-5p	GRMZM2G007681_T01	NA	DNA-directed RNA polymerase [Source:UniProtKB/TrEMBL;Acc:C0JR65]	4	23.79	Cleavage	miRNA 21 GAGGAGACGUACGGGGAAGGU 1<br>::..:: ::::::..:::::<br>Target 1690 CUUUUCAGCAUGCUUCUUCCU 1710
zma-miR528b-5p	GRMZM2G007681_T02	NA	DNA-directed RNA polymerase [Source:UniProtKB/TrEMBL;Acc:C0JR65]	4	23.79	Cleavage	miRNA 21 GAGGAGACGUACGGGGAAGGU 1<br>::..:: ::::::..:::::<br>Target 1690 CUUUUCAGCAUGCUUCUUCCU 1710

Potential targets of zma-miRNAs.

miRNA	Target	Gene name	Gene description	Expectation	UPE	Inhibition	Hybrid
zma-miR528b-5p	GRMZM2G009575_T01	NA	NA	4	19.375	Cleavage	miRNA 20 AGGAGACGUACGGGGAAGGU 1<br>:::::.:::::::: :..<br>Target 1369 ACCUCUGUAUGCCCCUGCUG 1388
zma-miR528b-5p	GRMZM2G009575_T02	NA	NA	4	19.375	Cleavage	miRNA 20 AGGAGACGUACGGGGAAGGU 1<br>:::::.:::::::: :..<br>Target 1234 ACCUCUGUAUGCCCCUGCUG 1253
zma-miR528b-5p	GRMZM2G015011_T01	NA	NA	4	15.476	Cleavage	miRNA 21 GAGGAGACGUACGGGGAAGGU 1<br>.::::::.:::.. :::..:<br>Target 902 UUCCUCUGUAUGUUGCUUUUA 922
zma-miR528b-5p	GRMZM2G025227_T02	NA	NA	4	10.963	Cleavage	miRNA 20 AGGAGACGUACGGGGAAGGU 1<br>:.::::::::::..:: ::<br>Target 738 UUCUCUGCAUGCUUCUCCCC 757
zma-miR528b-5p	GRMZM2G031721_T01	NA	Uncharacterized protein [Source:UniProtKB/TrEMBL;Acc:K7TQQ1]	4	11.9	Cleavage	miRNA 21 GAGGAGACGUACGGGGAAGGU 1<br>:: :::: ::::::..::::<br>Target 2046 CUGCUCUCCAUGCCUUUUCCC 2066
zma-miR528b-5p	GRMZM2G031721_T03	NA	Uncharacterized protein [Source:UniProtKB/TrEMBL;Acc:K7TQQ1]	4	11.9	Cleavage	miRNA 21 GAGGAGACGUACGGGGAAGGU 1<br>:: :::: ::::::..::::<br>Target 1818 CUGCUCUCCAUGCCUUUUCCC 1838
zma-miR528b-5p	GRMZM2G033649_T01	NA	Putative patellin family protein [Source:UniProtKB/TrEMBL;Acc:K7V6Z1]	4	5.132	Cleavage	miRNA 21 GAGGAGACGUACGGGGAAGGU 1<br>.:.:::: :.::.::.::::<br>Target 2411 UUUCUCUUCGUGUCCUUUCCC 2431
zma-miR528b-5p	GRMZM2G039254_T01	NA	Pentatricopeptide repeat (PPR) superfamily protein [Source:Projected from Arabidopsis thaliana (AT1G71210) TAIR;Acc:AT1G71210]	4	18.312	Cleavage	miRNA 21 GAGGAGACGUACGGGGAAGGU 1<br>::::::: .:::.::: :::.<br>Target 3384 CUCCUCUAUAUGUCCCAUCCG 3404
zma-miR528b-5p	GRMZM2G039254_T02	NA	Pentatricopeptide repeat (PPR) superfamily protein [Source:Projected from Arabidopsis thaliana (AT1G71210) TAIR;Acc:AT1G71210]	4	18.312	Cleavage	miRNA 21 GAGGAGACGUACGGGGAAGGU 1<br>::::::: .:::.::: :::.<br>Target 3374 CUCCUCUAUAUGUCCCAUCCG 3394
zma-miR528b-5p	GRMZM2G039254_T03	NA	Pentatricopeptide repeat (PPR) superfamily protein [Source:Projected from Arabidopsis thaliana (AT1G71210) TAIR;Acc:AT1G71210]	4	18.312	Cleavage	miRNA 21 GAGGAGACGUACGGGGAAGGU 1<br>::::::: .:::.::: :::.<br>Target 3267 CUCCUCUAUAUGUCCCAUCCG 3287
zma-miR528b-5p	GRMZM2G041765_T01	NA	IQ calmodulin-binding motif family protein [Source:UniProtKB/TrEMBL;Acc:B6TKK9]	4	14.639	Translation	miRNA 21 GAGGAGACGUACGGGGAAGGU 1<br>: ::::::::: ::..::::<br>Target 83 CGCCUCUGCAUUCCUUUUCCU 103
zma-miR528b-5p	GRMZM2G042181_T01	NA	Leucine-rich repeat (LRR) family protein; Uncharacterized protein [Source:UniProtKB/TrEMBL;Acc:B4FAA2]	4	18.393	Cleavage	miRNA 21 GAGGAGACGUACGGGGAAGGU 1<br>:::.:.: :::::. ::::::<br>Target 491 CUCUUUUCCAUGCUGCUUCCA 511
zma-miR528b-5p	GRMZM2G042181_T02	NA	Leucine-rich repeat (LRR) family protein; Uncharacterized protein [Source:UniProtKB/TrEMBL;Acc:B4FAA2]	4	18.393	Cleavage	miRNA 21 GAGGAGACGUACGGGGAAGGU 1<br>:::.:.: :::::. ::::::<br>Target 467 CUCUUUUCCAUGCUGCUUCCA 487
zma-miR528b-5p	GRMZM2G042181_T04	NA	Leucine-rich repeat (LRR) family protein; Uncharacterized protein [Source:UniProtKB/TrEMBL;Acc:B4FAA2]	4	18.393	Cleavage	miRNA 21 GAGGAGACGUACGGGGAAGGU 1<br>:::.:.: :::::. ::::::<br>Target 467 CUCUUUUCCAUGCUGCUUCCA 487
zma-miR528b-5p	GRMZM2G057950_T01	NA	Ribosomal protein L32 containing protein isoform 1; Ribosomal protein L32 containing protein isoform 2 [Source:UniProtKB/TrEMBL;Acc:K7VLL0]	4	10.709	Cleavage	miRNA 21 GAGGAGACGUACGGGGAAGGU 1<br>::::::: : ::::::: .::<br>Target 169 CUCCUCUCCCUGCCCCUCUCA 189

Potential targets of zma-miRNAs.

miRNA	Target	Gene name	Gene description	Expectation	UPE	Inhibition	Hybrid
zma-miR528b-5p	GRMZM2G057950_T02	NA	Ribosomal protein L32 containing protein isoform 1; Ribosomal protein L32 containing protein isoform 2 [Source:UniProtKB/TrEMBL;Acc:K7VLL0]	4	10.709	Cleavage	miRNA 21 GAGGAGACGUACGGGGAAGGU 1<br>::::::: : ::::::: .::<br>Target 169 CUCCUCUCCCUGCCCCUCUCA 189
zma-miR528b-5p	GRMZM2G062641_T01	NA	Uncharacterized protein [Source:UniProtKB/TrEMBL;Acc:B4G057]	4	23.201	Cleavage	miRNA 21 GAGGAGACGUACGGGGAAGGU 1<br>.:::: :::::::: .:::..<br>Target 477 UUCCUAUGCAUGCCAUUUCUG 497
zma-miR528b-5p	GRMZM2G062641_T02	NA	Uncharacterized protein [Source:UniProtKB/TrEMBL;Acc:B4G057]	4	23.201	Cleavage	miRNA 21 GAGGAGACGUACGGGGAAGGU 1<br>.:::: :::::::: .:::..<br>Target 873 UUCCUAUGCAUGCCAUUUCUG 893
zma-miR528b-5p	GRMZM2G067036_T01	NA	Uncharacterized protein [Source:UniProtKB/TrEMBL;Acc:C4J4Z8]	4	8.755	Cleavage	miRNA 21 GAGGAGACGUACGGGGAAGGU 1<br>: ::::::: ::::.:::: :<br>Target 13 CCCCUCUGCCUGCCUCUUCAA 33
zma-miR528b-5p	GRMZM2G070708_T01	NA	Uncharacterized protein [Source:UniProtKB/TrEMBL;Acc:B4FU32]	4	16.686	Translation	miRNA 21 GAGGAGACGUACGGGGAAGGU 1<br>.::.:.:::. :..:.:::::<br>Target 1797 UUCUUUUGCGGGUUCUUUCCA 1817
zma-miR528b-5p	GRMZM2G070708_T02	NA	Uncharacterized protein [Source:UniProtKB/TrEMBL;Acc:B4FU32]	4	16.686	Translation	miRNA 21 GAGGAGACGUACGGGGAAGGU 1<br>.::.:.:::. :..:.:::::<br>Target 1704 UUCUUUUGCGGGUUCUUUCCA 1724
zma-miR528b-5p	GRMZM2G071071_T01	NA	Mitochondrial Rho GTPase [Source:UniProtKB/TrEMBL;Acc:B6U4D3]	4	17.224	Cleavage	miRNA 21 GAGGAGACGUACGGGGAAGGU 1<br>.:..:::::::::::. :..:<br>Target 990 UUUUUCUGCAUGCCCUAUUUA 1010
zma-miR528b-5p	GRMZM2G078656_T02	NA	NA	4	18.552	Cleavage	miRNA 21 GAGGAGACGUACGGGGAAGGU 1<br>:::::.:: .:::.:.::::<br>Target 557 CUCCUUUGGGUGCUCUUUCCC 577
zma-miR528b-5p	GRMZM2G095839_T01	NA	Uncharacterized protein [Source:UniProtKB/TrEMBL;Acc:B4FLL0]	4	15.19	Cleavage	miRNA 21 GAGGAGACGUACGGGGAAGGU 1<br>::: ::: :.::...::::::<br>Target 80 CUCGUCUUCGUGUUUCUUCCA 100
zma-miR528b-5p	GRMZM2G123411_T01	NA	Uncharacterized protein [Source:UniProtKB/TrEMBL;Acc:B6UGS7]	4	22.767	Translation	miRNA 21 GAGGAGACGUACGGGGAAGGU 1<br>:::::.: :::: ..::::.:<br>Target 106 CUCCUUUCCAUGGUUCUUCUA 126
zma-miR528b-5p	GRMZM2G123411_T02	NA	Uncharacterized protein [Source:UniProtKB/TrEMBL;Acc:B6UGS7]	4	22.767	Translation	miRNA 21 GAGGAGACGUACGGGGAAGGU 1<br>:::::.: :::: ..::::.:<br>Target 106 CUCCUUUCCAUGGUUCUUCUA 126
zma-miR528b-5p	GRMZM2G136534_T01	NA	Putative uncharacterized protein [Source:UniProtKB/TrEMBL;Acc:B6U210]	4	7.032	Cleavage	miRNA 21 GAGGAGACGUACGGGGAAGGU 1<br>::::::::. ::...:::::<br>Target 1349 CUCCUCUGUUUGUUUCUUCCU 1369
zma-miR528b-5p	GRMZM2G136534_T02	NA	Putative uncharacterized protein [Source:UniProtKB/TrEMBL;Acc:B6U210]	4	7.032	Cleavage	miRNA 21 GAGGAGACGUACGGGGAAGGU 1<br>::::::::. ::...:::::<br>Target 1774 CUCCUCUGUUUGUUUCUUCCU 1794
zma-miR528b-5p	GRMZM2G146041_T01	NA	switch 2 [Source:Projected from Arabidopsis thaliana (AT1G03750) TAIR;Acc:AT1G03750]	4	15.427	Cleavage	miRNA 21 GAGGAGACGUACGGGGAAGGU 1<br>:: .::: .::::::::::..<br>Target 1432 CUGUUCUAUAUGCCCCUUCUG 1452
zma-miR528b-5p	GRMZM2G146041_T02	NA	switch 2 [Source:Projected from Arabidopsis thaliana (AT1G03750) TAIR;Acc:AT1G03750]	4	15.427	Cleavage	miRNA 21 GAGGAGACGUACGGGGAAGGU 1<br>:: .::: .::::::::::..<br>Target 1432 CUGUUCUAUAUGCCCCUUCUG 1452

Potential targets of zma-miRNAs.

miRNA	Target	Gene name	Gene description	Expectation	UPE	Inhibition	Hybrid
zma-miR528b-5p	GRMZM2G146041_T03	NA	switch 2 [Source:Projected from Arabidopsis thaliana (AT1G03750) TAIR;Acc:AT1G03750]	4	15.427	Cleavage	miRNA 21 GAGGAGACGUACGGGGAAGGU 1<br>:: .::: .:::::::::..<br>Target 1621 CUGUUCUAUAUGCCCCUUCUG 1641
zma-miR528b-5p	GRMZM2G156388_T01	NA	RAB	4	20.759	Translation	miRNA 21 GAGGAGACGUACGGGGAAGGU 1<br>:::::.: :.: .::.::::<br>Target 1428 CUCCUUUUCGUUUCCUUUCCA 1448
zma-miR528b-5p	GRMZM2G156388_T02	NA	RAB	4	20.759	Translation	miRNA 21 GAGGAGACGUACGGGGAAGGU 1<br>:::::.: :.: .::.::::<br>Target 1517 CUCCUUUUCGUUUCCUUUCCA 1537
zma-miR528b-5p	GRMZM2G162112_T01	NA	Uncharacterized protein [Source:UniProtKB/TrEMBL;Acc:K7VWF7]	4	9.072	Cleavage	miRNA 21 GAGGAGACGUACGGGGAAGGU 1<br>: :: :::::::..::::::<br>Target 190 CGCCGCUGCAUGUUCCUUCCC 210
zma-miR528b-5p	GRMZM2G341410_T01	NA	NA	4	4.306	Translation	miRNA 21 GAGGAGACGUACGGGGAAGGU 1<br>::::::::: : :.::: :::<br>Target 382 CUCCUCUGCCUCCUCCUCCCA 402
zma-miR528b-5p	GRMZM2G404132_T01	NA	NA	4	7.281	Cleavage	miRNA 21 GAGGAGACGUACGGGGAAGGU 1<br>::::::::..:::.:::: :<br>Target 15 CUCCUCUGUGUGCUCCUUGCC 35
zma-miR528b-5p	GRMZM2G510796_T01	NA	Uncharacterized protein [Source:UniProtKB/TrEMBL;Acc:K7W3D5]	4	18.528	Cleavage	miRNA 20 AGGAGACGUACGGGGAAGGU 1<br>:::: :.::::.:.::.:<br>Target 175 UCCUCAGUAUGCUCUUUUCC 194
zma-miR528b-5p	GRMZM5G816274_T01	NA	Putative uncharacterized protein orf158-a1; Putative uncharacterized protein orf158-a2;<br>Uncharacterized protein [Source:UniProtKB/TrEMBL;Acc:Q6R9K1]	4	9.982	Cleavage	miRNA 20 AGGAGACGUACGGGGAAGGU 1<br>..::: :.:::::::::.<br>Target 207 AUUUCUCCGUGCCCCUUCCG 226
zma-miR528b-5p	GRMZM5G846550_T01	NA	NA	4	18.912	Cleavage	miRNA 21 GAGGAGACGUACGGGGAAGGU 1<br>::.:::: :::::..::::.<br>Target 644 CUUCUCUUCAUGCUUCUUCUU 664
zma-miR528b-5p	GRMZM5G867983_T01	NA	NA	4	20.692	Translation	miRNA 20 AGGAGACGUACGGGGAAGGU 1<br>:::::::: ..::::.:.<br>Target 190 CCCUCUGCAUUUUCCUUUCG 209
zma-miR528b-5p	GRMZM5G885061_T01	NA	NA	4	10.991	Cleavage	miRNA 21 GAGGAGACGUACGGGGAAGGU 1<br>.:: :.::..::.:.:::.::<br>Target 414 UUCAUUUGUGUGUCUCUUUCA 434
zma-miR528b-5p	GRMZM5G897988_T01	NA	Ataxin-3; Uncharacterized protein [Source:UniProtKB/TrEMBL;Acc:B4FXQ4]	4	18.498	Translation	miRNA 21 GAGGAGACGUACGGGGAAGGU 1<br>::::::::.: ::.::::<br>Target 1 CUCCUCUGCGUUACCUUUCCC 21
zma-miR528b-5p	GRMZM5G897988_T02	NA	Ataxin-3; Uncharacterized protein [Source:UniProtKB/TrEMBL;Acc:B4FXQ4]	4	19.85	Translation	miRNA 21 GAGGAGACGUACGGGGAAGGU 1<br>::::::::.: ::.::::<br>Target 4 CUCCUCUGCGUUACCUUUCCC 24
zma-miR528b-5p	GRMZM5G898772_T01	NA	Putative uncharacterized protein orf158-a1; Putative uncharacterized protein orf158-a2;<br>Uncharacterized protein [Source:UniProtKB/TrEMBL;Acc:Q6R9K1]	4	9.982	Cleavage	miRNA 20 AGGAGACGUACGGGGAAGGU 1<br>..::: :.:::::::::.<br>Target 207 AUUUCUCCGUGCCCCUUCCG 226
zma-miR528b-5p	GRMZM2G013187_T01	NA	NA	4.5	13.451	Cleavage	miRNA 21 GAGGAGACGUACGGGGAAGGU 1<br>::::::::.:::.:: :..<br>Target 106 CUCCUCUGCGUGCUCCCUUUU 126

Potential targets of zma-miRNAs.

miRNA	Target	Gene name	Gene description	Expectation	UPE	Inhibition	Hybrid
zma-miR528b-5p	GRMZM2G020666_T01	NA	Uncharacterized protein [Source:UniProtKB/TrEMBL;Acc:K7VI07]	4.5	17.547	Cleavage	miRNA 21 GAGGAGACGUACGGGGAAGGU 1<br>::..:::::::::.:. :..:<br>Target 1642 CUUUUCUGCAUGCUCUAUUUA 1662
zma-miR528b-5p	GRMZM2G025855_T01	NA	Ribosomal protein S5 family protein [Source:Projected from Arabidopsis thaliana (AT2G33800) TAIR;Acc:AT2G33800]	4.5	13.949	Translation	miRNA 21 GAGGAGACGUACGGGGAAGGU 1<br>::..:.:::: .:.::::..<br>Target 1195 CUUUUUUGCAUCUCUCUUCUG 1215
zma-miR528b-5p	GRMZM2G061078_T05	NA	Receptor expression-enhancing protein 3; Uncharacterized protein [Source:UniProtKB/TrEMBL;Acc:B4FTU0]	4.5	15.281	Cleavage	miRNA 21 GAGGAGACGUACGGGGAAGGU 1<br>::.: .:::::::.:.:::.<br>Target 692 CUUCCUUGCAUGCUCUUUCUC 712
zma-miR528b-5p	GRMZM2G086541_T01	NA	Uncharacterized protein [Source:UniProtKB/TrEMBL;Acc:K7TMP2]	4.5	16.73	Cleavage	miRNA 21 GAGGAGACGUACGGGGAAGGU 1<br>::.::::. :::..:::.:<br>Target 153 CUUCUCUGUUUGCUUCUUUCU 173
zma-miR528b-5p	GRMZM2G108416_T01	NA	Aminotransferase	4.5	12.69	Cleavage	miRNA 21 GAGGAGACGUACGGGGAAGGU 1<br>:::::::: ::.:::: : :<br>Target 1058 CUCCUCUGCCUGUCCCUGCAA 1078
zma-miR528b-5p	GRMZM2G124151_T01	NA	Uncharacterized protein [Source:UniProtKB/TrEMBL;Acc:B4G1B2]	4.5	15.035	Cleavage	miRNA 21 GAGGAGACGUACGGGGAAGGU 1<br>:: :::::::::. ::::.<br>Target 432 CUACUCUGCAUGCUACUUCUU 452
zma-miR528b-5p	GRMZM2G124151_T02	NA	Uncharacterized protein [Source:UniProtKB/TrEMBL;Acc:B4G1B2]	4.5	15.035	Cleavage	miRNA 21 GAGGAGACGUACGGGGAAGGU 1<br>:: :::::::::. ::::.<br>Target 479 CUACUCUGCAUGCUACUUCUU 499
zma-miR528b-5p	GRMZM2G133444_T01	NA	Uncharacterized protein [Source:UniProtKB/TrEMBL;Acc:C0PHW7]	4.5	12.398	Cleavage	miRNA 21 GAGGAGACGUACGGGGAAGGU 1<br>.:.::.::.::::.:. ::.:<br>Target 1562 UUUCUUUGUAUGCUCUCUCUA 1582
zma-miR528b-5p	GRMZM2G135286_T01	NA	Uncharacterized protein [Source:UniProtKB/TrEMBL;Acc:K7TGP8]	4.5	21.403	Translation	miRNA 21 GAGGAGACGUACGGGGAAGGU 1<br>:::.:::::.:: .::: ::.<br>Target 1641 CUCUUCUGCGUGAUCCUGCCG 1661
zma-miR528b-5p	GRMZM2G136367_T05	NA	Uncharacterized protein [Source:UniProtKB/TrEMBL;Acc:B4FAR6]	4.5	21.018	Cleavage	miRNA 20 AGGAGACGUACGGGGAAGGU 1<br>::::::..::..:::::.:<br>Target 1301 GCCUCUGUGUGUUCCUUUCC 1320
zma-miR528b-5p	GRMZM2G142241_T01	NA	Uncharacterized protein [Source:UniProtKB/TrEMBL;Acc:K7VIL8]	4.5	11.427	Cleavage	miRNA 21 GAGGAGACGUACGGGGAAGGU 1<br>:::::::::.:::.:: :..<br>Target 106 CUCCUCUGCGUGCUCCCUUUU 126
zma-miR528b-5p	GRMZM2G142685_T01	NA	Uncharacterized protein [Source:UniProtKB/TrEMBL;Acc:K7TSM8]	4.5	19.598	Translation	miRNA 21 GAGGAGACGUACGGGGAAGGU 1<br>:::.:::::.:: .::: ::.<br>Target 1648 CUCUUCUGCGUGAUCCUGCCG 1668
zma-miR528b-5p	GRMZM2G146152_T01	NA	Putative laccase family protein [Source:UniProtKB/TrEMBL;Acc:K7UTI2]	4.5	18.441	Cleavage	miRNA 21 GAGGAGACGUACGGGGAAGGU 1<br>:::: ::::.::::.: :.:.<br>Target 119 CUCCCCUGCGUGCCUCCUUCG 139
zma-miR528b-5p	GRMZM2G146240_T01	NA	Putative uncharacterized protein [Source:UniProtKB/TrEMBL;Acc:B6TDY6]	4.5	24.389	Cleavage	miRNA 21 GAGGAGACGUACGGGGAAGGU 1<br>:::::: ::::::.:: :::<br>Target 1612 CUCCUCCGCAUGCUCCCGCCA 1632
zma-miR528b-5p	GRMZM2G154648_T04	NA	Uncharacterized protein [Source:UniProtKB/TrEMBL;Acc:B4F9A7]	4.5	12.559	Cleavage	miRNA 21 GAGGAGACGUACGGGGAAGGU 1<br>:::::.: ::::.:.: :.::<br>Target 2199 CUCCUUUACAUGUCUCCUUCA 2219

Potential targets of zma-miRNAs.

miRNA	Target	Gene name	Gene description	Expectation	UPE	Inhibition	Hybrid
zma-miR528b-5p	GRMZM2G155340_T01	NA	Uncharacterized protein [Source:UniProtKB/TrEMBL;Acc:B4F9C2]	4.5	20.802	Cleavage	miRNA 21 GAGGAGACGUACGGGGAAGGU 1<br>::::: :::::::::.: .:<br>Target 696 CUCCUGUGCAUGCCCUUGUCC 716
zma-miR528b-5p	GRMZM2G155340_T03	NA	Uncharacterized protein [Source:UniProtKB/TrEMBL;Acc:B4F9C2]	4.5	20.802	Cleavage	miRNA 21 GAGGAGACGUACGGGGAAGGU 1<br>::::: :::::::::.: .:<br>Target 674 CUCCUGUGCAUGCCCUUGUCC 694
zma-miR528b-5p	GRMZM2G319138_T01	NA	Uncharacterized protein [Source:UniProtKB/TrEMBL;Acc:K7TN88]	4.5	12.995	Cleavage	miRNA 21 GAGGAGA-CGUACGGGGAAGGU 1<br>::::::: :::::..::::: :<br>Target 4547 CUCCUCUAGCAUGUUCCUUCAA 4568
zma-miR528b-5p	GRMZM2G466543_T01	NA	Putative uncharacterized protein [Source:UniProtKB/TrEMBL;Acc:B6UD01]	4.5	10.441	Cleavage	miRNA 21 GAGGAGACGUACGGGGAAGGU 1<br>:::. ::::::::...:::.<br>Target 1932 CUCUCCUGCAUGCUUUUUCUU 1952
zma-miR528b-5p	GRMZM2G700065_T01	NA	NA	4.5	14.864	Cleavage	miRNA 21 GAGGAGACGUACGGGGAAGGU 1<br>:::: ::::.:::.:: ::..<br>Target 32 CUCCCCUGCGUGCUCCCUCUG 52
zma-miR528b-5p	GRMZM5G872443_T01	NA	Uncharacterized protein [Source:UniProtKB/TrEMBL;Acc:C0P8T5]	4.5	24.554	Cleavage	miRNA 21 GAGGAGACGUACGGGGAAGGU 1<br>:::.::: ::::..::: ::.<br>Target 2161 CUCUUCUUCAUGUUCCUGCCG 2181
zma-miR528b-5p	GRMZM2G072911_T01	NA	nucleotide-rhamnose synthase/epimerase-reductase [Source:Projected from Arabidopsis thaliana (AT1G63000) TAIR;Acc:AT1G63000]	5	16.843	Translation	miRNA 21 GAGGAGACGUACGGGGAAGGU 1<br>::::::: :. ::::::::<br>Target 162 CUCCUCUCCGCGCCCCUUCAU 182
zma-miR528b-5p	GRMZM2G168428_T02	NA	Uncharacterized protein [Source:UniProtKB/TrEMBL;Acc:C0HG68]	5	15.312	Cleavage	miRNA 21 GAGGAGACGUACGGGGAAGGU 1<br>:::.:.::..::....::::<br>Target 1584 CUCUUUUGUGUGUUUUUUCCC 1604
