# Supplementary material for: Maize miRNA and target regulation in response to hormone depletion and light exposure during somatic embryogenesis
Source: Front Plant Sci. 2015 Jul 22;6:555. doi: 10.3389/fpls.2015.00555 (PMC4510349; doi:10.3389/fpls.2015.00555)
Supplement: Supplementary file 3 [file Table3.PDF]

Table S3. Primers used for qRT-PCR analysis of miRNA targets.
Target cDNA sequence was retrieved from Ensembl (http://plants.ensembl.org). Primers were designed with Primer3Plus (Untergasser et al., 2007).

ID	Primers
GRMZM2G126018_T01	Forward 5’ ACACCAACGCGATGAATTGG
	Reverse 5’ ACCCTGAAAAACCAGAACGG
GRMZM2G139688_T01	Forward 5’CAGGCAGGCTTTAGGTTTTG
	Reverse 5’CAGATTCACTGAAGGGGTCG
GRMZM2G393433_T01	Forward 5’TTCGCTGCACTACATGGTTG
	Reverse 5’AACGACGACCCAGTCACTTAC
GRMZM2G039455_T01	Forward 5’GGGAGGTAGTGTTGGACGAA
	Reverse 5’TGCTCAAACTGTTGCTGGAC
GRMZM2G146152_T01	Forward 5’ AATCGTTTGGCCTGTGCAAC
	Reverse 5’ ATGGCGCAACAACACATGAC
GRMZM2G058522_T01	Forward 5’-TTTCACCCAAGAGGGAGATG
	Reverse 5’-TTGCTCGCAGGATTGTAGTG
GRMZM2G384327_T03	Forward 5’ AAGTCTGTGCAAGCTGATGC
	Reverse 5’ AAGCTTGCCAGTTTGTCAGC
GRMZM2G106928_T01	Forward 5’-CAATGGGTGCATATCGACAG
	Reverse 5’-GCAACAATGTTTCCCAGGTC
GRMZM2G107562_T01	Forward 5’-TGGCCGTATGAGCATTTAGC
	Reverse 5’-GATGTTCCCGAAGCAAAGTC
