# Supplementary material for: Maize miRNA and target regulation in response to hormone depletion and light exposure during somatic embryogenesis
Source: Front Plant Sci. 2015 Jul 22;6:555. doi: 10.3389/fpls.2015.00555 (PMC4510349; doi:10.3389/fpls.2015.00555)
Supplement: Supplementary file 4 [file DataSheet1.PDF]

Supplementary methods.

Medium used for maize SE.

Callus initiation medium (N6I)

The callus initiation medium contained N6 salts (Chu et al., 1975) and cocktail 20 vitamins (Loza-Rubio et al., 2008), 2 mg L^{-1} 2,4-dichlorophenoxyacetic acid (2,4-D), 10 mg L^{-1} adenine, 2.76 g L^{-1} proline, 200 mg L^{-1} casein hydrolysate, 30 g L^{-1} sucrose, and 3.3 g L^{-1} gelrite (Sigma-Aldrich). The pH was adjusted to 5.7

Callus proliferation medium (N6P)

The callus proliferation medium contained N6 salts (Chu et al., 1975) and cocktail 20 vitamins (Loza-Rubio et al., 2008), 2 mg L^{-1} 2,4-dichlorophenoxyacetic acid (2,4-D), 0.1 mg L^{-1} kinetin, 10 mg L^{-1} adenine, 2.76 g L^{-1} proline, 200 mg L^{-1} casein hydrolysate, 30 g L^{-1} sucrose. The pH was adjusted to 5.7.

MS medium

MS salts and vitamins (Murashige & Skoog, 1962), 30 g L^{-1} sucrose, and 3.3 g L^{-1} gelrite (Sigma-Aldrich). The pH was adjusted to 5.7.

Potential targets of zma-miRNAs (Table S2)

psRNAtarget (http://plantgrn.noble.org/psRNATarget) was used to predict potential targets of zma-miR156a, zma-miR159a, zma-miR164a, zma-miR168a, zma-miR397a, zma-miR398a, zma-miR408a and zma-miR528a. The genomic library used to search the potential targets was: 'Zea mays (maize), transcript, NSF-funded Maize Genome Sequencing Project, Release 5a, filtered set'. The maximum score was set at 5. The column "Expectation" refers to the score of the complementarity between miRNA and their target transcript, minimum values are better. "UPE" (Unpair energy) is the energy required to open secondary structure around target site; the less energy the more possibility that miRNA is able to cleave target mRNA. "Inhibition", refers to the predicted mechanism of inhibition that the miRNA has over its target (Cleavage or Translational inhibition).
