# Supplementary material for: Maize miRNA and target regulation in response to hormone depletion and light exposure during somatic embryogenesis
Source: Front Plant Sci. 2015 Jul 22;6:555. doi: 10.3389/fpls.2015.00555 (PMC4510349; doi:10.3389/fpls.2015.00555)

Figure S1. Northern Blots for selected miRNAs. (A) VS-535 and (B) H-565. Samples of embryogenic callus were taken at 100% hormones (a); 50% hormones (b, d); 0% hormones (c, e); and regenerated plantlet (f). The signal was normalized by the rRNA 5S as control. The baseline was set according to the signal of 100% hormones and numbers refer to fold change with respect to the baseline.

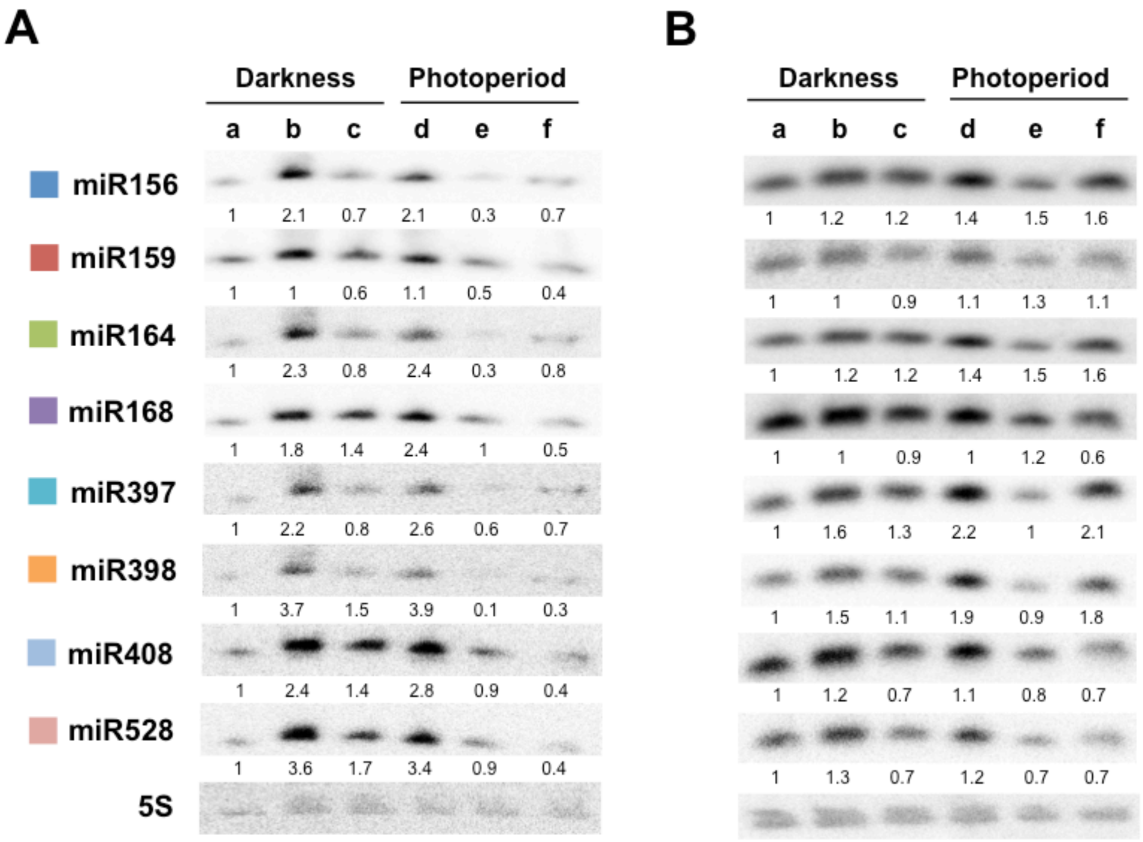

Supplement: Supplementary file 5 [file Image1.PDF]
